# Safety and efficacy of the monoclonal antibody L9LS for malaria prevention in children exposed to perennial malaria transmission in Kenya: a randomised, double-blind, placebo-controlled, phase 2 trial

**DOI:** 10.1016/S0140-6736(26)00258-8

**Published:** 2026-04-25

**Authors:** Laura C Steinhardt, Titus K Kwambai, Martina Oneko, Eunice Ouma, Ruth Njoroge, Viviane Callier, Zonghui Hu, Julie R Gutman, Reuben Yego, Kephas Otieno, Kelvin Onoka, Lilian Otieno, Kennedy Oduol, Leonid Serebryannyy, Bob C Lin, Will Adams, Somia Hickman, Anne C Preston, Kevin Carlton, Michael Holdsworth, Yan Xiao, Feiko O ter Kuile, Wycliffe Odongo, Sean C Murphy, Tuan M Tran, Simon Kariuki, Peter D Crompton, Robert A Seder, the Kenya Malaria mAb Trials Team

**Affiliations:** **Malaria Branch, National Center for Emerging and Zoonotic Infectious Diseases, US Centers for Disease Control and Prevention, Atlanta, GA, USA** (L C Steinhardt PhD, J R Gutman MD, W Odongo MSc)**;Malaria Branch, National Center for Emerging and Zoonotic Infectious Diseases, US Centers for Disease Control and Prevention, Kisumu, Kenya** (T K Kwambai MD)**; Kenya Medical Research Institute, Global Health Research Centre, Kisumu, Kenya** (M Oneko MD, E Ouma MD, R Njoroge BPharm, R Yego MSc, K Otieno MSc, K Onoka MSc, L Otieno BA, K Oduol BA, S Kariuki MD)**;Clinical Monitoring Research Program Directorate, Frederick National Laboratory, Frederick, MD, USA** (V Callier PhD)**; Office of Biostatistics Research,Division of Clinical Research, National Institute of Allergy and Infectious Diseases, National Institutes of Health, Rockville, MD, USA** (Z Hu PhD)**;Vaccine Research Center, National Institute of Allergy and Infectious Diseases, National Institutes of Health, Bethesda, MD, USA** (L Serebryannyy PhD, B C Lin BS, W Adams MPH, S Hickman PhD, K Carlton PhD, R A Seder MD)**;Malaria Infection Biology and Immunity Section, Laboratory of Immunogenetics, Division of Intramural Research, National Institute of Allergy and Infectious Diseases, National Institutes of Health, Rockville, MD, USA** (A C Preston RN,P D Crompton MD)**; Office of Cyber Infrastructure and Computational Biology, National Institute of Allergy and Infectious Diseases, National Institutes of Health, Rockville, MD, USA** (M Holdsworth, Y Xiao BS)**; Department of Clinical Sciences, Liverpool School of Tropical Medicine, Liverpool, UK** (Prof F O ter Kuile MD)**; Malaria Molecular Diagnostic Laboratory, Department of Laboratory Medicine and Pathology, and the Center for Emerging and Re-emerging Infectious Diseases, University of Washington, Seattle, WA, USA** (S C Murphy MD)**; Division of Infectious Diseases, Department of Medicine and Ryan White Center for Pediatric Infectious Disease and Global Health, Department of Pediatrics, Indiana University School of Medicine, Indianapolis, IN, USA** (T M Tran MD)

## Abstract

**Background:**

Malaria remains a major cause of mortality globally, especially among young children in sub-Saharan Africa. The long-acting monoclonal antibody L9LS has shown high efficacy in preventing malaria in children aged 6–10 years exposed to seasonal transmission but remains untested in perennial transmission settings and younger children. We assessed the safety, tolerability, and efficacy of L9LS in infants and children in a high perennial malaria transmission setting.

**Methods:**

This double-blind, two-part, randomised, placebo-controlled, phase 2 trial was done in Siaya county in western Kenya. In parts 1a and 1b, we tested the safety and tolerability of L9LS using an age de-escalation and dose escalation approach and randomly assigned (3:1) cohorts of healthy children (three cohorts aged 5–10 years, three cohorts aged 5–59 months, and two cohorts aged 5–71 months) to L9LS at doses of 5, 10, 20, 30, or 40 mg/kg subcutaneously or to placebo (normal saline). In part 2, healthy children aged 5–59 months were randomly assigned (1:1:1) by use of centralised computer-generated lists to receive two doses of L9LS at 10–20 mg/kg at baseline and month 6, one dose of L9LS at baseline and placebo at month 6, or placebo at both timepoints. Children were followed up for 12 months with monthly clinic visits and blood smear collections. Primary safety outcomes were incidence and severity of local and systemic solicited adverse events within 7 days of dosing and serious adverse events throughout follow-up. The primary efficacy endpoint was *Plasmodium falciparum* infection detected by blood smear over 12 months. Primary analyses were done in the modified intention-to-treat population, consisting of all randomly assigned participants who received the study intervention. This trial is registered with ClinicalTrials.gov (NCT05400655) and is complete.

**Findings:**

In parts 1a and 1b, 96 children were enrolled and randomly assigned between Oct 1, 2022, and Jan 16, 2024; 72 participants were assigned to L9LS and 24 were assigned to placebo. In part 2, 324 children aged 5–59 months were enrolled and randomly assigned between Jan 26 and June 2, 2023; 108 children were assigned to one-dose L9LS, 106 to two-dose L9LS, and 110 to placebo. Across all study parts, grade 3 or worse treatment-related adverse events occurred after four (1%) of 384 L9LS injections and two (1%) of 338 placebo injections; these events all resolved by study end. The proportion of solicited and unsolicited adverse events was similar across all L9LS dose groups. There were no serious adverse events related to the trial. In part 2, 70 (66%) of 106 children in the two-dose L9LS group had at least one *P falciparum* infection during the 12-month follow-up versus 91 (83%) of 110 children in the placebo group (protective efficacy 42·7%, 95% CI 22·5–57·7; p=0·0003).

**Interpretation:**

L9LS was protective against malaria in young children in western Kenya without evident safety concerns over 6–12 months. A higher dose of L9LS might be needed to achieve high-level efficacy against malaria in young children exposed to intense perennial *P falciparum* transmission.

## Introduction

Malaria caused 282 million clinical cases and 610 000 deaths globally in 2024, with children younger than 5 years accounting for approximately three-quarters of all malaria deaths.^1^ Despite substantial reductions in the burden of malaria between 2000 and 2015, progress has stalled during the past decade, and efforts to reverse this trend are threatened by the emergence and spread of insecticide-resistant mosquitoes^2^ and drug-resistant *Plasmodium falciparum* parasites.^3^ Although recently approved malaria vaccines offer important advances, they require multiple doses to generate and maintain protection, and their efficacy varies with the age of recipient. Thus, there is an urgent need for complementary, long-acting interventions that provide immediate, high-level protection to populations of any age who are at high risk of malaria for defined periods.

Antimalaria monoclonal antibodies represent a potentially transformative new intervention that can directly neutralise sporozoites, the infectious form of the malaria parasite that mosquitoes inject into the skin and blood, preventing malaria infection before liver-stage development.^4–6^ Unlike vaccines, which require the host to induce an effective immune response after multiple immunisations, monoclonal antibodies can provide immediate malaria prevention with a single administration for up to 6 months.^7,8^ As a result, monoclonal antibodies could be particularly valuable as a potential replacement for seasonal malaria chemoprevention in children, which requires monthly dosing; for protecting infants before they become eligible for multidose malaria vaccines at 5–6 months of age; and for other high-risk groups such as immunocompromised individuals, pregnant women, and children discharged after hospital admission for severe anaemia or severe malaria, who have an increased risk of fatal reinfection.^9^

Three antimalaria monoclonal antibodies (MAM01, CIS43LS, and L9LS) targeting different regions of the *P falciparum* circumsporozoite protein, the most abundant antigen expressed on sporozoites, are in clinical development.^10–12^ Phase 1 and 2 field trials in Africa have shown that CIS43LS and L9LS are safe and highly efficacious against *P falciparum* infection. In a highly seasonal transmission setting in Mali, CIS43LS provided 75% efficacy against infection at a dose of 10 mg/kg and 88% efficacy at a dose of 40 mg/kg when administered intravenously to Malian adults,^7^ while L9LS showed 70% efficacy against infection and 77% efficacy against clinical malaria following a single 300 mg dose administered subcutaneously to Malian children aged 6–10 years.^8^ Because L9LS demonstrated superior potency compared with CIS43LS in mouse models and showed more favourable characteristics for product development,^13^ it was prioritised for further clinical development and evaluation in additional settings and age groups.

This trial was designed to address several questions in the development of monoclonal antibodies for key at-risk populations: first, whether monoclonal antibodies can maintain efficacy in areas of intense perennial transmission, where continuous *P falciparum* exposure might overwhelm protection or potentially accelerate monoclonal antibody clearance; second, whether protective efficacy can be achieved in infants and young children—the population bearing the highest malaria burden; and third, whether repeated monoclonal antibody dosing can extend protection to 12 months. To address these crucial knowledge gaps, this phase 2 trial assessed the safety of L9LS in children aged 5 months to 10 years, and the 12-month efficacy of one or two doses of L9LS given subcutaneously to infants and young children aged 5–59 months in western Kenya, where intense perennial *P falciparum* transmission provides a stringent test of monoclonal antibody efficacy.

## Methods

### Study design

We did a double-blind, two-part, randomised, placebo-controlled, phase 2 trial in children aged 5 months to 10 years to assess the safety and efficacy of the antimalarial monoclonal antibody L9LS in an area of western Kenya that has intense perennial malaria transmission with seasonal peaks during the long rains (April–July) and short rains (November–December). The trial comprised three sequential components. In part 1a, we did an age de-escalation, dose escalation safety and tolerability evaluation of L9LS administered subcutaneously at a single dose of 5, 10, or 20 mg/kg in children aged 5–10 years followed by children aged 5–59 months. In part 2, we recruited additional children to assess efficacy of a single dose or two doses of L9LS at 10–20 mg/kg administered 6 months apart in children aged 5–59 months during 12 months of follow-up. The dose range for this trial was based on data from previous phase 1 and 2 trials. In a phase 1 trial, 15 (88%) of 17 malaria-naive, healthy adults who received L9LS either intravenously or subcutaneously at a dose of 1 mg/kg, 5 mg/kg, or 20 mg/kg were protected against *P falciparum* infection after controlled human malaria infection.^12^ In a phase 2 trial in children aged 6–10 years in Mali, efficacy of L9LS against clinical malaria at doses of 150 mg or 300 mg subcutaneously (weight-based dose range 5–20 mg/kg) was 67% and 77%, respectively, over 6 months of follow-up; exploratory analyses suggested that approximately 12 mg/kg of L9LS corresponded to protective efficacy of approximately 75% over 6 months.^8^ On the basis of these clinical data, a dose of 10–20 mg/kg was chosen for the part 2 assessment of efficacy against malaria over 6 months. Part 1b was added through a protocol amendment to evaluate the safety, tolerability, and pharmacokinetics of 30 mg/kg and 40 mg/kg of L9LS in children aged 5–71 months to inform future trials that would potentially assess efficacy endpoints up to 12 months of follow-up with a single dose.

This study took place in Siaya county in western Kenya, which has a high entomological inoculation rate estimated in 2019 to be 16–30 infectious bites per person per month.^14^ Previous studies in the area have shown an incidence of two to five episodes of clinical malaria per child per year in children aged 5–17 months^15^ and incident parasitaemia of 66·2% in children aged 5–12 months during 6 months of follow-up.^16^

The study protocol was approved by the institutional review boards of the Kenya Medical Research Institute (Scientific and Ethics Research Unit; number 4413), the US Centers for Disease Control and Prevention, and the Liverpool School of Tropical Medicine, with regulatory review by the Kenya Pharmacy and Poisons Board (ECCT/22/05/03). A data and safety monitoring board reviewed the trial protocol, consent documents, and adverse event summaries, and conducted an interim safety review after day 7 data were available for all participants in part 1a before recommending progression to part 2, and then twice yearly thereafter. The study design was also shared with community advisory boards for their input. The full study protocol is available in the appendix (pp 64–193). This study is registered with ClinicalTrials.gov (NCT05400655) and is complete.

### Participants

Healthy children were recruited from the catchment area of Siaya County Referral Hospital, the main tertiary hospital in Siaya county, western Kenya. For part 1a, three cohorts of 12 children each in the older age group (aged 5–10 years at the time of dosing) and three cohorts of 12 children each in the younger age group (aged 5–59 months) were randomly assigned to receive L9LS (at doses of 5, 10, or 20 mg/kg) or placebo (normal saline) in a dose escalating, age de-escalating design (appendix p 16), with subsequent cohorts enrolled after confirming that no concerning safety signals, including abnormal laboratory results, had occurred within 7 days after dosing in the previous cohort(s). For part 1b, two cohorts of 12 children aged 5–71 months were randomly assigned to 30 mg/kg L9LS, 40 mg/kg L9LS, or placebo. For part 2, children aged 5–59 months were recruited from the catchment areas of Siaya County Referral Hospital and Kogelo Dispensary, a satellite clinic located 17 km away.

Eligibility criteria for all study parts included HIVnegative status, absence of sickle cell disease, no wasting or stunting, no killed or live vaccine within 14 days (changed from 28 days in a protocol amendment in October, 2023) before study agent administration, no receipt of malaria vaccine, and normal blood counts and chemistry values. A full list of inclusion and exclusion criteria is in the appendix (pp 2–3). Parents or guardians provided written informed consent for their child’s participation.

### Randomisation and masking

In parts 1a and 1b, within each age-dose cohort, participants were randomly assigned (3:1) to L9LS or placebo by use of permuted block randomisation in R with block sizes of four. In part 2, randomisation was stratified according to participant age, so that there was one cohort for children aged 5–17 months and one cohort for children aged 18–59 months. Within each age stratum and site, participants were randomly assigned (1:1:1) to two doses of L9LS (L9LS at baseline and 6 months), one dose of L9LS (L9LS at baseline and placebo at 6 months), or placebo at baseline and 6 months, by use of permuted block randomisation with block sizes of six and nine.

An unmasked trial statistician (VC) provided computer-generated randomisation lists that were shared only with unmasked pharmacy staff, who sequentially assigned eligible participants at their dosing visit. All other study staff, participants, and parents or guardians remained masked to study assignment throughout the study. Randomisation lists were secured in a locked pharmacy cabinet. To maintain blinding, syringes were covered with transparent yellow tape to mask the yellowish tint of L9LS versus the colourless placebo. Additionally, since L9LS is more viscous than normal saline, dedicated nurses administered study product separately from the masked team conducting follow-up assessments. All study staff, apart from the unmasked statistician and pharmacy team, remained masked to treatment assignment until database lock in January, 2025.

### Procedures

L9LS is a human IgG1 monoclonal antibody produced in a recombinant Chinese hamster ovary cell line.^12,13^ The Vaccine Production Program (Vaccine Research Center, National Institute of Allergy and Infectious Diseases [NIAID], National Institutes of Health [NIH], Bethesda, MD, USA) developed the manufacturing processes for L9LS and then transferred them to the Vaccine Clinical Materials Program for Good Manufacturing Practice-compliant production. L9LS was supplied at a concentration of 150 mg/mL, with 2·2 mL per vial.

Clinicians assessed participant eligibility through medical history, physical examination, anthropometry, and laboratory assessments for complete blood counts, liver and kidney function, haemoglobin typing, and HIV testing. All eligible participants received dihydroartemisinin–piperaquine to clear any baseline parasitaemia. Participants returned 2–3 weeks after dihydroartemisinin–piperaquine treatment for randomisation and dosing of L9LS or placebo.

In parts 1a and 1b, dosing was based on bodyweight, with up to 1·5 mL per injection for doses of 5 mg and 10 mg per kg of bodyweight and up to 2 mL for the higher doses. For volumes exceeding these limits, L9LS was divided equally between two injections, up to a maximum of 2 mL in each of two syringes. In part 2, bodyweight-tiered dosing was used: 75 mg of L9LS (0·5 mL) for participants weighing 5·0 kg to 7·5 kg, 150 mg (1 mL) for participants weighing more than 7·5 kg to 15·0 kg, and 225 mg (1·5 mL) for those weighing more than 15·0 kg to 22·5 kg, the upper weight limit in part 2, resulting in an overall dose range of 10–20 mg/kg. This bodyweight-tiered dosing strategy was used to model an easy-to-deploy approach while enabling pharmacokinetic and pharmacodynamic analysis across a dosing range to better define the protective dose. All subcutaneous injections were administered in the posterior upper arm, although the protocol also allowed for subcutaneous injections in the abdomen and inner thigh area.

After dosing (day 0), study field workers visited participants at home on day 1 and day 3 to inspect injection sites and collect solicited adverse event data (see appendix pp 6–8 for more details on assessment of solicited adverse events). Participants returned to the clinic on day 7 for a physical examination, adverse event assessment, and safety laboratory tests (complete blood count, liver, and kidney function). Participants in parts 1a and 1b were followed up on days 14, 21, 28, 56, and 84.

For part 2, active surveillance included monthly clinic visits from day 28 until day 364. Home visits occurred 2 weeks after each scheduled monthly clinic visit to assess health status and facilitate clinic referral when indicated. At 6 months, participants received their second dose of L9LS or placebo, with home visits 1 day and 3 days later and a clinic visit on day 7, and monthly visits thereafter.

Parents or guardians were encouraged to seek care for any illness between scheduled visits, with transport reimbursement provided. Study visits in parts 1a and 1b occurred at Siaya County Referral Hospital and those in part 2 either at Siaya County Referral Hospital or Kogelo Dispensary.

Blood was collected, primarily by fingerprick, at all scheduled clinic visits and, when indicated, at unscheduled sick visits for blood smears and dried blood spots. Blood smears were read by two independent microscopists (appendix p 9) within 48 h (or within 1 h for symptomatic participants), with a third microscopist adjudicating discordant results. Participants with any parasitaemia, regardless of symptoms, were treated with an antimalarial drug (typically artemether–lumefantrine) within 72 h.

Venous blood samples (up to 4·5 mL) for pharmacokinetic and antidrug antibody analyses were collected at baseline (2–3 weeks before L9LS or placebo dosing), on day 7, and either day 28 or day 84 (parts 1a and 1b), or days 28, 196, and 336 (part 2). Samples were centrifuged, aliquoted, and stored at −80°C before shipment to the Vaccine Research Center (NIAID, NIH). Dried blood spots were similarly stored and shipped to the University of Washington (Seattle, WA, USA) for highly sensitive *P falciparum* 18S rRNA quantitative RT-PCR (qRT-PCR) analysis.^17^ Serum L9LS concentrations were measured as described elsewhere.^8^ Antidrug antibody detection methods are detailed in the appendix (pp 9–10).

### Outcomes

Primary safety outcomes were incidence and severity of local and systemic solicited adverse events within 7 days of dosing and serious adverse events throughout followup. Unsolicited adverse events were collected throughout the study in parts 1a and 1b and up to 28 days after each dose in part 2; thereafter, they were recorded only if grade 3 or worse, or if deemed related to study product.

The primary efficacy endpoint for part 2 was asexual *P falciparum* infection detected by blood smear over 52 weeks of follow-up, comparing the two-dose L9LS group with the placebo group. Secondary efficacy endpoints included clinical malaria, defined as either: (1) parasitaemia of more than 5000 parasites per μL with axillary temperature at least 37·5°C; or (2) any parasitaemia with either temperature at least 37·5°C or history of fever within the past 24 h. The second definition aligns with Kenyan malaria treatment guidelines and is emphasised here.

Additional secondary endpoints included efficacy against parasitaemia detected by qRT-PCR;^17^ efficacy of a single dose of L9LS versus placebo at 3, 6, and 12 months and by age stratum; and L9LS pharmacokinetics, overall and in relation to *P falciparum* infection risk. Exploratory analyses included assessment of antidrug antibodies to L9LS and examination of whether pre-existing parasitaemia modified efficacy. Additional exploratory endpoints not presented here included assessment of IgG1 allotypes and allotype-specific effects on L9LS pharmacokinetics, whether L9LS efficacy is specific to certain parasite genotypes, effect of pre-existing circumsporozoite protein antibodies on efficacy and pharmacokinetics of L9LS, effect of L9LS on measles antibodies in participants in the 5–17-month age group (analyses ongoing), effect of L9LS on hospital admissions with malaria, and efficacy of one versus two doses of L9LS over 12 months of follow-up (insufficient statistical power).

### Statistical analysis

For part 2, the sample size was calculated to detect a target 60% protective efficacy of two doses of L9LS against infection at 12 months in the younger age group (5–17 months) with 80% power, assuming a 45% infection rate in the placebo group by 12 months, and 25% attrition, yielding 324 participants (162 per age stratum).

The primary analyses for safety and efficacy were done in the modified intention-to-treat population, consisting of all randomly assigned participants who received the study intervention. A sensitivity per-protocol analysis included participants who received both doses as randomly assigned, attended the close-out visit, and missed fewer than two consecutive clinic visits.

The primary efficacy analysis examined the incidence of *P falciparum* infection from 7 days post-initial dosing (estimated peak L9LS concentration) up to 52 weeks. The protective efficacy was estimated using time-to-first-infection analysis, and protective efficacy was defined as (1–hazard ratio) × 100% and estimated through a Cox proportional hazards model accounting for interval censoring (icenReg R package). Secondary analyses included proportion infected using Kaplan–Meier estimates (1–relative risk of infection) with melding method CIs^17^ and recurrent event analysis using the Anderson–Gill method,^18^ as well as analyses that excluded participant time when protected by antimalarial treatment. No multiplicity adjustments were applied to the analyses. Post-hoc analyses included comparison of proportions of serious adverse events in L9LS and placebo groups, assessment of the effect of seasonality on L9LS efficacy by including a covariate for month of enrolment in the Cox regression model, and comparisons of L9LS concentrations to protective efficacy. Participant data were entered directly into an electronic database (appendix p 10) during study visits. R statistical software (version 4.5.0) was used for all analyses.

### Role of the funding source

The funder of the study had no role in study design, data collection, data analysis, data interpretation, or writing of the report.

## Results

From September to October, 2022, 156 children had consent given for part 1a, of whom 72 (46%) were enrolled and randomly assigned from Oct 1 to Nov 7, 2022; 79 children had consent given for part 1b in December, 2023, of whom 24 (30%) were enrolled and randomly assigned from Jan 3 to 16, 2024 (appendix p 16). In total, 72 participants were assigned to L9LS and 24 were assigned to placebo in part 1.

**Figure 1:**
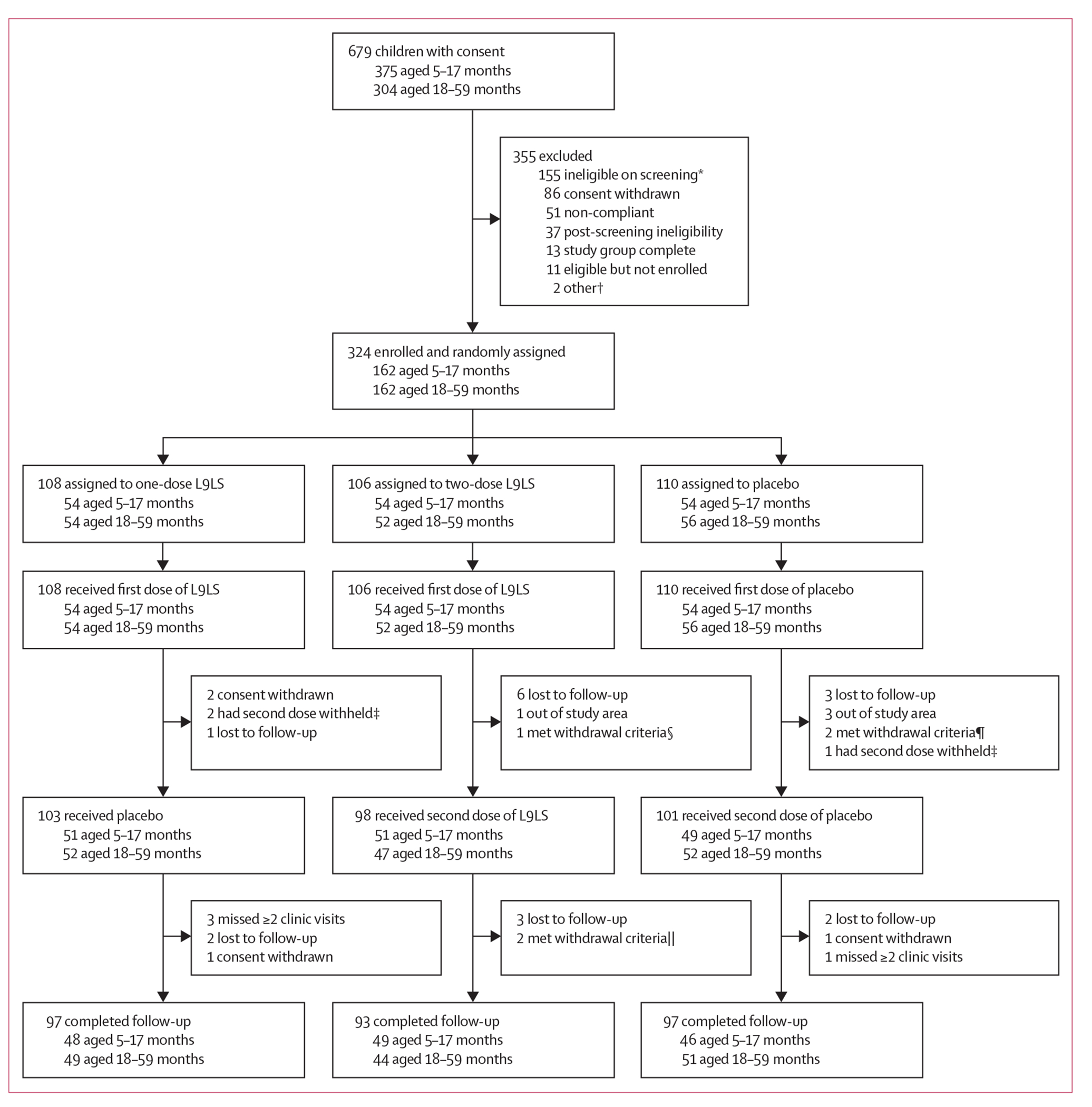
Trial profile for efficacy cohort *Reasons for ineligibility included weight-for-age or height-for-age Z score less than −2 (n=68); underlying illness (n=34); abnormal laboratory test (n=23); receipt of vaccine within prohibited window (n=14); participating in another study (n=13); or planning to move out of study area (n=3). †Other reasons were difficult veins for venepuncture and participant due for measles vaccine. ‡Two participants (one in the placebo group and one in the one-dose L9LS group) developed epilepsy and one (in the one-dose L9LS group) developed patent ductus arteriosus; second dose withheld but these three participants continued to be monitored up to 12 months. §Child received L9LS within 28 days of (live) rotavirus vaccine. ¶Both children received RTS,S vaccine. ||Both chidren received RTS,S vaccine.

For part 2, 679 children had consent given from January to May, 2023, of whom 324 (48%) were enrolled and randomly assigned between Jan 26 and June 2, 2023 (figure 1), with 162 children in each age stratum (5–17 months and 18–59 months). 108 children were assigned to one dose of L9LS (L9LS at baseline and placebo at 6 months), 106 were assigned to two doses of L9LS (L9LS at baseline and 6 months), and 110 were assigned to placebo at baseline and 6 months.

In part 2, 152 (47%) of 324 participants were male, 172 (53%) were female, 199 (61%) were enrolled at Siaya Hospital, and the remaining 125 (39%) were enrolled at Kogelo Dispensary (table 1). At baseline, 2–3 weeks before L9LS or placebo dosing when eligible participants received dihydroartemisinin–piperaquine to clear parasitaemia, 53 (16%) of 324 had a *P falciparum*-positive blood smear, and 145 (46%) of 315 had *P falciparum* detected by qRT-PCR (table 1). All participants had negative blood smears on the day L9LS or placebo was administered. Baseline characteristics for participants in parts 1a and 1b are shown in the appendix (pp 14–15).

In parts 1a and 1b, solicited local reactions were infrequent: in part 1a, injection site swelling occurred in three (11%) of 27 participants aged 5–10 years and in five (19%) of 27 participants aged 5–59 months in the L9LS group (all dose groups), compared with one (11%) of nine participants aged 5–10 years and none of nine participants aged 5–59 months in the placebo group; in part 1b, injection site swelling occurred in three (17%) of 18 participants in the L9LS group compared with none of six in the placebo group (appendix pp 17–19). Mild injection site pain occurred in two (7%) of 27 participants aged 5–10 years and three (11%) of 27 children aged 5–59 months assigned to L9LS in part 1a compared with none assigned to placebo (appendix pp 17–18). The only grade 3 (severe) local reaction was injection site swelling in one child assigned to placebo in part 1a (5–10-year age group). Solicited systemic events were predominantly mild, and included chills, headache, and pyrexia, with one case each of severe pyrexia and moderate nausea in the 40 mg/kg L9LS group in part 1b. No dose-related trends emerged for solicited or unsolicited adverse events (appendix pp 17–28). One child in the placebo group (in the 30 mg/kg cohort of part 1b) died 23 days post-dosing from severe dehydration following thermal burns (appendix pp 29–30).

In part 2, solicited adverse events were also uncommon: local reactions occurred in four (2%) of 214 participants after the first L9LS dose and in three (3%) of 98 participants after the second dose; among those receiving placebo, local reactions occurred in none of 110 participants after the first dose, and in one (<1%) of 204 participants receiving placebo for the second dose (table 2). Systemic events occurred in 20 (9%) of 214 participants after the first L9LS dose and in 14 (14%) of 98 participants after the second L9LS dose; among those receiving placebo, systemic events occurred in six (5%) of 110 participants after the first dose, and in 22 (11%) of 204 participants receiving placebo for the second dose (table 2). These events consisted of pain, swelling, or reaction at the injection site, and nausea, malaise, pyrexia, and headache, all of which were mild or moderate apart from three grade 3 events: one injection site induration starting 1 day after the second L9LS dose (resolved by day 3), and two episodes of pyrexia after the second L9LS dose (one at 39·6°C on day 3, resolving within 48 h; another with concurrent varicella [chickenpox] that began the day of dosing [maximum fever of 39·8°C 7 days after dosing] and resolved 11 days later). In part 2, unsolicited adverse events occurred in 252 (78%) of 324 participants within 28 days after the first dose of L9LS or placebo and in 246 (81%) of 302 participants after the second dose, with similar rates across study groups (table 2, appendix pp 31–35). Across all study parts (parts 1a, 1b, and part 2), grade 3 or worse treatment-related adverse events occurred after four (1%) of 384 L9LS injections and two (1%) of 338 placebo injections; these events all resolved by study end. No deaths occurred in part 2, while 28 serious adverse events occurred, all deemed unrelated to L9LS in masked analysis (appendix pp 29–30). A post-hoc analysis of serious adverse events (most commonly severe malaria [three events in participants assigned to L9LS *vs* six in participants assigned to placebo] and pneumonia [two *vs* one]) showed that the proportion of serious adverse events was significantly higher in the placebo group than in the L9LS dose groups combined (χ^2^ 7·4, p=0·0067).

**Figure 2:**
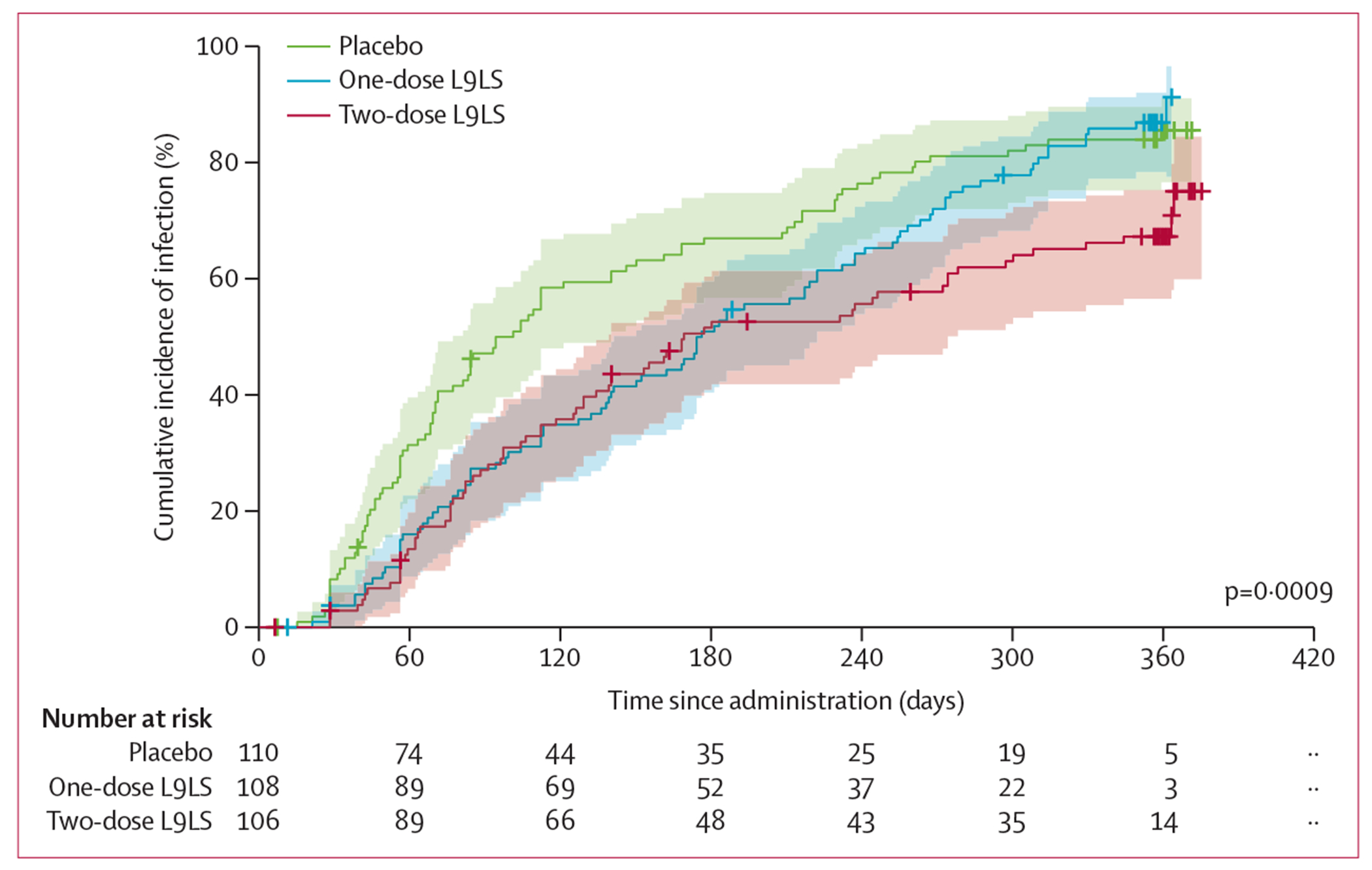
Kaplan–Meier curve of time to first *Plasmodium falciparum* infection as detected by blood smear, by treatment group Data for all participants (ie, 5–17-month and 18–59-month age groups combined). Surveillance for infection by blood smear began 7 days after the first dose of L9LS or placebo and continued until the close-out visit. Shaded areas represent 95% CIs. Log-rank test p=0·0009 for all participants comparing the two-dose L9LS group with the placebo group. Median time to first infection for all participants was 176 days (95% CI 141–222) in the one-dose L9LS group, 169 days (139–274) in the two-dose L9LS group, and 101 days (77–140) in the placebo group.

**Figure 3:**
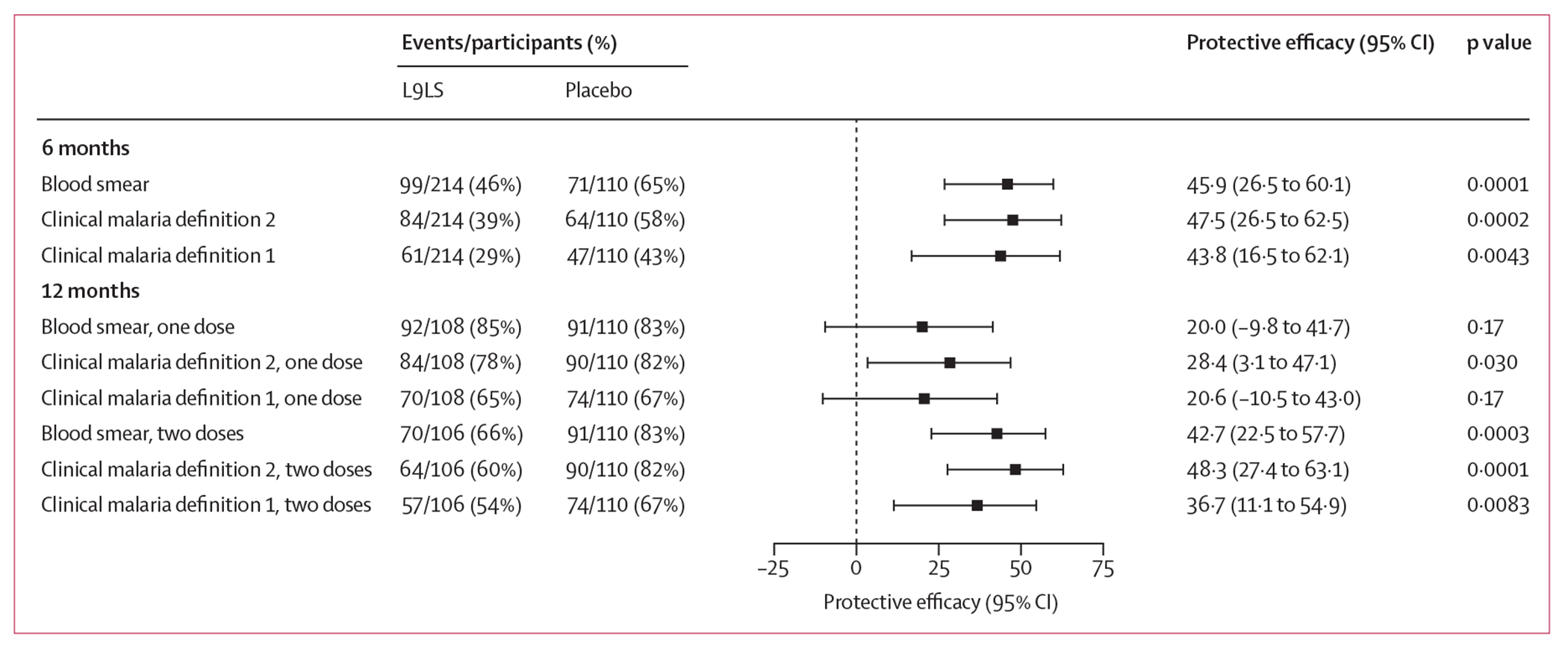
Protective efficacy of one or two doses of L9LS against *Plasmodium falciparum* infection and clinical malaria at 6 months and 12 months compared with placebo, by time-to-event analysis Protective efficacy calculated using Cox proportional hazards model ([1–hazard ratio] × 100%) accounting for interval censoring. Lines around protective efficacy point estimates represent 95% CIs. Clinical malaria defined as either: (1) parasitaemia of more than 5000 parasites per μL with axillary temperature at least 37·5°C; or (2) any parasitaemia with either temperature at least 37·5°C or history of fever within the past 24 h.

Of the 324 children enrolled in part 2, 253 (78%) had at least one *P falciparum* infection during 52 weeks of follow-up: 92 (85%) of 108 in the one-dose L9LS group, 70 (66%) of 106 in the two-dose L9LS group, and 91 (83%) of 110 in the placebo group (figure 2 , appendix p 36). Estimated by time to first infection and accounting for interval censoring, the protective efficacy of two doses of L9LS against *P falciparum* infection by blood smear at 12 months, the primary endpoint, was 42·7% (95% CI 22·5–57·7; p=0·0003; figures 2, 3), and was similar for the 5–17-month and 18–59-month age groups (appendix pp 37–38). At 6 months, after one dose of L9LS (both L9LS groups) or placebo, infections occurred in 99 (46%) of 214 children in the L9LS group and 71 (65%) of 110 in the placebo group (protective efficacy 45·9% [95% CI 26·5–60·1]; p=0·0001; figure 3, appendix p 36), and efficacy was higher in the 18–59-month age group (50·9% [25·3–67·7]) than in the 5–17-month age group (39·0% [1·9–62·0]), although this difference was not statistically significant (appendix p 38). One dose of L9LS provided no statistically significant protection against *P falciparum* infection at 12 months (protective efficacy 20·0% [95% CI –9·8 to 41·7]; figure 3, appendix p 36).

**Figure 4:**
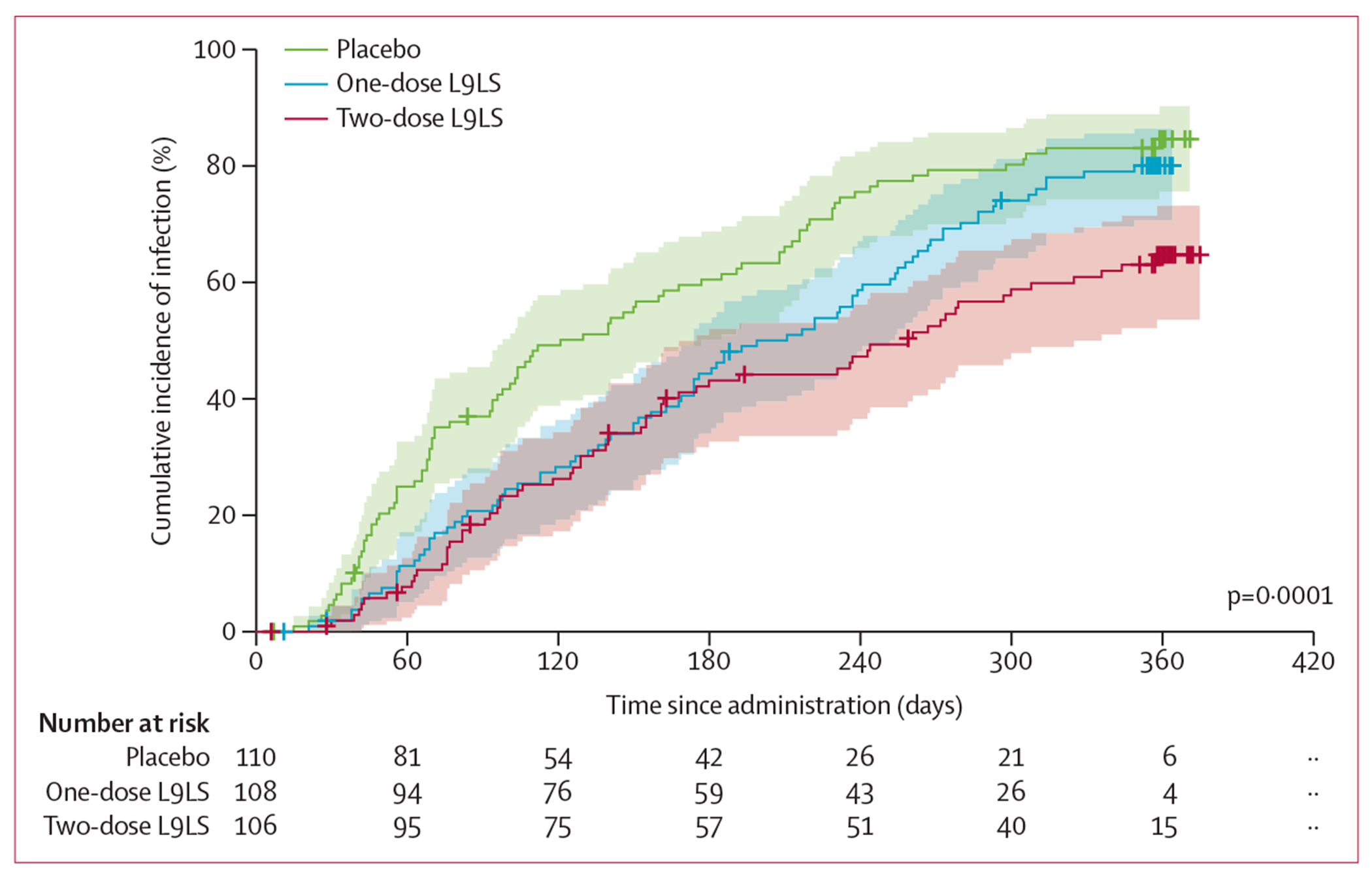
Kaplan–Meier curve of time to first clinical malaria, by treatment group Data for all participants (ie, 5–17-month and 18–59-month age groups combined). Clinical malaria defined as blood smear-detected *Plasmodium falciparum* infection plus high measured temperature (≥37·5°C) or history of fever in past 24 h. Surveillance for clinical malaria began 7 days after the first dose of L9LS or placebo and continued until the close-out visit. Shaded areas represent 95% CIs. Log-rank test p=0·0001 comparing the two-dose L9LS group with the placebo group. Median time to first clinical malaria for all participants was 199 days (95% CI 174–252) in the one-dose L9LS group, 259 days (175–336) in the two-dose L9LS group, and 121 days (98–177) in the placebo group.

Protective efficacy of two doses of L9LS against clinical malaria definition 2 (ie, any parasitaemia with either temperature at least 37·5°C or history of fever within the past 24 h) at 12 months was 48·3% (95% CI 27·4–63·1; figures 3, 4), without substantial differences by age group (appendix pp 38–39). One dose of L9LS provided modest protection against clinical malaria definition 2 at 12 months (28·4% [95% CI 3·1 to 47·1]), but not against clinical malaria definition 1 (ie, parasitaemia >5000 parasites per µL with axillary temperature at least 37·5°C; protective efficacy 20·6% [–10·5 to 43·0]; figure 3). At 3 months, one dose of L9LS had a protective efficacy of 51·6% (95% CI 28·9–67·1) against *P falciparum* infection and 55·2% (30·2–71·2) against clinical malaria (definition 2), with no meaningful differences by age group (appendix p 40).

As expected, protective efficacy estimates from Kaplan– Meier proportional analyses were consistently lower than those from time-to-first event analyses (appendix p 41). Protective efficacy estimates for recurrent events were higher than those from proportional analyses, but slightly lower than estimates from time-to-first-event analyses. For example, at 12 months, efficacy of two doses of L9LS was 31·4% (95% CI 7·1–49·4) against all *P falciparum* infections and 41·0% (20·2–56·4) against all clinical malaria episodes (definition 2; appendix p 42). Participants in the younger cohort (age 5–17 months) in the one-dose L9LS group had modestly higher *P falciparum* infection rates during months 7–12 (5·6 infections per person-year [95% CI 4·6–6·6]) compared with those in the placebo group (3·7 infections per person-year [2·9–4·5]), although the incidence of clinical malaria was similar during this time (appendix pp 39, 43). Efficacy of one dose of L9LS was 41·8% (95% CI 21·4–56·9) against all *P falciparum* infections and 42·3% (20·1–58·4) against all episodes of clinical malaria over 6 months by recurrent event analysis (appendix p 42).

Analyses that excluded periods of time when participants were protected after antimalarial treatment, as well as per-protocol analyses, yielded similar results to the primary modified intention-to-treat analyses (appendix pp 44–46). Participants positive for *P falciparum* by blood smear or qRT-PCR at baseline before dihydroartemisinin–piperaquine administration were more likely to have infection during follow-up, a finding that was consistent across all study groups and both age groups (appendix pp 47–50). One dose of L9LS showed slightly higher protective efficacy at 6 months in participants who were qRT-PCR-negative versus qRT-PCR-positive at baseline, while two doses of L9LS showed similar efficacy at 12 months in participants who were qRT-PCR-negative or qRT-PCR-positive at baseline (appendix p 51). A post-hoc analysis that included the participants’ month of enrolment as a regressor (surrogate for potential transmission seasonality) did not change the efficacy estimates significantly (appendix p 52). Efficacy against infection detected by qRT-PCR is not presented here because qRT-PCR analyses were not complete at the time of manuscript submission.

L9LS showed dose-proportional pharmacokinetics in this trial, with maximum serum concentrations of 52·5 μg/mL at 5 mg/kg, 104·8 μg/mL at 10 mg/kg, 197·0 μg/mL at 20 m/kg, 417·1 μg/mL at 30 mg/kg, and 542.0 μg/mL at 40 mg/kg (appendix pp 53–54). Notably, despite the 40 mg/kg dose group having a higher observed maximum concentration (C_max_) at day 7, L9LS serum concentrations by 28 days were similar between the 30 mg/kg and 40 mg/kg dose groups (appendix pp 53), as L9LS serum concentrations decreased at significantly faster rates from observed C_max_ to day 28 or day 84 for 40 mg/kg than in the lower dose groups, with the exception of the 30 mg/kg dose group, which had the smallest sample size (appendix pp 55–56). In part 2, in participants who received a single dose of L9LS, median terminal half-life estimated by non-compartmental analysis using sparse data was 39·9 days (95% CI 39·3–40·1) in participants aged 5–17 months and 41·6 days (40·9–42·5) in those aged 18–59 months (appendix pp 58–59).

No antidrug antibodies were detected among children in the L9LS groups at any timepoints in parts 1a and 1b or in part 2 (measured at baseline and days 28, 196, and 336 in part 2), although one participant in the placebo group had functional antidrug antibodies detected at three timepoints after the first dose (appendix p 60), suggesting a likely non-specific interference leading to a false-positive result.

In part 2, bodyweights ranged from 6·5 kg to 12·2 kg in children aged 5–17 months and from 9·3 kg to 20·4 kg in children aged 18–59 months, resulting in weight-based L9LS dose ranges during the first dose of 10·0–19·7 mg/kg (mean 15·5 mg/kg) for participants aged 5–17-months and 10·0–16·1 mg/kg (mean 12·3 mg/kg) for those aged 18–59 months (appendix pp 61–62). Post-hoc analyses showed positive relations between weight-based dosing and protective efficacy across all trial endpoints (appendix p 63). Formal analyses correlating pharmacokinetics with efficacy are in progress.

## Discussion

This phase 2 trial provides evidence that subcutaneous administration of L9LS is safe, well tolerated, and efficacious against *P falciparum* infection and clinical malaria in infants and young children in an area of intense perennial *P falciparum* transmission in western Kenya. L9LS doses up to 40 mg/kg and subcutaneous injections up to 2 mL were well tolerated in children as young as 5 months. Two doses of L9LS at 10–20 mg/kg, administered 6 months apart, showed comparable safety profiles and did not elicit antidrug antibodies. One dose of L9LS at 10–20 mg/kg in children aged 5–59 months provided 46% efficacy against *P falciparum* infection detected by blood smear and 48% efficacy against clinical malaria over 6 months, while two doses administered 6 months apart provided 43% efficacy against infection and 48% efficacy against clinical malaria over 12 months. While multitrial pharmacokinetic and pharmacodynamic modelling of L9LS is underway, this trial suggests that higher doses of L9LS might be needed to achieve more than 70% efficacy in young children, particularly in areas of intense perennial transmission. In part 1b of this study, 30 mg/kg yielded a higher serum concentration of L9LS than the lower doses assessed in part 1a. Ongoing trials in Mali and Kenya are investigating L9LS doses of 30 mg/kg and higher in infants and children (NCT06461026 and NCT07082205) and adults (NCT07060508).

The favourable safety and tolerability profile of L9LS, with only four (2%) of 214 participants having local reactions and 20 (9%) of 214 having solicited systemic events after their first L9LS dose, aligns with findings from previous L9LS trials across different age groups.^8,12^ By comparison, phase 3 trials of WHO-recommended malaria vaccines found that up to 47% of participants had fever with R21/Matrix-M,^19^ and up to 33% had fever with RTS,S/AS01.^20^ This difference in adverse events between monoclonal antibodies and vaccines probably reflects immune-stimulating adjuvants required for vaccine-induced immune responses. Indeed, widespread use of other monoclonal antibodies, including nirsevimab for respiratory syncytial virus prevention, show similarly low adverse event rates in infants.^21^

The efficacy of two doses of L9LS over 12 months, and one dose over 6 months in this study, was lower than the 70% efficacy against *P falciparum* infection and 77% efficacy against clinical malaria over 6 months observed in Malian children aged 6–10 years when 300 mg of L9LS (approximate weight-based dose range 10–20 mg/kg) was administered just before the 6-month malaria season.^8^ Seasonal transmission patterns might result in higher efficacy estimates for interventions like monoclonal antibodies that have waning efficacy over time when administered at the start of the malaria season, compared with efficacy estimates in perennial settings.^22^ However, during the first 3 months of this trial, when L9LS concentrations remained well above the limit of detection, efficacy was only 52% against *P falciparum* infection and 55% against clinical malaria. Furthermore, 81% of participants in the placebo group in Mali^8^ versus 63% in Kenya had at least one *P falciparum* infection over 6 months, suggesting comparable transmission intensities at both sites. Ongoing genotype analyses of *P falciparum* infections detected in this trial and the Mali trials will provide additional transmission intensity measures^23^ at the two sites and might also determine whether polymorphisms in the *P falciparum* circumsporozoite protein locus are associated with differential L9LS efficacy. The lower efficacy of L9LS in this trial might also reflect different L9LS pharmacokinetics in Kenyan children 5–59 months old compared with Malian children 6–10 years old. L9LS serum concentrations in the 5–59-month and 5–10-year age groups from part 1a showed more rapid decreases compared with those reported in US adults who received 5 mg/kg L9LS in the phase 1 trial (appendix p 57).^12^ Previous studies of other monoclonal antibodies have shown shorter half-lives in infants and young children than in adults.^24–26^ Population-specific factors, including Fc receptor polymorphisms^27^ and expression levels as well as differences in baseline immunological states,^28^ could influence L9LS pharmacokinetics and efficacy; these factors are being investigated in continuing studies.

This study has limitations. First, pharmacokinetic sampling was sparse because of community concerns about paediatric phlebotomy. Second, the narrow range of L9LS doses in part 2 (due to bodyweight-tiered dosing) limited exploration of dose–efficacy relations. Ongoing pharmacokinetic and pharmacodynamic modelling with data from multiple L9LS trials in various transmission settings will further refine dosing regimens across age groups. Third, the study was done within a single area where *P falciparum* transmission is intense and perennial. It will be of interest in future studies to investigate L9LS in areas of low and moderate perennial transmission. Fourth, all participants received dihydroartemisinin–piperaquine before L9LS or placebo administration to clear possible *P falciparum* infection so that the primary efficacy endpoint of infection could be assessed. Consequently, this trial could not assess whether pretreatment with dihydroartemisinin–piperaquine affected efficacy estimates of L9LS. Ongoing (NCT06461026) and future trials of L9LS that have clinical malaria as an endpoint and do not include pretreatment with an antimalarial drug could provide insight into this question. Finally, this trial included healthy children only. A continuing trial is addressing this limitation by evaluating the safety and efficacy of L9LS in children with severe anaemia or severe malaria to prevent malaria after hospital discharge (NCT07082205).

The results of this study support conducting further trials to assess the safety and efficacy of L9LS in infants and children in various transmission settings and for different use cases. For example, in perennial transmission settings, L9LS could be assessed when administered at routine immunisation visits at 6 weeks or 10 weeks of age, potentially complementing existing countermeasures (eg, long-lasting insecticidal nets) to enhance protection of infants before completion of the three-dose malaria vaccine primary series several months later. Importantly, a trial in Mali is investigating whether L9LS affects subsequent R21/Matrix-M immunogenicity in infants (NCT06461026). To protect children exposed to seasonal malaria transmission, a single annual dose of L9LS could be compared with monthly seasonal malaria chemoprevention—the current standard of care that is limited by the challenge of delivering frequent treatment courses.^29^ Finally, a trial in Kenya is assessing the safety and efficacy of a single dose of L9LS in children with severe anaemia or severe malaria to prevent malaria after hospital discharge over 6 months compared with the WHO-recommended three courses of monthly post-discharge malaria chemoprevention (NCT07082205). Larger trials in the future can also explore the effect of L9LS on severe and fatal malaria, as well as the potential for increased malaria risk after a period of L9LS-mediated protection.^30^

In conclusion, this phase 2 trial represents the first L9LS evaluation in children younger than 5 years and in the setting of intense perennial *P falciparum* transmission. The results suggest that L9LS was well tolerated and moderately protective against malaria in infants and young children at the doses used, without evident safety concerns. A higher dose of L9LS might be necessary to achieve more than 70% efficacy over 6 months in infants and young children when exposed to intense perennial transmission. In the longer term, higher efficacy could also be achieved through ongoing research to enhance the potency and durability of L9LS.^4^

## Supplementary Material

Supplementary materials

## Figures and Tables

**Table 1:** Characteristics of participants enrolled in efficacy cohort

	18–59 months of age*			5–17 months of age*		
	One-dose L9LS (n=54)	Two-dose L9LS (n=52)	Placebo (n=56)	One-dose L9LS (n=54)	Two-dose L9LS (n=54)	Placebo (n=54)
Sex						
Female	29 (54%)	27 (52%)	27 (48%)	28 (52%)	34 (63%)	27 (50%)
Male	25 (46%)	25 (48%)	29 (52%)	26 (48%)	20 (37%)	27 (50%)

Age, months	37 (25–45)	34 (27–49)	31 (24–41)	13 (9–15)	10 (6–14)	13 (7–15)

Study site						
Kogelo	22 (41%)	21 (40%)	24 (43%)	19 (35%)	19 (35%)	20 (37%)
Siaya	32 (59%)	31 (60%)	32 (57%)	35 (65%)	35 (65%)	34 (63%)

Weight at first dose, kg	13·6 (2·4)	14·1 (2·4)	13·2 (2·2)	9·3 (1·3)	8·9 (1·3)	9·2 (1·4)

L9LS dose, mg/kg	12·4 (1·5)	12·3 (1·5)	12·6 (1·5)	15·4 (2·4)	15·6 (2·8)	14·9 (2·5)

Positive blood smear at baseline†	11 (20%)	14 (27%)	12 (21%)	5 (10%)	9 (17%)	2 (4%)

Positive qRT-PCR at baseline†‡	26 (49%)	31 (61%)	32 (58%)	23 (45%)	19 (37%)	14 (26%)

Data are n (%), median (IQR), or mean (SD). qRT-PCR=quantitative RT-PCR.

* Age at time of dosing.

† Baseline refers to the pre-enrolment visit for giving dihydroartemisinin–piperaquine for parasite clearance, 2–3 weeks before antibody administration. Positivity is only for *Plasmodium falciparum*.

‡ qRT-PCR unable to be run on nine baseline samples: four in the one-dose L9LS group, three in the two-dose L9LS group, and two in the placebo group. Other variables have no missing data.

**Table 2:** Summary of adverse events in efficacy cohort

	One-dose L9LS		Two-dose L9LS		Placebo	
	After dose 1 (n=108)	After dose 2 (n=103)	After dose 1 (n=106)	After dose 2 (n=98)	After dose 1 (n=110)	After dose 2 (n=101)
Solicited adverse events						
Participants with at least one solicited local adverse event within 7 days of dosing	1 (1%)	0	3 (3%)	3 (3%)	0	1 (1%)
Participants with at least one solicited systemic adverse event within 7 days of dosing	12 (11%)	10 (10%)	8 (8%)	14 (14%)	6 (5%)	12 (12%)
Local reactogenicity adverse events						
Injection site induration*						
Severe	0	0	0	1 (1%)	0	0
Injection site pain						
Mild	0	0	1 (1%)	0	0	0
Injection site reaction†						
Mild	0	0	1 (1%)	0	0	0
Injection site swelling						
Mild	1 (1%)	0	1 (1%)	2 (2%)	0	1 (1%)
Systemic solicited adverse events						
Nausea						
Moderate	2 (2%)	2 (2%)	3 (3%)	3 (3%)	2 (2%)	2 (2%)
Mild	3 (3%)	1 (1%)	2 (2%)	4 (4%)	1 (1%)	4 (4%)
Malaise						
Mild	0	1 (1%)	0	0	0	0
Pyrexia						
Severe‡	0	0	0	2 (2%)	0	0
Moderate	1 (1%)	2 (2%)	1 (1%)	2 (2%)	0	3 (3%)
Mild	7 (6%)	5 (5%)	3 (3%)	5 (5%)	3 (3%)	5 (5%)
Headache						
Mild	1 (1%)	0	1 (1%)	0	0	1 (1%)
Unsolicited adverse events						
Participants with at least one unsolicited adverse event within 28 days of dosing	83 (77%)	84 (82%)	83 (78%)	81 (83%)	86 (78%)	81 (80%)
Participants with at least one related unsolicited adverse event within 28 days of dosing	6 (6%)	9 (9%)	1 (1%)	6 (6%)	5 (5%)	7 (7%)
All adverse events						
Participants with a grade 3 related adverse event (solicited or unsolicited)	0	0	0	3 (3%)	0	1 (1%)
Participants with a serious adverse event§	3 (3%)	4 (4%)	3 (3%)	1 (1%)	6 (5%)	10 (10%)

No participants had local reactions of injection site tenderness, redness, pruritus, or bruising. No participants had systemic solicited events of muscle aches, chills, or joint pain. All local events were deemed related to the study. All systemic events were deemed related to the study, apart from one case of nausea in a participant in the placebo group after dose 2, who had concomitant malaria and bacterial infection.

* Injection site induration began 1 day after dosing (maximum swelling of 30 mm) and resolved by day 3.

† Injection site reaction was a muscle tension under the injection site that appeared within 1 h of dosing and resolved by day 3.

‡ Severe pyrexia defined as axillary temperature from 39·5°C to 41·9°C.

§ No deaths occurred in part 2, and none of the serious adverse events were deemed related to the study.

## Data Availability

Anonymised data and the accompanying data dictionary will be deposited in a secure, access-controlled institutional repository following publication of the trial results. Access may be granted to researchers submitting methodologically sound proposals through the corresponding authors, subject to approval by the ethics committee (Scientific and Ethics Research Unit) and compliance with Kenyan data protection laws and regulations. Data access will require a formal data use agreement.

## Research in context

### Evidence before this study

We searched PubMed and the Cochrane Library without language restrictions from Jan 1, 2017, when the first antimalaria monoclonal antibodies were in initial clinical development, until Dec 1, 2025, using the search terms “monoclonal antibodies” or “monoclonal antibody” and “malaria”. Three long-acting antimalaria monoclonal antibodies for mediating protection that are specific for the *Plasmodium falciparum* circumsporozoite protein—namely, CIS43LS, L9LS, and MAM01—are currently in clinical development. These monoclonal antibodies were isolated from blood samples of study participants immunised with either a radiation-attenuated whole sporozoite vaccine (CIS43LS and L9LS) or the RTS,S/AS01 vaccine (MAM01) and engineered to extend their half-lives. CIS43LS and L9LS were shown to be safe and efficacious against controlled human malaria infection in the USA and natural infection in endemic settings. In Mali, where intense *P falciparum* transmission occurs during the 6-month rainy season, a phase 2 study in healthy adults showed that a single intravenous dose of CIS43LS was safe and 88% efficacious against infection over 6 months at a dose of 40 mg/kg and 75% efficacious at a dose of 10 mg/kg, compared with placebo. Another phase 2 study at the same site in Mali in children aged 6–10 years showed that a single subcutaneous dose of L9LS was safe and 77% efficacious against clinical malaria over 6 months at a dose of 10–20 mg/kg and 67% efficacious at a dose of 5–10 mg/kg, compared with placebo. Efficacy of MAM01 against controlled human malaria infection was assessed in US adults (NCT05891236), and its safety and pharmacokinetics are being assessed in an ongoing phase 1b trial in adults and children in Uganda (NCT06408857). However, to date, no published studies have assessed the safety and efficacy of antimalaria monoclonal antibodies in children younger than 6 years, who are most vulnerable to severe malaria, or in a perennial transmission setting.

### Added value of this study

This two-part, placebo-controlled, randomised, phase 2 clinical trial tested the safety of a range of L9LS doses (5 mg/kg bodyweight, 10 mg/kg, 20 mg/kg, 30 mg/kg, and 40 mg/kg) administered subcutaneously to children aged 5–59 months and 5–10 years, and the safety and efficacy of one or two doses of L9LS, administered 6 months apart at a dose of 10–20 mg/kg, for prevention of malaria in children aged 5–59 months over 12 months of follow-up. The trial took place in western Kenya, a site of intense, perennial malaria transmission. L9LS was safe and well tolerated in children and infants at all doses tested. Two doses of L9LS, given 6 months apart, achieved 43% protective efficacy against infection (48% against clinical malaria), and one dose provided 46% protection over 6 months (48% against clinical malaria). Pharmacokinetics of L9LS showed the expected dose-linear relation. Additionally, at the 5 mg/kg dose, L9LS serum concentrations decreased to lower levels by 3 months in Kenyan children when compared with US adults, a finding previously observed with other monoclonal antibodies, such as nirsevimab for respiratory syncytial virus prevention.

### Implications of all the available evidence

Building on evidence from phase 1 and 2 studies in the USA and Mali, findings from this trial suggest that L9LS is safe and efficacious in infants and young children, paving the way for future implementation in this age group that is particularly vulnerable to malaria. Although protection against infection was significant (for both one dose at 6 months and two doses at 12 months), efficacy was approximately 35% lower than that seen in older children in Mali (aged 6–10 years) who received L9LS subcutaneously at a similar dose range. Age-dependent differences in L9LS pharmacokinetics, potential differences in malaria transmission between Mali and Kenya, and population-specific immunological profiles, along with other factors, might explain the lower efficacy in young children in western Kenya. Because the previous studies have shown dose-dependent protection by CIS43LS and L9LS, higher dosing might contribute to achieving optimal efficacy, especially in young children and infants in perennial transmission sites. On the basis of these findings, planned and continuing studies (NCT06461026) will evaluate a dose of about 30 mg/kg of L9LS across different clinical use cases, which include an ongoing study in vulnerable children discharged from hospital with severe malaria or severe anaemia in Kenya (NCT07082205). Additional studies are needed to understand potential differences in pharmacokinetics of L9LS in infants and young children and to elucidate other possible factors affecting protective efficacy of antimalarial monoclonal antibodies in sites of intense, perennial transmission.
