# Supplementary material for: Safety and efficacy of the monoclonal antibody L9LS for malaria prevention in children exposed to perennial malaria transmission in Kenya: a randomised, double-blind, placebo-controlled, phase 2 trial
Source: Lancet. Author manuscript; Available in PMC 2026 Jun 15. (PMC13266303; doi:10.1016/S0140-6736(26)00258-8)
Supplement: Supplementary materials [file NIHMS2175795-supplement-Supplementary_materials.pdf]

THE LANCET

Supplementary appendix

This appendix formed part of the original submission and has been peer reviewed. We post it as supplied by the authors.

Supplement to: Steinhardt LC, Kwambai TK, Oneko M, et al. Safety and efficacy of the monoclonal antibody L9LS for malaria prevention in children exposed to perennial malaria transmission in Kenya: a randomised, double-blind, placebo-controlled, phase 2 trial. *Lancet* 2026; **407:** 1614–25.

Supplementary materials

Contents

Kenya Malaria mAb Trial Group Members 1
Supplementary Methods 2
- Inclusion and exclusion criteria 2
- Study objectives and endpoints 4
- Grading and relatedness of solicited adverse events 6
- Microscopy reading of blood smears 9
- Anti-drug antibody (ADA) analyses 9
- Electronic data capture 10
- Additional details on statistical analyses 10

Supplementary Tables and Figures 12
Study Protocol 64
Statistical analysis plan 194

Kenya Malaria mAb Trial Group Members

Kenya Medical Research Institute (KEMRI)

Steven Achieng, Fredrick Adhola, Eliakim Agono, Elizabeth Ambululi, Lynn Amondi, Mary Awuor, Benard Ayatta, Dennis Bii, Lilian Chemada, Linedi Chemwok, Miriam Chomba, Gentrix Indoshi, Lwanga Karoli, Martha Kimungui, Franklin Komino, Nypha M'mayi, Judy Magoma, Sharon N'getich, George N'gonga, Nancy Nyamache, Joshua Obiya, Louizah Ochola, Telephorus Odawo, Patricia Odera Ojwang, Maureen Odhiambo, Ronald Odhiambo, Clevers Odhiambo, Leonard Odipo, Esther Odongo, Beatrice Odoyo, Kennedy Oduol, Charles Oduor, Kevin Ogamba, Willis Ogillo, Veronicah Okuna, Damaris Okwiri, Edwin Olweny, Dennis Omondi, Postine Omwalo, Mary Omwalo, Francis Orwa, Shaltone Osele, Benard Otero, Daniel Ouko, Felix Ouko, Diana Owera, Ann Owino, Faith Oyier, Linet Sewe.

Vaccine Research Center, NIAID, NIH

Martin Apgar, Mimi Campbell, Robin Carroll, Mike Castro, Jason Gall, Wesley Goss-Geffrard, Janel Holland-Linn, Yogita Jethmalani, Rachel Kazmierski, Richard A. Koup, Jeffery Liao, Kwang Low, Allen Mueller, Madeeha Mughal, Sandeep Narpala, Leigh Stephens.

Office of Cyber Infrastructure and Computational Biology, NIAID, NIH

Laxmi Buyyani, Kanwaldeep Bajwa, Michael Holdsworth, Harish Kandaswamay, Diane Rock Kress, Mariam Namawejje, Pengchong Tang

Office of Clinical Research Policy and Regulatory Operations, NIAID/Leidos Biomed

Neelam Gulati, Liam Harmon, Shirley Jankelevich, Yvonne Jato, Aaren King, Stacy Kopka,
Valerie Opher, John Tierney, Susan Vogel, Saran Wells, Kristin Young

Malaria Molecular Diagnostic Laboratory, Dept. of Laboratory Medicine and Pathology,

University of Washington

Sammi Cheung, Annette M. Seilie, Weston Staubus, Ashley Subijanto

Supplementary Methods

Inclusion and exclusion criteria

Individuals must meet all of the following criteria to be eligible for study participation:

1. Healthy children aged 5 months to 10 years (Part 1a), 5 to 71 months (Part 1b) or 5-59 months (Part 2)
2. Weight ≥5 kg and weight ≤30 kg (Part 1a) or weight ≥5 kg and ≤22.5 kg (Part 2) or weight ≥5 kg and ≤20 kg (for 30 mg/kg group) or weight ≥5 kg and ≤15 kg (for 40 mg/kg group), to ensure a maximum volume of two 2-mL (Part 1b)
3. Hemoglobin level ≥8 g/dL.
4. Height and weight Z-scores >-2
5. Living within Alego-Usonga sub-county.
6. Able to participate for the duration of the trial
7. Parent and/or guardian of participant able to provide informed consent.

Individuals meeting any of the following criteria will be excluded from study participation:

1. Taking long-term cotrimoxazole.
2. Participation or planned participation in any other interventional trial with an investigational product prior to the last required protocol visit or receipt of an investigational product within the past 30 days. (Note: Past, current, or planned participation in observational studies is NOT exclusionary.)
3. Received any doses of any malaria vaccine.
4. Participation in part 1 of this study (for individuals being screened for enrollment into part 2)
5. Age < 12 months at the time the RTS,S/AS01 vaccine is anticipated to become available in the whole of Siaya County
6. Current significant medical condition (neurologic, cardiac, pulmonary, hepatic, endocrine, rheumatologic, autoimmune, renal, oncologic, or hematological) or evidence of any serious underlying medical condition identified by medical history, physical examination, or laboratory examination.
    a. Known sickle cell disease. (Note: Known sickle cell trait is NOT exclusionary.)
    b. White blood cell, absolute neutrophil, or platelet count outside the local laboratory-defined limits of normal. Subjects may be included at the investigator’s discretion for values that are not clinically significant (i.e., do not require any repeat or follow-up).
    c. Alanine transaminase (ALT) or creatinine (Cr) level above the local laboratory-defined upper limit of normal. (Subjects may be included at the investigator’s discretion for values that are not clinically significant.)
    d. Infected with HIV.
    e. History of a severe allergic reaction or anaphylaxis.

f. Severe asthma (defined as asthma that is unstable or required emergency care, urgent care, hospitalization, or intubation during the past 2 years, or that has required the use of oral or parenteral corticosteroids at any time during the past 2 years).

g. Pre-existing autoimmune or antibody-mediated diseases including but not limited to: systemic lupus erythematosus, rheumatoid arthritis, multiple sclerosis, Sjogren's syndrome, or autoimmune thrombocytopenia.

h. Known immunodeficiency syndrome.

i. Use of chronic (≥14 days) oral or IV corticosteroids (excluding topical or nasal) at immunosuppressive doses (i.e., prednisone >10 mg/day) or immunosuppressive drugs within 30 days of day 0.

j. Known asplenia or functional asplenia.

k. Clinical signs of malnutrition.

l. Receipt of immunoglobulins and/or blood products within the past 6 months.

7. Any history of menses.
8. Behavioral, cognitive, or psychiatric disease that in the opinion of the investigator affects the ability of the subject to understand and comply with the study protocol.
9. Parental/guardian study comprehension examination score of <80% correct or per investigator discretion.
10. Receipt of a live vaccine or a killed vaccine within the past 2 weeks prior to study agent administration.
11. Known allergies or contraindication to dihydroartemisinin-piperaquine.
12. Use or known need at the time of pre-enrolment (DP administration) of concomitant prohibited medication, including:
    a. Antimicrobial agents of the following classes (systemic use only):
        i. Macrolides (e.g. erythromycin, clarithromycin, azithromycin, roxithromycin)
        ii. Fluoroquinolones (e.g., levofloxacin, moxifloxacin, sparfloxacin)
        iii. Pentamidine
    b. Antiarrhythmic agents (e.g. amiodarone, sotalol)
    c. Antihistamines (e.g. promethazine)
    d. Antifungals (systemic): ketoconazole, fluconazole, itraconazole
    e. Antiretrovirals: Saquinavir
    f. Diuretics (e.g. hydrochlorothiazide, furosemide)
    g. Antipsychotics (neuroleptics): haloperidol, thioridazine
    h. Antidepressants: imipramin, citalopram, escitalopram
    i. Antiemetics: domperidone, chlorpromazine, ondansetron
13. Increased risk of salivary gland hypofunction (dryness of the mouth, swelling under the tongue and/or below the ear, halitosis)
14. History of any other illness or condition which, in the investigator's judgment, may substantially increase the risk associated with the subject's participation in the protocol or compromise the scientific objectives, or other condition(s) that, in the opinion of the investigator, would jeopardize the safety or rights of a subject participating in the trial, interfere with the evaluation of the study objectives, or render the subject unable to comply with the protocol.

Study objectives and endpoints

OBJECTIVES	ENDPOINTS
1. Part 1: Safety: To evaluate the safety and tolerability of L9LS administered at doses of 5 mg/kg, 10 mg/kg, and 20 mg/kg by SC administration to healthy Kenyan children.<br>2. Part 2: Safety: To evaluate the safety and tolerability of L9LS administered at doses of 10-20 mg/kg by SC administration to healthy Kenyan children<br>3. Part 2: Efficacy: To assess the efficacy of two doses of L9LS at a concentration of 10-20 mg/kg, administered SC to participants aged 5-59 months of age against first/only Pf malaria infection diagnosed by blood smear microscopy (irrespective of fever) over 12 months compared to placebo.	1. Parts 1 & 2: Incidence and severity of local and systemic AEs occurring within 7 days after the administration of L9LS, and incidence of SAEs throughout the study period.<br>2. Part 2: Pf blood-stage infection as detected by microscopic examination of thick blood smear for 52 weeks after administration of L9LS or placebo.
1. To assess the efficacy of one dose of L9LS at 10-20 mg/kg against first/only Pf malaria infection diagnosed by blood smear microscopy over 3 and 6 months compared to placebo.<br>2. To assess the efficacy of one dose of L9LS at 10-20 mg/kg against first/only Pf malaria infection diagnosed by blood smear microscopy over 12 months compared to placebo.<br>3. To evaluate the efficacy of L9LS at 10-20 mg/kg against first/only Pf malaria infection as detected by PCR over 3, 6 and 12 months compared to placebo.<br>4. To assess protection of L9LS at 10-20 mg/kg against clinical malaria (first/only and all episodes) at 3, 6 and 12 months compared to a placebo.<br>5. To assess the primary objective and secondary objectives 1-4 among children 5-17 months of age.<br>6. To assess the primary objective and secondary objectives 1-4 among children 18-59 months of age.<br>7. To evaluate the PK of L9LS throughout the study at weight-based dose levels of 5 mg/kg, 10 mg/kg, and 20 mg/kg (part 1) and fixed doses of 10-20 mg/kg (part 2) in healthy Kenyan children, and<br>a. To correlate L9LS serum concentration with Pf infection risk.<br>b. To correlate L9LS serum concentration with clinical malaria risk.	1. Pf blood-stage infection as detected by microscopic examination of thick blood smear for 12 and 24 weeks after administration of L9LS or placebo.<br>2. Pf blood-stage infection as detected by microscopic examination of thick blood smear for 52 weeks after administration of L9LS or placebo.<br>3. Pf blood-stage infection as detected by RT-PCR for 12, 24 and 52 weeks after administration of L9LS or placebo.<br>4. Incidence of clinical malaria (definitions 1 and 2) for 12, 24 and 52 weeks after administration of L9LS or placebo.<br>5. See 1 – 4 above<br>6. See 1 – 4 above<br>7. Measurement of L9LS in sera of recipients, including PK analysis of L9LS and the association of L9LS concentration with Pf infection risk and with clinical malaria risk.
1. To assess the impact of L9LS on hospitalizations with malaria	1. Proportion of participants hospitalized with malaria

OBJECTIVES	ENDPOINTS
2. To determine whether ADA to L9LS can be detected in sera of recipients at specific timepoints throughout the study.<br>3. To assess for IgG1 allotypes and allotype specific effects on L9LS PK.<br>4. To determine if the efficacy of L9LS is specific to certain Pf parasite genotypes at the CSP locus.<br>5. To explore the impact of pre-existing parasitemia on the protective efficacy and PK of L9LS.<br>6. To explore the impact of pre-existing CSP antibodies on the protective efficacy and PK of L9LS.<br>7. To assess the effect of L9LS on antibodies to measles in the younger age group.<br>8. To compare the efficacy of one versus two doses of L9LS against Pf infection and clinical malaria over 12 months.	2. Measurement of ADA to L9LS in sera of recipients.<br>3. Assessment of IgG1 allotypes and allotype-specific effects on L9LS PK.<br>4. CSP genotyping of parasites isolated from study subjects.<br>5. Pre-existing parasitemia detected by microscopic examination of thick blood smears or RT-PCR before L9LS administration.<br>6. Pre-existing CSP-specific antibodies measured in sera collected before L9LS administration.<br>7. Measles virus-specific IgG antibodies among subjects <9 months of age at enrollment.<br>8. Pf infection and clinical malaria over 12 months.
Follow-on Safety (part 1b)<br>1. To evaluate the safety and tolerability of L9LS administered at doses of 30 mg/kg and 40 mg/kg by SC administration to healthy Kenyan children.<br>*Follow-on Part 2 Extension*<br>2. To evaluate the safety and tolerability of a third and fourth SC dose of L9LS at 20–40 mg/kg (compared to placebo) in healthy Kenyan children.<br>3. To evaluate the PK of L9LS throughout the follow-up phase at 20–40 mg/kg in healthy Kenyan children.<br>4. To determine if ADAs to L9LS can be detected in sera of recipients at specific timepoints throughout the study and to correlate the occurrence of ADAs with L9LS PK.	1. Incidence and severity of local and systemic AEs occurring within 7 days after the administration of L9LS and incidence of serious adverse events (SAEs) throughout the study period.<br>2. Measurement of L9LS in sera of recipients.<br>3. Measurement of ADA in sera of recipients.
1. To assess the efficacy of an additional one or two SC doses of L9LS over one year at 20–40 mg/kg in mediating protection against Pf infection and clinical malaria in healthy Kenyan children during a second year of follow-up compared to those who received placebo only (arms 1 vs. 3; arms 2 vs. 3) and in those receiving two L9LS doses in the extension versus one (arms 1 vs. 2)<br>2. To evaluate the PK of L9LS throughout the study and to correlate L9LS serum concentration with Pf infection risk and clinical malaria risk<br>3. To evaluate the effect of a third (and fourth) dose of L9LS on development of acquired immunity (measured by antibodies)	1. Pf blood-stage infection as detected by microscopic examination of thick blood smears obtained between 1 week and 52 weeks after administration of L9LS or placebo.<br>2. Pf blood-stage infection as detected by RT-PCR from dried blood spots obtained between 1 week and 52 weeks after administration of L9LS or placebo.<br>3. Incidence of clinical malaria () between 1 week and 52 weeks after administration of L9LS or placebo.<br>4. PK analysis of L9LS and the association of L9LS concentration with Pf infection risk.

OBJECTIVES	ENDPOINTS
	5. PK analysis of L9LS and the association of L9LS concentration with clinical malaria risk.<br>6. Levels of naturally occurring antimalaria antibodies in participant blood at selected time points

Grading and relatedness of solicited adverse events

The following job aide was used to assist in grading solicited adverse events (local and systemic) and in determining their relatedness.

SOLICITED LOCAL ADVERSE EVENTS (REACTOGENICITY)

**Local reaction**	**Grade 0**	**Mild Grade 1**	**Moderate Grade 2**	**Severe Grade 3**	**Life threatening Grade 4**
**Pain at injection site**	Absent	Minor disturbance Child moves arm normally	Pain when moving the affected limb, young child avoids moving the affected arm or leg	Cries/ protests when the affected limb is moved, pain disturbs sleep	Older child complains of severe pain, refuses to move the affected limb; young child cries inconsolably when limb is moved
**Tenderness**	Absent	Minor reaction to touch	Cries/ protests on touch	Cries/ protests when the affected limb is moved	Older child complains of severe pain, young child cries inconsolably when injection site is touched
**Redness**	Absent	< 10 mm	10-25 mm	26-50 mm	>50 mm
**Swelling**	Absent	< 10 mm	10-25 mm	26-50 mm	>50 mm
**Bruising**	Absent	< 10 mm	10-25 mm	Ulceration	Necrosis
**Pruritus**	Absent	Minor disturbance	Frequent scratching, young child cries more than usual	Constant scratching, disturbs sleep	Older child complains of severe pruritus; young child cries inconsolably because of pruritus

Relatedness: Most local reactions will be 'definitely related' unless there is another explanation like an insect bite or a burn at the injection site.

- **Relatedness coding:**

- **1 = *Unrelated* - this can only be chosen when the alternative etiology is the only cause of the symptom**
- **2 = *Unlikely Related* - this is the more commonly used option, there is an alternative etiology, and it is the most likely cause of the symptom**
- **3 = *Possibly Related* - while there is an alternative etiology, it is less likely to be the cause of the symptom**
- **4 = *Probably Related* - there is no evident alternative etiology, but the investigations were not exhaustive**
- **5 = *Definitely Related* - all other causes have been ruled out**

SOLICITED SYSTEMIC ADVERSE EVENTS

Systemic Reaction	Grade 0	Mild Grade 1	Moderate Grade 2	Severe Grade 3	Life threatening Grade 4
**Fever (axillary temperature) In Degree Celsius[1]**	35.5–37.4 °C	37.5 –38.5 °C	38.6–39.5 °C	39.6–40 °C	40.1 °C and above
**Malaise**	Absent	Easily tolerated	Interferes with daily activity	Prevents normal activity/ lethargic	Wants to sleep/ lie in bed all the time
**Muscle ache**	Absent	Crying/ complaining more than usual when moving, no effect on normal activity	Crying/ complaining more than usual when moving, interferes with normal activity	Crying/ complaining a lot when moving, prevents normal activity	Inconsolable, crying that cannot be comforted, child refuses to move
**Headache / Irritability**	Absent	Complaining of headache, crying more than usual, no effect on normal activity	Complaining of headache, crying more than usual, interferes with normal activity	Complaining of headache, crying a lot, prevents normal activity	Inconsolable, crying that cannot be comforted
**Chills**	Absent	Easily tolerated	Interferes with normal activity	Prevents normal activity	Needs medical intervention
**Nausea**	Absent	Loss of appetite without alteration in eating habits	Oral intake decreased without significant dehydration, possibly vomited once since last assessment	Vomited more than once since last assessment, dehydration present, needs outpatient treatment	Dehydration in need of hospitalization/ i.v. fluids
**Joint pain**	Absent	Crying/ complaining more than usual when joint is moved, no effect on normal activity	Crying/ complaining when joint is moved, interferes with normal activity	Crying/ complaining a lot when joint is moved, prevents normal activity	Inconsolable, crying that cannot be comforted when joint is moved, possible joint swelling

Relatedness as above

[1] Fever grading in part 2 was changed to the following: Grade 1: 37.5 – 38.4; Grade 2: 38.5 – 39.4; Grade 3: 39.5 – 41.9; Grade 4: ≥ 42 ºC

Microscopy reading of blood smears

Thick and thin blood smears were prepared at the laboratory using 6 µL and 2 µL of blood respectively, giemsa stained and microscopically examined following High-Powered Fields (HPFs) method according to WHO malaria microscopy standards in a research setting.[1]

All smears were independently examined by two microscopists who had passed an external quality assurance program provided by the Contract Laboratory Services (CLS), Johannesburg, South Africa and certified at the equivalent of WHO competence level 1 or 2 for the accuracy in detection of, species identification, and parasite counts.[2] Detection and quantification of parasite were done following High-Powered Fields (HPFs) method.[1]

Thick and thin blood smears were prepared, stained and examined under the microscopes by competent readers according to WHO malaria microscopy standards in a research setting.

Blood smear reads were considered discordant if they differed on malaria parasite detection (positive/negative) or species identification. Additionally, reads were considered discordant if they differed quantitatively by the following parameters:

- For high and medium parasitemia results (parasite density ≥400 parasites/µL): if the higher count divided by the lower count is ≥2
- For low parasitemia results (parasite density ≤400 parasites/µL): if the higher count divided by the lower count is ≥10
- If one parasitemia result is ≥400 parasites/µL and the other is ≤400 parasites/µL: if the higher count divided by the lower count is ≥10

A third microscopist, blinded to the results of prior examinations, confirmed discordant results. Where there was a difference on malaria parasite detection or species identification, the majority decision obtained following the reading by the third microscopist was adopted; if positive, the final result was the mean of the two positive results. Where all three readings were positive, the final result was the mean of the two closest readings.

Anti-drug antibody (ADA) analyses

A three-tiered qualified assay approach was used to detect anti-drug antibodies (ADAs) on a Beckman Biomek i7 liquid handler. The tier 1 assay, based on the Meso Scale Diagnostics (MSD) electroluminescence bridging assay, was used to screen for the presence of ADAs in the sera. Serum was diluted 1:2 and incubated with Sulfo-tag L9LS (reporter molecule) and biotinylated L9LS (capture molecule) for two hours at room temperature. Following incubation, sample-L9LS mixture was added to blocked streptavidin-coated 384-well MSD plates and incubated at room temperature on Heidolph Titramax 1000 plate shaker with speed between 300-700 RPM for 3 hours ± 10 minutes. Following incubation, MSD Read Buffer was added to each well. Luminescence intensity (ECL unit) was measured on an MSD Sector S600 plate reader. Samples with ECL signal above the floating positivity cut-point of the assay were reported as tier 1 ADA positive. Samples that tested positive in the screening assay were subjected to a confirmatory tier 2 assay to assess specificity of tier 1 positive samples against L9LS. The tier 2 confirmatory assay was performed following the same procedures described for tier 1 assay except for a drug product pre-incubation step. In the tier 2 assay, undiluted samples were incubated with and without unlabeled L9LS (10 ug/mL) for one hour at 37°C. Positivity in the tier 2 assay was determined based on the percent inhibition which was calculated by comparing raw ECL signal in the competed sample to the un-competed sample relative to a fixed positivity cutpoint. Tier 2 positive samples were tested in a tier 3 competitive target binding assay of L9LS to its cognate antigens to determine if the identified ADA may be functionally inhibitory. To perform the tier 3 assay, MSD 96-well plates were coated with L9LS overnight, washed then blocked in MSD blocking buffer for 1 hour at room temperature. Plates were washed and 1:2 diluted test sample was added in duplicate for 1 hour at room temperature, shaking. Plates were again washed and biotinylated CSP protein was added for 1 hour at room temperature, shaking. Plates were washed and MSD Sulfo-tag streptavidin detection was added for 1 hour at room

temperature, shaking, to detect bound biotinylated CSP. Plates were washed and MSD Read Buffer was added to each well. Luminescence intensity (ECL unit) was measured on an MSD Sector S600 plate reader. Percent inhibition was calculated as signal observed in test sample relative to a negative control, where presence of functional ADA would reduce the ability of biotinylated CSP to bind the L9LS coated on the plate and lead to a signal reduction. Percent inhibition above the assay limit of detection was quantified.

To determine if identified ADA was specific to the Fab portion of L9LS, a research-grade assay was developed using purified Fab preparations of L9LS (as well as anti-malarial CIS43LS and anti-HIV VRC07-523LS as non-specific comparators). Fab fragments were coated on 96-well MSD plates overnight. Plates were washed then blocked in MSD blocking buffer for 1 hour at room temperature. Plates were washed and serial dilutions of test sample in duplicate were added for 2 hours at room temperature, shaking. Plates were again washed and MSD Sulfo-tag anti-human IgG Fc-specific detection was added for 1 hour at room temperature, shaking. Plates were washed and MSD Read Buffer was added to each well. Luminescence intensity (ECL unit) was measured on an MSD Sector S600 plate reader.

Electronic data capture

The DFdiscover clinical data management system developed by NIH was used to collect data for this trial. DFdiscover is an agile clinical trial data management solution used to collect data from a variety of sources, including paper, electronic data capture (EDC), and tablets (both online and offline), and provides tools that support the management, verification, and analysis of the clinical data.

DFdiscover secures data with AES 256 encryption and is fully compliant with HIPAA, GDPR, FDA 21 CFR Part 11 regulations and ISO 9001:2015 certified.

Additional details on statistical analyses

Efficacy

To analyze the PE against the repeated occurrence of events, we used the Anderson-Gill method, an extension of the Cox proportional hazards model for recurrent events. In this analysis, participants during the 14 days following treatment with artemether lumefantrine, and 28 days following treatment with dihydroartemisinin piperaquine, were removed from the risk set. Because some participants received these antimalarial treatments before their first infection (e.g., from a non-study clinic prior to their first positive blood smear at the study clinic), a sensitivity analysis was done for the time to first infection using the Anderson-Gill method up to the first infection (Figure S5).

Pharmacokinetics

A non-compartmental (NC) PK analysis was performed on the L9LS concentration data from each participant. Individual participant and dosing arm concentration-versus-time profiles were constructed in semi-log scales, and maximum concentrations (Cmax) were summarized as geometric means and geometric coefficients of variation by dose and age group directly from the observed data. The time of maximum concentration (Tmax) was summarized as the mean and standard deviation by dose and age group from the observed data. Terminal elimination half-lives were estimated from observed data from Part 2 1-dose participants who had L9LS serum concentrations from 3 time points by fitting the natural logarithm of concentration by time using the PKNCA package, version 0.12.1. Given sparse data, options were specified to include Tmax and ≥3 time points in the half-life calculation as to allow inclusion of the first (day 28) time point, which was the observed Tmax for Part 2 participants. Since none of the

participants who had 3 PK observations had observations prior to day 22 post-L9LS administration, the area under the concentration-time curve during the entire period of drug exposure ($AUC_{0\text{-last}}$) could not be reliably determined.

To compare the slopes between dosing groups in Part 1 participants, linear mixed effects models were evaluated using the lmerTest package. Separate models were evaluated for Part 1 participants for whom the last observed concentration was either at day 28 or at day 84. In each model, L9LS concentration was the dependent variable; time (day) and dose were independent variables with an interaction term between dose and time (dose * day); and participant was modeled as a random effect. Reference level for interaction is 40 mg/kg * day.

References

1. World Health Organization. Microscopy for the detection, identification and quantification of malaria parasites on stained thick and thin blood films in research settings. . Geneva, 2015.
2. Swysen C, Vekemans J, Bruls M, et al. Development of standardized laboratory methods and quality processes for a phase III study of the RTS, S/AS01 candidate malaria vaccine. *Malar J* 2011; **10**: 223.

Supplementary Tables and Figures

Tables

Table S 1: Baseline characteristics of participants in part 1a 14
Table S 2: Baseline characteristics of participants in part 1b 15
Table S 3: Summary of adverse events in safety cohorts 17
Table S 4 Listing of unsolicited adverse events in part 1a 20
Table S 5 Listing of unsolicited adverse events in part 1b 26
Table S 6: Listing of serious adverse events (SAEs) 29
Table S 7: Listing of unsolicited adverse events in part 2 31
Table S 8: Proportion with P· falciparum infection and clinical malaria at 6 and 12 months, by study group (n/N) . 36
Table S 9: Incidence of Pf infection and clinical malaria (infections/episodes/person-year) by time period and age group 43
Table S 10: C_{max} by dose received and age group 54
Table S 11: Linear mixed effects models examining differences in concentration versus time slopes by dosing arm 56
Table S 12: Weight-based dosing bands and resulting L9LS concentrations in Part 2 61

Figures

Figure S 1: Consort diagrams for age de-escalation, dose escalation safety studies 16
Figure S 2: Kaplan-Meier curves of time to first P. falciparum infection as detected by blood smear, by treatment arm and age group 36
Figure S 3 Protective efficacy of one or two doses of L9LS against P· falciparum infection and clinical malaria at six and 12 months, as compared to placebo, by time-to-event analysis, by age group 38
Figure S 4: Kaplan-Meier curve of time to first clinical malaria, by treatment arm and age group 39
Figure S 5: Protective efficacy of one dose of L9LS against P. falciparum infection and clinical malaria at three months, as compared to placebo, by time-to-event analysis, overall and by age group 40
Figure S 6: Protective efficacy of one or two doses of L9LS against *P. falciparum* infection and clinical malaria at six and 12 months, as compared to placebo, by Kaplan-Meier proportional analysis, overall and by age group 41
Figure S 7: Protective efficacy of one or two doses of L9LS against *P. falciparum* infection and clinical malaria at six and 12 months, as compared to placebo, by recurrent event analysis, overall and by age group 42
Figure S 8: Protective efficacy of one or two doses of L9LS against P. falciparum infection and clinical malaria at six and 12 months, as compared to placebo, by time-to-event analysis and censoring protected periods, overall and by age group 44
Figure S 9: Protective efficacy of L9LS against P. falciparum infection and clinical malaria at six and 12 months, as compared to placebo, by time-to-event analysis, overall and by age group, using per-protocol population 45
Figure S 10: Protective efficacy of L9LS against P. falciparum infection and clinical malaria at six and 12 months, as compared to placebo, by recurrent event analysis, overall and by age group, using per-protocol population... 46
Figure S 11: Kaplan-Meier curves of P. falciparum infection risk, by blood smear status and by PCR status prior to antimalarial administration, all arms combined 47
Figure S 12: Kaplan-Meier curve of P. falciparum infection risk, by PCR status at baseline, by study arm 48
Figure S 13: Kaplan-Meier curve of P. falciparum infection risk among those positive by PCR at baseline, by age and dose group 49
Figure S 14: Kaplan-Meier curve of P. falciparum infection risk among those negative by PCR at baseline, by age and dose group 50
Figure S 15: Protective efficacy of L9LS against P. falciparum infection and clinical malaria at six and 12 months, as compared to placebo, by time-to-event analysis, by PCR status at baseline 51
Figure S 16 Protective efficacy of one or two doses of L9LS, using time-to-first event analysis with interval censoring and controlling for month of enrollment 52

Figure S 17: Mean L9LS serum concentrations versus time, by dose and age groups· 53
Figure S 18: Serum concentrations by L9LS dose and last measured concentration... 55
Figure S 19: Geometric mean serum concentrations versus time at 5 mg/kg L9LS administered subcutaneously (SC) in current study participants compared to US adults from the VRC 614 study... 57
Figure S 20: Comparison of estimated L9LS half-lives between age groups 58
Figure S 21: Relationship between age and estimated L9LS half life 59
Figure S 22: Antigen-specific fragment binding of study participant exhibiting ADA against L9LS 60
Figure S 23: Weight-based L9LS dose concentrations at dose 1, by age group 62
Figure S 24: Plots of protective efficacy versus dose for Pf infection and clinical malaria endpoints 63

Table S 1: Baseline characteristics of participants in part 1a

	5-10 years of age				5-59 months of age			
**Characteristic**	**10 mg/kg L9LS** N = 9[1]	**20 mg/kg L9LS** N = 9[1]	**5 mg/kg L9LS** N = 9[1]	**Placebo** N = 9[1]	**10 mg/kg L9LS** N = 9[1]	**20 mg/kg L9LS** N = 9[1]	**5 mg/kg L9LS** N = 9[1]	**Placebo** N = 9[1]
**Sex**								
Female	7 (78%)	4 (44%)	6 (67%)	5 (56%)	3 (33%)	6 (67%)	6 (67%)	5 (56%)
Male	2 (22%)	5 (56%)	3 (33%)	4 (44%)	6 (67%)	3 (33%)	3 (33%)	4 (44%)
**Age (months)**	84 [72, 89]	90 [68, 109]	104 [67, 109]	98 [75, 111]	28 [17, 44]	39 [27, 43]	33 [29, 34]	24 [11, 46]
**Weight at dose 1 (kg)**	20.6 (2.9)	22.7 (3.4)	22.9 (4.6)	24.5 (3.8)	13.5 (2.5)	13.9 (2.0)	13.1 (2.5)	12.2 (2.6)
**Pos smear at baseline**	3 (33%)	3 (33%)	3 (33%)	2 (22%)	0 (0%)	1 (11%)	3 (33%)	2 (22%)
**Pos PCR at baseline**	4 (44%)	4 (44%)	5 (56%)	3 (33%)	0 (0%)	4 (44%)	4 (44%)	2 (25%)

[1]n (%); Median [Q1, Q3]; Mean (SD).

Note: Baseline represents pre-enrollment visit for giving dihydroartemisinin-piperaquine for parasite clearance, two to three weeks prior to antibody administration.

Blood smear and PCR positivity are only for plasmodium falciparum. PCR unable to be run on 1 baseline sample (younger child randomized to placebo within 20 mg/kg group). Other variables have no missing values.

Table S 2: Baseline characteristics of participants in part 1b

Characteristic	30 mg/kg N = 9[1]	40 mg/kg N = 9[1]	Placebo N = 6[1]
**Sex**			
Female	5 (56%)	1 (11%)	2 (33%)
Male	4 (44%)	8 (89%)	4 (67%)
**Age (months)**	38 [21, 57]	12 [7, 26]	30 [24, 38]
**Weight at dose 1 (kg)**	13.9 (3.6)	10.9 (2.7)	13.3 (1.1)
**Pos smear at baseline**	3 (33%)	2 (22%)	1 (17%)
**Pos PCR at baseline**	5 (56%)	4 (44%)	2 (33%)

[1]n (%); Median [Q1, Q3]; Mean (SD)
Note: Baseline represents pre-enrollment visit for giving dihydroartemisinin-piperaquine for parasite clearance, two to three weeks prior to antibody administration.

Blood smear and PCR positivity are only for plasmodium falciparum.

Figure S 1: Consort diagrams for age de-escalation, dose escalation safety studies

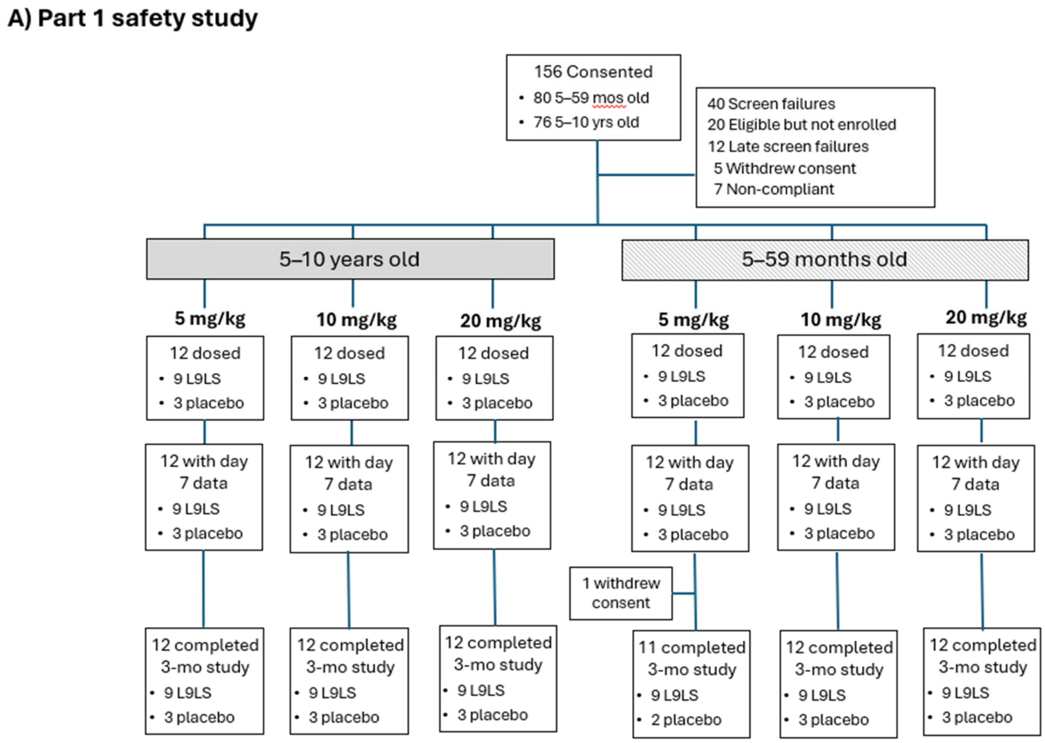

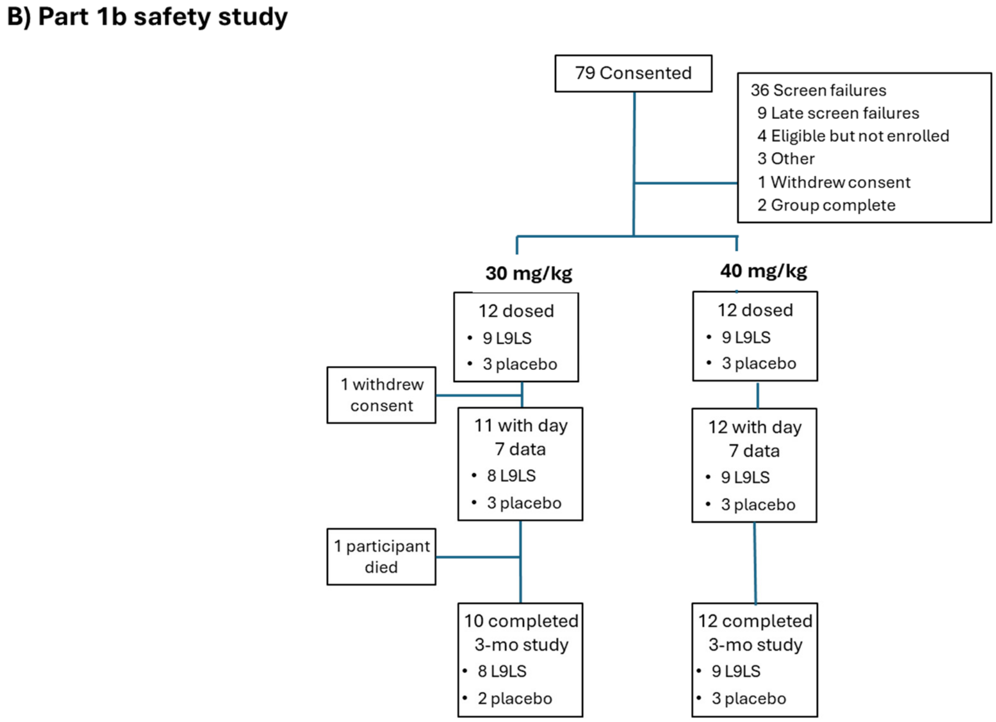

Table S 3: Summary of adverse events in safety cohorts

Part 1a	Older Age Group (5-10 years)				Younger Age Group (5-59 months)			
**Adverse Event**	**5 mg/kg (N=9)**	**10 mg/kg (N=9)**	**20 mg/kg (N=9)**	**Placebo (N=9)**	**5 mg/kg (N=9)**	**10 mg/kg (N=9)**	**20 mg/kg (N=9)**	**Placebo (N=9)**
Participants with at least 1 Solicited Local AE within 7 days of dosing	0 (0.0%)	2 (22.2%)	1 (11.1%)	1 (11.1%)	2 (22.2%)	1 (11.1%)	2 (22.2%)	0 (0.0%)
Participants with at least 1 Solicited Systemic AE within 7 days of dosing	1 (11.1%)	1 (11.1%)	2 (22.2%)	0 (0.0%)	0 (0.0%)	0 (0.0%)	0 (0.0%)	2 (22.2%)
**Local reactogenicity AEs**								
Injection site pain								
Mild	0 (0.0%)	1 (11.1%)	1 (11.1%)	0 (0.0%)	1 (11.1%)	1 (11.1%)	1 (11.1%)	0 (0.0%)
Injection site swelling								
Severe	0 (0.0%)	0 (0.0%)	0 (0.0%)	1 (11.1%)	0 (0.0%)	0 (0.0%)	0 (0.0%)	0 (0.0%)
Moderate	0 (0.0%)	1 (11.1%)	1 (11.1%)	0 (0.0%)	1 (11.1%)	1 (11.1%)	1 (11.1%)	0 (0.0%)
Mild	0 (0.0%)	1 (11.1%)	0 (0.0%)	0 (0.0%)	1 (11.1%)	0 (0.0%)	1 (11.1%)	0 (0.0%)
**Systemic solicited AEs**								
Chills								
Mild	1(11.1%)	0 (0.0%)	0 (0.0%)	0 (0.0%)	0 (0.0%)	0 (0.0%)	0 (0.0%)	0 (0.0%)
Headache								
Mild	0 (0.0%)	1(11.1%)	3 (33.3%)	0 (0.0%)	0 (0.0%)	0 (0.0%)	0 (0.0%)	1(11.1%)
Pyrexia								
Mild	0 (0.0%)	0 (0.0%)	0 (0.0%)	0 (0.0%)	0 (0.0%)	0 (0.0%)	0 (0.0%)	1(11.1%)
**Unsolicited AEs**								
Participants with at least 1 unsolicited AE within 28 days of dosing	7 (77.8%)	5 (55.6%)	8 (88.9%)	6 (66.7%)	9 (100.0%)	9 (100.0%)	7 (77.8%)	9 (100.0%)
Participants with at least 1 related unsolicited AE within 28 days of dosing	0 (0.0%)	0 (0.0%)	1 (11.1%)	1 (11.1%)	1 (11.1%)	0 (0.0%)	0 (0.0%)	1 (11.1%)
**All**								
Participants experiencing a grade 3 related AE (solicited or unsolicited)	0 (0.0%)	0 (0.0%)	0 (0.0%)	1 (11.1%)	0 (0.0%)	0 (0.0%)	0 (0.0%)	0 (0.0%)

Participants experiencing an SAE	0 (0.0%)	0 (0.0%)	0 (0.0%)	0 (0.0%)	0 (0.0%)	0 (0.0%)	0 (0.0%)	0 (0.0%)
Notes: No participants experienced local reactions of injection site tenderness, redness, pruritus, or bruising. No participants experienced systemic solicited events of nausea, muscle aches, or joint pain. All local events were considered related to the study								

**Part 1b Adverse Event**	**30 mg/kg (N=9)**	**40 mg/kg (N=9)**	**Placebo (N=6)**
Participants with at least 1 Solicited Local AE within 7 days of dosing	0 (0.0%)	3 (33.3%)	0 (0.0%)
Participants with at least 1 Solicited Systemic AE within 7 days of dosing	0 (0.0%)	2 (22.2%)	0 (0.0%)
**Local reactogenicity AEs**			
Injection site swelling			
Mild	0 (0.0%)	3 (33.3%)	0 (0.0%)
**Systemic solicited AEs**			
Nausea			
Moderate	0 (0.0%)	1(11.1%)	0 (0.0%)
Pyrexia			
Severe*	0 (0.0%)	1(11.1%)	0 (0.0%)
**Unsolicited AEs**			
Participants with at least 1 unsolicited AE within 28 days of dosing	7 (77.8%)	6 (66.7%)	6 (100.0%)
Participants with at least 1 related unsolicited AE within 28 days of dosing	1 (11.1%)	0 (0.0%)	1 (16.7%)
**All**			
Participants experiencing a grade 3 related AE (solicited or unsolicited)	0 (0.0%)	1(11.1%)	0 (0.0%)
Participants experiencing an SAE	0 (0.0%)	0 (0.0%)	1(11.1%)

Notes: No participants experienced local reactions of injection site tenderness, redness, pruritus, or bruising. No participants experienced systemic solicited events of chills, muscle aches, or joint pain. All local events were considered related to the study

* Severe pyrexia defined as axillary temperature from 38.5 – 39.5 °C.

Table S 4 Listing of unsolicited adverse events in part 1a

Younger children ages 5–59 months

		Arm 1 5 mg/kg L9LS SC		Arm 2 10 mg/kg L9LS SC		Arm 3 20 mg/kg L9LS SC		Arm 4 Placebo SC	
**Primary System Organ Class MedDRA Preferred Term**	**All Study Arms**	**G1 Mild**	**G2 Moderate**	**G1 Mild**	**G2 Moderate**	**G1 Mild**	**G2 Moderate**	**G1 Mild**	**G2 Moderate**
**BLOOD AND LYMPHATIC SYSTEM DISORDERS**	5								
Anaemia	4	1(1)11.1%	0(0)0.0%	1(1)11.1%	0(0)0.0%	0(0)0.0%	0(0)0.0%	2(2)22.2%	0(0)0.0%
Lymphadenitis	1	0(0)0.0%	0(0)0.0%	0(0)0.0%	1(1)11.1%	0(0)0.0%	0(0)0.0%	0(0)0.0%	0(0)0.0%
**EAR AND LABYRINTH DISORDERS**	3								
Ear Pain	2	0(0)0.0%	0(0)0.0%	0(0)0.0%	0(0)0.0%	0(0)0.0%	0(0)0.0%	2(2)22.2%	0(0)0.0%
Excessive Cerumen Production	1	1(1)11.1%	0(0)0.0%	0(0)0.0%	0(0)0.0%	0(0)0.0%	0(0)0.0%	0(0)0.0%	0(0)0.0%
**EYE DISORDERS**	2								
Conjunctivitis Allergic	2	1(1)11.1%	0(0)0.0%	1(1)11.1%	0(0)0.0%	0(0)0.0%	0(0)0.0%	0(0)0.0%	0(0)0.0%
**GASTROINTESTINAL DISORDERS**	12								
Abdominal Pain	4	0(0)0.0%	0(0)0.0%	2(2)22.2%	0(0)0.0%	0(0)0.0%	0(0)0.0%	2(2)22.2%	0(0)0.0%
Constipation	1	0(0)0.0%	0(0)0.0%	1(1)11.1%	0(0)0.0%	0(0)0.0%	0(0)0.0%	0(0)0.0%	0(0)0.0%
Diarrhoea	5	1(1)11.1%	0(0)0.0%	2(2)22.2%	0(0)0.0%	0(0)0.0%	0(0)0.0%	2(2)22.2%	0(0)0.0%
Vomiting	2	0(0)0.0%	0(0)0.0%	0(0)0.0%	0(0)0.0%	1(1)11.1%	0(0)0.0%	1(1)11.1%	0(0)0.0%
**GENERAL DISORDERS AND ADMINISTRATION SITE CONDITIONS**	15								
Feeling Hot	14	1(1)11.1%	0(0)0.0%	6(5)55.6%	0(0)0.0%	2(2)22.2%	0(0)0.0%	5(2)22.2%	0(0)0.0%
Pyrexia	1	1(1)11.1%	0(0)0.0%	0(0)0.0%	0(0)0.0%	0(0)0.0%	0(0)0.0%	0(0)0.0%	0(0)0.0%
**INFECTIONS AND INFESTATIONS**	135								
Acrodermatitis	1	1(1)11.1%	0(0)0.0%	0(0)0.0%	0(0)0.0%	0(0)0.0%	0(0)0.0%	0(0)0.0%	0(0)0.0%
Body Tinea	1	0(0)0.0%	0(0)0.0%	1(1)11.1%	0(0)0.0%	0(0)0.0%	0(0)0.0%	0(0)0.0%	0(0)0.0%
Cellulitis	1	0(0)0.0%	0(0)0.0%	0(0)0.0%	0(0)0.0%	0(0)0.0%	0(0)0.0%	1(1)11.1%	0(0)0.0%
Conjunctivitis	12	2(2)22.2%	0(0)0.0%	6(5)55.6%	0(0)0.0%	0(0)0.0%	0(0)0.0%	4(3)33.3%	0(0)0.0%
Dysentery	1	0(0)0.0%	0(0)0.0%	1(1)11.1%	0(0)0.0%	0(0)0.0%	0(0)0.0%	0(0)0.0%	0(0)0.0%

		Arm 1<br>5 mg/kg L9LS SC		Arm 2<br>10 mg/kg L9LS SC		Arm 3<br>20 mg/kg L9LS SC		Arm 4<br>Placebo SC	
**Primary System Organ Class<br>MedDRA Preferred Term**	**All Study Arms**	**G1 Mild**	**G2 Moderate**	**G1 Mild**	**G2 Moderate**	**G1 Mild**	**G2 Moderate**	**G1 Mild**	**G2 Moderate**
Gastroenteritis	19	2(2)22.2%	0(0)0.0%	8(6)66.7%	2(2)22.2%	2(2)22.2%	0(0)0.0%	5(3)33.3%	0(0)0.0%
Helminthic Infection	1	0(0)0.0%	0(0)0.0%	0(0)0.0%	0(0)0.0%	1(1)11.1%	0(0)0.0%	0(0)0.0%	0(0)0.0%
Hepatitis A	1	0(0)0.0%	0(0)0.0%	0(0)0.0%	0(0)0.0%	1(1)11.1%	0(0)0.0%	0(0)0.0%	0(0)0.0%
Malaria	5	3(2)22.2%	0(0)0.0%	0(0)0.0%	0(0)0.0%	1(1)11.1%	0(0)0.0%	0(0)0.0%	1(1)11.1%
Otitis Externa	1	0(0)0.0%	0(0)0.0%	0(0)0.0%	0(0)0.0%	0(0)0.0%	0(0)0.0%	1(1)11.1%	0(0)0.0%
Otitis Media	3	0(0)0.0%	0(0)0.0%	1(1)11.1%	0(0)0.0%	0(0)0.0%	0(0)0.0%	1(1)11.1%	1(1)11.1%
Otitis Media Acute	1	0(0)0.0%	0(0)0.0%	0(0)0.0%	1(1)11.1%	0(0)0.0%	0(0)0.0%	0(0)0.0%	0(0)0.0%
Pertussis	1	0(0)0.0%	0(0)0.0%	0(0)0.0%	0(0)0.0%	0(0)0.0%	0(0)0.0%	1(1)11.1%	0(0)0.0%
Pneumonia	5	0(0)0.0%	2(2)22.2%	2(2)22.2%	0(0)0.0%	0(0)0.0%	0(0)0.0%	0(0)0.0%	1(1)11.1%
Rash Pustular	4	2(2)22.2%	0(0)0.0%	1(1)11.1%	0(0)0.0%	0(0)0.0%	0(0)0.0%	1(1)11.1%	0(0)0.0%
Rhinitis	3	1(1)11.1%	0(0)0.0%	0(0)0.0%	0(0)0.0%	1(1)11.1%	1(1)11.1%	0(0)0.0%	0(0)0.0%
Septic Rash	2	1(1)11.1%	0(0)0.0%	0(0)0.0%	0(0)0.0%	0(0)0.0%	1(1)11.1%	0(0)0.0%	0(0)0.0%
Tinea Capitis	1	0(0)0.0%	0(0)0.0%	0(0)0.0%	0(0)0.0%	1(1)11.1%	0(0)0.0%	0(0)0.0%	0(0)0.0%
Tonsillitis	7	0(0)0.0%	0(0)0.0%	4(4)44.4%	1(1)11.1%	2(2)22.2%	0(0)0.0%	0(0)0.0%	0(0)0.0%
Upper Respiratory Tract Infection	61	16(9)100.0%	0(0)0.0%	15(9)100.0%	2(2)22.2%	14(8)88.9%	0(0)0.0%	14(8)88.9%	0(0)0.0%
Upper Respiratory Tract Infection Bacterial	1	0(0)0.0%	0(0)0.0%	1(1)11.1%	0(0)0.0%	0(0)0.0%	0(0)0.0%	0(0)0.0%	0(0)0.0%
Varicella	1	0(0)0.0%	0(0)0.0%	0(0)0.0%	0(0)0.0%	0(0)0.0%	0(0)0.0%	1(1)11.1%	0(0)0.0%
Wound Sepsis	2	0(0)0.0%	0(0)0.0%	0(0)0.0%	0(0)0.0%	0(0)0.0%	0(0)0.0%	2(2)22.2%	0(0)0.0%
**INJURY, POISONING AND PROCEDURAL COMPLICATIONS**	3								
Soft Tissue Injury	1	0(0)0.0%	0(0)0.0%	1(1)11.1%	0(0)0.0%	0(0)0.0%	0(0)0.0%	0(0)0.0%	0(0)0.0%
Thermal Burn	1	0(0)0.0%	0(0)0.0%	0(0)0.0%	0(0)0.0%	1(1)11.1%	0(0)0.0%	0(0)0.0%	0(0)0.0%
Wound	1	0(0)0.0%	1(1)11.1%	0(0)0.0%	0(0)0.0%	0(0)0.0%	0(0)0.0%	0(0)0.0%	0(0)0.0%
**INVESTIGATIONS**	1								
Alanine Aminotransferase Increased	1	0(0)0.0%	0(0)0.0%	1(1)11.1%	0(0)0.0%	0(0)0.0%	0(0)0.0%	0(0)0.0%	0(0)0.0%
**METABOLISM AND NUTRITION DISORDERS**	2								

		Arm 1<br>5 mg/kg L9LS SC		Arm 2<br>10 mg/kg L9LS SC		Arm 3<br>20 mg/kg L9LS SC		Arm 4<br>Placebo SC	
**Primary System Organ Class<br>MedDRA Preferred Term**	**All Study Arms**	**G1 Mild**	**G2 Moderate**	**G1 Mild**	**G2 Moderate**	**G1 Mild**	**G2 Moderate**	**G1 Mild**	**G2 Moderate**
Decreased Appetite	2	1(1)11.1%	0(0)0.0%	0(0)0.0%	0(0)0.0%	0(0)0.0%	0(0)0.0%	1(1)11.1%	0(0)0.0%
**NERVOUS SYSTEM DISORDERS**	1								
Headache	1	1(1)11.1%	0(0)0.0%	0(0)0.0%	0(0)0.0%	0(0)0.0%	0(0)0.0%	0(0)0.0%	0(0)0.0%
**RESPIRATORY, THORACIC AND MEDIASTINAL DISORDERS**	6								
Asthma	1	0(0)0.0%	0(0)0.0%	1(1)11.1%	0(0)0.0%	0(0)0.0%	0(0)0.0%	0(0)0.0%	0(0)0.0%
Bronchospasm	4	1(1)11.1%	0(0)0.0%	1(1)11.1%	0(0)0.0%	0(0)0.0%	1(1)11.1%	1(1)11.1%	0(0)0.0%
Cough	1	0(0)0.0%	0(0)0.0%	0(0)0.0%	0(0)0.0%	1(1)11.1%	0(0)0.0%	0(0)0.0%	0(0)0.0%
**SKIN AND SUBCUTANEOUS TISSUE DISORDERS**	14								
Dermatitis Allergic	1	0(0)0.0%	0(0)0.0%	1(1)11.1%	0(0)0.0%	0(0)0.0%	0(0)0.0%	0(0)0.0%	0(0)0.0%
Eczema	1	0(0)0.0%	0(0)0.0%	1(1)11.1%	0(0)0.0%	0(0)0.0%	0(0)0.0%	0(0)0.0%	0(0)0.0%
Pruritus	1	0(0)0.0%	0(0)0.0%	1(1)11.1%	0(0)0.0%	0(0)0.0%	0(0)0.0%	0(0)0.0%	0(0)0.0%
Rash	1	1(1)11.1%	0(0)0.0%	0(0)0.0%	0(0)0.0%	0(0)0.0%	0(0)0.0%	0(0)0.0%	0(0)0.0%
Rash Papular	6	2(1)11.1%	0(0)0.0%	0(0)0.0%	0(0)0.0%	1(1)11.1%	0(0)0.0%	2(2)22.2%	1(1)11.1%
Rash Pruritic	1	0(0)0.0%	0(0)0.0%	0(0)0.0%	0(0)0.0%	0(0)0.0%	0(0)0.0%	1(1)11.1%	0(0)0.0%
Rash Vesicular	3	2(2)22.2%	1(1)11.1%	0(0)0.0%	0(0)0.0%	0(0)0.0%	0(0)0.0%	0(0)0.0%	0(0)0.0%

(1) Treatment: Arm 1: 5 mg/kg of Study Drug L9LS SC; Arm 2: 10 mg/kg of Study Drug L9LS SC; Arm 3: 20 mg/kg of Study Drug L9LS SC; Arm 4: Placebo SC.
(2) MedDRA: Medical Dictionary for Regulatory Activities. Reported terms were coded using MedDRA version 24.1.
(3) Severity: Blank; Grade1=Mild; Grade2=Moderate; Grade3=Severe; Grade4=Potentially Life Threatening; Grade5=Death.
(4) N=Total Number of Participants who have received the Study Drug L9LS Product or Placebo. Each Field has a format # (X) %. Where # is the Number of Adverse Events. “X” is the Number of Participants with one or more episodes of the given event. % is the Number of Participants with one or more episodes of the given event divided by the Total Number of Participants in the Arm.

Older children ages 5–10 years

		Arm 1<br>5 mg/kg L9LS SC		Arm 2<br>10 mg/kg<br>L9LS SC	Arm 3<br>20 mg/kg L9LS SC		Arm 4<br>Placebo SC		
**Primary System Organ Class<br>MedDRA Preferred Term**	**All Study Arms**	**G1 Mild**	**G2 Moderate**	**G1 Mild**	**G1 Mild**	**G2 Moderate**	**G1 Mild**	**G2 Moderate**	**G3 Severe**
**BLOOD AND LYMPHATIC SYSTEM DISORDERS**	2								
Anaemia	2	1(1)11.1%	0(0)0.0%	1(1)11.1%	0(0)0.0%	0(0)0.0%	0(0)0.0%	0(0)0.0%	0(0)0.0%
**EAR AND LABYRINTH DISORDERS**	1								
Cerumen Impaction	1	1(1)11.1%	0(0)0.0%	0(0)0.0%	0(0)0.0%	0(0)0.0%	0(0)0.0%	0(0)0.0%	0(0)0.0%
**EYE DISORDERS**	1								
Conjunctivitis Allergic	1	0(0)0.0%	0(0)0.0%	0(0)0.0%	1(1)11.1%	0(0)0.0%	0(0)0.0%	0(0)0.0%	0(0)0.0%
**GASTROINTESTINAL DISORDERS**	17								
Abdominal Pain	4	0(0)0.0%	0(0)0.0%	0(0)0.0%	3(2)22.2%	0(0)0.0%	1(1)11.1%	0(0)0.0%	0(0)0.0%
Dental Caries	2	0(0)0.0%	0(0)0.0%	1(1)11.1%	1(1)11.1%	0(0)0.0%	0(0)0.0%	0(0)0.0%	0(0)0.0%
Dental Discomfort	1	0(0)0.0%	0(0)0.0%	0(0)0.0%	1(1)11.1%	0(0)0.0%	0(0)0.0%	0(0)0.0%	0(0)0.0%
Diarrhoea	2	0(0)0.0%	0(0)0.0%	0(0)0.0%	2(2)22.2%	0(0)0.0%	0(0)0.0%	0(0)0.0%	0(0)0.0%
Gastritis	3	1(1)11.1%	0(0)0.0%	1(1)11.1%	1(1)11.1%	0(0)0.0%	0(0)0.0%	0(0)0.0%	0(0)0.0%
Nausea	1	0(0)0.0%	0(0)0.0%	0(0)0.0%	1(1)11.1%	0(0)0.0%	0(0)0.0%	0(0)0.0%	0(0)0.0%
Toothache	2	0(0)0.0%	0(0)0.0%	1(1)11.1%	0(0)0.0%	1(1)11.1%	0(0)0.0%	0(0)0.0%	0(0)0.0%
Vomiting	2	0(0)0.0%	0(0)0.0%	1(1)11.1%	1(1)11.1%	0(0)0.0%	0(0)0.0%	0(0)0.0%	0(0)0.0%
**GENERAL DISORDERS AND ADMINISTRATION SITE CONDITIONS**	10								
Feeling Hot	9	2(2)22.2%	0(0)0.0%	4(3)33.3%	2(1)11.1%	0(0)0.0%	1(1)11.1%	0(0)0.0%	0(0)0.0%
Pyrexia	1	1(1)11.1%	0(0)0.0%	0(0)0.0%	0(0)0.0%	0(0)0.0%	0(0)0.0%	0(0)0.0%	0(0)0.0%
**INFECTIONS AND INFESTATIONS**	100								
Abscess	2	1(1)11.1%	0(0)0.0%	0(0)0.0%	0(0)0.0%	0(0)0.0%	1(1)11.1%	0(0)0.0%	0(0)0.0%
Bacterial Infection	1	1(1)11.1%	0(0)0.0%	0(0)0.0%	0(0)0.0%	0(0)0.0%	0(0)0.0%	0(0)0.0%	0(0)0.0%
Body Tinea	2	1(1)11.1%	0(0)0.0%	0(0)0.0%	0(0)0.0%	0(0)0.0%	1(1)11.1%	0(0)0.0%	0(0)0.0%
Bronchitis	1	0(0)0.0%	0(0)0.0%	1(1)11.1%	0(0)0.0%	0(0)0.0%	0(0)0.0%	0(0)0.0%	0(0)0.0%

		Arm 1<br>5 mg/kg L9LS SC		Arm 2<br>10 mg/kg<br>L9LS SC	Arm 3<br>20 mg/kg L9LS SC		Arm 4<br>Placebo SC		
**Primary System Organ Class<br>MedDRA Preferred Term**	**All Study Arms**	**G1 Mild**	**G2 Moderate**	**G1 Mild**	**G1 Mild**	**G2 Moderate**	**G1 Mild**	**G2 Moderate**	**G3 Severe**
Conjunctivitis	2	1(1)11.1%	0(0)0.0%	0(0)0.0%	1(1)11.1%	0(0)0.0%	0(0)0.0%	0(0)0.0%	0(0)0.0%
Conjunctivitis Bacterial	1	0(0)0.0%	0(0)0.0%	0(0)0.0%	0(0)0.0%	0(0)0.0%	0(0)0.0%	1(1)11.1%	0(0)0.0%
Ear Lobe Infection	1	0(0)0.0%	0(0)0.0%	1(1)11.1%	0(0)0.0%	0(0)0.0%	0(0)0.0%	0(0)0.0%	0(0)0.0%
Gastroenteritis	5	1(1)11.1%	0(0)0.0%	0(0)0.0%	4(3)33.3%	0(0)0.0%	0(0)0.0%	0(0)0.0%	0(0)0.0%
Gingivitis	2	1(1)11.1%	0(0)0.0%	0(0)0.0%	0(0)0.0%	1(1)11.1%	0(0)0.0%	0(0)0.0%	0(0)0.0%
Helminthic Infection	1	0(0)0.0%	0(0)0.0%	0(0)0.0%	0(0)0.0%	0(0)0.0%	1(1)11.1%	0(0)0.0%	0(0)0.0%
Hepatitis A	2	0(0)0.0%	0(0)0.0%	0(0)0.0%	1(1)11.1%	0(0)0.0%	1(1)11.1%	0(0)0.0%	0(0)0.0%
Malaria	15	5(4)44.4%	1(1)11.1%	2(2)22.2%	2(2)22.2%	0(0)0.0%	4(3)33.3%	1(1)11.1%	0(0)0.0%
Oral Pustule	1	1(1)11.1%	0(0)0.0%	0(0)0.0%	0(0)0.0%	0(0)0.0%	0(0)0.0%	0(0)0.0%	0(0)0.0%
Otitis Media	2	0(0)0.0%	0(0)0.0%	0(0)0.0%	0(0)0.0%	1(1)11.1%	0(0)0.0%	1(1)11.1%	0(0)0.0%
Otitis Media Acute	1	0(0)0.0%	0(0)0.0%	0(0)0.0%	1(1)11.1%	0(0)0.0%	0(0)0.0%	0(0)0.0%	0(0)0.0%
Pneumonia	3	0(0)0.0%	0(0)0.0%	0(0)0.0%	0(0)0.0%	3(2)22.2%	0(0)0.0%	0(0)0.0%	0(0)0.0%
Rash Pustular	1	0(0)0.0%	0(0)0.0%	1(1)11.1%	0(0)0.0%	0(0)0.0%	0(0)0.0%	0(0)0.0%	0(0)0.0%
Rhinitis	3	0(0)0.0%	0(0)0.0%	2(2)22.2%	1(1)11.1%	0(0)0.0%	0(0)0.0%	0(0)0.0%	0(0)0.0%
Septic Rash	1	0(0)0.0%	0(0)0.0%	0(0)0.0%	0(0)0.0%	0(0)0.0%	1(1)11.1%	0(0)0.0%	0(0)0.0%
Tinea Capitis	7	1(1)11.1%	0(0)0.0%	1(1)11.1%	2(2)22.2%	1(1)11.1%	2(2)22.2%	0(0)0.0%	0(0)0.0%
Tonsillitis	7	0(0)0.0%	0(0)0.0%	1(1)11.1%	2(1)11.1%	2(2)22.2%	1(1)11.1%	1(1)11.1%	0(0)0.0%
Upper Respiratory Tract Infection	36	9(5)55.6%	2(2)22.2%	11(8)88.9%	9(7)77.8%	0(0)0.0%	5(4)44.4%	0(0)0.0%	0(0)0.0%
Upper Respiratory Tract Infection Bacterial	1	0(0)0.0%	0(0)0.0%	0(0)0.0%	0(0)0.0%	0(0)0.0%	0(0)0.0%	1(1)11.1%	0(0)0.0%
Urinary Tract Infection	1	0(0)0.0%	1(1)11.1%	0(0)0.0%	0(0)0.0%	0(0)0.0%	0(0)0.0%	0(0)0.0%	0(0)0.0%
Viral Rash	1	0(0)0.0%	0(0)0.0%	0(0)0.0%	0(0)0.0%	0(0)0.0%	1(1)11.1%	0(0)0.0%	0(0)0.0%
**INJURY, POISONING AND PROCEDURAL COMPLICATIONS**	4								
Radius Fracture	1	0(0)0.0%	0(0)0.0%	0(0)0.0%	0(0)0.0%	0(0)0.0%	0(0)0.0%	0(0)0.0%	1(1)11.1%
Soft Tissue Injury	1	1(1)11.1%	0(0)0.0%	0(0)0.0%	0(0)0.0%	0(0)0.0%	0(0)0.0%	0(0)0.0%	0(0)0.0%

		Arm 1<br>5 mg/kg L9LS SC		Arm 2<br>10 mg/kg<br>L9LS SC	Arm 3<br>20 mg/kg L9LS SC		Arm 4<br>Placebo SC		
**Primary System Organ Class<br>MedDRA Preferred Term**	**All Study Arms**	**G1 Mild**	**G2 Moderate**	**G1 Mild**	**G1 Mild**	**G2 Moderate**	**G1 Mild**	**G2 Moderate**	**G3 Severe**
Wound	2	1(1)11.1%	0(0)0.0%	0(0)0.0%	1(1)11.1%	0(0)0.0%	0(0)0.0%	0(0)0.0%	0(0)0.0%
**NERVOUS SYSTEM DISORDERS**	4								
Headache	4	0(0)0.0%	0(0)0.0%	1(1)11.1%	2(2)22.2%	0(0)0.0%	1(1)11.1%	0(0)0.0%	0(0)0.0%
**RESPIRATORY, THORACIC AND MEDIASTINAL DISORDERS**	2								
Bronchospasm	2	0(0)0.0%	1(1)11.1%	0(0)0.0%	0(0)0.0%	1(1)11.1%	0(0)0.0%	0(0)0.0%	0(0)0.0%
**SKIN AND SUBCUTANEOUS TISSUE DISORDERS**	7								
Rash	1	1(1)11.1%	0(0)0.0%	0(0)0.0%	0(0)0.0%	0(0)0.0%	0(0)0.0%	0(0)0.0%	0(0)0.0%
Rash Papular	4	1(1)11.1%	0(0)0.0%	0(0)0.0%	2(1)11.1%	0(0)0.0%	1(1)11.1%	0(0)0.0%	0(0)0.0%
Rash Pruritic	2	1(1)11.1%	0(0)0.0%	1(1)11.1%	0(0)0.0%	0(0)0.0%	0(0)0.0%	0(0)0.0%	0(0)0.0%

(1) Treatment: Arm 1: 5 mg/kg of Study Drug L9LS SC; Arm 2: 10 mg/kg of Study Drug L9LS SC; Arm 3: 20 mg/kg of Study Drug L9LS SC; Arm 4: Placebo SC.
(2) MedDRA: Medical Dictionary for Regulatory Activities. Reported terms were coded using MedDRA version 24.1.
(3) Severity: Blank; Grade1=Mild; Grade2=Moderate; Grade3=Severe; Grade4=Potentially Life Threatening; Grade5=Death.
(4) N=Total Number of Participants who have received the Study Drug L9LS Product or Placebo. Each Field has a format # (X) %. Where # is the Number of Adverse Events. “X” is the Number of Participants with one or more episodes of the given event. % is the Number of Participants with one or more episodes of the given event divided by the Total Number of Participants in the Arm.

Table S 5 Listing of unsolicited adverse events in part 1b

		Arm 1 5 mg/kg L9LS SC		Arm 2 10 mg/kg L9LS SC	Arm 3 20 mg/kg L9LS SC		Arm 4 Placebo SC		
**Primary System Organ Class MedDRA Preferred Term**	**All Study Arms**	**G1 Mild**	**G2 Moderate**	**G1 Mild**	**G1 Mild**	**G2 Moderate**	**G1 Mild**	**G2 Moderate**	**G3 Severe**
**BLOOD AND LYMPHATIC SYSTEM DISORDERS**	2								
Anaemia	2	1(1)11.1%	0(0)0.0%	1(1)11.1%	0(0)0.0%	0(0)0.0%	0(0)0.0%	0(0)0.0%	0(0)0.0%
**EAR AND LABYRINTH DISORDERS**	1								
Cerumen Impaction	1	1(1)11.1%	0(0)0.0%	0(0)0.0%	0(0)0.0%	0(0)0.0%	0(0)0.0%	0(0)0.0%	0(0)0.0%
**EYE DISORDERS**	1								
Conjunctivitis Allergic	1	0(0)0.0%	0(0)0.0%	0(0)0.0%	1(1)11.1%	0(0)0.0%	0(0)0.0%	0(0)0.0%	0(0)0.0%
**GASTROINTESTINAL DISORDERS**	17								
Abdominal Pain	4	0(0)0.0%	0(0)0.0%	0(0)0.0%	3(2)22.2%	0(0)0.0%	1(1)11.1%	0(0)0.0%	0(0)0.0%
Dental Caries	2	0(0)0.0%	0(0)0.0%	1(1)11.1%	1(1)11.1%	0(0)0.0%	0(0)0.0%	0(0)0.0%	0(0)0.0%
Dental Discomfort	1	0(0)0.0%	0(0)0.0%	0(0)0.0%	1(1)11.1%	0(0)0.0%	0(0)0.0%	0(0)0.0%	0(0)0.0%
Diarrhoea	2	0(0)0.0%	0(0)0.0%	0(0)0.0%	2(2)22.2%	0(0)0.0%	0(0)0.0%	0(0)0.0%	0(0)0.0%
Gastritis	3	1(1)11.1%	0(0)0.0%	1(1)11.1%	1(1)11.1%	0(0)0.0%	0(0)0.0%	0(0)0.0%	0(0)0.0%
Nausea	1	0(0)0.0%	0(0)0.0%	0(0)0.0%	1(1)11.1%	0(0)0.0%	0(0)0.0%	0(0)0.0%	0(0)0.0%
Toothache	2	0(0)0.0%	0(0)0.0%	1(1)11.1%	0(0)0.0%	1(1)11.1%	0(0)0.0%	0(0)0.0%	0(0)0.0%
Vomiting	2	0(0)0.0%	0(0)0.0%	1(1)11.1%	1(1)11.1%	0(0)0.0%	0(0)0.0%	0(0)0.0%	0(0)0.0%
**GENERAL DISORDERS AND ADMINISTRATION SITE CONDITIONS**	10								
Feeling Hot	9	2(2)22.2%	0(0)0.0%	4(3)33.3%	2(1)11.1%	0(0)0.0%	1(1)11.1%	0(0)0.0%	0(0)0.0%
Pyrexia	1	1(1)11.1%	0(0)0.0%	0(0)0.0%	0(0)0.0%	0(0)0.0%	0(0)0.0%	0(0)0.0%	0(0)0.0%
**INFECTIONS AND INFESTATIONS**	100								
Abscess	2	1(1)11.1%	0(0)0.0%	0(0)0.0%	0(0)0.0%	0(0)0.0%	1(1)11.1%	0(0)0.0%	0(0)0.0%
Bacterial Infection	1	1(1)11.1%	0(0)0.0%	0(0)0.0%	0(0)0.0%	0(0)0.0%	0(0)0.0%	0(0)0.0%	0(0)0.0%
Body Tinea	2	1(1)11.1%	0(0)0.0%	0(0)0.0%	0(0)0.0%	0(0)0.0%	1(1)11.1%	0(0)0.0%	0(0)0.0%
Bronchitis	1	0(0)0.0%	0(0)0.0%	1(1)11.1%	0(0)0.0%	0(0)0.0%	0(0)0.0%	0(0)0.0%	0(0)0.0%

		Arm 1<br>5 mg/kg L9LS SC		Arm 2<br>10 mg/kg<br>L9LS SC	Arm 3<br>20 mg/kg L9LS SC		Arm 4<br>Placebo SC		
**Primary System Organ Class<br>MedDRA Preferred Term**	**All Study Arms**	**G1 Mild**	**G2 Moderate**	**G1 Mild**	**G1 Mild**	**G2 Moderate**	**G1 Mild**	**G2 Moderate**	**G3 Severe**
Conjunctivitis	2	1(1)11.1%	0(0)0.0%	0(0)0.0%	1(1)11.1%	0(0)0.0%	0(0)0.0%	0(0)0.0%	0(0)0.0%
Conjunctivitis Bacterial	1	0(0)0.0%	0(0)0.0%	0(0)0.0%	0(0)0.0%	0(0)0.0%	0(0)0.0%	1(1)11.1%	0(0)0.0%
Ear Lobe Infection	1	0(0)0.0%	0(0)0.0%	1(1)11.1%	0(0)0.0%	0(0)0.0%	0(0)0.0%	0(0)0.0%	0(0)0.0%
Gastroenteritis	5	1(1)11.1%	0(0)0.0%	0(0)0.0%	4(3)33.3%	0(0)0.0%	0(0)0.0%	0(0)0.0%	0(0)0.0%
Gingivitis	2	1(1)11.1%	0(0)0.0%	0(0)0.0%	0(0)0.0%	1(1)11.1%	0(0)0.0%	0(0)0.0%	0(0)0.0%
Helminthic Infection	1	0(0)0.0%	0(0)0.0%	0(0)0.0%	0(0)0.0%	0(0)0.0%	1(1)11.1%	0(0)0.0%	0(0)0.0%
Hepatitis A	2	0(0)0.0%	0(0)0.0%	0(0)0.0%	1(1)11.1%	0(0)0.0%	1(1)11.1%	0(0)0.0%	0(0)0.0%
Malaria	15	5(4)44.4%	1(1)11.1%	2(2)22.2%	2(2)22.2%	0(0)0.0%	4(3)33.3%	1(1)11.1%	0(0)0.0%
Oral Pustule	1	1(1)11.1%	0(0)0.0%	0(0)0.0%	0(0)0.0%	0(0)0.0%	0(0)0.0%	0(0)0.0%	0(0)0.0%
Otitis Media	2	0(0)0.0%	0(0)0.0%	0(0)0.0%	0(0)0.0%	1(1)11.1%	0(0)0.0%	1(1)11.1%	0(0)0.0%
Otitis Media Acute	1	0(0)0.0%	0(0)0.0%	0(0)0.0%	1(1)11.1%	0(0)0.0%	0(0)0.0%	0(0)0.0%	0(0)0.0%
Pneumonia	3	0(0)0.0%	0(0)0.0%	0(0)0.0%	0(0)0.0%	3(2)22.2%	0(0)0.0%	0(0)0.0%	0(0)0.0%
Rash Pustular	1	0(0)0.0%	0(0)0.0%	1(1)11.1%	0(0)0.0%	0(0)0.0%	0(0)0.0%	0(0)0.0%	0(0)0.0%
Rhinitis	3	0(0)0.0%	0(0)0.0%	2(2)22.2%	1(1)11.1%	0(0)0.0%	0(0)0.0%	0(0)0.0%	0(0)0.0%
Septic Rash	1	0(0)0.0%	0(0)0.0%	0(0)0.0%	0(0)0.0%	0(0)0.0%	1(1)11.1%	0(0)0.0%	0(0)0.0%
Tinea Capitis	7	1(1)11.1%	0(0)0.0%	1(1)11.1%	2(2)22.2%	1(1)11.1%	2(2)22.2%	0(0)0.0%	0(0)0.0%
Tonsillitis	7	0(0)0.0%	0(0)0.0%	1(1)11.1%	2(1)11.1%	2(2)22.2%	1(1)11.1%	1(1)11.1%	0(0)0.0%
Upper Respiratory Tract Infection	36	9(5)55.6%	2(2)22.2%	11(8)88.9%	9(7)77.8%	0(0)0.0%	5(4)44.4%	0(0)0.0%	0(0)0.0%
Upper Respiratory Tract Infection Bacterial	1	0(0)0.0%	0(0)0.0%	0(0)0.0%	0(0)0.0%	0(0)0.0%	0(0)0.0%	1(1)11.1%	0(0)0.0%
Urinary Tract Infection	1	0(0)0.0%	1(1)11.1%	0(0)0.0%	0(0)0.0%	0(0)0.0%	0(0)0.0%	0(0)0.0%	0(0)0.0%
Viral Rash	1	0(0)0.0%	0(0)0.0%	0(0)0.0%	0(0)0.0%	0(0)0.0%	1(1)11.1%	0(0)0.0%	0(0)0.0%
**INJURY, POISONING AND PROCEDURAL COMPLICATIONS**	4								
Radius Fracture	1	0(0)0.0%	0(0)0.0%	0(0)0.0%	0(0)0.0%	0(0)0.0%	0(0)0.0%	0(0)0.0%	1(1)11.1%
Soft Tissue Injury	1	1(1)11.1%	0(0)0.0%	0(0)0.0%	0(0)0.0%	0(0)0.0%	0(0)0.0%	0(0)0.0%	0(0)0.0%

		Arm 1<br>5 mg/kg L9LS SC		Arm 2<br>10 mg/kg<br>L9LS SC	Arm 3<br>20 mg/kg L9LS SC		Arm 4<br>Placebo SC		
**Primary System Organ Class<br>MedDRA Preferred Term**	**All Study Arms**	**G1 Mild**	**G2 Moderate**	**G1 Mild**	**G1 Mild**	**G2 Moderate**	**G1 Mild**	**G2 Moderate**	**G3 Severe**
Wound	2	1(1)11.1%	0(0)0.0%	0(0)0.0%	1(1)11.1%	0(0)0.0%	0(0)0.0%	0(0)0.0%	0(0)0.0%
**NERVOUS SYSTEM DISORDERS**	4								
Headache	4	0(0)0.0%	0(0)0.0%	1(1)11.1%	2(2)22.2%	0(0)0.0%	1(1)11.1%	0(0)0.0%	0(0)0.0%
**RESPIRATORY, THORACIC AND MEDIASTINAL DISORDERS**	2								
Bronchospasm	2	0(0)0.0%	1(1)11.1%	0(0)0.0%	0(0)0.0%	1(1)11.1%	0(0)0.0%	0(0)0.0%	0(0)0.0%
**SKIN AND SUBCUTANEOUS TISSUE DISORDERS**	7								
Rash	1	1(1)11.1%	0(0)0.0%	0(0)0.0%	0(0)0.0%	0(0)0.0%	0(0)0.0%	0(0)0.0%	0(0)0.0%
Rash Papular	4	1(1)11.1%	0(0)0.0%	0(0)0.0%	2(1)11.1%	0(0)0.0%	1(1)11.1%	0(0)0.0%	0(0)0.0%
Rash Pruritic	2	1(1)11.1%	0(0)0.0%	1(1)11.1%	0(0)0.0%	0(0)0.0%	0(0)0.0%	0(0)0.0%	0(0)0.0%

(1) Treatment: Arm 1: 5 mg/kg of Study Drug L9LS SC; Arm 2: 10 mg/kg of Study Drug L9LS SC; Arm 3: 20 mg/kg of Study Drug L9LS SC; Arm 4: Placebo SC.
(2) MedDRA: Medical Dictionary for Regulatory Activities. Reported terms were coded using MedDRA version 24.1.
(3) Severity: Blank; Grade1=Mild; Grade2=Moderate; Grade3=Severe; Grade4=Potentially Life Threatening; Grade5=Death.
(4) N=Total Number of Participants who have received the Study Drug L9LS Product or Placebo. Each Field has a format # (X) %. Where # is the Number of Adverse Events. “X” is the Number of Participants with one or more episodes of the given event. % is the Number of Participants with one or more episodes of the given event divided by the Total Number of Participants in the Arm.

Table S 6: Listing of serious adverse events (SAEs)

No.	Diagnosis (primary SAE)	Study arm	Date of first IP dose	Date of last IP dose	Days after IP dose	Date of onset	Date of full recovery
**1**	EDTA dependent Pseudothrombocytopenia	1-dose L9LS	7-Feb-23	7-Feb-23	7	14-Feb-23	17-Feb-23
**2**	Pneumonia	1-dose L9LS	2-Mar-23	2-Mar-23	6	8-Mar-23	16-Mar-23
**3**	Severe malaria with convulsions and prostration	Placebo	2-Feb-23	2-Feb-23	66	9-Apr-23	13-Apr-23
**4**	Salmonella typhi Gastroenteritis	Placebo	9-Feb-23	9-Feb-23	75	25-Apr-23	3-May-23
**5**	Febrile illness unknown cause	2-dose L9LS	11-Apr-23	11-Apr-23	21	2-May-23	8-May-23
**6**	Severe malaria with prostration	Placebo	6-Mar-23	6-Mar-23	70	15-May-23	21-May-23
**7**	Prolonged febrile illness	2-dose L9LS	5-Apr-23	5-Apr-23	41	16-May-23	23-Jun-23
**8**	Pneumonia	Placebo	26-Apr-23	26-Apr-23	21	17-May-23	24-May-23
**9**	Epilepsy	1-dose L9LS	12-May-23	12-May-23	64	15-Jul-23	Recovering at closeout
**10**	Gastroenteritis with severe dehydration	Placebo	26-Apr-23	26-Apr-23	88	23-Jul-23	29-Jul-23
**11**	Epilepsy	Placebo	18-Apr-23	18-Apr-23	118	14-Aug-23	Recovering at closeout
**12**	Typhoid fever	Placebo	13-Feb-23	24-Jul-23	26	19-Aug-23	13-Sep-23
**13**	Pneumonia	1-dose L9LS	2-Feb-23	13-Jul-23	39	21-Aug-23	3-Sep-23
**14**	Hydrocele	Placebo	6-Feb-23	18-Jul-23	42	29-Aug-23	11-Oct-23
**15**	Severe malaria with prostration and respiratory distress	2-dose L9LS	2-Jun-23	2-Jun-23	111	21-Sep-23	25-Sep-23
**16**	Bronchiolitis	Placebo	31-Jan-23	11-Jul-23	82	1-Oct-23	16-Oct-23
**17**	Partial esophageal obstruction due to foreign body ingestion	Placebo	10-Mar-23	18-Aug-23	48	5-Oct-23	7-Oct-23
**18**	Mumps	Placebo	16-May-23	16-May-23	158	19-Oct-23	25-Oct-23
**19**	Hydrocele bilateral	2-dose L9LS	28-Mar-23	5-Sep-23	74	18-Nov-23	5-Mar-24

**20**	Burns, 2nd degree	Placebo	29-May-23	6-Nov-23	37	13-Dec-23	29-Jan-24
**21**	Severe malaria with convulsions	1-dose L9LS	6-Feb-23	17-Jul-23	153	17-Dec-23	21-Dec-23
**22**	Severe malaria with convulsions	Placebo	3-Mar-23	14-Aug-23	125	17-Dec-23	23-Dec-23
**23**	Severe malaria with prostration	Placebo	2-Feb-23	18-Jul-23	151	17-Dec-23	24-Dec-23
**24**	Malaria	1-dose L9LS	2-Mar-23	16-Aug-23	127	21-Dec-23	26-Dec-23
**25**	Severe malaria	1-dose L9LS	6-Apr-23	14-Sep-23	109	1-Jan-24	22-Jan-24
**26**	Severe malaria	Placebo	15-Feb-23	27-Jul-23	168	11-Jan-24	15-Jan-24
**27**	Burns, 2nd degree	Placebo	4-Jan-24	4-Jan-24	21	25-Jan-24	N/A
**28**	Severe malaria	Placebo	16-May-23	24-Oct-23	135	7-Mar-24	14-Mar-24
**29**	Severe acute malnutrition	Placebo	26-Apr-23	8-Nov-23	137	24-Mar-24	4-Apr-24

Table S 7: Listing of unsolicited adverse events in part 2

		L9LS 1 Dose (N=108)			L9LS 2 Doses (N=106)			Placebo (N=110)		
**Primary System Organ Class MedDRA Preferred Term**	**All Study Arms**	**G1 Mild**	**G2 Moderate**	**G3 Severe**	**G1 Mild**	**G2 Moderate**	**G3 Severe**	**G1 Mild**	**G2 Moderate**	**G3 Severe**
**BLOOD AND LYMPHATIC SYSTEM DISORDERS**	40									
Anaemia	31	13(11)10.2%	0(0)0.0%	1(1)0.9%	10(9)8.5%	0(0)0.0%	0(0)0.0%	6(6)5.5%	0(0)0.0%	1(1)0.9%
Lymphadenitis	1	0(0)0.0%	0(0)0.0%	0(0)0.0%	0(0)0.0%	0(0)0.0%	1(1)0.9%	0(0)0.0%	0(0)0.0%	0(0)0.0%
Lymphadenopathy	1	1(1)0.9%	0(0)0.0%	0(0)0.0%	0(0)0.0%	0(0)0.0%	0(0)0.0%	0(0)0.0%	0(0)0.0%	0(0)0.0%
Neutropenia	3	2(2)1.9%	0(0)0.0%	0(0)0.0%	0(0)0.0%	0(0)0.0%	0(0)0.0%	0(0)0.0%	0(0)0.0%	1(1)0.9%
Thrombocytopenia	4	2(2)1.9%	0(0)0.0%	0(0)0.0%	0(0)0.0%	0(0)0.0%	0(0)0.0%	1(1)0.9%	1(1)0.9%	0(0)0.0%
**CARDIAC DISORDERS**	1									
Palpitations	1	0(0)0.0%	0(0)0.0%	1(1)0.9%	0(0)0.0%	0(0)0.0%	0(0)0.0%	0(0)0.0%	0(0)0.0%	0(0)0.0%
**CONGENITAL, FAMILIAL AND GENETIC DISORDERS**	2									
Hydrocele	2	0(0)0.0%	0(0)0.0%	0(0)0.0%	0(0)0.0%	0(0)0.0%	1(1)0.9%	0(0)0.0%	0(0)0.0%	1(1)0.9%
**EAR AND LABYRINTH DISORDERS**	5									
Cerumen Impaction	5	2(2)1.9%	0(0)0.0%	0(0)0.0%	2(2)1.9%	0(0)0.0%	0(0)0.0%	1(1)0.9%	0(0)0.0%	0(0)0.0%
**EYE DISORDERS**	1									
Conjunctivitis Allergic	1	1(1)0.9%	0(0)0.0%	0(0)0.0%	0(0)0.0%	0(0)0.0%	0(0)0.0%	0(0)0.0%	0(0)0.0%	0(0)0.0%
**GASTROINTESTINAL DISORDERS**	21									
Abdominal Discomfort	1	0(0)0.0%	0(0)0.0%	0(0)0.0%	1(1)0.9%	0(0)0.0%	0(0)0.0%	0(0)0.0%	0(0)0.0%	0(0)0.0%
Abdominal Pain	2	2(2)1.9%	0(0)0.0%	0(0)0.0%	0(0)0.0%	0(0)0.0%	0(0)0.0%	0(0)0.0%	0(0)0.0%	0(0)0.0%
Aphthous Ulcer	2	1(1)0.9%	0(0)0.0%	0(0)0.0%	1(1)0.9%	0(0)0.0%	0(0)0.0%	0(0)0.0%	0(0)0.0%	0(0)0.0%
Enteritis	1	0(0)0.0%	0(0)0.0%	0(0)0.0%	0(0)0.0%	0(0)0.0%	0(0)0.0%	1(1)0.9%	0(0)0.0%	0(0)0.0%
Faeces Hard	1	0(0)0.0%	0(0)0.0%	0(0)0.0%	0(0)0.0%	0(0)0.0%	0(0)0.0%	1(1)0.9%	0(0)0.0%	0(0)0.0%
Haematochezia	1	0(0)0.0%	0(0)0.0%	0(0)0.0%	0(0)0.0%	0(0)0.0%	0(0)0.0%	1(1)0.9%	0(0)0.0%	0(0)0.0%
Lip Swelling	1	0(0)0.0%	0(0)0.0%	0(0)0.0%	0(0)0.0%	0(0)0.0%	0(0)0.0%	1(1)0.9%	0(0)0.0%	0(0)0.0%
Nausea	5	0(0)0.0%	1(1)0.9%	0(0)0.0%	3(3)2.8%	0(0)0.0%	0(0)0.0%	1(1)0.9%	0(0)0.0%	0(0)0.0%
Oesophageal Obstruction	1	0(0)0.0%	0(0)0.0%	0(0)0.0%	0(0)0.0%	0(0)0.0%	0(0)0.0%	0(0)0.0%	0(0)0.0%	1(1)0.9%

Primary System Organ Class<br>MedDRA Preferred Term	All Study Arms	L9LS 1 Dose (N=108)			L9LS 2 Doses (N=106)			Placebo (N=110)		
		G1 Mild	G2 Moderate	G3 Severe	G1 Mild	G2 Moderate	G3 Severe	G1 Mild	G2 Moderate	G3 Severe
Stomatitis	4	0(0)0.0%	0(0)0.0%	0(0)0.0%	0(0)0.0%	0(0)0.0%	0(0)0.0%	4(4)3.6%	0(0)0.0%	0(0)0.0%
Vomiting	2	1(1)0.9%	0(0)0.0%	0(0)0.0%	1(1)0.9%	0(0)0.0%	0(0)0.0%	0(0)0.0%	0(0)0.0%	0(0)0.0%
**GENERAL DISORDERS AND ADMINISTRATION SITE CONDITIONS**	55									
Asthenia	1	0(0)0.0%	0(0)0.0%	0(0)0.0%	0(0)0.0%	0(0)0.0%	0(0)0.0%	1(1)0.9%	0(0)0.0%	0(0)0.0%
Feeling Hot	42	18(16)14.8%	0(0)0.0%	0(0)0.0%	12(11)10.4%	0(0)0.0%	0(0)0.0%	12(12)10.9%	0(0)0.0%	0(0)0.0%
Malaise	1	1(1)0.9%	0(0)0.0%	0(0)0.0%	0(0)0.0%	0(0)0.0%	0(0)0.0%	0(0)0.0%	0(0)0.0%	0(0)0.0%
Pyrexia	11	3(2)1.9%	0(0)0.0%	0(0)0.0%	4(4)3.8%	1(1)0.9%	1(1)0.9%	1(1)0.9%	1(1)0.9%	0(0)0.0%
**INFECTIONS AND INFESTATIONS**	1784									
Abscess	3	2(2)1.9%	0(0)0.0%	0(0)0.0%	0(0)0.0%	0(0)0.0%	0(0)0.0%	1(1)0.9%	0(0)0.0%	0(0)0.0%
Acrodermatitis	9	2(2)1.9%	0(0)0.0%	0(0)0.0%	5(5)4.7%	0(0)0.0%	0(0)0.0%	1(1)0.9%	1(1)0.9%	0(0)0.0%
Bacterial Infection	5	1(1)0.9%	0(0)0.0%	0(0)0.0%	2(2)1.9%	0(0)0.0%	0(0)0.0%	2(2)1.8%	0(0)0.0%	0(0)0.0%
Body Tinea	12	7(7)6.5%	0(0)0.0%	0(0)0.0%	3(3)2.8%	0(0)0.0%	0(0)0.0%	2(2)1.8%	0(0)0.0%	0(0)0.0%
Bronchiolitis	5	0(0)0.0%	0(0)0.0%	0(0)0.0%	2(2)1.9%	1(1)0.9%	1(1)0.9%	0(0)0.0%	0(0)0.0%	1(1)0.9%
Cellulitis	1	0(0)0.0%	0(0)0.0%	0(0)0.0%	0(0)0.0%	0(0)0.0%	0(0)0.0%	1(1)0.9%	0(0)0.0%	0(0)0.0%
Conjunctivitis	25	6(6)5.6%	0(0)0.0%	0(0)0.0%	12(11)10.4%	0(0)0.0%	0(0)0.0%	7(7)6.4%	0(0)0.0%	0(0)0.0%
Ear Infection	1	0(0)0.0%	0(0)0.0%	0(0)0.0%	1(1)0.9%	0(0)0.0%	0(0)0.0%	0(0)0.0%	0(0)0.0%	0(0)0.0%
Febrile Infection	1	0(0)0.0%	0(0)0.0%	0(0)0.0%	0(0)0.0%	0(0)0.0%	1(1)0.9%	0(0)0.0%	0(0)0.0%	0(0)0.0%
Furuncle	4	1(1)0.9%	0(0)0.0%	0(0)0.0%	2(2)1.9%	0(0)0.0%	0(0)0.0%	1(1)0.9%	0(0)0.0%	0(0)0.0%
Gastroenteritis	124	39(31)28.7%	0(0)0.0%	0(0)0.0%	39(35)33.0%	0(0)0.0%	0(0)0.0%	43(33)30.0%	2(2)1.8%	1(1)0.9%
Gastroenteritis Salmonella	2	0(0)0.0%	0(0)0.0%	0(0)0.0%	1(1)0.9%	0(0)0.0%	0(0)0.0%	0(0)0.0%	0(0)0.0%	1(1)0.9%
Helminthic Infection	1	0(0)0.0%	0(0)0.0%	0(0)0.0%	0(0)0.0%	0(0)0.0%	0(0)0.0%	1(1)0.9%	0(0)0.0%	0(0)0.0%
Hepatitis A	5	1(1)0.9%	0(0)0.0%	0(0)0.0%	2(2)1.9%	0(0)0.0%	0(0)0.0%	2(2)1.8%	0(0)0.0%	0(0)0.0%
Hordeolum	1	0(0)0.0%	0(0)0.0%	0(0)0.0%	1(1)0.9%	0(0)0.0%	0(0)0.0%	0(0)0.0%	0(0)0.0%	0(0)0.0%
Impetigo	1	0(0)0.0%	0(0)0.0%	0(0)0.0%	1(1)0.9%	0(0)0.0%	0(0)0.0%	0(0)0.0%	0(0)0.0%	0(0)0.0%
Malaria	1134	310(87)80.6%	83(58)53.7%	3(3)2.8%	256(67)63.2%	49(37)34.9%	1(1)0.9%	356(89)80.9%	70(45)40.9%	6(5)4.5%

		L9LS 1 Dose (N=108)			L9LS 2 Doses (N=106)			Placebo (N=110)		
**Primary System Organ Class<br>MedDRA Preferred Term**	**All Study Arms**	**G1 Mild**	**G2 Moderate**	**G3 Severe**	**G1 Mild**	**G2 Moderate**	**G3 Severe**	**G1 Mild**	**G2 Moderate**	**G3 Severe**
Mumps	4	1(1)0.9%	0(0)0.0%	0(0)0.0%	2(2)1.9%	0(0)0.0%	0(0)0.0%	0(0)0.0%	0(0)0.0%	1(1)0.9%
Oral Candidiasis	2	2(2)1.9%	0(0)0.0%	0(0)0.0%	0(0)0.0%	0(0)0.0%	0(0)0.0%	0(0)0.0%	0(0)0.0%	0(0)0.0%
Oral Herpes	1	0(0)0.0%	0(0)0.0%	0(0)0.0%	0(0)0.0%	0(0)0.0%	0(0)0.0%	1(1)0.9%	0(0)0.0%	0(0)0.0%
Otitis Media	11	6(6)5.6%	0(0)0.0%	0(0)0.0%	1(1)0.9%	1(1)0.9%	0(0)0.0%	3(3)2.7%	0(0)0.0%	0(0)0.0%
Parotitis	2	1(1)0.9%	0(0)0.0%	0(0)0.0%	1(1)0.9%	0(0)0.0%	0(0)0.0%	0(0)0.0%	0(0)0.0%	0(0)0.0%
Pertussis	1	0(0)0.0%	0(0)0.0%	0(0)0.0%	0(0)0.0%	0(0)0.0%	0(0)0.0%	1(1)0.9%	0(0)0.0%	0(0)0.0%
Pharyngitis	1	0(0)0.0%	0(0)0.0%	0(0)0.0%	1(1)0.9%	0(0)0.0%	0(0)0.0%	0(0)0.0%	0(0)0.0%	0(0)0.0%
Pneumonia	32	8(8)7.4%	1(1)0.9%	2(2)1.9%	12(12)11.3%	2(2)1.9%	0(0)0.0%	6(6)5.5%	0(0)0.0%	1(1)0.9%
Rash Pustular	16	6(6)5.6%	0(0)0.0%	0(0)0.0%	5(5)4.7%	0(0)0.0%	0(0)0.0%	5(5)4.5%	0(0)0.0%	0(0)0.0%
Rhinitis	29	10(9)8.3%	0(0)0.0%	0(0)0.0%	10(9)8.5%	0(0)0.0%	0(0)0.0%	9(8)7.3%	0(0)0.0%	0(0)0.0%
Septic Rash	8	1(1)0.9%	0(0)0.0%	0(0)0.0%	7(7)6.6%	0(0)0.0%	0(0)0.0%	0(0)0.0%	0(0)0.0%	0(0)0.0%
Tinea Capitis	14	2(2)1.9%	0(0)0.0%	0(0)0.0%	10(8)7.5%	0(0)0.0%	0(0)0.0%	2(2)1.8%	0(0)0.0%	0(0)0.0%
Tonsillitis	23	7(7)6.5%	3(3)2.8%	0(0)0.0%	5(5)4.7%	0(0)0.0%	0(0)0.0%	8(8)7.3%	0(0)0.0%	0(0)0.0%
Typhoid Fever	1	0(0)0.0%	0(0)0.0%	0(0)0.0%	0(0)0.0%	0(0)0.0%	0(0)0.0%	0(0)0.0%	0(0)0.0%	1(1)0.9%
Upper Respiratory Tract Infection	295	97(71)65.7%	1(1)0.9%	0(0)0.0%	105(71)67.0%	0(0)0.0%	0(0)0.0%	90(69)62.7%	2(2)1.8%	0(0)0.0%
Urinary Tract Infection	2	1(1)0.9%	0(0)0.0%	0(0)0.0%	0(0)0.0%	0(0)0.0%	0(0)0.0%	1(1)0.9%	0(0)0.0%	0(0)0.0%
Varicella	3	1(1)0.9%	0(0)0.0%	0(0)0.0%	1(1)0.9%	1(1)0.9%	0(0)0.0%	0(0)0.0%	0(0)0.0%	0(0)0.0%
Wound Infection	5	0(0)0.0%	0(0)0.0%	0(0)0.0%	4(4)3.8%	0(0)0.0%	0(0)0.0%	1(1)0.9%	0(0)0.0%	0(0)0.0%
**INJURY, POISONING AND PROCEDURAL COMPLICATIONS**	23									
Accidental Exposure To Product	1	0(0)0.0%	0(0)0.0%	0(0)0.0%	0(0)0.0%	0(0)0.0%	0(0)0.0%	1(1)0.9%	0(0)0.0%	0(0)0.0%
Burns Second Degree	1	0(0)0.0%	0(0)0.0%	0(0)0.0%	0(0)0.0%	0(0)0.0%	0(0)0.0%	0(0)0.0%	0(0)0.0%	1(1)0.9%
Contusion	4	0(0)0.0%	0(0)0.0%	0(0)0.0%	4(4)3.8%	0(0)0.0%	0(0)0.0%	0(0)0.0%	0(0)0.0%	0(0)0.0%
Greenstick Fracture	1	0(0)0.0%	0(0)0.0%	0(0)0.0%	1(1)0.9%	0(0)0.0%	0(0)0.0%	0(0)0.0%	0(0)0.0%	0(0)0.0%
Joint Dislocation	1	0(0)0.0%	0(0)0.0%	0(0)0.0%	0(0)0.0%	0(0)0.0%	0(0)0.0%	1(1)0.9%	0(0)0.0%	0(0)0.0%
Limb Injury	1	0(0)0.0%	0(0)0.0%	0(0)0.0%	0(0)0.0%	0(0)0.0%	0(0)0.0%	1(1)0.9%	0(0)0.0%	0(0)0.0%

		L9LS 1 Dose (N=108)			L9LS 2 Doses (N=106)			Placebo (N=110)		
**Primary System Organ Class**<br>**MedDRA Preferred Term**	**All Study Arms**	**G1 Mild**	**G2 Moderate**	**G3 Severe**	**G1 Mild**	**G2 Moderate**	**G3 Severe**	**G1 Mild**	**G2 Moderate**	**G3 Severe**
Skin Laceration	1	0(0)0.0%	0(0)0.0%	0(0)0.0%	0(0)0.0%	0(0)0.0%	0(0)0.0%	1(1)0.9%	0(0)0.0%	0(0)0.0%
Soft Tissue Injury	1	0(0)0.0%	0(0)0.0%	0(0)0.0%	1(1)0.9%	0(0)0.0%	0(0)0.0%	0(0)0.0%	0(0)0.0%	0(0)0.0%
Thermal Burn	5	3(3)2.8%	0(0)0.0%	0(0)0.0%	1(1)0.9%	0(0)0.0%	0(0)0.0%	1(1)0.9%	0(0)0.0%	0(0)0.0%
Wound	7	1(1)0.9%	0(0)0.0%	0(0)0.0%	3(3)2.8%	0(0)0.0%	0(0)0.0%	3(3)2.7%	0(0)0.0%	0(0)0.0%
**INVESTIGATIONS**	3									
False Positive Investigation Result	1	0(0)0.0%	0(0)0.0%	1(1)0.9%	0(0)0.0%	0(0)0.0%	0(0)0.0%	0(0)0.0%	0(0)0.0%	0(0)0.0%
Transaminases Increased	2	1(1)0.9%	0(0)0.0%	0(0)0.0%	0(0)0.0%	0(0)0.0%	0(0)0.0%	1(1)0.9%	0(0)0.0%	0(0)0.0%
**METABOLISM AND NUTRITION DISORDERS**	6									
Decreased Appetite	4	2(2)1.9%	0(0)0.0%	0(0)0.0%	1(1)0.9%	0(0)0.0%	0(0)0.0%	1(1)0.9%	0(0)0.0%	0(0)0.0%
Malnutrition	2	1(1)0.9%	0(0)0.0%	0(0)0.0%	0(0)0.0%	0(0)0.0%	0(0)0.0%	0(0)0.0%	0(0)0.0%	1(1)0.9%
**MUSCULOSKELETAL AND CONNECTIVE TISSUE DISORDERS**	1									
Neck Pain	1	1(1)0.9%	0(0)0.0%	0(0)0.0%	0(0)0.0%	0(0)0.0%	0(0)0.0%	0(0)0.0%	0(0)0.0%	0(0)0.0%
**NERVOUS SYSTEM DISORDERS**	24									
Epilepsy	2	0(0)0.0%	0(0)0.0%	1(1)0.9%	0(0)0.0%	0(0)0.0%	0(0)0.0%	0(0)0.0%	0(0)0.0%	1(1)0.9%
Febrile Convulsion	18	0(0)0.0%	0(0)0.0%	5(3)2.8%	0(0)0.0%	0(0)0.0%	5(4)3.8%	0(0)0.0%	0(0)0.0%	8(8)7.3%
Seizure	2	0(0)0.0%	0(0)0.0%	0(0)0.0%	0(0)0.0%	0(0)0.0%	1(1)0.9%	0(0)0.0%	0(0)0.0%	1(1)0.9%
Tonic Convulsion	2	0(0)0.0%	0(0)0.0%	0(0)0.0%	0(0)0.0%	0(0)0.0%	1(1)0.9%	0(0)0.0%	0(0)0.0%	1(1)0.9%
**RENAL AND URINARY DISORDERS**	2									
Dysuria	2	0(0)0.0%	0(0)0.0%	0(0)0.0%	1(1)0.9%	0(0)0.0%	0(0)0.0%	1(1)0.9%	0(0)0.0%	0(0)0.0%
**REPRODUCTIVE SYSTEM AND BREAST DISORDERS**	3									
Breast Enlargement	1	0(0)0.0%	0(0)0.0%	0(0)0.0%	0(0)0.0%	0(0)0.0%	0(0)0.0%	1(1)0.9%	0(0)0.0%	0(0)0.0%
Genital Pain	1	0(0)0.0%	0(0)0.0%	0(0)0.0%	0(0)0.0%	0(0)0.0%	0(0)0.0%	1(1)0.9%	0(0)0.0%	0(0)0.0%
Pruritus Genital	1	1(1)0.9%	0(0)0.0%	0(0)0.0%	0(0)0.0%	0(0)0.0%	0(0)0.0%	0(0)0.0%	0(0)0.0%	0(0)0.0%
**RESPIRATORY, THORACIC AND MEDIASTINAL DISORDERS**	3									

		L9LS 1 Dose (N=108)			L9LS 2 Doses (N=106)			Placebo (N=110)		
**Primary System Organ Class MedDRA Preferred Term**	**All Study Arms**	**G1 Mild**	**G2 Moderate**	**G3 Severe**	**G1 Mild**	**G2 Moderate**	**G3 Severe**	**G1 Mild**	**G2 Moderate**	**G3 Severe**
Bronchospasm	2	0(0)0.0%	0(0)0.0%	0(0)0.0%	0(0)0.0%	1(1)0.9%	0(0)0.0%	1(1)0.9%	0(0)0.0%	0(0)0.0%
Epistaxis	1	0(0)0.0%	0(0)0.0%	0(0)0.0%	0(0)0.0%	0(0)0.0%	0(0)0.0%	1(1)0.9%	0(0)0.0%	0(0)0.0%
**SKIN AND SUBCUTANEOUS TISSUE DISORDERS**	100									
Dermatitis Allergic	1	1(1)0.9%	0(0)0.0%	0(0)0.0%	0(0)0.0%	0(0)0.0%	0(0)0.0%	0(0)0.0%	0(0)0.0%	0(0)0.0%
Dermatitis Bullous	1	1(1)0.9%	0(0)0.0%	0(0)0.0%	0(0)0.0%	0(0)0.0%	0(0)0.0%	0(0)0.0%	0(0)0.0%	0(0)0.0%
Dermatitis Diaper	3	1(1)0.9%	0(0)0.0%	0(0)0.0%	0(0)0.0%	0(0)0.0%	0(0)0.0%	2(1)0.9%	0(0)0.0%	0(0)0.0%
Dermatitis Exfoliative	1	0(0)0.0%	0(0)0.0%	0(0)0.0%	0(0)0.0%	0(0)0.0%	0(0)0.0%	1(1)0.9%	0(0)0.0%	0(0)0.0%
Eczema	1	0(0)0.0%	0(0)0.0%	0(0)0.0%	1(1)0.9%	0(0)0.0%	0(0)0.0%	0(0)0.0%	0(0)0.0%	0(0)0.0%
Miliaria	2	2(2)1.9%	0(0)0.0%	0(0)0.0%	0(0)0.0%	0(0)0.0%	0(0)0.0%	0(0)0.0%	0(0)0.0%	0(0)0.0%
Rash	2	0(0)0.0%	0(0)0.0%	0(0)0.0%	1(1)0.9%	0(0)0.0%	0(0)0.0%	1(1)0.9%	0(0)0.0%	0(0)0.0%
Rash Macular	1	0(0)0.0%	0(0)0.0%	0(0)0.0%	0(0)0.0%	0(0)0.0%	0(0)0.0%	1(1)0.9%	0(0)0.0%	0(0)0.0%
Rash Maculo-Papular	1	1(1)0.9%	0(0)0.0%	0(0)0.0%	0(0)0.0%	0(0)0.0%	0(0)0.0%	0(0)0.0%	0(0)0.0%	0(0)0.0%
Rash Papular	71	27(24)22.2%	0(0)0.0%	0(0)0.0%	23(20)18.9%	0(0)0.0%	0(0)0.0%	21(19)17.3%	0(0)0.0%	0(0)0.0%
Rash Pruritic	9	6(6)5.6%	0(0)0.0%	0(0)0.0%	2(2)1.9%	0(0)0.0%	0(0)0.0%	1(1)0.9%	0(0)0.0%	0(0)0.0%
Rash Vesicular	6	1(1)0.9%	0(0)0.0%	0(0)0.0%	1(1)0.9%	0(0)0.0%	0(0)0.0%	4(4)3.6%	0(0)0.0%	0(0)0.0%
Urticaria	1	0(0)0.0%	0(0)0.0%	0(0)0.0%	0(0)0.0%	0(0)0.0%	0(0)0.0%	1(1)0.9%	0(0)0.0%	0(0)0.0%

(1) Treatment Arm: Arm 1: 1 Dose of Study Agent L9LS SC; Arm 2: 2 Doses of Study Agent L9LS SC; Arm 3 Placebo SC.
(2) MedDRA: Medical Dictionary for Regulatory Activities. Reported terms were coded using MedDRA version 24.1.
(3) Severity: Blank; Grade1=Mild; Grade2=Moderate; Grade3=Severe; Grade4=Potentially Life Threatening; Grade5=Death.
(4) N=Total Number of Participants who have received the Study Agent L9LS Product or Placebo. Each Field has a format # (X) %. Where # is the Number of Adverse Events. "X" is the Number of Participants with one or more episodes of the given event. % is the Number of Participants with one or more episodes of the given event divided by the Total Number of Participants in the Arm.
(5) We will only record and report unsolicited AEs (excluding malaria) of grade 1 or grade 2 after 28 days of Study Agent 1&2 or Placebo if they are related.
(6) Severe malaria in the site AE description is further described by adding the symptoms defining the severe malaria diagnosis: PROST-prostration; ANEM-severe anemia; RESP-respiratory distress due to acidosis or pulmonary edema; CONV-more than 2 convulsions; CEREB-Cerebral malaria; GLYC-hypoglycemia; JAUN-Jaundice; SHOCK-shock; BLEED-prolonged or spontaneous bleeding; REN-Renal impairment.

Table S 8: Proportion with P· falciparum infection and clinical malaria at 6 and 12 months, by study group (n/N)

	**Placebo**	**1-dose L9LS**	**2-dose L9LS**
6-month blood smear	71/110 (65%)	48/108 (44%)	51/106 (48%)
6-month clinical malaria 2	64/110 (58%)	42/108 (39%)	42/106 (40%)
6-month clinical malaria 1	47/110 (43%)	31/108 (29%)	30/106 (28%)
12-month blood smear	91/110 (83%)	92/108 (85%)	70/106 (66%)
12-month clinical malaria 2	90/110 (82%)	84/108 (78%)	64/106 (60%)
12-month clinical malaria 1	74/110 (67%)	70/108 (65%)	57/106 (54%)

Figure S 2: Kaplan-Meier curves of time to first *P. falciparum* infection as detected by blood smear, by treatment arm and age group

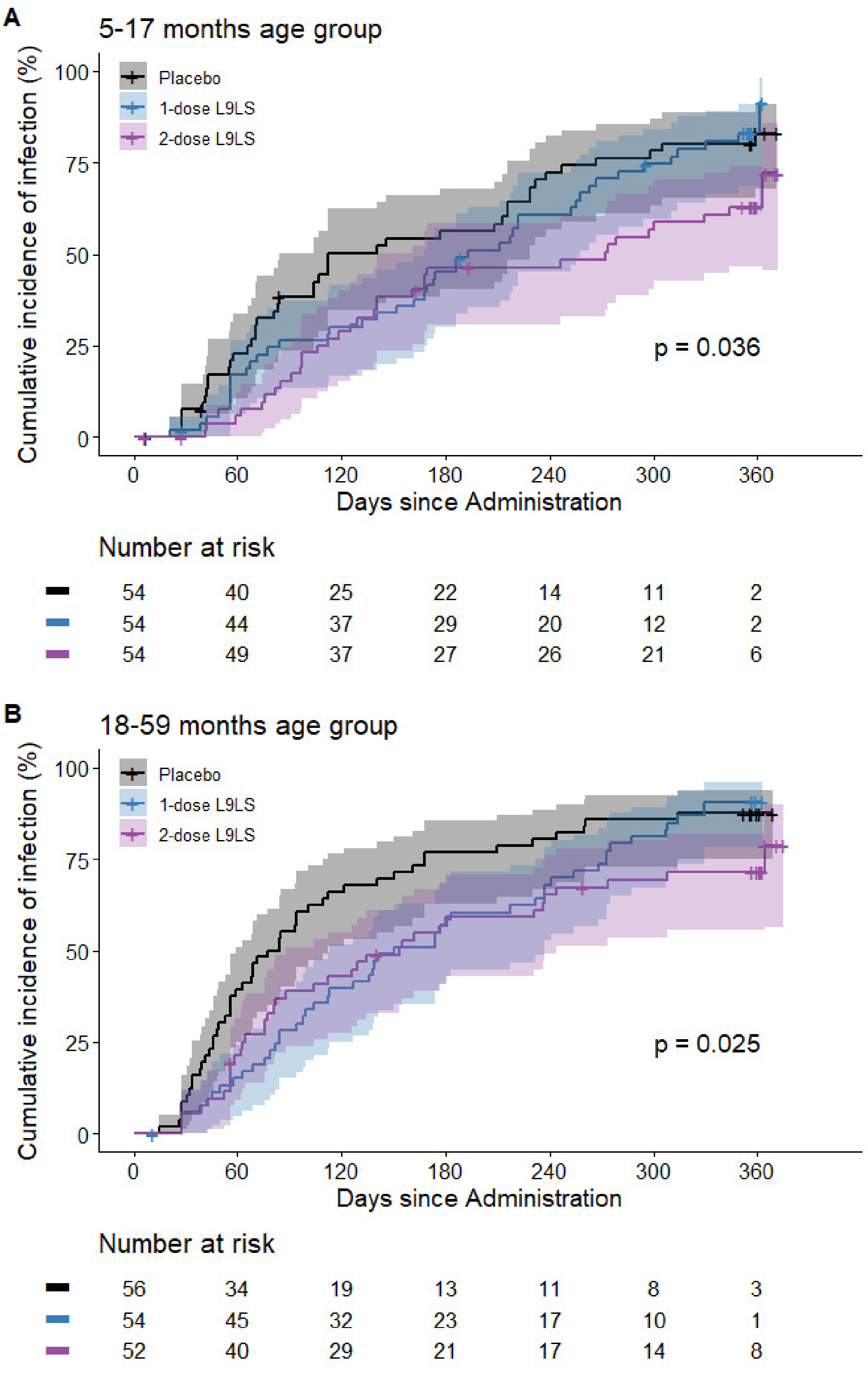

Legend: Surveillance for infection by blood smear and for clinical malaria began 7 days after the first dose of L9LS/placebo and continued through participant close-out. Shading represents 95% confidence intervals. Log-rank tests indicated significant differences: p = 0·036 for 5 – 17 months age group and p = 0.025 for 18 – 59 months age group.

Figure S 3 Protective efficacy of one or two doses of L9LS against P· falciparum infection and clinical malaria at six and 12 months, as compared to placebo, by time-to-event analysis, by age group

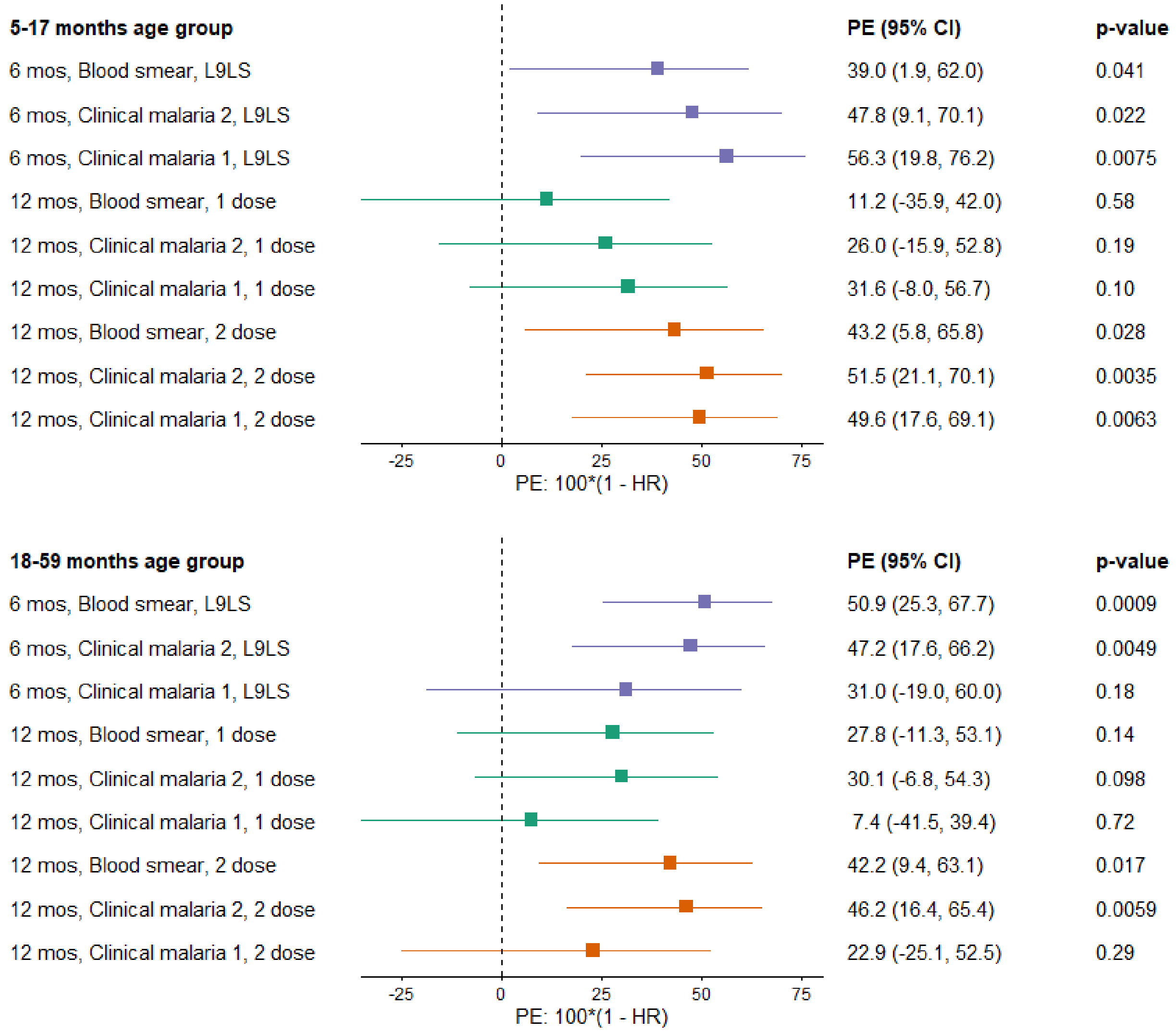

Proportion with P· falciparum infection and clinical malaria at 6 and 12 months, by study and age group (n/N)

	5–17 months			18–59 months		
	Placebo	1-dose L9LS	2-dose L9LS	Placebo	1-dose L9LS	2-dose L9LS
6-month blood smear	28/54 (52%)	21/54 (39%)	23/54 (43%)	43/56 (77%)	27/54 (50%)	28/52 (54%)
6-month clinical malaria 2	26/54 (48%)	16/54 (30%)	18/54 (33%)	38/56 (68%)	26/54 (48%)	24/52 (46%)
6-month clinical malaria 1	22/54 (41%)	11/54 (20%)	13/54 (24%)	25/56 (45%)	20/54 (37%)	17/52 (33%)
12-month blood smear	42/54 (78%)	44/54 (81%)	33/54 (61%)	49/56 (88%)	48/54 (89%)	37/52 (71%)
12-month clinical malaria 2	42/54 (78%)	40/54 (74%)	29/54 (54%)	48/56 (86%)	44/54 (81%)	35/52 (67%)
12-month clinical malaria 1	37/54 (69%)	33/54 (61%)	26/54 (48%)	37/56 (66%)	37/54 (69%)	31/52 (60%)

Figure S 4: Kaplan-Meier curve of time to first clinical malaria, by treatment arm and age group

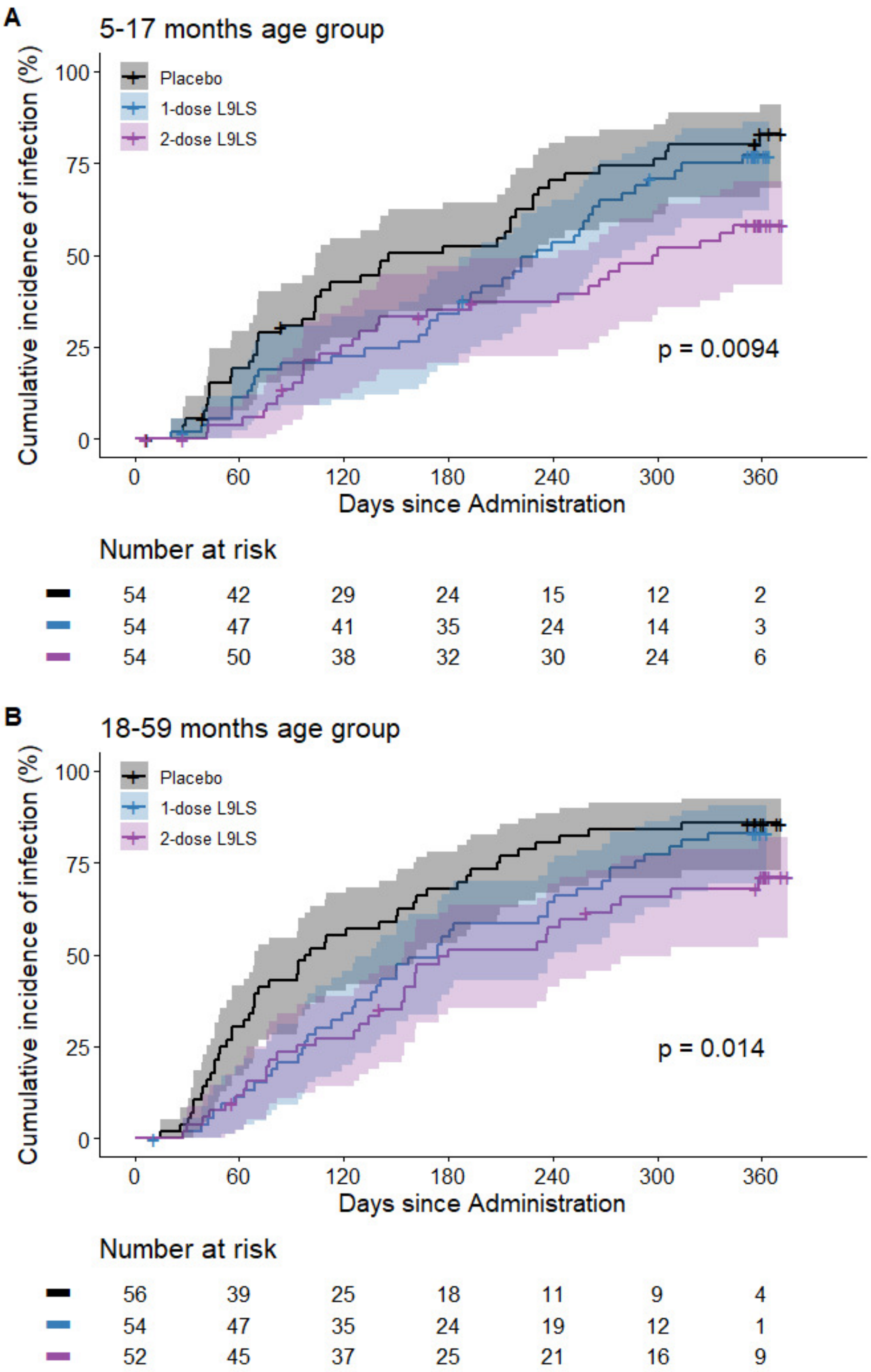

Legend: Clinical malaria defined as blood smear-detected P· falciparum infection plus high measured temperature (≥37·5 ℃) or history of fever in last 24 hours. Surveillance for infection by blood smear and for clinical malaria began 7 days after the first dose of L9LS/placebo and continued through participant close-out. Shading represents 95% confidence intervals. Log-rank tests indicated significant differences for both age groups: p = 0·0094 for 5 – 17 months age group and p = 0.014 for 18 – 59 months age group.

Figure S 5: Protective efficacy of one dose of L9LS against *P. falciparum* infection and clinical malaria at three months, as compared to placebo, by time-to-event analysis, overall and by age group

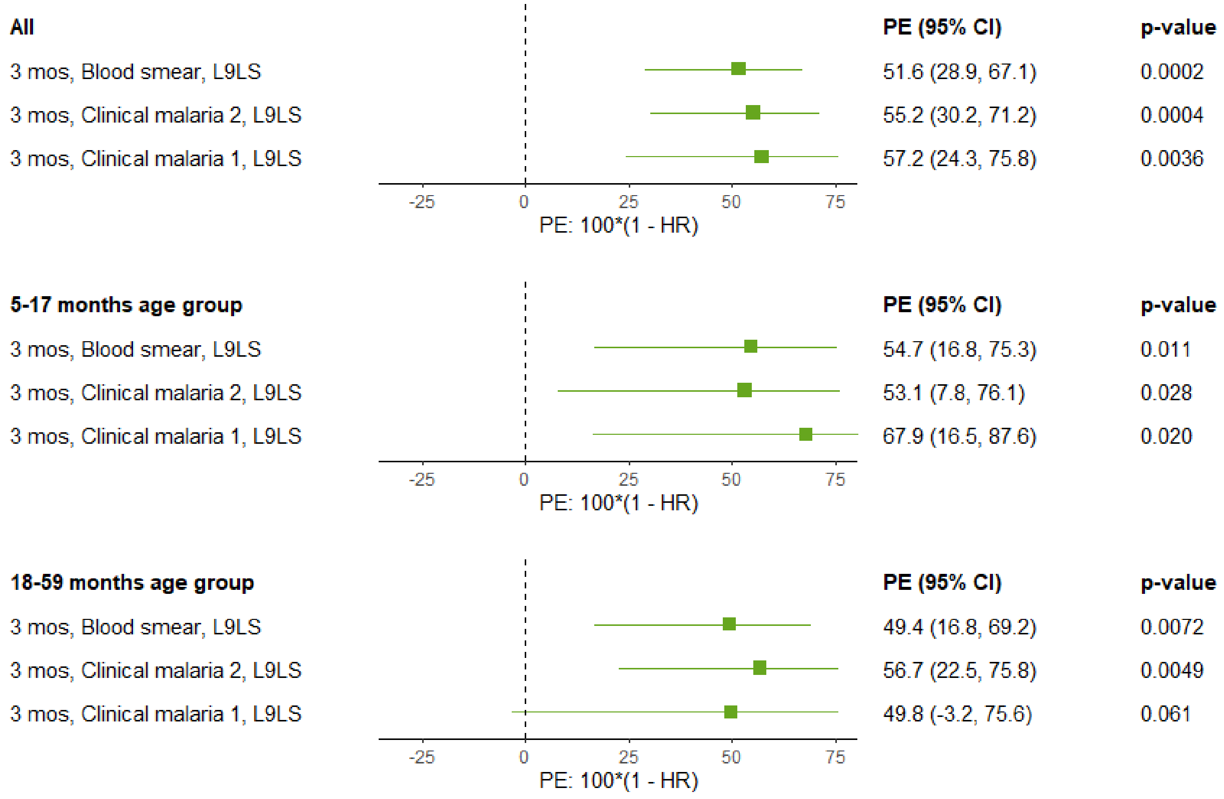

Figure S 6: Protective efficacy of one or two doses of L9LS against *P. falciparum* infection and clinical malaria at six and 12 months, as compared to placebo, by Kaplan-Meier proportional analysis, overall and by age group

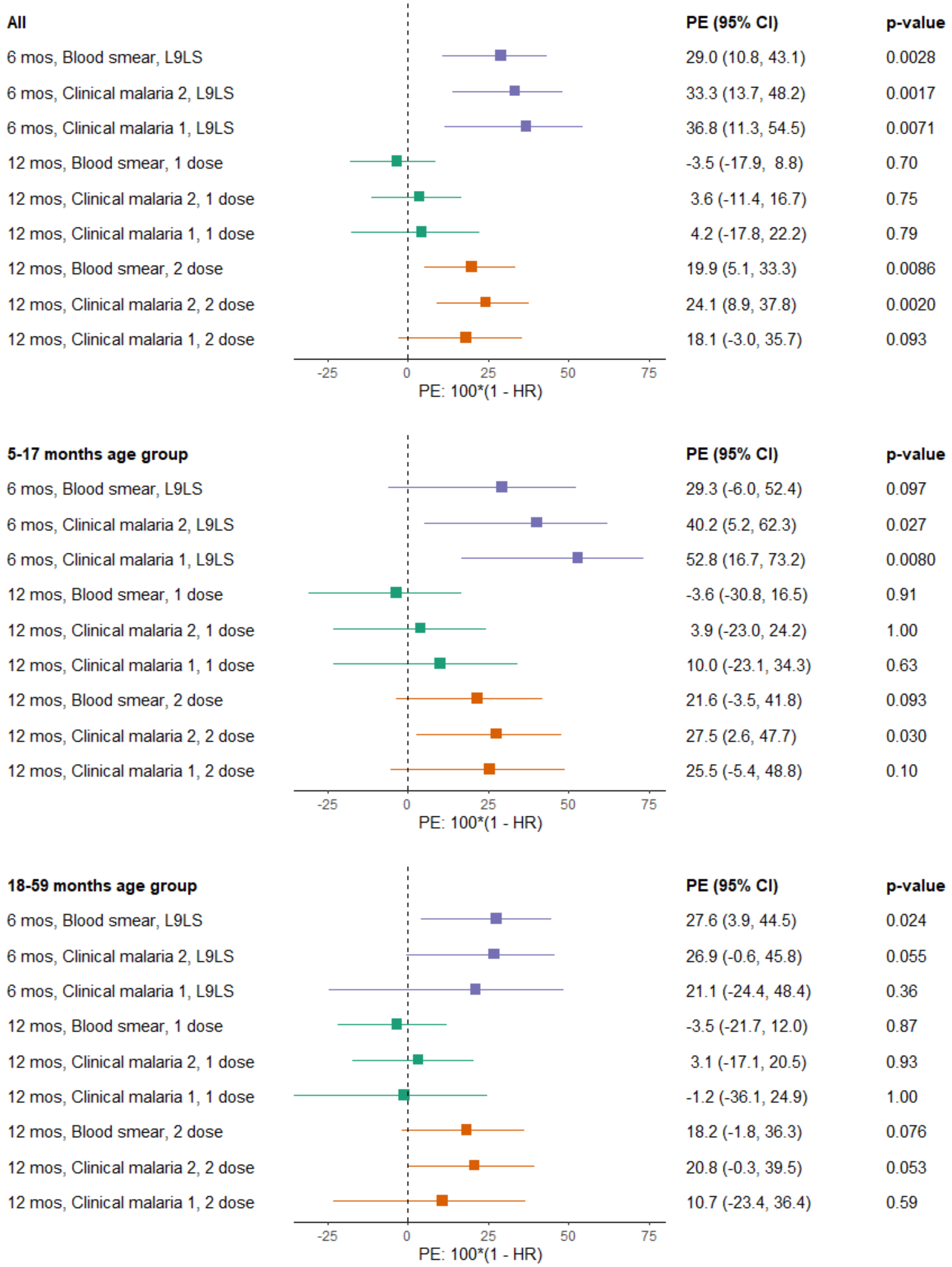

Figure S 7: Protective efficacy of one or two doses of L9LS against *P. falciparum* infection and clinical malaria at six and 12 months, as compared to placebo, by recurrent event analysis, overall and by age group

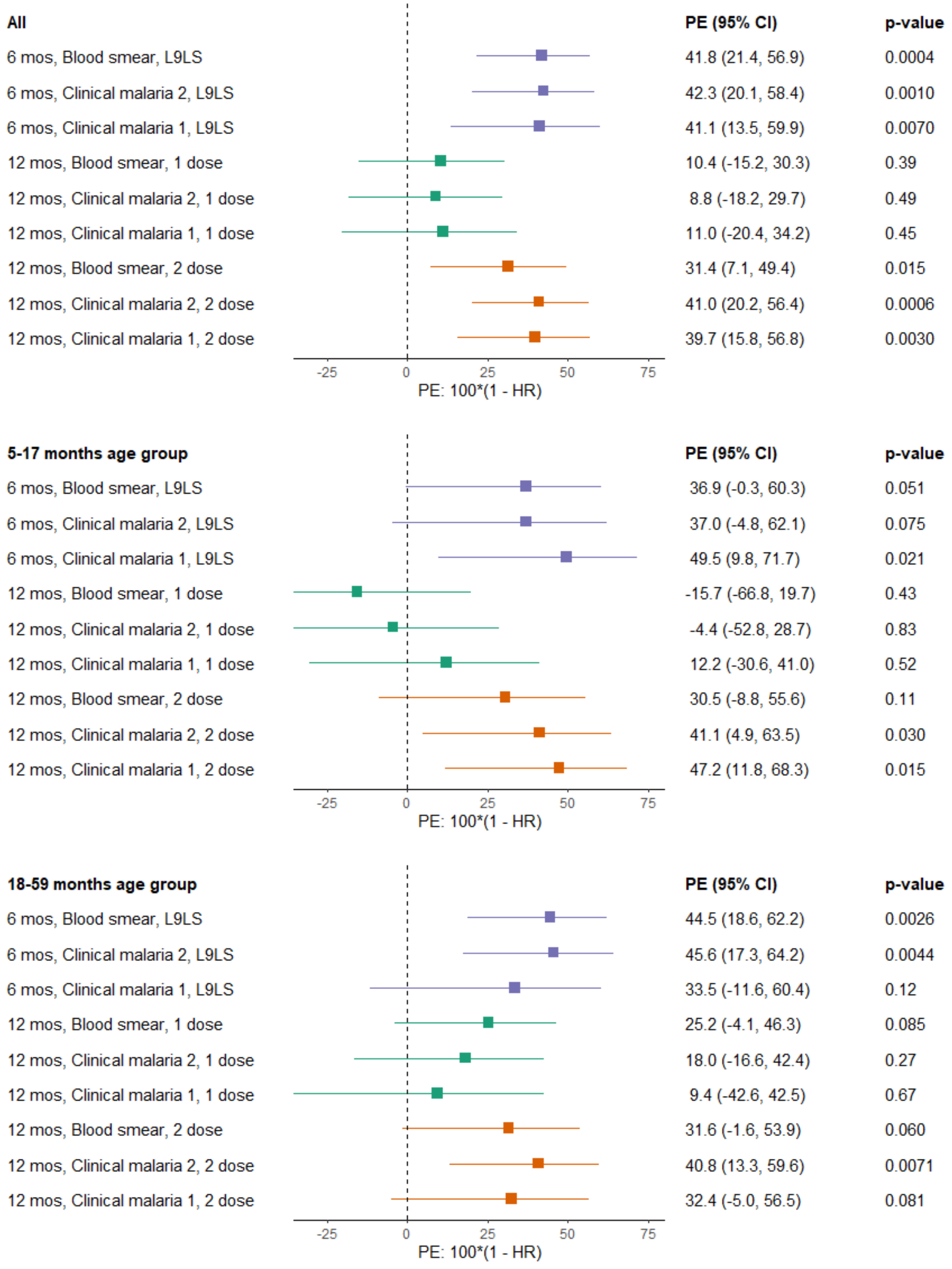

Table S 9: Incidence of Pf infection and clinical malaria (infections/episodes/person-year) by time period and age group

assignment	Overall	5-17 months	18-59 months
Blood smear, 12 months			
Placebo	4.35 (3.90, 4.79)	3.19 (2.66, 3.72)	5.51 (4.81, 6.21)
1-dose L9LS	3.86 (3.45, 4.27)	3.67 (3.10, 4.23)	4.06 (3.46, 4.66)
2-dose L9LS	2.98 (2.61, 3.34)	2.23 (1.79, 2.67)	3.79 (3.20, 4.39)
Blood smear, first 6 months			
Placebo	3.81 (3.22, 4.41)	2.68 (1.98, 3.38)	5.00 (4.03, 5.97)
1-dose L9LS	2.08 (1.66, 2.51)	1.78 (1.23, 2.34)	2.39 (1.74, 3.03)
2-dose L9LS	2.40 (1.93, 2.86)	1.62 (1.09, 2.15)	3.25 (2.46, 4.03)
Blood smear, last 6 months			
Placebo	4.84 (4.20, 5.49)	3.69 (2.88, 4.49)	5.98 (4.96, 6.99)
1-dose L9LS	5.67 (4.97, 6.38)	5.61 (4.62, 6.61)	5.73 (4.73, 6.74)
2-dose L9LS	3.55 (2.99, 4.11)	2.83 (2.14, 3.53)	4.32 (3.43, 5.22)
Clinical malaria 2, 12 months			
Placebo	3.20 (2.83, 3.58)	2.65 (2.17, 3.14)	3.76 (3.18, 4.33)
1-dose L9LS	2.90 (2.54, 3.25)	2.73 (2.24, 3.22)	3.06 (2.55, 3.58)
2-dose L9LS	1.89 (1.60, 2.18)	1.58 (1.21, 1.95)	2.23 (1.77, 2.68)
Clinical malaria 2, first 6 months			
Placebo	2.67 (2.18, 3.17)	1.97 (1.37, 2.56)	3.40 (2.61, 4.20)
1-dose L9LS	1.51 (1.15, 1.87)	1.20 (0.75, 1.65)	1.83 (1.26, 2.40)
2-dose L9LS	1.62 (1.24, 2.00)	1.30 (0.83, 1.78)	1.96 (1.35, 2.57)
Clinical malaria 2, last 6 months			
Placebo	3.70 (3.14, 4.27)	3.32 (2.56, 4.08)	4.08 (3.24, 4.91)
1-dose L9LS	4.30 (3.69, 4.92)	4.31 (3.44, 5.18)	4.30 (3.43, 5.16)
2-dose L9LS	2.16 (1.72, 2.59)	1.85 (1.29, 2.41)	2.49 (1.81, 3.16)
Clinical malaria 1, 12 months			
Placebo	1.79 (1.51, 2.07)	1.75 (1.36, 2.15)	1.83 (1.43, 2.23)
1-dose L9LS	1.58 (1.32, 1.84)	1.51 (1.15, 1.87)	1.65 (1.27, 2.03)
2-dose L9LS	1.08 (0.86, 1.30)	0.94 (0.65, 1.22)	1.23 (0.90, 1.57)
Clinical malaria 1, first 6 months			
Placebo	1.48 (1.11, 1.85)	1.36 (0.86, 1.85)	1.60 (1.06, 2.15)
1-dose L9LS	0.83 (0.57, 1.10)	0.62 (0.30, 0.95)	1.05 (0.62, 1.48)
2-dose L9LS	0.94 (0.65, 1.23)	0.76 (0.40, 1.13)	1.13 (0.67, 1.59)
Clinical malaria 1, last 6 months			
Placebo	2.09 (1.66, 2.51)	2.14 (1.53, 2.75)	2.04 (1.45, 2.63)
1-dose L9LS	2.33 (1.88, 2.79)	2.43 (1.77, 3.08)	2.24 (1.61, 2.87)
2-dose L9LS	1.22 (0.89, 1.54)	1.10 (0.67, 1.54)	1.34 (0.84, 1.84)

Figure S 8: Protective efficacy of one or two doses of L9LS against *P. falciparum* infection and clinical malaria at six and 12 months, as compared to placebo, by time-to-event analysis and censoring protected periods, overall and by age group

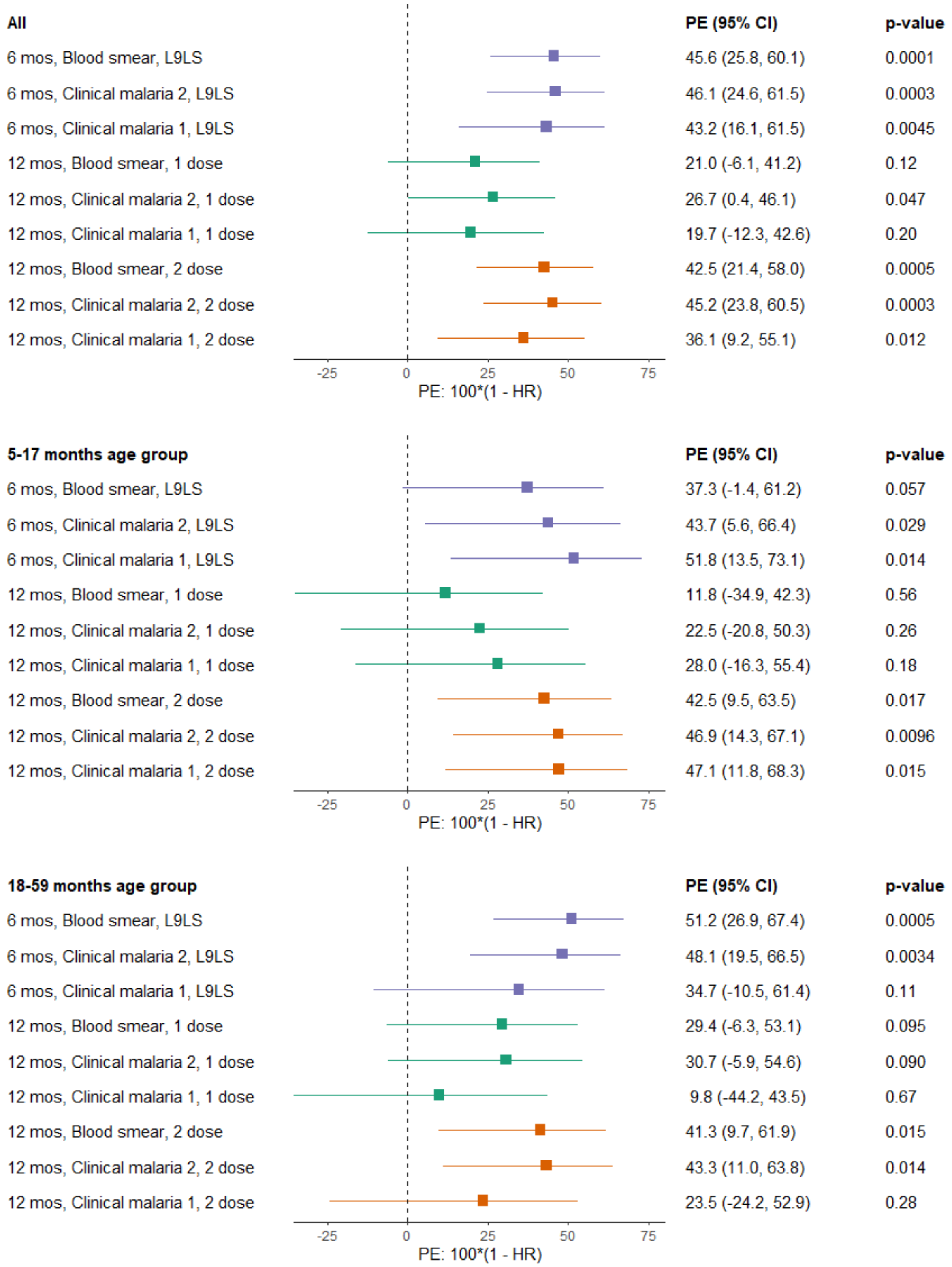

Figure S 9: Protective efficacy of L9LS against *P. falciparum* infection and clinical malaria at six and 12 months, as compared to placebo, by time-to-event analysis, overall and by age group, using per-protocol population

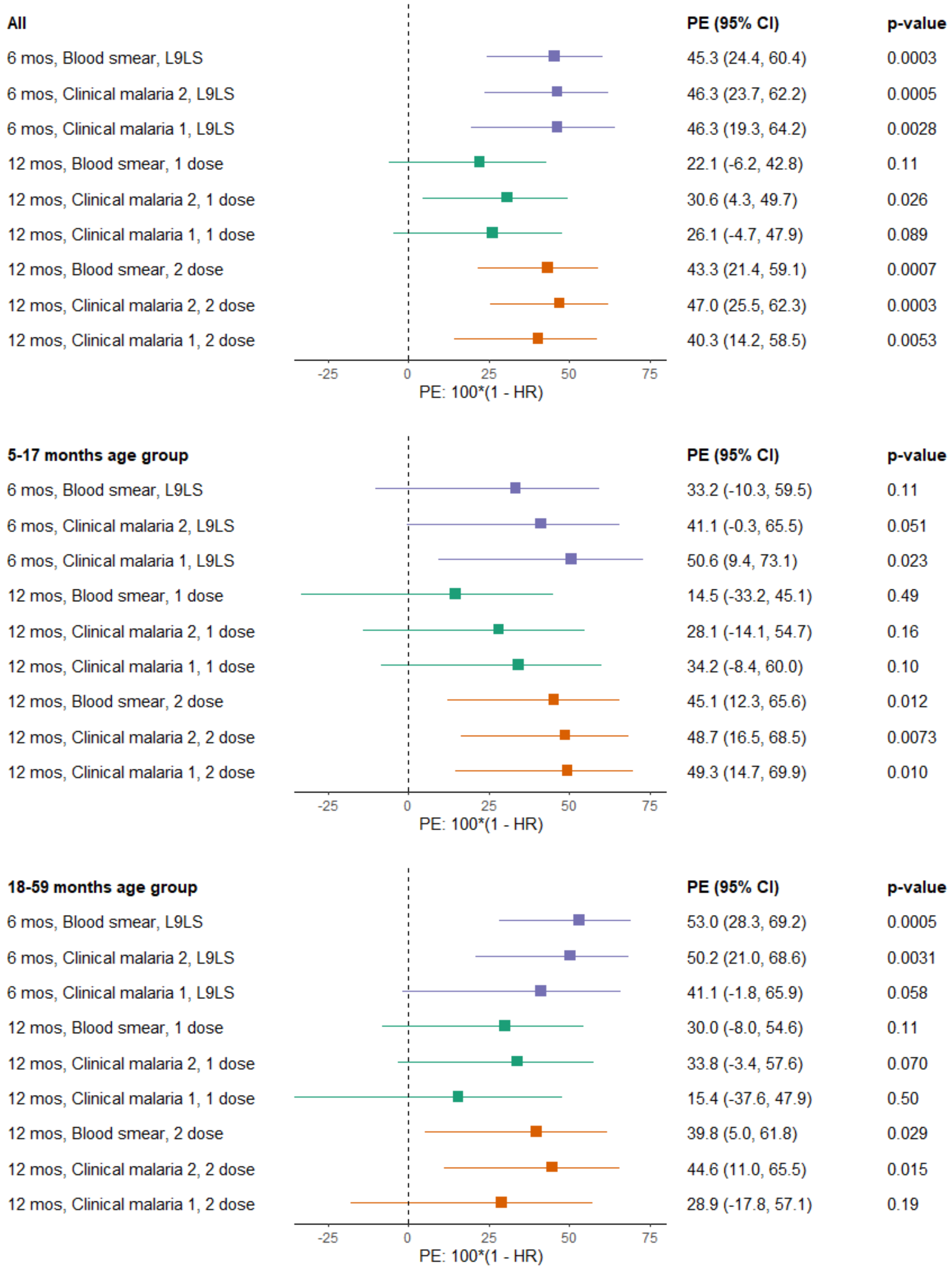

Figure S 10: Protective efficacy of L9LS against *P. falciparum* infection and clinical malaria at six and 12 months, as compared to placebo, by recurrent event analysis, overall and by age group, using per-protocol population

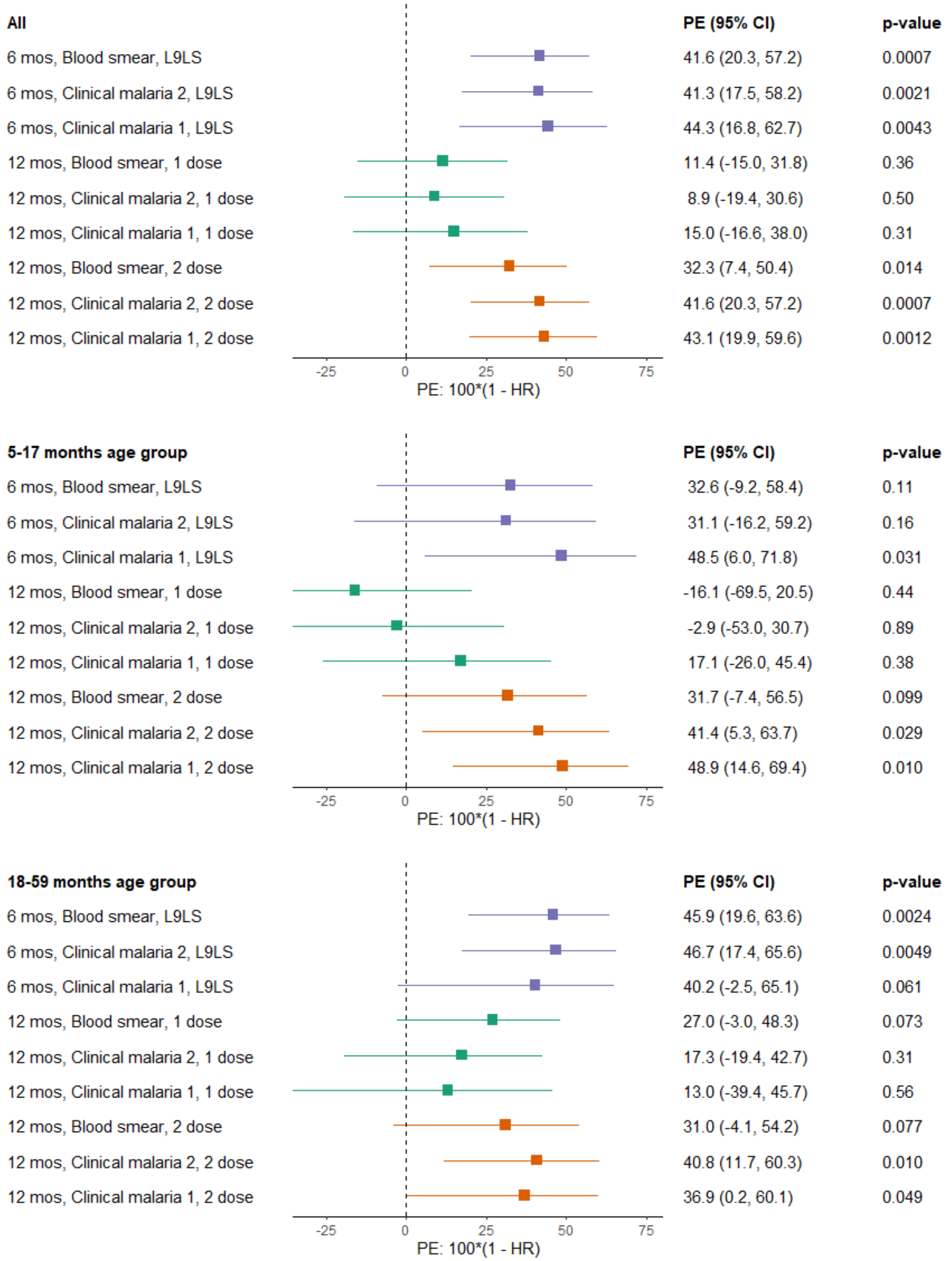

Figure S 11: Kaplan-Meier curves of *P. falciparum* infection risk, by blood smear status and by PCR status prior to antimalarial administration, all arms combined

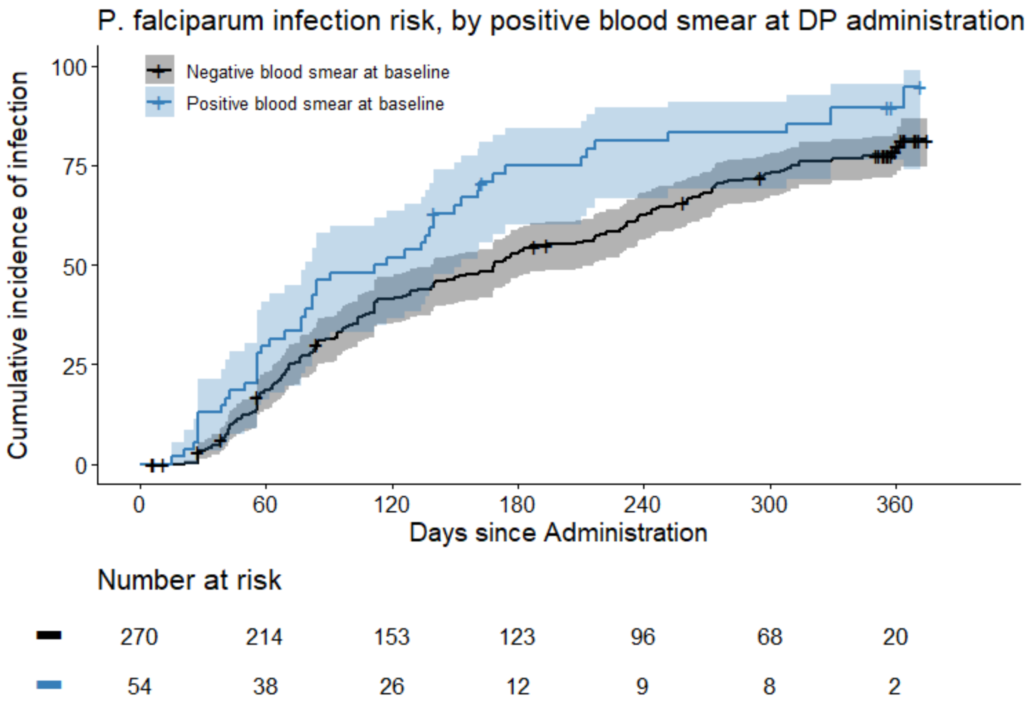

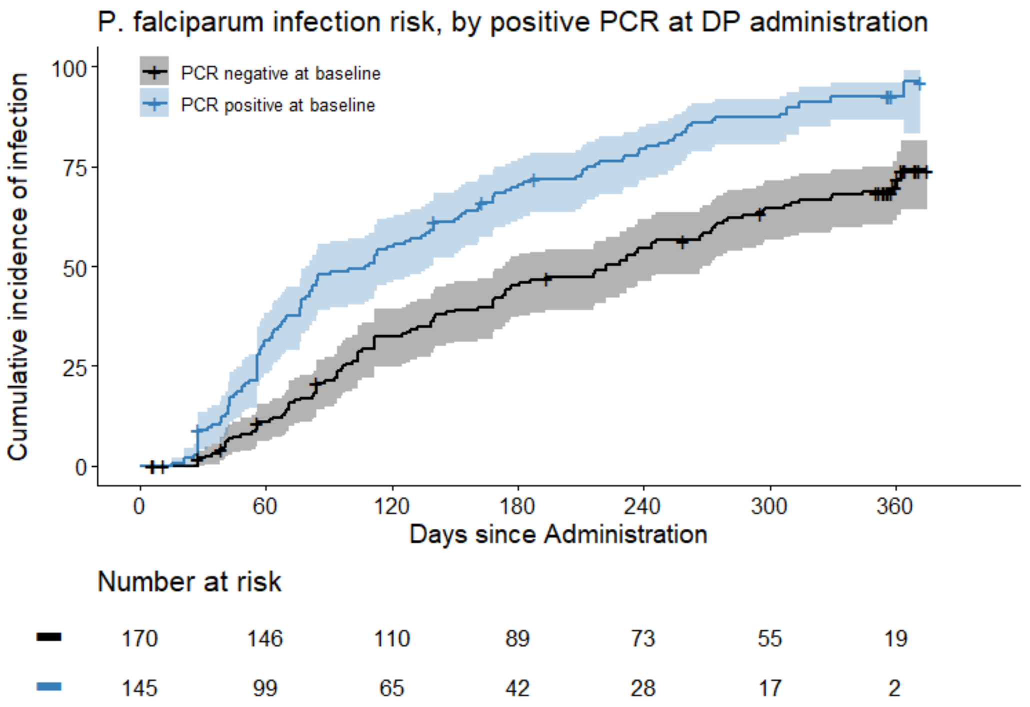

Figure S 12: Kaplan-Meier curve of P. falciparum infection risk, by PCR status at baseline, by study arm

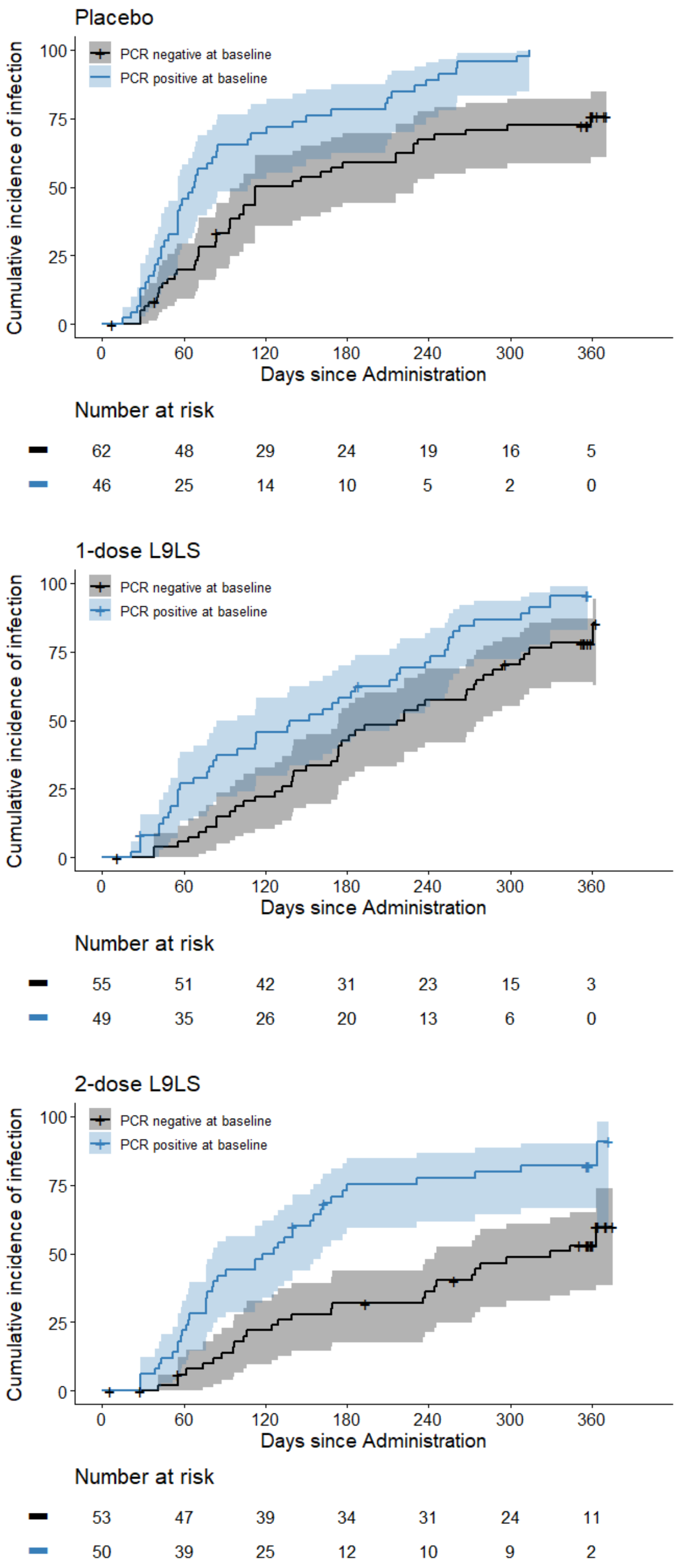

Figure S 13: Kaplan-Meier curve of P. falciparum infection risk among those positive by PCR at baseline, by age and dose group

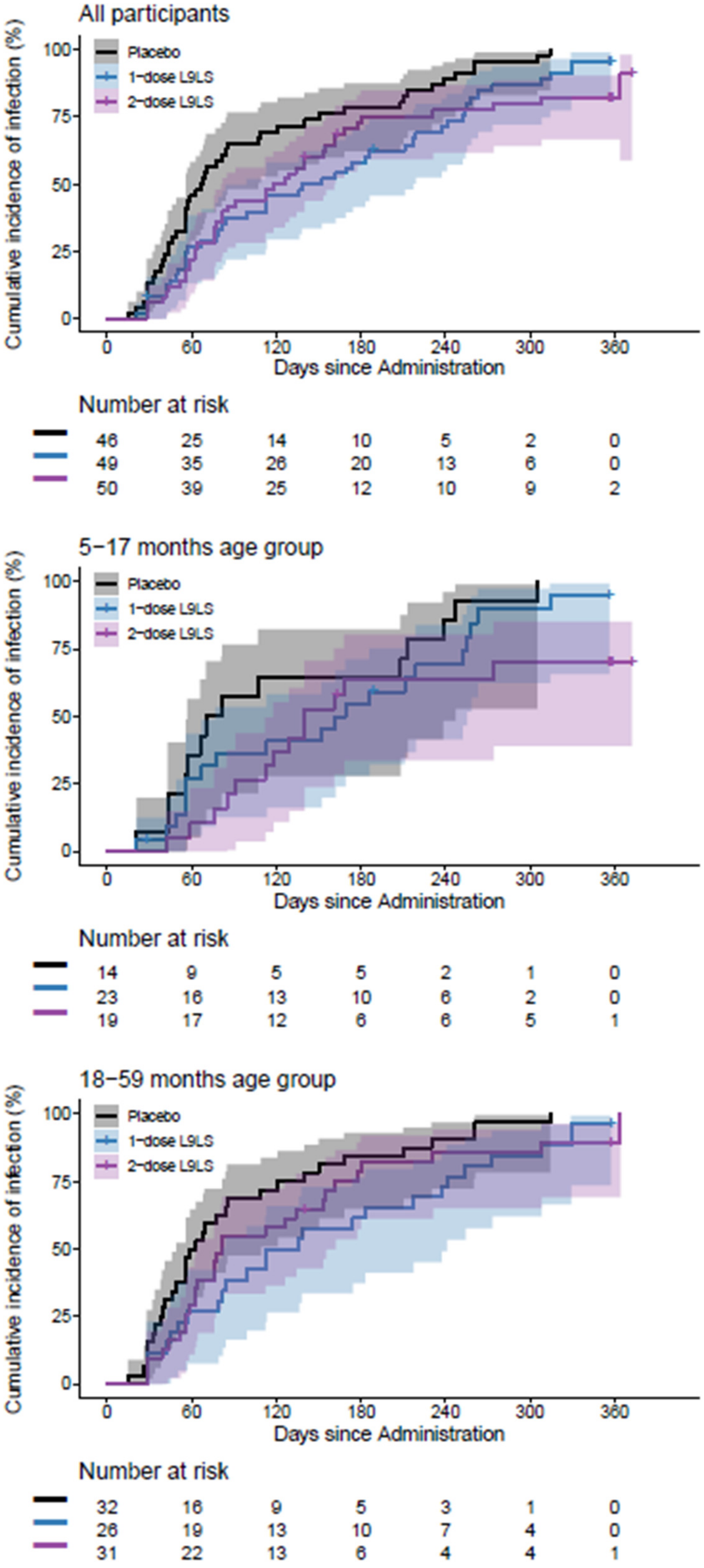

Figure S 14: Kaplan-Meier curve of P. falciparum infection risk among those negative by PCR at baseline, by age and dose group

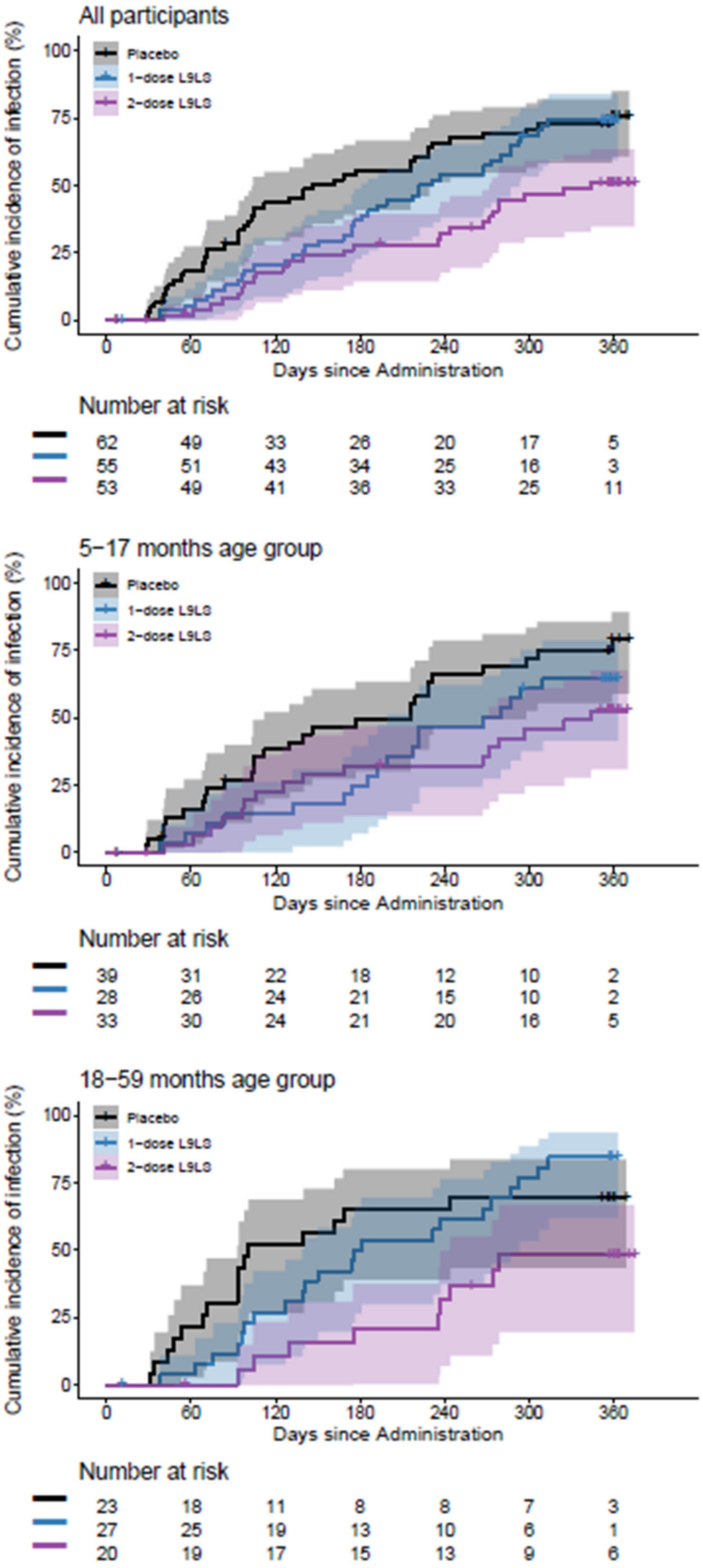

Figure S 15: Protective efficacy of L9LS against *P. falciparum* infection and clinical malaria at six and 12 months, as compared to placebo, by time-to-event analysis, by PCR status at baseline

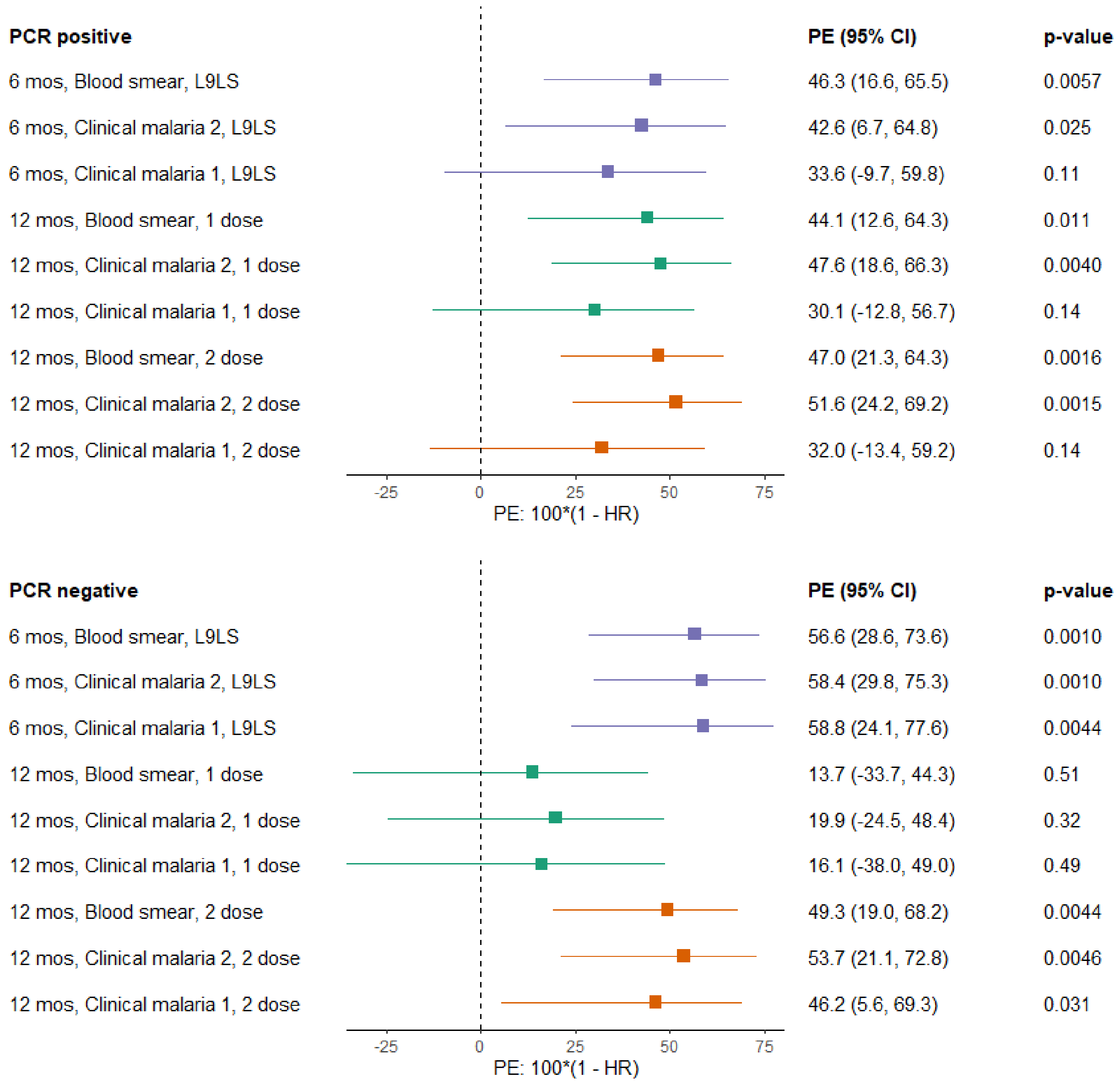

Figure S 16 Protective efficacy of one or two doses of L9LS, using time-to-first event analysis with interval censoring and controlling for month of enrollment

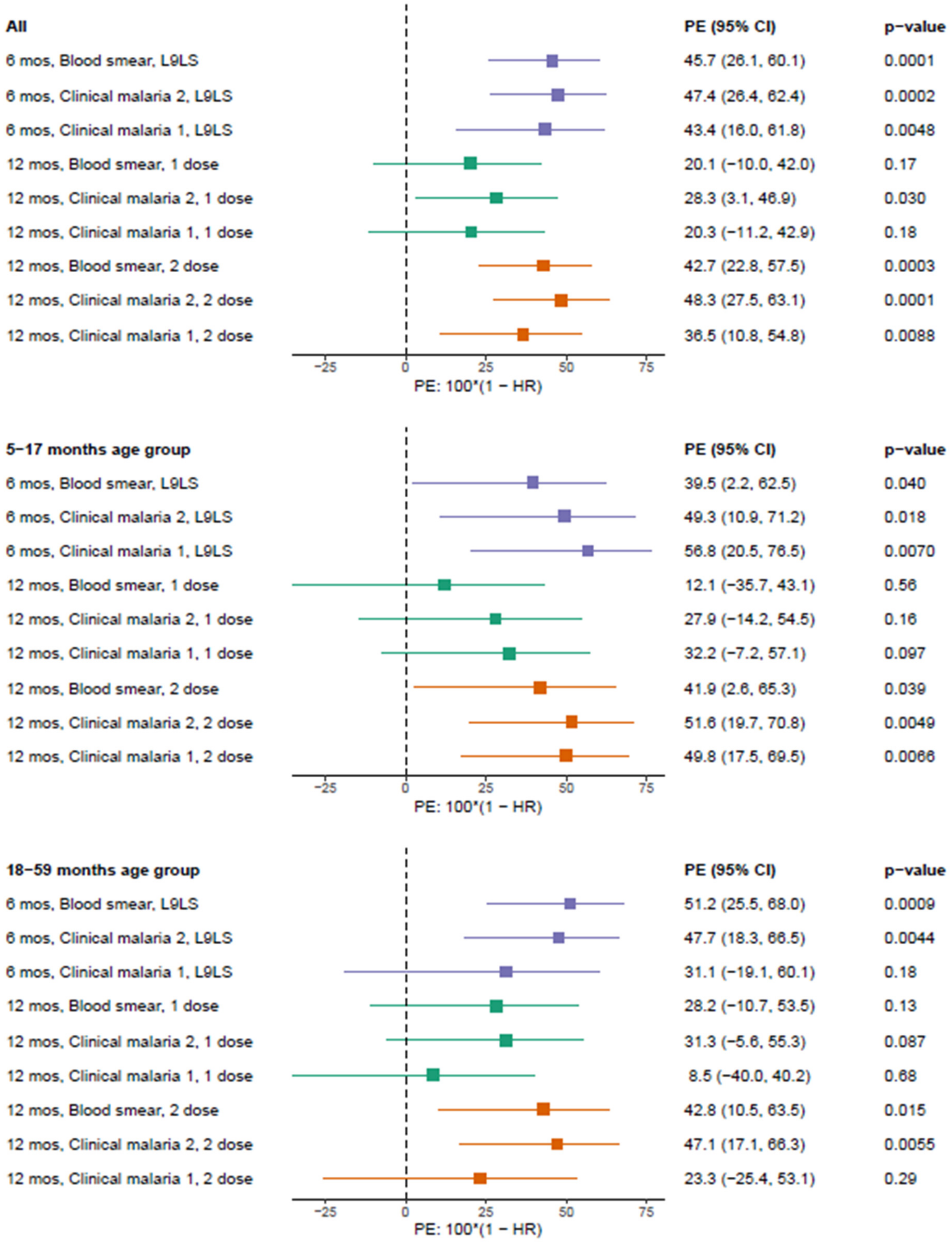

Figure S 17: Mean L9LS serum concentrations versus time, by dose and age groups·

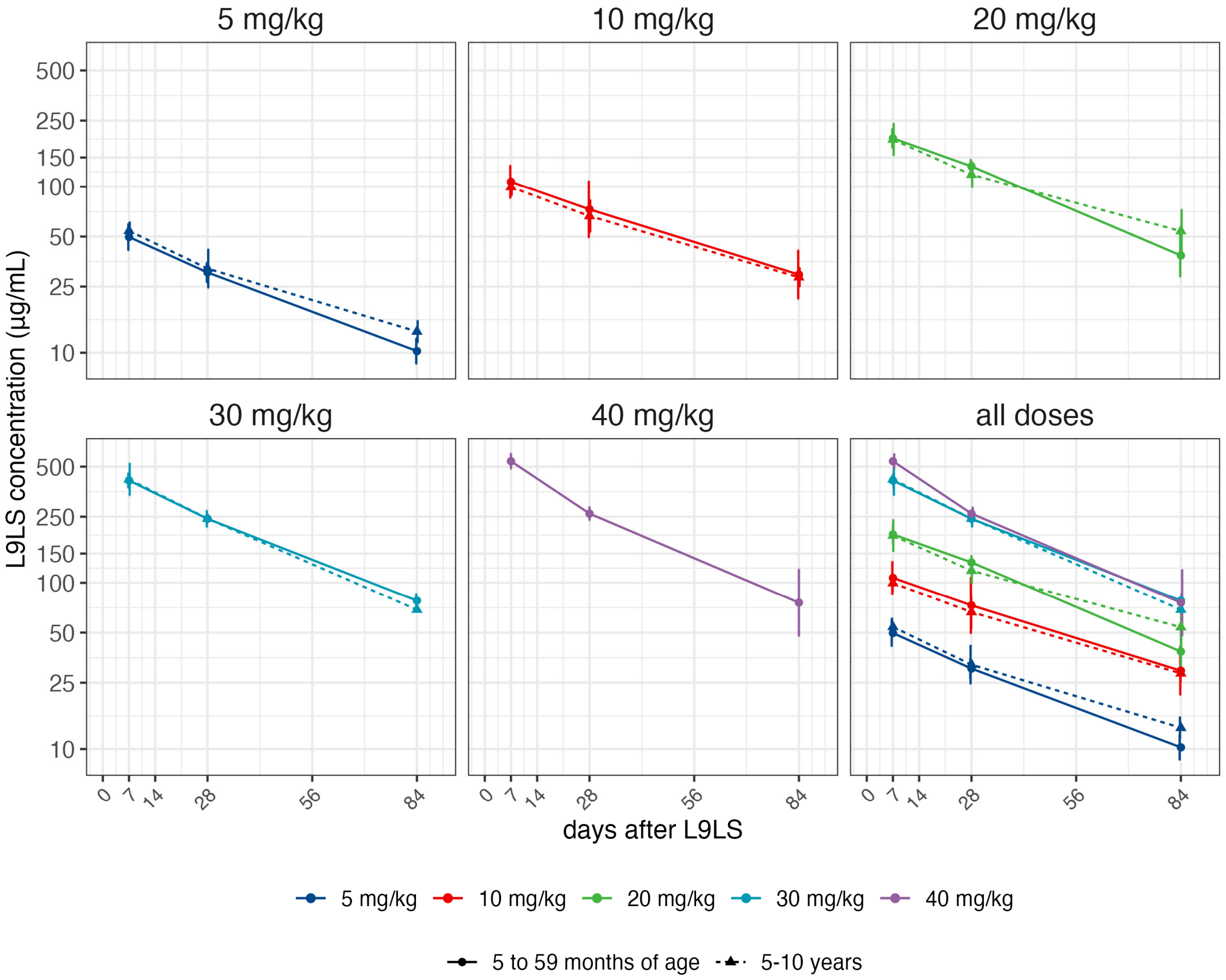

Legend: Data are for participants in Part 1a. Error bars indicate the geometric standard deviation around the geometric mean. In the 30 mg/kg dose group, two of the participants were slightly older than 59 months (63 months and 71 months at the time of L9LS administration).

Table S 10: C_{max} by dose received and age group

Dose number	Part	Dose	Age group	N	Geometric mean	Geometric CV
Only dose	Part 1a	5 mg/kg	5 to 59 months	9	49.68	19.05
Only dose	Part 1a	5 mg/kg	5-10 years	9	54.11	12.73
Only dose	Part 1a	5 mg/kg	5 mos to 10 years	18	51.85	16.32
Only dose	Part 1a	10 mg/kg	5 to 59 months	9	106.97	23.55
Only dose	Part 1a	10 mg/kg	5-10 years	9	99.46	12.24
Only dose	Part 1a	10 mg/kg	5 mos to 10 years	18	103.14	18.54
Only dose	Part 1a	20 mg/kg	5 to 59 months	9	195.71	13.64
Only dose	Part 1a	20 mg/kg	5-10 years	9	192.48	22.90
Only dose	Part 1a	20 mg/kg	5 mos to 10 years	18	194.09	18.26
Only dose	Part 1b	30 mg/kg	5-71 months	8	414.23	12.56
Only dose	Part 1b	40 mg/kg	5-71 months	9	539.13	10.74
1st/only dose	Part 2	10.00-12.50 mg/kg	18 to 59 months at dose 1	13	73.72	19.38
1st/only dose	Part 2	10.00-12.50 mg/kg	5 to 17 months at dose 1	58	89.75	14.99
1st/only dose	Part 2	12.51-15.00 mg/kg	18 to 59 months at dose 1	25	114.19	14.67
1st/only dose	Part 2	12.51-15.00 mg/kg	5 to 17 months at dose 1	44	96.66	72.98
1st/only dose	Part 2	15.01-17.50 mg/kg	18 to 59 months at dose 1	42	121.23	19.76
1st/only dose	Part 2	15.01-17.50 mg/kg	5 to 17 months at dose 1	2	133.07	2.08
1st/only dose	Part 2	17.51-20 mg/kg	5 to 17 months at dose 1	24	135.27	12.29
2nd dose	Part 2	10.00-12.50 mg/kg	18 to 59 months at dose 1	6	101.01	6.89
2nd dose	Part 2	10.00-12.50 mg/kg	5 to 17 months at dose 1	27	90.76	18.85
2nd dose	Part 2	12.51-15.00 mg/kg	18 to 59 months at dose 1	18	108.09	16.42
2nd dose	Part 2	12.51-15.00 mg/kg	5 to 17 months at dose 1	19	106.39	10.56
2nd dose	Part 2	15.01-17.50 mg/kg	5 to 17 months at dose 1	22	98.57	71.22
2nd dose	Part 2	17.51-20 mg/kg	5 to 17 months at dose 1	5	122.84	14.18

Note: Data are from Day 7 in part 1/1b and from day 28 (after dose 1 or after dose 2) in part 2; CV = coefficient of variation

Figure S 18: Serum concentrations by L9LS dose and last measured concentration.

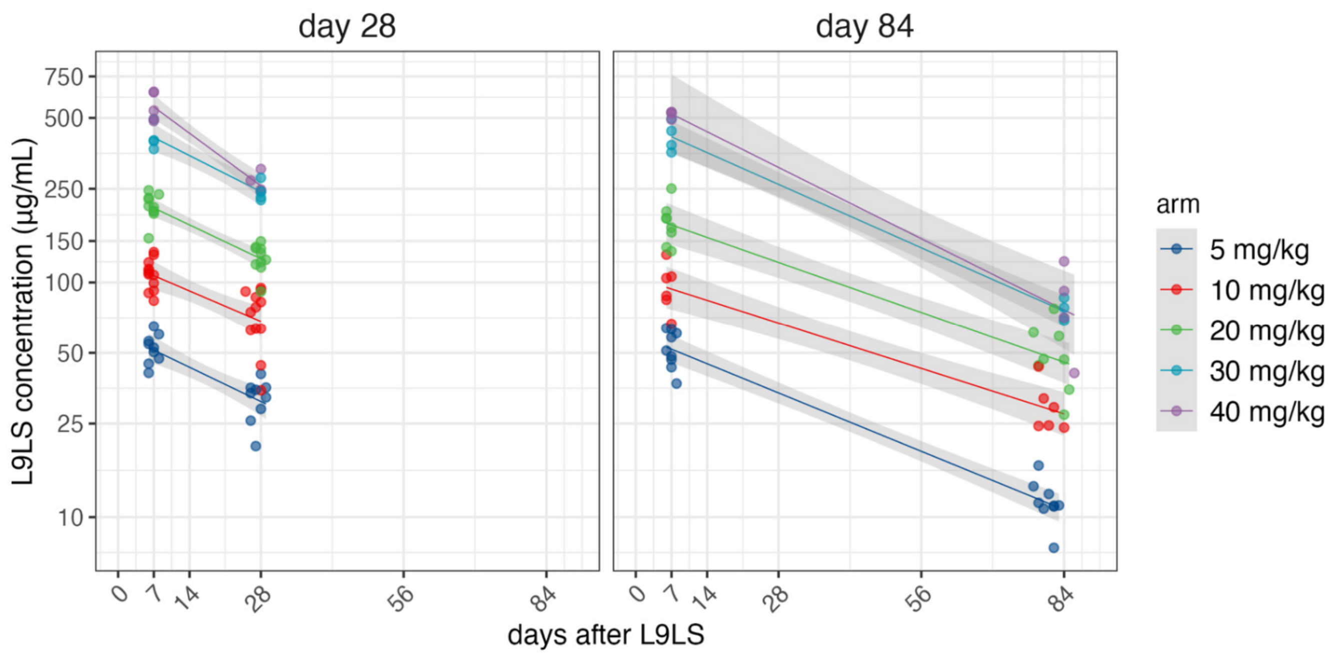

Legend: Points are individual observations for participants in Part 1a by dose group. Participants had a blood draw for serum measurement on day 7 and were randomized to have another blood draw on day 28 or on day 84. Shown are linear regression fits with 95% confidence bands for each dosing arm.

Table S 11: Linear mixed effects models examining differences in concentration versus time slopes by dosing arm

	Last observation day 28				Last observation day 84			
**Characteristic**	**N**	**Beta**	**95% CI**	**p-value**	**N**	**Beta**	**95% CI**	**p-value**
dose								
40 mg/kg	10	—	—		8	—	—	
5 mg/kg	18	-2.5	-2.7, -2.2	<0.0001	18	-2.3	-2.7, -2.0	<0.0001
10 mg/kg	24	-1.7	-2.0, -1.5	<0.0001	12	-1.8	-2.1, -1.4	<0.0001
20 mg/kg	20	-1.1	-1.3, -0.83	<0.0001	16	-1.1	-1.4, -0.81	<0.0001
30 mg/kg	8	-0.38	-0.67, -0.085	0.012	8	-0.24	-0.61, 0.13	0.20
day	80	-0.036	-0.044, -0.029	<0.0001	62	-0.025	-0.029, -0.021	<0.0001
dose * day	80				62			
5 mg/kg * day	18	0.012	0.0026, 0.021	0.014	18	0.0055	0.00048, 0.010	0.033
10 mg/kg * day	24	0.015	0.0063, 0.024	0.0014	12	0.010	0.0042, 0.015	0.0011
20 mg/kg * day	20	0.012	0.0029, 0.021	0.012	16	0.0079	0.0028, 0.013	0.0037
30 mg/kg * day	8	0.011	-0.00047, 0.022	0.060	8	0.0028	-0.0031, 0.0087	0.34

Legend: Separate models were evaluated for Part 1a participants for whom the last observed concentration was either at day 28 or at day 84. In each model, natural log-transformed L9LS concentration was the dependent variable; time (day) and dose were independent variables with an interaction term between dose and time (dose * day); and participant was modeled as a random effect. The reference level for interaction is 40 mg/kg * day. N = number of observations (i.e., serum measurement, with 2 measurements per participant included here) for 40 participants in the "day 28" table and 31 participants in "day 84" table, CI = confidence interval.

Figure S 19: Geometric mean serum concentrations versus time at 5 mg/kg L9LS administered subcutaneously (SC) in current study participants compared to US adults from the VRC 614 study.

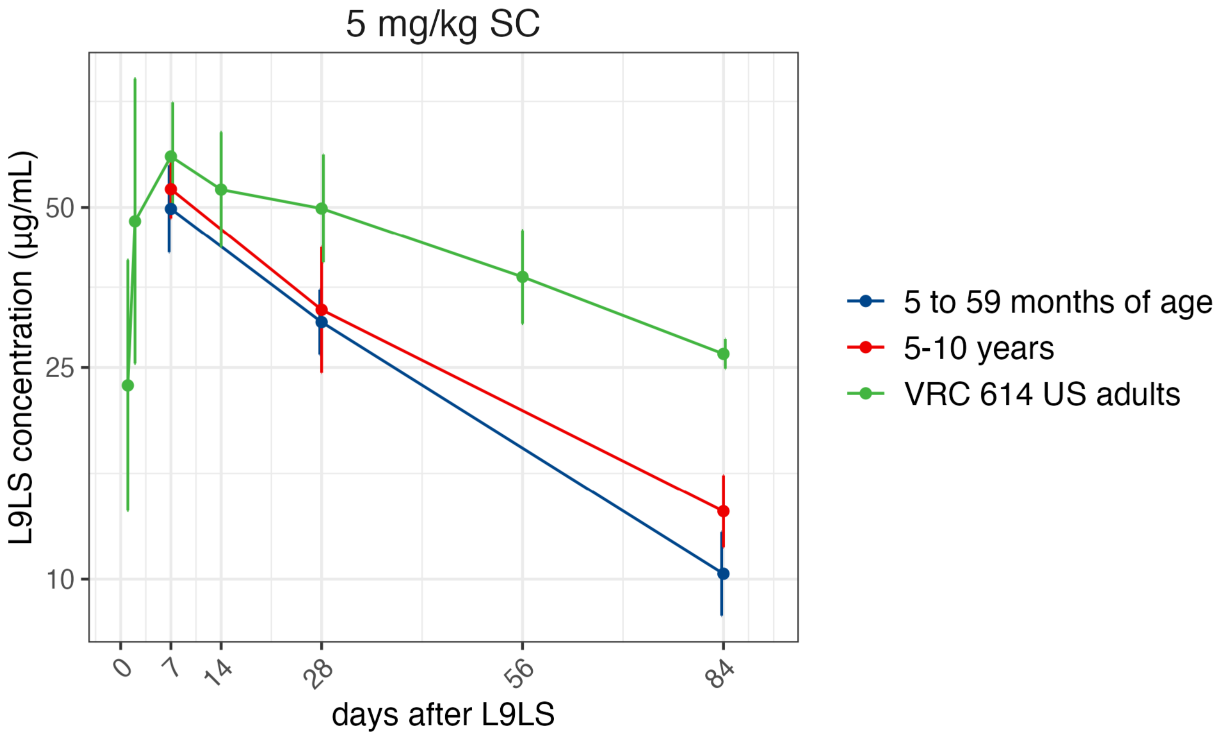

Legend: Data for paediatric participants are from Part 1a. Error bars indicate the geometric standard deviation around the geometric mean.

Figure S 20: Comparison of estimated L9LS half-lives between age groups

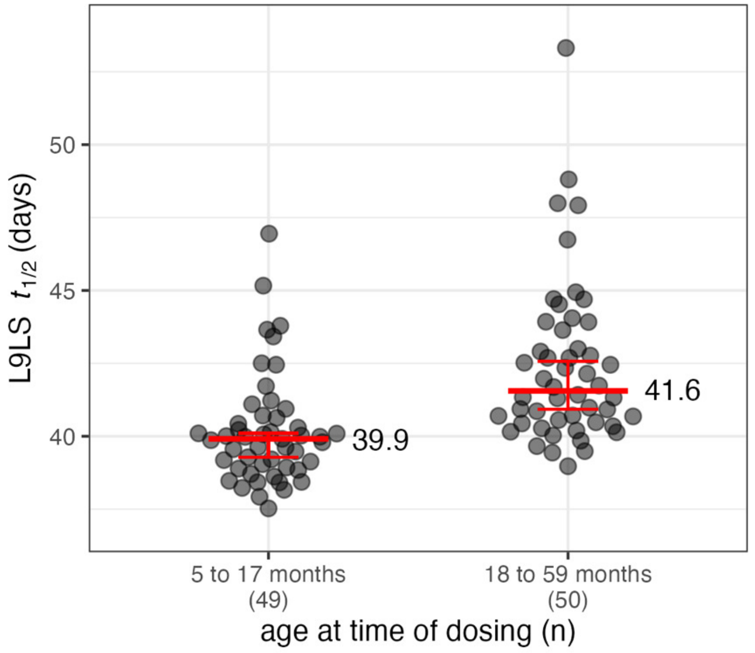

Legend: L9LS terminal elimination half-lives ($t_{1/2}$) were estimated by non-compartmental analysis for participants in Part 2 with 3 time points by fitting the natural logarithm of concentration by time in the

"PKNCA" package. Significance was determined by the Wilcoxon rank sum test.

Figure S 21: Relationship between age and estimated L9LS half life

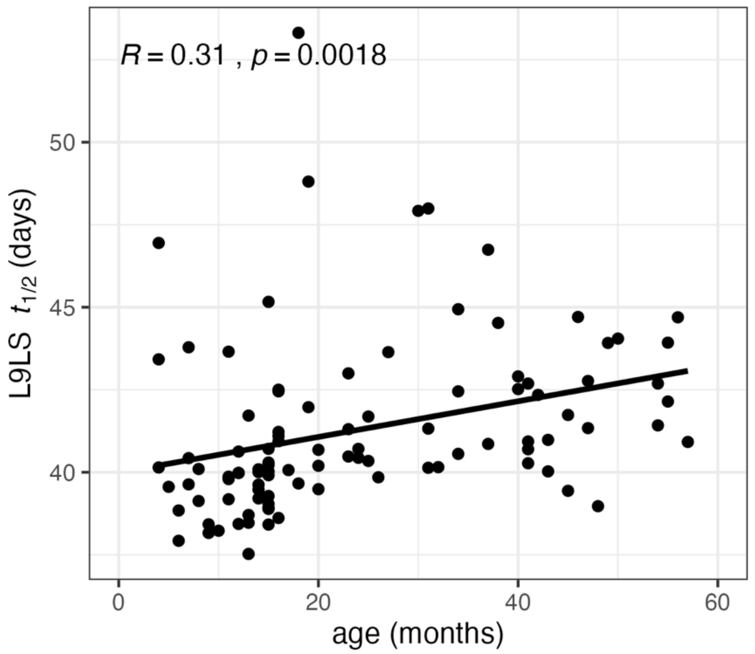

Legend: L9LS terminal elimination half-lives ($t_{1/2}$) were estimated by non-compartmental analysis using the "PKNCA" package for participants in Part 2 who received a single dose of L9LS and had L9LS serum concentrations for 3 time points. Shown are individual half-life estimates with a linear regression fit. P-value determined by Pearson's correlation.

Figure S 22: Antigen-specific fragment binding of study participant exhibiting ADA against L9LS

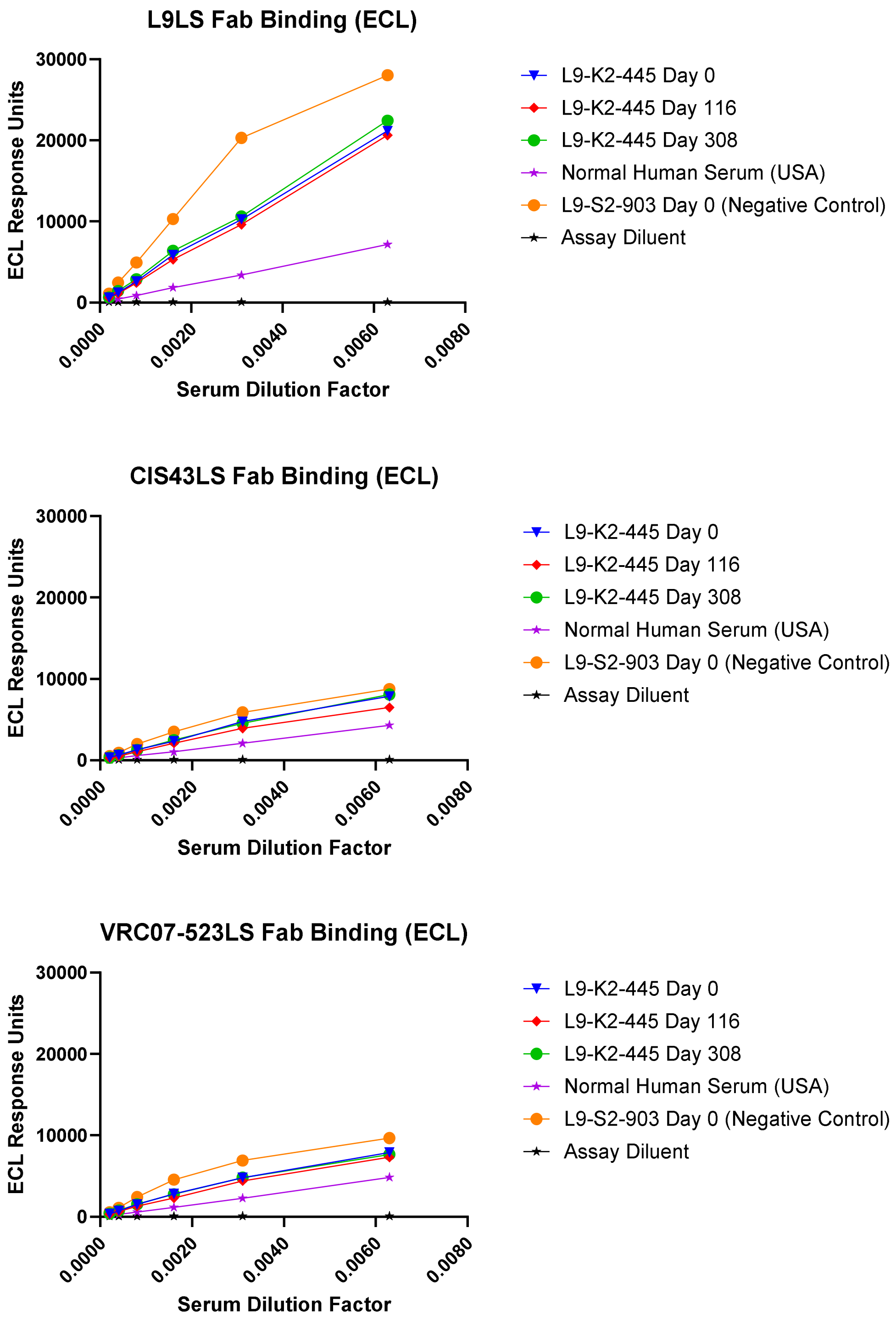

Legend: Participant L9-K2-445 with ADA received placebo. Binding is presented for the Fab portion of L9LS (top panel), with CIS43LS and VRC07-523LS Fabs (middle and bottom panels) used as controls. Binding is higher than in a USA-derived normal human serum sample but does not increase with time and is lower than binding from a randomly selected participant from the trial with negative ADA (L9-S2-903).

Table S 12: Weight-based dosing bands and resulting L9LS concentrations in Part 2

Subject weight (kg)	Total dose	Total volume (mL)	Effective dose in mg/kg
5	75	0.5	15.0
6	75	0.5	12.5
7.0 - 7.5	75	0.5	10.3
7.6 - 7.9	150	1.0	19.4
8	150	1.0	18.8
9	150	1.0	16.7
10	150	1.0	15.0
11	150	1.0	13.6
12	150	1.0	12.5
13	150	1.0	11.5
14	150	1.0	10.7
15.0	150	1.0	10.0
15.1 - 15.9	225	1.5	14.5
16	225	1.5	14.1
17	225	1.5	13.2
18	225	1.5	12.5
19	225	1.5	11.8
20	225	1.5	11.3
21	225	1.5	10.7
22	225	1.5	10.2
22.5	225	1.5	10.0

Figure S 23: Weight-based L9LS dose concentrations at dose 1, by age group

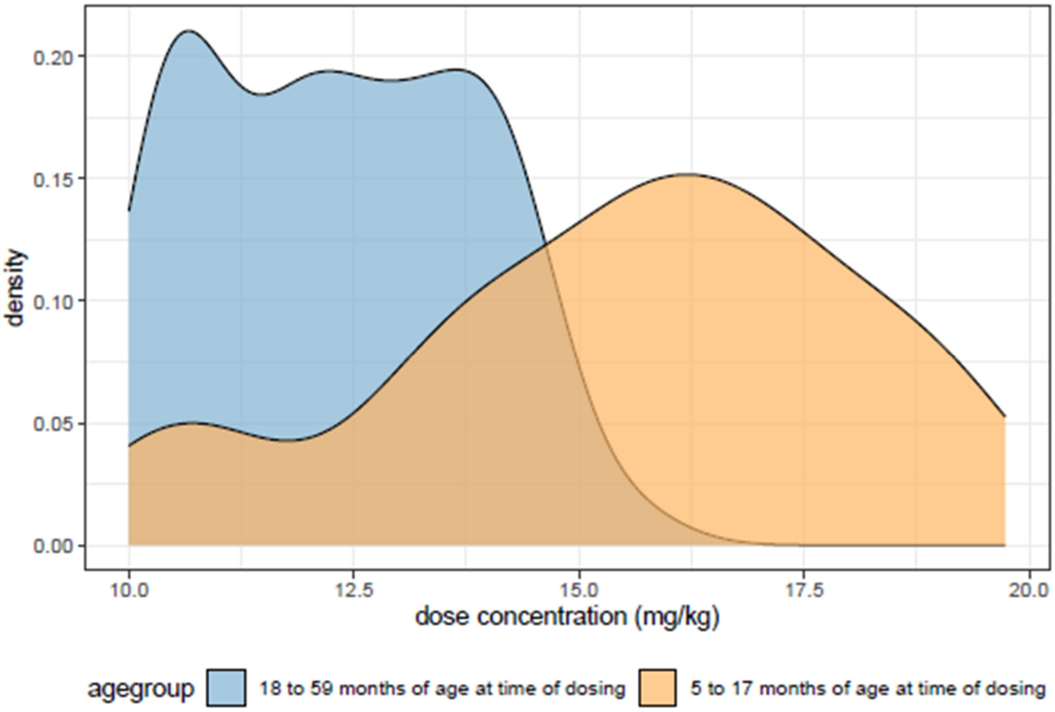

Figure S 24: Plots of protective efficacy versus dose for Pf infection and clinical malaria endpoints

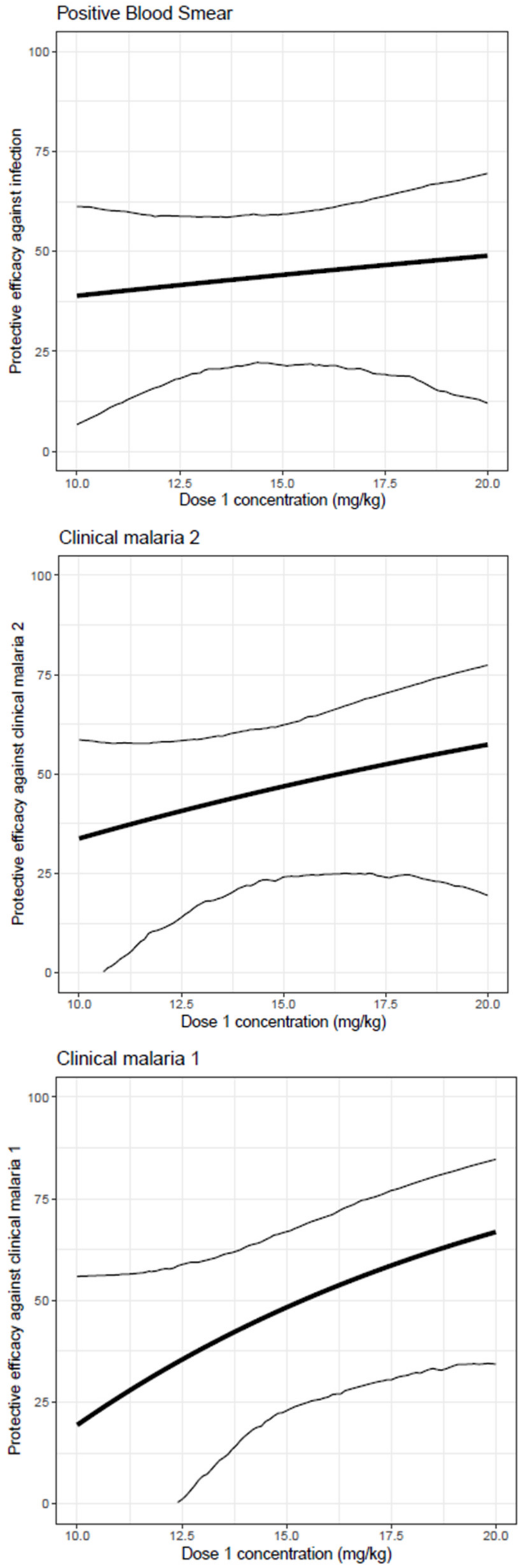

Legend: Relationships derived from Cox regression models, with the response as the infection/clinical malaria outcome over 6 months and the weight-based dose as a covariate.

Study Protocol

Abbreviated Title: Anti-malaria MAb in Kenyan children

Protocol #: 4413

Version 6.0 **Date:** 2 Apr 2024

Title: Safety and Efficacy of L9LS, a Human Monoclonal Antibody Against *Plasmodium falciparum*, in an Age De-Escalation, Dose-Escalation Trial and a Randomized, Placebo-Controlled, Double-Blind Trial of Children in Western Kenya

Study Identifiers:				
KEMRI SSC#4413	NIH			

Co-Principal Investigators:

Titus Kwambai, MD, PhD, Epidemiologist Centers for Disease Control and Prevention Kenya Malaria Program Phone: +254 722207281 Email: qbb5@cdc.gov

Laura Steinhardt, PhD, MPH Malaria Branch, US CDC 1600 Clifton Rd., Atlanta, GA 30329 Phone: +1-404-718-4794 Email: LSteinhardt@cdc.gov

Peter D. Crompton, MD, MPH, Chief, Malaria Infection Biology and Immunity Section Laboratory of Immunogenetics (LIG), National Institute of Allergy and Infectious Diseases (NIAID), National Institutes of Health (NIH), Phone: 240-383-7640 Email: pcrompton@niaid.nih.gov

Co-Investigators:

NIH:

Robert A. Seder, MD, NIH Protocol Chair: Chief, Cellular Immunology Section, Vaccine Research Center (VRC), National Institute of Allergy and Infectious Diseases (NIAID), NIH, Phone: 301-594-8483, Email: rseder@mail.nih.gov

CDC:

Julie Gutman, MD, MS, Malaria Branch US CDC,1600 Clifton Rd., Atlanta, GA 30329 Phone: 404-718-4730, Email: fff2@cdc.gov Role(s): D, F, G

Peter McElroy, PhD, MPH,Malaria Branch US CDC,1600 Clifton Rd., Atlanta, GA 30329 Phone: 404-718-4724, Email : pgm9@cdc.gov, Role (s): F, G

LSTM:

Feiko ter Kuile, MD, PhD, Liverpool School of Tropical Medicine, Pembroke Place, Liverpool L3 5QA, UK, Mobile: +44 (0)7846 377 369 (UK), +254 (0)708 739 228 (Kenya) Email: feiko.terkuile@lstmed.ac.uk, Role(s): B, E, F

KEMRI:

Eunice Ouma, MD, KEMRI Center for Global Health Research (CGHR) P.O. Box: 1578 - 40100, Kisumu, Kenya , Phone: +254722324496, E-mail: eunice.ouma@lstmed.ac.uk, Role(s): A, B, C, D, E, F, G

Martina Oneko, MD, KEMRI CGHR,P.O. Box: 1578 - 40100, Kisumu, Kenya, Phone: +254-717-747-279, Email: tinaoneko@gmail.com, Role(s): A, B, C, D, E, F, G

Simon Kariuki, PhD,KEMRI CGHR,Phone: +254 725 389 246, Email: skariuki1578@gmail.com, Role(s): A, B, E, F, G

Ruth Njoroge, BPharm,KEMRI CGHR,P.O. Box: 1578 - 40100, Kisumu, Kenya
Phone: +254 702 964 102,Email: ruthwambuinjoroge1@gmail.com, Role(s): A, B, E, F, G

Kephas Otieno, MSc,KEMRI CGHR, P.O. Box: 1578 - 40100, Kisumu, Kenya
Phone: +254 721 405 643,Email: kotieno72@yahoo.com, Role(s): B, E, F, G

Collaborators:

Sean C. Murphy, MD, PhD,University of Washington,Department of Laboratory Medicine, NW120,Box 357110,1959 Pacific St.,Seattle, WA 98195–7110,Phone: 206-685-6162 Email: murphysc@uw.edu
Role(s): F

Daniel Neafsey, PhD,Harvard T.H. Chan School of Public Health,665 Huntington Avenue, Building 1, Room 711,Boston, MA 02115,Phone: 617-432-5404 Email: neafsey@hsph.harvard.edu
Role(s): F

Edmund Capparelli, PharmD,University of California, San Diego 9500 Gilman Drive La Jolla, CA 92093
Phone: +1-858-246-0009 Email: ecapparelli@ucsd.edu,
Role(s): F

**Investigational Agent:**	
Drug Name	L9LS
Investigational New Drug (IND) Number	160213
Manufacturer	Vaccine Research Center (VRC), NIAID, NIH

Sponsor: OCRPRO, NIH		Office of The Clinical Research Policy and Regulatory Operations, NIH

Revision chronology:

Date	Protocol Version	Details of Changes	Authors	Signature Principal Investigator
10 Feb 2022	*1.0*	*Original*	Laura Steinhardt	
			Titus Kwambai	
22 March 2022	*2.0*	*Revised*	Laura Steinhardt	
			Titus Kwambai	
29 July 2022	*3.0*	*Revised*	Laura Steinhardt	
			Titus Kwambai	
23 Nov 2022	*4.0*	*Revised*	Laura Steinhardt	
			Titus Kwambai	
12 Sep 2023	*5.0*	*Revised*	Laura Steinhardt	
		Revised	Titus Kwambai	
2 Apr 2024	*6.0*	*Revised*	Laura Steinhardt	
			Titus Kwambai	

Confidentiality Statement: This document contains confidential information that must not be disclosed to anyone other than the sponsor, the investigator team, host institution, relevant ethics committee and regulatory authorities

Data and Safety Monitoring Board (DSMB): NIAID Intramural DSMB

STUDY STAFF ROSTER

CDC Investigators:

CDC investigators are involved in study design, implementation, analysis of coded samples and data, and writing and dissemination of reports of study results. The CDC Co-Principal Investigators will have access to identifiable data.

The research activities of the United States (US) CDC investigators will be reviewed by:

Scientific and Ethics Review Unit (SERU)

Kenya Medical Research Institute (KEMRI)
P.O. Box 54840 00200 Off Mbagathi Road.

Nairobi, Kenya.
Tel : +254 722205901

Email : kemriseru18@gmail.com

CDC Principal Investigator: Laura Steinhardt, PhD, MPH
Malaria Branch, US CDC

1600 Clifton Rd., Atlanta, GA 30329
Phone: +1-404-718-4794
Email: LSteinhardt@cdc.gov
CITI Expiration: December 24, 2024
Role(s): B, E, F, G

CDC Kenya Principal Investigator: Titus Kwambai, MD PhD

US CDC, Kenya Malaria Program

Address: P.O. Box: 1578 - 40100

Kisumu, Kenya

Phone: +254 722207281

Email: qbb5@cdc.gov

CITI Expiration: March 10, 2023

Role(s): A, B, C, D, E, F, G

CDC Co-Investigators: Julie Gutman, MD, MS
Malaria Branch US CDC

1600 Clifton Rd., Atlanta, GA 30329

Phone: 404-718-4730

Email: fff2@cdc.gov

CITI Expiration: July 17, 2025

Role(s): D, F, G

Peter McElroy, PhD, MPH
Malaria Branch US CDC

1600 Clifton Rd., Atlanta, GA 30329

Phone: 404-718-4724

Email : pgm9@cdc.gov

CITI Expiration: May 25, 2025

Role (s) : F, G

KEMRI Investigators:

The research activities of the KEMRI investigators will be reviewed by:

SERU Ethics Committee
Kenya Medical Research Institute
P.O. Box 54840 00200 Off Mbagathi Road.

Nairobi, Kenya.
Tel : +254 722205901

Email : kemriseru18@gmail.com

KEMRI Co-Investigators: Eunice Ouma, MD

KEMRI Center for Global Health Research (CGHR)

P.O. Box: 1578 - 40100, Kisumu, Kenya Phone:

E-mail: eunice.ouma@lstmed.ac.uk

Role(s): A, B, C, D, E, F, G

Martina Oneko, MD
KEMRI CGHR

P.O. Box: 1578 - 40100, Kisumu, Kenya
Phone: +254-717-747-279
Email: tinaoneko@gmail.com
Role(s): A, B, C, D, E, F, G

Simon Kariuki, PhD
KEMRI CGHR
Phone: +254 725 389 246
Email: skariuki1578@gmail.com
Role(s): A, B, E, F, G

Ruth Njoroge, BPharm
KEMRI CGHR

P.O. Box: 1578 - 40100, Kisumu, Kenya
Phone: +254 702 964 102
Email: ruthwambuinjoroge1@gmail.com
Role(s): A, B, E, F, G

Kephas Otieno, MSc

KEMRI CGHR

P.O. Box: 1578 - 40100, Kisumu, Kenya
Phone: +254 721 405 643

Email: kotieno72@yahoo.com
Role(s): B, E, F, G

NIH, LSTM and other non-CDC U.S. Investigators:

NIH, LSTM and other non-CDC U.S. Investigators are involved in study design, implementation, analysis of coded samples and data, and writing and dissemination of reports of study results. Although they may support KEMRI and CDC investigators in monitoring/oversight capacities, NIH, LSTM, and other non-CDC U.S. investigators will not be engaged in human subjects research.

NIH Protocol Chair: Robert A. Seder, MD
Chief, Cellular Immunology Section
VRC, NIAID, NIH
Phone: 301-594-8483
Email: rseder@mail.nih.gov
Role(s): F

NIH Principal Investigator and Referral Contact: Peter D. Crompton, MD, MPH
Laboratory of Immunogenetics (LIG), NIAID, NIH
5625 Fishers Lane Room 4N07D
Rockville, MD 20852
Phone: 240-383-7640
Email: pcrompton@niaid.nih.gov
Role(s): F

Statistician: Zonghui Hu, PhD
Biostatistics Research Branch, NIAID, NIH
Phone: 240-669-5240
Email: huzo@niaid.nih.gov
Role(s): F

LSTM Co-investigator Feiko ter Kuile, MD, PhD

Liverpool School of Tropical Medicine, Pembroke Place,
Liverpool L3 5QA, UK,

Mobile: +44 (0)7846 377 369 (UK), +254 (0)708 739 228 (Kenya)

Role(s): B, E, F

Sean C. Murphy, MD, PhD

University of Washington

Collaborators: Department of Laboratory Medicine, NW120

Box 357110

1959 Pacific St.

Seattle WA 98195-7110

Phone: 206-685-6162

Email : murphysc@uw.edu

Role (s) : F

Daniel Neafsey, PhD

Harvard T.H. Chan School of Public Health

665 Huntington Avenue

Building 1, Room 711

Boston, MA 02115

Phone : 617-432-5404

Email : neafsey@hsph.harvard.edu

Role (s) : F

Edmund Capparelli, PharmD

University of California, San Diego

9500 Gilman Drive

La Jolla, CA 92093

Phone : +1-858-246-0009

Email : ecapparelli@ucsd.edu

Role (s) : F

Email : murphysc@uw.edu

Role (s) : F

Daniel Neafsey, PhD

Harvard T.H. Chan School of Public Health

665 Huntington Avenue

Building 1, Room 711

Boston, MA 02115

Phone : 617-432-5404

Email : neafsey@hsph.harvard.edu

Role (s) : F

Edmund Capparelli, PharmD

University of California, San Diego

9500 Gilman Drive

La Jolla, CA 92093

Phone : +1-858-246-0009

Email : ecapparelli@ucsd.edu

Role (s) : F

Role definitions:

A. Obtain information by intervening or interacting with living individuals for research purposes

B. Obtaining identifiable private information about living individuals

C. *Obtaining the voluntary informed consent of individuals to be subjects*
D. *Makes decisions about subject eligibility*
E. *Studying, interpreting, or analyzing identifiable private information or data/specimens for research purposes*
F. *Studying, interpreting, or analyzing coded (linked) data or specimens for research purposes*
G. *Some/all research activities performed outside NIH*

Study Site: KEMRI CGHR

Siaya Clinical Research Centre
Siaya County Referral Hospital, Siaya County
Kenya

Kogelo Dispensary, Siaya County

Kenya

Sponsor Medical Monitor (SMM): Saran Wells, MD (Contractor)

Leidos Biomedical Research, Inc.

In support of NIAID, NIH

Phone 240-529-4337

Email: saran.wells@nih.gov

Independent Safety Monitor (ISM): Dr. Janet Oyieko, MD
KEMRI CCR, Po Box 54 -40100 Kisumu.
Email : janet.oyieko@gmail.com.
Tel +254721996988

US Office of Managaement and Budget/Paperwork Reduction Act (OMB/PRA) Information

This study is being sponsored by the Office of Clinical Research Policy and Regulatory Operations (OCRPRO) within the National Institute of Allergies and Infectious Diseases (NIAID) within the US National Institutes of Health (NIH). The data collection in western Kenya is being carried out by KEMRI, which will initiate, direct, collect and own the data arising from this study. Data will be shared with CDC, LSTM, and NIH at KEMRI's discretion. CDC and NIH staff are providing technical assistance for protocol development, training of

staff, collection of data, data analysis, laboratory analyses, and preparation of scientific manuscripts.

Funding for this study is being provided the Bill and Melinda Gates Foundation (BMGF), Grant ID# INV-035091. Funding from BMGF is provided to the CDC via a gift from the Centers for Diseases Control and Prevention (CDC) Foundation. CDC is providing funding to LSTM via a Cooperative Agreement (#GH002290) and LSTM (Federalwide Assurance Number: FWA00001877) is providing funding to KEMRI (Federalwide Assurance Number: FWA00002066) in a subagreement. The Vaccine Research Center (VRC) of NIAID (Federalwide Assurance Number: FWA00005897) is providing the investigational product for this trial.

TABLE OF CONTENTS

STUDY STAFF ROSTER 67
TABLE OF CONTENTS..74
List of Figures..78
List of Tables 78
ABBREVIATIONS 79
STATEMENT OF COMPLIANCE 83
1 ....PROTOCOL SUMMARY 84
1.1 Abstract..84
1.3 Schema..92
1.4 Schedule of Activities (SOA) 96
1.4.1 Part 1 Study Schedule 96
1.4.2 Part 2 Study Schedule 99
1.4.3 Part 1b Study Schedule 103
1.4.4 Part 2 Extension Study Schedule 106
2 .... INTRODUCTION 110
2.1 Study Rationale 110
2.2 Background 110
2.2.1 Study Agent: L9LS 112
2.2.2 Laboratory Assessments of L9LS 115
2.2.3 Repeat Dosing: ADA, PK 117
2.3 Risk/Benefit Assessment..119
2.3.1 Known Potential Risks 119
2.3.2 Known Potential Benefits 122
2.3.3 Assessment of Potential Risks and Benefits 122
3 .... OBJECTIVES AND ENDPOINTS 123
4 .... STUDY DESIGN 128
4.1 Overall Design 128
4.1.1 Year 2 Follow-on Study (Safety and Extension) 130
4.2 Scientific Rationale for Study Design 132
4.2.1 Year 2 Follow-on Study (Safety and Extension) 132

4.2.2 Justification for Dose 133

5 .... STUDY POPULATION 134

5.1 Inclusion Criteria 134

5.1.1 Year 2 Extension Inclusion Criteria 134

5.2 Exclusion Criteria 134

5.3 Inclusion of Vulnerable Participants 136

5.4 Inclusion of Pregnant Women, Fetuses or Neonates 137

5.5 Lifestyle Considerations 137

5.6 Screen Failures 137

5.7 Strategies for Recruitment and Retention 138

5.7.1 Costs 138

5.7.2 Compensation 138

5.8 COVID-19 Risk Mitigation 139

6 .... STUDY INTERVENTION 139

6.1 Study Interventions(s) Administration 139

6.1.1 Study Intervention Description 139

6.1.2 Dosing and Administration. 139

6.2 Preparation/Handling/Storage/Accountability 141

6.2.1 Acquisition and Accountability 141

6.2.2 Formulation, Appearance, Packaging, and Labeling 142

6.2.3 Product Storage and Stability 142

6.2.4 Preparation 143

6.3 Measures to Minimize Bias: Randomization and Blinding 144

6.3.1 Randomization 144

6.3.2 Blinding 145

6.4 Study Intervention Compliance 145

6.5 Concomitant Therapy 145

7 .. STUDY INTERVENTION DISCONTINUATION AND PARTICIPANT DISCONTINUATION/WITHDRAWAL 146

7.1 Discontinuation of Study Intervention 146

7.2 Participant Discontinuation/Withdrawal from the Study 147

7.3 Lost to Follow-up 147

8 ....STUDY ASSESSMENTS AND PROCEDURES 147

8.1 Screening and Pre-Enrollment Procedures 147

8.2 Efficacy Assessments 149

8.2.1 Clinical Evaluations 149

8.2.2 Biospecimen Evaluations 150

8.2.3 Correlative Studies for Research/Pharmacokinetic Studies 151

8.2.4 Samples for Genomic Analysis 152

8.3 Safety and Other Assessments 152

8.4 Safety Definitions, Management, and Sponsor Reporting 152

8.4.1 Definitions 152

8.4.2 Documenting, Assessing, Recording, and Reporting Events 156

8.4.3 Withdrawal Criteria for an Individual Subject 162

8.4.4 Additional Safety Oversight 163

8.4.5 Pausing Rules 164

8.4.6 Halting Rules for the Protocol 165

8.5 Unanticipated Problems 166

8.5.1 Definition of Unanticipated Problems 166

8.5.2 Unanticipated Problem Reporting 166

8.6 ADDITIONAL REPORTING REQUIREMENTS 166

8.6.1 Protocol Deviation Reporting 166

8.6.2 Reporting Protocol Deviations that Result from the COVID-19 Pandemic 167

9 ....STATISTICAL CONSIDERATIONS 168

9.1 Statistical Hypothesis 168

9.2 Sample Size Determination 169

9.2.1 Sample Size Considerations for the Dose-Escalation Study 169

9.2.2 Sample Size Considerations for the Efficacy Study 169

9.3 Sample Size Considerations for the Year 2 Extension Study 174

9.4 Populations for Analyses 175

9.4.1 Evaluable for toxicity 176

9.4.2 Evaluable for objective response 176

9.4.3 Evaluable Non-Target Disease Response 176

9.5 Statistical Analyses 176

9.5.1 General Approach 176
9.5.2 Analysis of the Primary Endpoints 177
9.5.3 Analysis of the Secondary Endpoint(s) 177
9.5.4 Efficacy Analyses 177
9.5.5 Safety Analyses 179
9.5.6 Pharmacokinetics Analysis 179
9.5.7 Baseline Descriptive Statistics 180
9.5.8 Planned Interim Analyses 180
9.5.9 Sub-Group Analyses 180
9.5.10 Tabulation of individual Participant Data 181
9.5.11 Exploratory Analyses 181
10 .... REGULATORY AND OPERATIONAL CONSIDERATIONS 181
10.1 Informed Consent Process 181
10.1.1 Consent/Assent Procedures and Documentation 181
10.2 Study Discontinuation and Closure 182
10.3 Confidentiality and Privacy 182
10.4 Future use of Stored Specimens and Data 183
10.5 Safety Oversight 183
10.6 Clinical Monitoring 184
10.7 Quality Assurance and Quality Control 184
10.8 Data Handling and Record Keeping 184
10.8.1 Data Collection and Management Responsibilities 184
10.8.2 Study Records Retention 185
10.9 Protocol Deviations and Non-Compliance 185
10.9.1 NIH Definition of Protocol Deviation 185
10.10 Publication and Data Sharing Policy 185
10.10.1 Human Data Sharing Plan 185
10.10.2 Genomic Data Sharing Compliance 186
10.11 Collaborative Agreements 186
10.11.1 Agreement Type 186
10.12 Conflict of Interest Policy 186
11.1 Budget 186

12 .... REFERENCES
....189
APPENDIX A: KENYA NATIONAL IMMUNIZATION SCHEDULE 191
APPENDIX B: KENYA ADVERSE EVENT GRADING SCALE..192
PART 1 AND 1B: 192
PART 2 AND THE EXTENSION PHASE:...193

LIST OF FIGURES

Figure 1. Enrollment and study flow schematics for part 1. 92
Figure 2. Study flow schematic for part 2. 94
Figure 3. Study flow schematic for part 1b (additional safety study) 95
Figure 4. Study flow scheme for part 2 extension study 95

LIST OF TABLES

Table 1. Study agent assignment and dosing by age group/arm 140
Table 2. Dosing of L9LS (10 to 20 mg/kg) by weight of child for part 2....................................143
Table 3. Dosing schedule for dihydroartemisinin piperaquine 149
Table 4. Standard event recording, assessment, and reporting timeframes. 157
Table 5. Probability of events for different safety scenarios within an arm (n=9). 169
Table 6. Sample size calculations based on secondary 12-month endpoint, to achieve 80% power to detect efficacy of 70% at 6 months and 60% at 12 months, assuming 35% of the control group are infected by 6 months and 45% by 12 months. 171
Table 7. The minimum detectable difference in event rate between L9LS arm and placebo arm 174
Table 8.The comparison power with 108 participants per arm and a lost to follow up rate of 20% 175
Table 9. The comparison power between two L9LS arms with 108 participants per arm and a lost to follow up rate of 20% 175

ABBREVIATIONS

ADA	Anti-Drug Antibody
AE	Adverse Event
AL	Artemether-Lumefantrine
ALT	Alanine Transaminase
AR	Adverse Reaction
AUC	Area Under the Concentrations vs. Time Curve
Cave	Average Concentrations
CDC	Centers for Disease Control and Prevention
CBC	Complete Blood Count
CFR	Code of Federal Regulations
CGHR	Center for Global Health Research
cGMP	Current Good Manufacturing Practices
CHMI	Controlled Human Malaria Infection
CL	Clearance
Cmax	Maximum Concentration
CONSORT	Consolidated Standards of Reporting Trials
Cr	Creatinine
CRF	Case Report Form
CRS	Cytokine Release Syndrome
CSO	Clinical Safety Office
CTM	Clinical Trials Management
DCR	Division of Clinical Research
DNA	Deoxyribonucleic Acid
DP	Dihydroartemisinin-piperaquine
DS	Drug Substance
DSMB	Data and Safety Monitoring Board
EC	Effective Concentration

ED	Effective Dose
FDA	Food and Drug Administration
GCP	Good Clinical Practice
GLP	Good Laboratory Practices
HIV	Human Immunodeficiency Virus
ICH	International Council on Harmonisation
IND	Investigational New Drug Application
IPC	Infection Prevention and Control
IRB	Institutional Review Board
ISM	Independent Safety Monitor
ITT	Intention-To-Treat
IV	Intravenous(ly)
KEMRI	Kenya Medical Research Institute
KPPB	Kenya Pharmacy and Poisons Board
KSh	Kenyan Shilling
LIG	Laboratory of Immunogenetics
MAb	Monoclonal Antibody
MITT	Modified Intention-To-Treat
MoH	Ministry of Health
NC	Non-Compartmental
NCT	National Clinical Trial
NHP	Non-Human Primate
NIAID	National Institute of Allergy and Infectious Diseases
NIH	National Institutes of Health
NOAEL	No Observed Adverse Effect Level
OCRPRO	Office of Clinical Research Policy and Regulatory Operations
OHRP	Office for Human Research Protections
PCR	Polymerase Chain Reaction

Pf	*Plasmodium falciparum*
PfCSP	*Plasmodium falciparum* Circumsporozoite Protein
PK	Pharmacokinetic(s)
PP	Per-Protocol
Q	Intercompartmental Clearance
QL	Quantitative Limit
RDT	Rapid Diagnostic Test
REDCap	Research Electronic Data Capture
REF	Reportable Event Form
RNA	Ribonucleic Acid
rRNA	Ribosomal Ribonucleic Acid
RT-PCR	Reverse Transcription Polymerase Chain Reaction
SAE	Serious Adverse Event
SAR	Suspected Adverse Reaction
SC	Subcutaneous
SD	Standard Deviation(s)
SERF	Safety Expedited Report Form
SERU	Scientific and Ethics Review Unit
SMM	Sponsor Medical Monitor
SOA	Schedule of Activities
SOP	Standard Operating Procedure
SUSAR	Serious and Unexpected Suspected Adverse Reaction
SRCP	Safety Review and Communication Plan
TCR	Tissue Cross-Reactivity
Tmax	Maximal Concentration
UP	Unanticipated Problem
UPnonAE	Unanticipated Problem that is not an Adverse Event
US	United States

VCMP	Vaccine Clinical Materials Program
Vd	Volume of Distribution
Vdss	Volume of Distribution at Steady-State
VE	Vaccine Efficacy
VRC	Vaccine Research Center
WHO	World Health Organization

STATEMENT OF COMPLIANCE

The trial will be carried out in accordance with International Council on Harmonisation (ICH) Good Clinical Practice (GCP) and the following:

- US Code of Federal Regulations (CFR) applicable to clinical studies (45 CFR Part 46, 21 CFR Part 50, 21 CFR Part 56, 21 CFR Part 312, and/or 21 CFR Part 812).

NIH-funded investigators and clinical trial site staff who are responsible for the conduct, management, or oversight of NIH-funded clinical trials have completed Human Subjects Protection and ICH GCP Training.

The protocol and informed consent/assent forms will be submitted to the Kenya Medical Research Institute's Scientific and Ethics Review Unit (SERU) and to the Kenya Pharmacy and Poisons Board (KPPB) as well as National Commission for Science, Technology and Innovation (NACOSTI) for review and approval. These review boards are collectively referred to as institutional review boards (IRBs) throughout this protocol. Approval of both the protocol and the consent/assent forms must be obtained before any subject is enrolled. Any amendment to the protocol will require review and approval by the IRBs before the changes are implemented to the study. In addition, all changes to the consent/assent forms will be IRB-approved; a determination will be made regarding whether a new consent/assent needs to be obtained from subjects who provided consent/assent using a previously approved form.

PROTOCOL SUMMARY

Abstract

**Title:**	Safety and Efficacy of L9LS, a Human Monoclonal Antibody Against *Plasmodium falciparum*, in an Age De-Escalation, Dose-Escalation Trial and a Randomized, Placebo-Controlled, Double-Blind Trial of Children in Western Kenya
**Study Description:**	A three-part, phase 2 trial evaluating the safety and tolerability of one-time subcutaneous (SC) administration of monoclonal antibody (MAb) L9LS in healthy Kenyan children aged 5 months to 10 years, as well as the safety and protective efficacy of up to four doses of L9LS against naturally occurring *Plasmodium falciparum* (Pf) infection among Kenyan children aged 5 to 59 months at enrollment, in a setting of perennial high transmission, plus the safety and continued efficacy of a third and fourth dose of L9LS in a second year of follow-up. The primary study hypotheses are that L9LS will be safe and will produce protection against malaria infection. Before study agent administration, all subjects will be given dihydro-artemisinin-piperaquine (DP) to clear any preexisting Pf blood-stage infection.

Part 1: Age de-escalation and dose-escalation study

In a stepwise fashion, children aged 5-10 years will receive 5 mg/kg of L9LS or placebo and be followed for 3 months to assess tolerability and safety. If acceptable tolerability and safety profiles are met at 1-week post-injection, we will administer the 5-mg/kg dose of L9LS or placebo to children aged 5-59 months while enrolling another cohort of children aged 5-10 years at a dose of 10 mg/kg of L9LS or placebo. If after 1 week, the 10-mg/kg dose is found to be safe in children aged 5-10 years and the 5-mg/kg dose is found to be safe in children aged 5-59 months, we will then administer a 20-mg/kg dose of L9LS or placebo to children aged 5-10 years and a 10-mg/kg dose of L9LS or placebo to children aged 5-59 months. If these doses are found safe after 1 week, we will administer a 20-mg/kg dose of L9LS or placebo to children aged 5-59 months. Should tolerability and safety of all doses be acceptable in children aged 5-59 months, we will move to part 2 of the trial. Dosing in part 1 of the study will be weight-based and all doses will be administered SC in a double-blinded

fashion. We will enroll 12 participants in each age-dose group in a 3:1 ratio of L9LS to placebo, for a total of 72 participants, and all participants will be followed for a total of 3 months.

Part 1b: Age de-escalation and dose-escalation addition

Based on data from Mali suggesting that doses higher than 20mg/kg are needed to protect for 12 months, we propose to assess the safety of an additional two doses: 30 mg/kg and 40 mg/kg in a similar fashion. This extension of part 1 will include an additional 24 children, and will utilize a similar design to before, with 12 participants in each age-dose group in a 3:1 ratio of L9LS to placebo; all participants will be followed for a total of 3 months. However, given that it appears that the younger children are clearing the product more quickly than older people, we will include only children aged 5-71 months, as these are the ages of the children to be included in the part 2 extension. As before, we will ensure safety out to 7 days before proceeding to dose additional subjects.

Part 2: Efficacy study

A total of 324 children 5-59 months of age will be randomized to receive a 10-20 mg/kg dose of L9LS or placebo by SC administration. There will be two L9LS cohorts, one 5-17 months of age and another 18-59 months of age, which will constitute one L9LS arm. A placebo arm will be composed of children 5–59 months of age. They will be followed over 12 months with monthly blood smear microscopy and polymerase chain reaction (PCR) and twice-monthly symptomatology and care-seeking behavior questionnaires. Dosing will be based on three weight bands; all doses will be administered SC with fixed doses of 75 mg L9LS, 150 mg L9LS, or 225 mg L9LS, resulting in a range of 10-20 mg/kg in a double-blinded fashion. Blood will be drawn to assess antibody titers at baseline, and at three additional time points over 12 months to establish pharmacokinetics (PK). Participants in the L9LS arm will be randomized 1:1 at baseline to receive either a second L9LS injection or a placebo injection to evaluate the additional efficacy of a second dose administered 6 months after the first dose. (Those in the placebo arm will receive a second injection of placebo.) Participants will be followed for an additional 6

months after the second injection with monthly blood smear microscopy and PCR, and a blood draw at month 11 to assess L9LS PK.

Part 2 extension: Assuming there are no safety signals from the day 7 review following dosing children with 30 and 40 mg/kg of product, and prior to the last study visit of the original protocol described above (September 2022 to June 2024), study participants who remain enrolled in the part 2 efficacy study will be invited to participate in a 12-month extension of the study. A separate written consent process will take place. Enrolled children will continue in the same study groups as in part 2: 1 dose L9LS, 2 doses L9LS, or placebo. A dose (of L9LS or placebo) will be given at the start of the extension and again after six months. Those receiving L9LS will receive a dose between 20 and 40 mg/kg, still in a double-blinded fashion. The monthly visit and sample collection schedule will mirror that in part 2.

Objectives:

Primary Objectives:

Part 1:

1. Safety: To evaluate the safety and tolerability of L9LS administered at doses of 5 mg/kg, 10 mg/kg, and 20 mg/kg by SC administration to healthy Kenyan children.

Part 1b:

1. Safety: To evaluate the safety and tolerability of L9LS administered at doses of 30 mg/kg and 40 mg/kg by SC administration to healthy Kenyan children

Part 2:

1. Safety: To evaluate the safety and tolerability of L9LS administered at doses of 10-20 mg/kg by SC administration to healthy Kenyan children.
2. Efficacy: To assess the efficacy of two doses of L9LS at a concentration of 10-20 mg/kg, administered SC to participants aged 5-59 months of age against first/only Pf malaria infection diagnosed by blood smear microscopy (irrespective of fever) over 12 months compared to placebo.

Secondary Objectives:

1. To assess the efficacy of one dose of L9LS at 10-20 mg/kg against first/only Pf malaria infection diagnosed by blood smear microscopy over 3 and 6 months compared to placebo.
2. To assess the efficacy of one dose of L9LS at 10-20 mg/kg against first/only Pf malaria infection diagnosed by blood smear microscopy over 12 months compared to placebo.
3. To evaluate the efficacy of L9LS at 10-20 mg/kg against first/only Pf malaria infection as detected by PCR over 3, 6 and 12 months compared to placebo.
4. To assess protection of L9LS at 10-20 mg/kg against clinical malaria (first/only and all episodes) at 3, 6 and 12 months compared to a placebo.
5. To assess the primary objective and secondary objectives 1-4 among children 5-17 months of age.
6. To assess the primary objective and secondary objectives 1-4 among children 18-59 months of age.
7. To evaluate the PK of L9LS throughout the study at weight-based dose levels of 5 mg/kg, 10 mg/kg, and 20 mg/kg (part 1) and fixed doses of 10-20 mg/kg (part 2) in healthy Kenyan children, and
    a. To correlate L9LS serum concentration with Pf infection risk.
    b. To correlate L9LS serum concentration with clinical malaria risk.

Part 2 Extension Primary Objectives

1. To evaluate the safety and tolerability of a third and fourth SC dose of L9LS at 20–40 mg/kg (compared to placebo) in healthy Kenyan children.
2. To evaluate the PK of L9LS throughout the follow-up phase at 20–40 mg/kg in healthy Kenyan children.
3. To determine if ADAs to L9LS can be detected in sera of recipients at specific timepoints throughout the study and to correlate the occurrence of ADAs with L9LS PK.

Part 2 Extension Exploratory Objectives (see Figure 4 for study arm definitions):

1. To assess the efficacy of a third and fourth SC dose of L9LS over one year at 20–40 mg/kg in mediating protection against Pf infection and clinical malaria in healthy Kenyan children during a second year of follow-up compared to those who received placebo only (arms 1 vs. 3; arms 2 vs. 3) and in those receiving two L9LS doses in the extension versus one (arms 1 vs. 2)
2. To evaluate the PK of L9LS throughout the study and to correlate L9LS serum concentration with Pf infection risk and clinical malaria risk
3. To evaluate the effect of a third and fourth dose of L9LS on development of acquired immunity (measured by antimalarial antibodies)

Endpoints:

Primary Endpoints:

1. Part 1, part 1b, and part 2: Incidence and severity of local and systemic adverse events (AEs) occurring within 7 days after the administration of L9LS, and incidence of serious adverse events (SAEs) throughout the study period.
2. Part 2: Pf blood-stage infection as detected by microscopic examination of thick blood smear for 52 weeks after administration of L9LS or placebo.

Secondary Endpoints:

Data from one or both parts of this study will be used to assess the following endpoints:

1. Pf blood-stage infection as detected by microscopic examination of thick blood smear for 12 and 24 weeks after administration of L9LS or placebo.
2. Pf blood-stage infection as detected by RT-PCR for 12, 24 and 52 weeks after administration of L9LS or placebo.
3. Incidence of clinical malaria (definition 1, see section 0) for 12, 24 and 52 weeks after administration of L9LS or placebo.

4. Incidence of clinical malaria (definition 2, see section 0) for 12, 24 and 52 weeks after administration of L9LS or placebo.
5. Measurement of L9LS in sera of recipients (parts 1 and 2).
6. PK analysis of L9LS and the association of L9LS concentration with Pf infection risk.

Part 2 Extension Primary Endpoints:

1. Incidence and severity of local and systemic AEs occurring within 7 days after the administration of L9LS and incidence of serious adverse events (SAEs) throughout the study period.
2. Measurement of L9LS in sera of recipients.
3. Measurement of ADA in sera of recipients.

Part 2 Extension Exploratory Endpoints:

1. Pf blood-stage infection as detected by microscopic examination of thick blood smears obtained between 1 week and 52 weeks after administration of a third dose of L9LS or placebo.
2. Pf blood-stage infection as detected by RT-PCR from dried blood spots obtained between 1 week and 52 weeks after administration of a third dose of L9LS or placebo.
3. Incidence of clinical malaria (see section 0) between 1 week and 52 weeks after administration of a third dose of L9LS or placebo.
4. PK analysis of L9LS and the association of L9LS concentration with Pf infection risk.
5. PK analysis of L9LS and the association of L9LS concentration with clinical malaria risk.
6. Levels of naturally occurring antimalarial antibodies in participant blood at selected time points

Study Population: We will enroll a total of 396 healthy Kenyan children, including 72 ages 5 months to 10 years residing in western Kenya for part 1 and 324 ages 5-59 months for part 2.

	The year 2 follow-on study will enroll an additional 24 children into an initial safety study (part 1b) and then participants who are currently enrolled in part 2 of the study and agree to continue in the extension phase will be followed for an additional year.
**Phase:**	2
**Description of Sites/Facilities Enrolling Participants:**	Study activities will take place at the KEMRI clinical trials unit based in the Siaya County Referral Hospital, which is the largest hospital in Siaya County, western Kenya. In part 2 and the extension phase, study activities will also take place at Kogelo Dispensary, located approximately 12 km from Siaya County Referral Hospital.
**Description of Study Intervention:**	Participants in part 1 of this study will receive one or two SC injections for a total dose of 5, 10, or 20 mg/kg of L9LS or placebo. Participants in part 1b will receive one or two SC injections for a total dose of 30 or 40 mg/kg.<br><br>Participants in part 2 of this study will receive 10-20 mg/kg of L9LS or placebo as a single dose, given in 1 or 2 SC injections, depending on the total dose required. At 6 months after the initial injection, participants who received an initial dose of L9LS will either receive a second L9LS SC injection or a placebo injection. (Those in the placebo arm will receive a second injection of placebo.)<br>In the part 2 extension study, the investigational product will be administered as two SC injection(s) of either L9LS at a dose of 20–40 mg/kg or matching placebo at the beginning of year 2 extension and after 6 months.
**Study Duration:**	36 months (including extension phase).
**Participant Duration:**	3 months (part 1or 1b) or 13 months (part 2) or 26 months (part 2 plus extension phase).

Lay Summary

Malaria is a disease that affects many people in the world, more so in sub-Saharan Africa. It is caused by parasites carried by mosquitoes. When the mosquitoes bite people, the parasites can pass into their blood and cause illness, which if not treated in a timely manner or sufficiently, can cause severe illness or even death. In effect, despite there being medicines to cure malaria, it is still responsible for a huge number of admissions and deaths, especially in Africa. This informs the need to come up with new ways to reduce the impact of malaria, for example, through preventing people from getting malaria in the first place.

This is a research study to test an experimental drug, called L9LS, for malaria. L9LS is a type of drug called a monoclonal antibody. Antibodies are natural products made by the human body to fight infection by blocking germs such as bacteria and parasites. Monoclonal means that all the antibodies in the drug are exactly the same. The study drug stays in the blood for several months and blocks the parasite that causes malaria. We hope that, if enough of the drug is in the blood, it will prevent people from getting malaria.

The study aims to find out how safe the drug is and whether it prevents malaria and if so, to what extent. This study will be conducted in two parts. In the first part, involving 72 participants, the study will investigate how safe and well tolerated three different increasing doses of the study drug are by administering each dose to older children first (5-10 years of age) then to younger children (5-59 months of age) 1 week after each different dose, if it is well tolerated and safe in the older age group. The study will proceed to part 2 if the doses are safe and well tolerated in the younger age group, as determined by a data safety and monitoring board. The second part of the study will investigate whether the drug prevents malaria, and if so, to what extent. It will enroll an equal number of children aged between 5-17 months and those aged 18-59 months, totaling to about 324 participants. Each age category will further have 3 groups of children assigned by chance to each group. The first group will receive two doses of the study drug 6 months apart, the 2nd group will receive the study drug then placebo (saltwater/an inactive substance similar to study drug) 6 months after, and the 3rd group will receive two doses of the placebo 6 months part.

Other objectives which will be explored by the study include comparing the study drug level with the possibility of getting a malaria infection or detecting malaria parasites in the blood.

The study will take place at Siaya County Referral Hospital and Kogelo Dispensary, both in Siaya County, western Kenya. The study duration will be approximately 24 months, with a participant in Part 1 of the study taking 4-5 months and a participant in Part 2 of the study taking 13-14 months.

The study procedures in Part 1 will entail assessing participant eligibility, getting consent and enrollment into the study. On enrollment, participants will receive a standard dose of antimalarial followed by the study drug or placebo after 2 weeks. They will then have another 10 study visits

within a 3 months period to monitor various safety measures. The study procedures in Part 2 will entail assessing participant eligibility, getting consent and enrollment into the study. On enrollment, participants will receive a standard dose of antimalarial followed by the study drug or placebo after 2 weeks. They will then have a study visit every month up to end of month 6 when they will receive the 2nd dose of either the study drug or placebo. Other follow up visits will be conducted monthly until end of 12 months from the date of initial study drug or placebo dose. These follow-up visits will assess both safety and efficacy measures.

Given new data showing that a higher dose might be needed to prevent malaria, a follow-on study will be done with consenting participants currently enrolled in part 2 who will receive an additional one or two doses of the study drug at a higher dose. Prior to this, a follow-on safety study will be done to look at these higher doses of L9LS to ensure they are safe and well tolerated. As part of the safety study, 24 children will be randomized to receive L9LS or placebo. They will then have another 10 study visits within a 3 months period to monitor various safety measures. If no safety signals are noted, then participants finishing part 2 will be consented for the follow-on study.

For the follow-on study, participants who consent will be enrolled once they complete 12 months of follow-up for part 2. They will receive another antimalarial treatment course and two weeks later they will receive their third dose of either L9LS or placebo; six months later, they will receive a fourth dose of L9LS or placebo. Participants will get the same product (L9LS or placebo) that they received in part 2. They will continue to have monthly study visits until the end of the study (12 months following dose 3). These follow-up visits will assess both safety and efficacy measures.

The study will provide information on the safety and usefulness of L9LS as a therapy used in the prevention of malaria.

Schema

Figure 1. Enrollment and study flow schematics for part 1.

Figure 1a. Age de-escalation, dose-escalation enrollment schematic for part 1. [a]

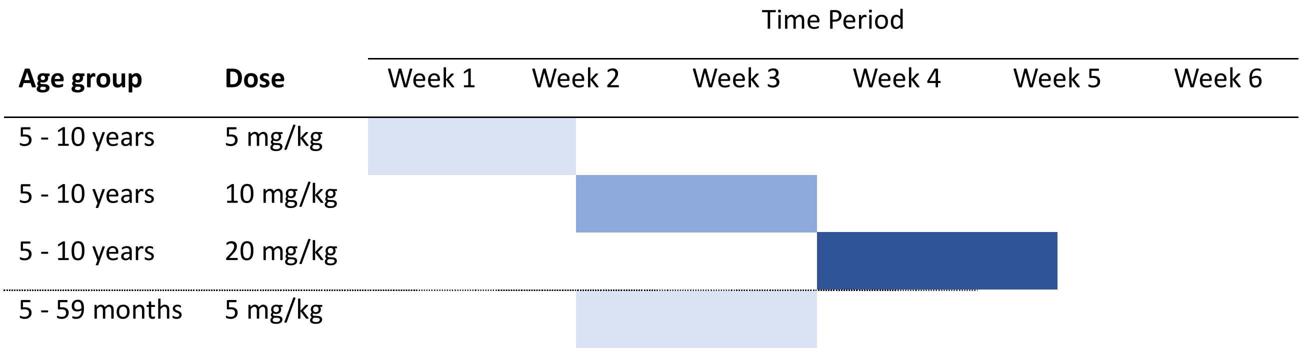

5 - 59 months	10 mg/kg
5 - 59 months	20 mg/kg

Note: n=12 per age/dose group (3 placebo, 9 L9LS)

[a] The table illustrates 1.5-week assessment periods prior to decision to proceed with enrollment into the next cohort; this allows all participants in the age/dose cohort to have their day 7 evaluation. All participants will be followed with safety assessments for a total of 3 months (see Figure 1b below).

Figure 1b. Study flow schematic for individual participants in part 1.

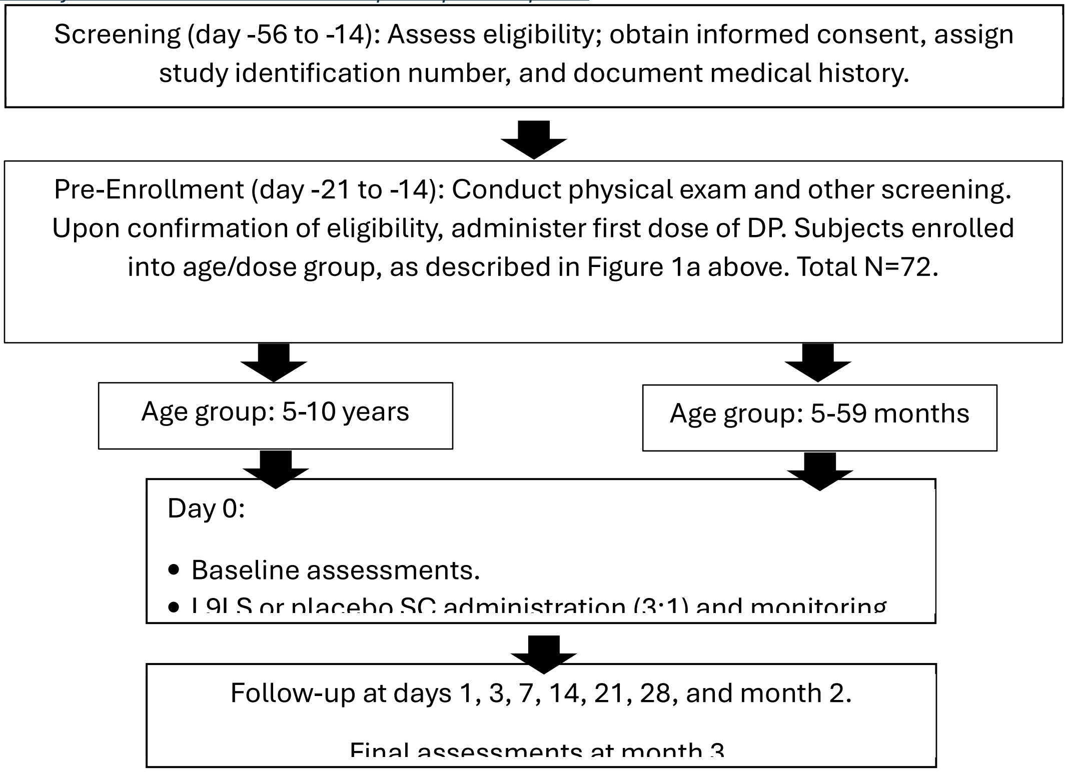

Figure 2. Study flow schematic for part 2.

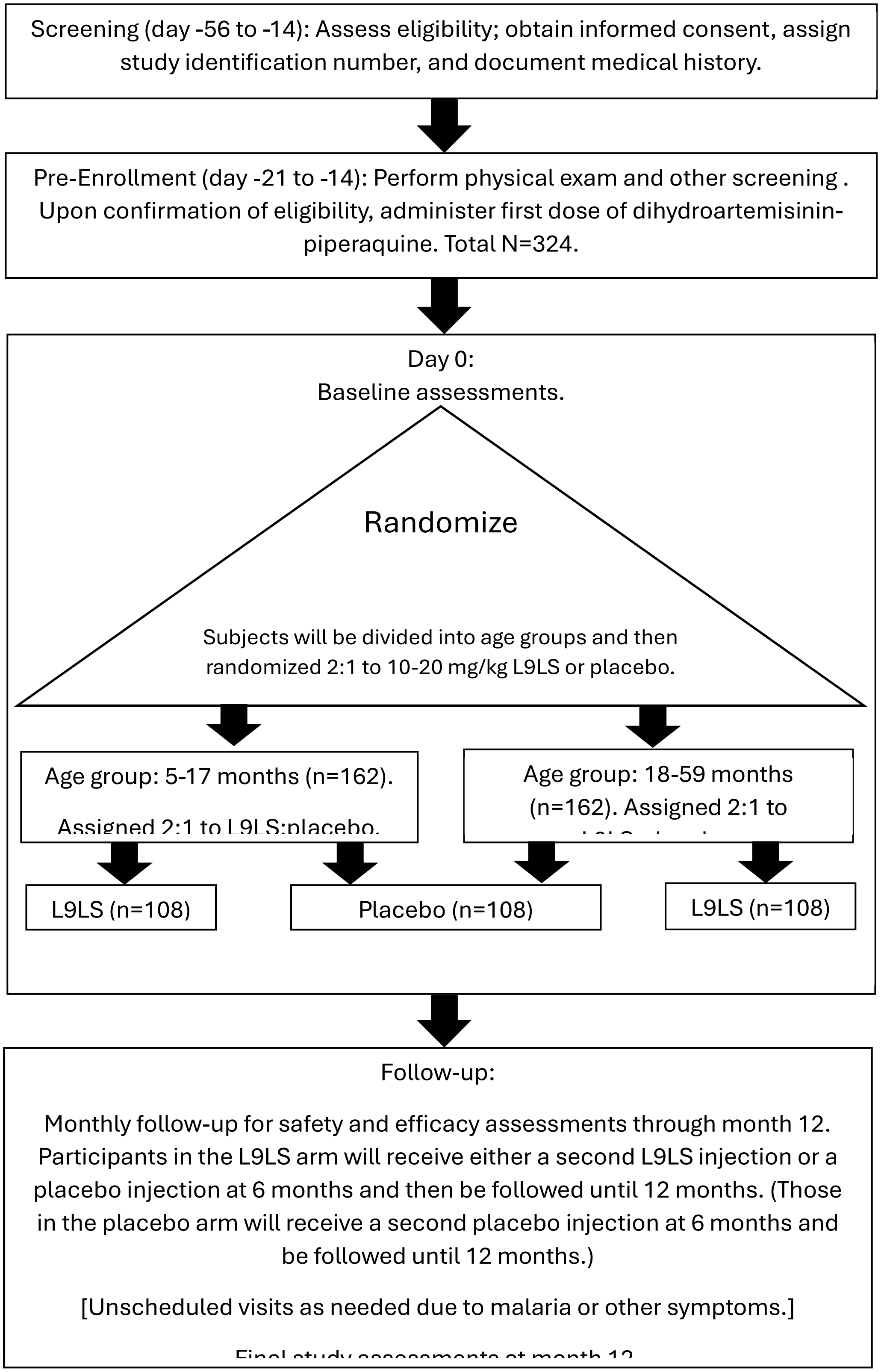

Figure 3. Study flow schematic for part 1b (additional safety study)

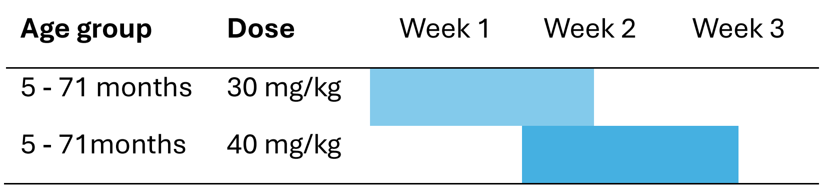

Figure 4. Study flow scheme for part 2 extension study

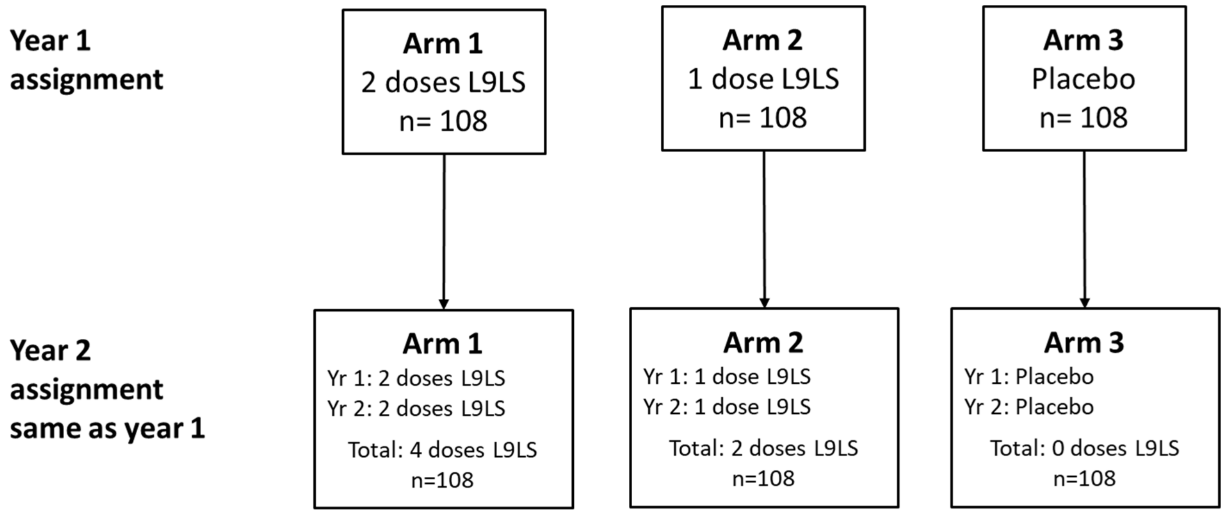

Schedule of Activities (SOA)

Part 1 Study Schedule

Study Timepoint	Screen [0]	Pre-Enrollment [0]	Enrolment (D0)	D1 [1]	D3 [1]	D7	D14	D21	D28	D56	D84	Unscheduled Visit [2]
Window (days)	-56 to -14	-21 to -14	-	+1	±1	±2	±3	±3	±7	±7	±7	
**Clinical Procedures/Evaluations**												
Study comprehension exam	X											
Informed consent	X											
Confirmation of eligibility		X										
Physical exam [3]		X	X			X	X	X	X	X	X	X
Height		X										
Weight		X	X								X	
Vital signs (temperature and pulse)		X	X			X	X	X	X	X	X	X
Medical history [3]	X	X	X	X	X	X	X	X	X	X	X	X
Concomitant medications [3]		X	X	X	X	X	X	X	X	X	X	X
Dihydroartemisinin-piperaquine administration		X [4]										
Randomization			X									
Study agent administration			X [5]									

Study Timepoint		Screen [0]	Pre-Enrollment [0]	Enrolment (D0)	D1 [1]	D3 [1]	D7	D14	D21	D28	D56	D84	Unscheduled Visit [2]
Window (days)		-56 to -14	-21 to -14	-	+1	±1	±2	±3	±3	±7	±7	±7	
Adverse event monitoring			X	X	X	X	X	X	X	X	X	X	
**Laboratory Evaluations**													
Test	Tube		(Blood volume in mL)										
HIV screen [6]	SST		0.2										
CBC with differential	EDTA		0.1				0.1	0.1		0.1		0.1	
Hemoglobin type	EDTA		0.5 [7]										
ALT, Cr	SST		0.2				0.2	0.2		0.2		0.2	
Blood smear and dried blood spot for Pf RT-PCR	-		0.4	0.4			0.4	0.4	0.4	0.4	0.4	0.4	0.4
PK studies	SST		3.1 [8]				3.1			3.1 [9]		3.1 [9]	
Serum storage	SST		(X)				(X)			(X)		(X)	
ADA	SST		(X)				(X)			(X)		(X)	
**Daily volume (mL)**		**-**	**4.5**	**0.4**	**-**	**-**	**3.8**	**0.7**	**0.4**	**3.8**	**0.4**	**3.8** [9]	
**Cumulative volume (mL)**		**-**	**4.5**	**4.9**	**4.9**	**4.9**	**8.7**	**9.4**	**9.8**	**13.6**	**14.0**	**14.7** [9]	**-**

Abbreviations: ADA, anti-drug antibodies; ALT, alanine transaminase; CBC, complete blood count; Cr, creatinine; D, day; EDTA, ethylenediaminetetraacetic acid; ELISA, enzyme-linked immunosorbent assay; HIV, human immunodeficiency virus; M, month; Pf, Plasmodium falciparum; PK, pharmacokinetics; SST, serum separating tube; RT-PCR, reverse transcription polymerase chain reaction.

(X) indicates that no additional blood will be drawn; the test will be performed from the tube shown in the preceding row.

Footnotes:

[0] Screening and pre-enrolment visits may be combined. Screening activities may be performed and/or repeated at pre-enrolment visit to ensure they are completed within the allowable window as described in section 8.1 (14 to 21 days prior to D0). As long as physical exam, height and weight measurement, vital signs, medical history, and concomitant medications are done at the pre-enrolment visit, they do not necessarily need to be done at the screening visit, if the two are separate visits.

[1] Days 1 and 3 will be home visits, with a phone call assessment if child is not able to be seen after 2 attempts.

[2] At any time during the study, the subject may have an unscheduled illness visit if experiencing malaria symptoms or other symptoms. The subject may be referred for standard care according to Kenya Ministry of Health treatment guidelines.

[3] Complete/comprehensive at screening; targeted/interim at other visits.

[4] The first dose of dihydroartemisinin-piperaquine will be directly observed in the clinic if possible, but might be given in the participant's home. The subsequent 2 doses given over the following 2 days will be observed by study staff in the subject's home or at the clinic. All dihydroartemisinin-piperaquine doses will be completed prior to day 0 and will be initiated between 21 and 14 days prior to D0.

[5] L9LS or placebo. All other study procedures must be completed prior to study agent administration. Each subject will be monitored for at least 60 minutes after study agent administration and vital signs will be recorded 1 hour after study agent administration. Prior to discharge, subject will be assessed for local reactogenicity (including pain/tenderness, swelling, redness, bruising, and pruritus at the site of injection) and systemic reactogenicity (including fever, feeling unusually tired or unwell, muscle aches, headache, chills, nausea, and joint pain). If the subject is assessed as being unwell or has ongoing reactogenicity symptoms, he or she will be asked to remain in the clinic until evaluation and discharge by a study clinician. Clinicians will follow any solicited symptoms that are ongoing until they have resolved.

[6] Viral screenings will be performed according to Kenyan MoH guidelines. HIV testing will be 2 rapid diagnostic tests (RDTs), plus DNA PCR if the RDTs are discordant; subject will be referred for medical care for 2 positive RDTs or a positive PCR. Pre- and post-test HIV counseling will be provided.

[7] EDTA tube will be stored at screening, and hemoglobin typing will be performed; in the rare case that hemoglobin typing detects sickle cell disease, the participant will be considered a late screening failure; sickle cell trait is not an exclusion criterion.

[8] For PK, 3.5 mL serum will be collected prior to malaria treatment administration.

[9] Half of the participants will have this blood draw on Day 28 and the other half will have it at Day 84. Accordingly, the total cumulative volume reflects a PK blood volume for Day 28 or Day 84, but not both.

Part 2 Study Schedule

Study Timepoint	Screen [0]	Pre-Enrollment [0]	Enrolment (D0)	D1 [1]	D3 [1]	D7	D28 (Wk 4)	D56 (Wk 8)	D84 (Wk 12)	D112 (Wk 16)	D140 (Wk 20)	D168 (Wk 24)*	D169 [1]	D171 [1]	D175	D196 (Wk 28)	D224 (Wk 32)	D252 (Wk 36)	D280 (Wk 40)	D308 (Wk 44)	D336 (Wk 48)	D364 (Wk 52)	Unscheduled
Window (days)	-56 to -14	-21 to -14	-	+1	±1	±1	±7	±7	±7	±7	±7	±7	+1	±1	±1	±7	±7	±7	±7	±7	±7	±7	
**Clinical Procedures/Evaluations**																							
Study comprehension exam	X																						
Informed consent	X																						
Confirmation of identity, age, and residency		X																					
Physical exam [3]		X	X			X	X	X	X	X	X	X			X	X	X	X	X	X	X	X	X
Height		X																					
Weight		X	X						X			X						X				X	
Vital signs (temperature and pulse)		X	X			X	X	X	X	X	X	X			X	X	X	X	X	X	X	X	X
Medical history [3]	X	X	X	X	X	X	X	X	X	X	X	X	X	X	X	X	X	X	X	X	X	X	X

**Study Timepoint**	**Screen[0]**	**Pre-Enrollment[0]**	**Enrolment (D0)**	**D1[1]**	**D3[1]**	**D7**	**D28 (Wk 4)**	**D56 (Wk 8)**	**D84 (Wk 12)**	**D112 (Wk 16)**	**D140 (Wk 20)**	**D168 (Wk 24)***	**D169[1]**	**D171[1]**	**D175**	**D196 (Wk 28)**	**D224 (Wk 32)**	**D252 (Wk 36)**	**D280 (Wk 40)**	**D308 (Wk 44)**	**D336 (Wk 48)**	**D364 (Wk 52)**	**Unscheduled**
Window (days)	-56 to -14	-21 to -14	-	+1	±1	±1	±7	±7	±7	±7	±7	±7	+1	±1	±1	±7	±7	±7	±7	±7	±7	±7	
Concomitant medications [3]		X	X	X	X	X	X	X	X	X	X	X	X	X	X	X	X	X	X	X	X	X	X
Dihydroartemisinin-piperaquine administration		X[4]																					
Randomization			X																				
Study agent administration			X[5]									X[5]											
Adverse events		X	X	X	X	X	X	X	X	X	X	X	X	X	X	X	X	X	X	X	X	X	

	**Laboratory Evaluations**																							
Test	Tube		(Blood volume in mL)																					
HIV[6]	SST		0.2																					
CBC with differential	EDTA		0.1	0.1			0.1	0.1					0.1			0.1						0.1		
Hemoglobin type	EDTA		0.5[7]																					
ALT, Cr	SST		0.2	0.2			0.2	0.2					0.2			0.2						0.2		

Study Timepoint		Screen[0]	Pre-Enrollment[0]	Enrolment (D0)	D1[1]	D3[1]	D7	D28 (Wk 4)	D56 (Wk 8)	D84 (Wk 12)	D112 (Wk 16)	D140 (Wk 20)	D168 (Wk 24)*	D169[1]	D171[1]	D175	D196 (Wk 28)	D224 (Wk 32)	D252 (Wk 36)	D280 (Wk 40)	D308 (Wk 44)	D336 (Wk 48)	D364 (Wk 52)	Unscheduled
Window (days)		-56 to -14	-21 to -14	-	+1	±1	±1	±7	±7	±7	±7	±7	±7	+1	±1	±1	±7	±7	±7	±7	±7	±7	±7	
Blood smear and dried blood spot for Pf RT-PCR	-		0.4	0.4[8]			0.4	0.4	0.4	0.4	0.4	0.4	0.4			0.4	0.4	0.4	0.4	0.4	0.4	0.4	0.4	0.4
PK studies	SST		3.1[9]					3.1									3.1					3.1		
Serum storage	SST		(X)					(X)									(X)					(X)		
ADA	SST		(X)					(X)									(X)					(X)		
**Daily volume (mL)**		**-**	**4.5**	**0.7**	**-**	**-**	**0.7**	**3.8**	**0.4**	**0.4**	**0.4**	**0.4**	**0.7**	**-**	**-**	**0.7**	**3.5**	**0.4**	**0.4**	**0.4**	**0.4**	**3.8**	**0.4**	
**Cumulative volume (mL)**		**-**	**4.5**	**5.2**	**5.2**	**5.2**	**5.9**	**9.7**	**10.1**	**10.5**	**10.9**	**11.3**	**12.0**	**12.0**	**12.0**	**12.7**	**16.2**	**16.6**	**17.0**	**17.4**	**17.8**	**21.6**	**22.0**	**-**

Abbreviations: ADA, anti-drug antibodies; ALT, alanine transaminase; CBC, complete blood count; Cr, creatinine; D, day; EDTA, ethylenediaminetetraacetic acid; HIV, human immunodeficiency virus; M, month; Pf, *Plasmodium falciparum*; PK, pharmacokinetics; SST, serum-separating tube; RT-PCR, reverse transcription polymerase chain reaction.

(X) indicates that no additional blood will be drawn; the test will be performed from the tube shown in the preceding row.

Footnotes:

[0] Screening and pre-enrolment visits may be combined. Screening activities may be performed and/or repeated at pre-enrolment visit to ensure they are completed within the allowable window as described in section 8.1 (14 to 21 days prior to D0). As long as physical exam, height and weight measurement, vital signs, medical history, and concomitant medications are done at the pre-enrolment visit, they do not necessarily need to be done at the screening visit, if the two are separate visits.

[1] Home-based visits.

[2] At any time during the study, the subject may have an unscheduled illness visit if experiencing malaria symptoms or other symptoms. The subject may be referred for standard care according to local guidelines.

[3] Complete/comprehensive at screening; targeted/interim at other visits.

[4] The first dose of dihydroartemisinin-piperaquine will be directly observed in the clinic if possible, but might be given in the participant's home. The subsequent 2 doses given over the following 2 days will be observed by guides in the subject's home or at the clinic. All dihydroartemisinin-piperaquine doses will be completed prior to day 0 and will be initiated between 21 and 14 days prior to D0.

[5] L9LS or placebo. All other study procedures must be completed prior to study agent administration. Subjects who received L9LS at day 0 will receive L9LS or placebo at month 6; those in the placebo arm will receive a second placebo injection at 6 months. Each subject will be monitored for at least 60 minutes after study agent administration and vital signs will be recorded 1 hour after study agent administration. Prior to discharge, subject will be assessed for local reactogenicity (including pain/tenderness, swelling, redness, bruising, and pruritus at the site of injection) and systemic reactogenicity (including fever, feeling unusually tired or unwell, muscle aches, headache, chills, nausea, and joint pain). If the subject is assessed as being unwell or has ongoing reactogenicity symptoms, he or she will be asked to remain in the clinic until evaluation and discharge by a study clinician. Clinicians will follow any solicited symptoms that are ongoing until they have resolved.

[6] Viral screenings will be performed according to international guidelines. HIV testing will be 2 rapid diagnostic tests (RDTs), plus DNA PCR if the RDTs are discordant; subject will be referred for medical care for 2 positive RDTs or a positive PCR. Pre- and post-test HIV counseling will be provided.

[7] EDTA tube will be stored at screening, and hemoglobin typing will be performed; in the rare case that hemoglobin typing detects sickle cell disease, the participant will be considered a late screening failure; sickle cell trait is not an exclusion criterion.

[8] Collected prior to study agent administration. Other indicated tests may be performed after study agent administration.

[9] For PK, 3.5 mL serum will be collected prior to malaria treatment administration.

* The timing of all visits subsequent to Day 168 (administration of dose 2) will be in relation to the Day 168 visit, whenever that occurs; for example, if the Day 168 visit occurs on Day 161, the first day of the window, the Days 169 and 171 home visits will occur 1 and 3 days later (so on days 162 and 164 in relation to dose 1).

Part 1b Study Schedule

Study Timepoint	Screen [0]	Pre-Enrollment [0]	Enrolment (D0)	D1 [1]	D3 [1]	D7	D14	D21	D28	D56	D84	Unscheduled Visit [2]
Window (days)	-56 to -14	-21 to -14	-	+1	±1	±2	±3	±3	±7	±7	±7	
**Clinical Procedures/Evaluations**												
Study comprehension exam	X											
Informed consent	X											
Confirmation of eligibility		X										
Physical exam [3]		X	X			X	X	X	X	X	X	X
Height		X										
Weight		X	X								X	
Vital signs (temperature and pulse)		X	X			X	X	X	X	X	X	X
Medical history [3]	X	X	X	X	X	X	X	X	X	X	X	X
Concomitant medications [3]		X	X	X	X	X	X	X	X	X	X	X
Dihydroartemisinin-piperaquine administration		X [4]										
Randomization			X									
Study agent administration			X [5]									
Adverse event monitoring		X	X	X	X	X	X	X	X	X	X	

Study Timepoint		Screen [0]	Pre-Enrollment [0]	Enrolment (D0)	D1 [1]	D3 [1]	D7	D14	D21	D28	D56	D84	Unscheduled Visit [2]
Window (days)		-56 to -14	-21 to -14	-	+1	±1	±2	±3	±3	±7	±7	±7	
**Laboratory Evaluations**													
Test	Tube		(Blood volume in mL)										
HIV screen [6]	SST		0.2										
CBC with differential	EDTA		0.1				0.1	0.1		0.1		0.1	
Hemoglobin type	EDTA		0.5 [7]										
ALT, Cr	SST		0.2				0.2	0.2		0.2		0.2	
Blood smear and dried blood spot for Pf RT-PCR	-		0.4	0.4			0.4	0.4	0.4	0.4	0.4	0.4	0.4
PK studies	SST		3.1 [8]				3.1			3.1 [9]		3.1 [9]	
Serum storage	SST		(X)				(X)			(X)		(X)	
ADA	SST		(X)				(X)			(X)		(X)	
**Daily volume (mL)**		**-**	**4.5**	**0.4**	**-**	**-**	**3.8**	**0.7**	**0.4**	**3.8**	**0.4**	**3.8** [9]	
**Cumulative volume (mL)**		**-**	**4.5**	**4.9**	**4.9**	**4.9**	**8.7**	**9.4**	**9.8**	**13.6**	**14.0**	**14.7** [9]	**-**

Abbreviations: ADA, anti-drug antibodies; ALT, alanine transaminase; CBC, complete blood count; Cr, creatinine; D, day; EDTA, ethylenediaminetetraacetic acid; ELISA, enzyme-linked immunosorbent assay; HIV, human immunodeficiency virus; M, month; Pf, Plasmodium falciparum; PK, pharmacokinetics; SST, serum separating tube; RT-PCR, reverse transcription polymerase chain reaction.

(X) indicates that no additional blood will be drawn; the test will be performed from the tube shown in the preceding row.

Footnotes:

[0] Screening and pre-enrolment visits may be combined. Screening activities may be performed and/or repeated at pre-enrolment visit to ensure they are completed within the allowable window as described in section 8.1 (14 to 21 days prior to D0). As long as physical exam, height and weight measurement, vital signs, medical history, and concomitant medications are done at the pre-enrolment visit, they do not necessarily need to be done at the screening visit, if the two are separate visits.

[1] Days 1 and 3 will be home visits, with a phone call assessment if child is not able to be seen after 2 attempts.

[2] At any time during the study, the subject may have an unscheduled illness visit if experiencing malaria symptoms or other symptoms. The subject may be referred for standard care according to Kenya Ministry of Health treatment guidelines.

[3] Complete/comprehensive at screening; targeted/interim at other visits.

[4] The first dose of dihydroartemisinin-piperaquine will be directly observed in the clinic if possible, but might be given in the participant's home. The subsequent 2 doses given over the following 2 days will be observed by study staff in the subject's home or at the clinic. All dihydroartemisinin-piperaquine doses will be completed prior to day 0 and will be initiated between 21 and 14 days prior to D0.

[5] L9LS or placebo. All other study procedures must be completed prior to study agent administration. Each subject will be monitored for at least 60 minutes after study agent administration and vital signs will be recorded 1 hour after study agent administration. Prior to discharge, subject will be assessed for local reactogenicity (including pain/tenderness, swelling, redness, bruising, and pruritus at the site of injection) and systemic reactogenicity (including fever, feeling unusually tired or unwell, muscle aches, headache, chills, nausea, and joint pain). If the subject is assessed as being unwell or has ongoing reactogenicity symptoms, he or she will be asked to remain in the clinic until evaluation and discharge by a study clinician. Clinicians will follow any solicited symptoms that are ongoing until they have resolved.

[6] Viral screenings will be performed according to Kenyan MoH guidelines. HIV testing will be 2 rapid diagnostic tests (RDTs), plus DNA PCR if the RDTs are discordant; subject will be referred for medical care for 2 positive RDTs or a positive PCR. Pre- and post-test HIV counseling will be provided.

[7] EDTA tube will be stored at screening, and hemoglobin typing will be performed; in the rare case that hemoglobin typing detects sickle cell disease, the participant will be considered a late screening failure; sickle cell trait is not an exclusion criterion.

[8] For PK, 3.5 mL serum will be collected prior to malaria treatment administration.

[9] Half of the participants will have this blood draw on Day 28 and the other half will have it at Day 84. Accordingly, the total cumulative volume reflects a PK blood volume for Day 28 or Day 84, but not both.

Part 2 Extension Study Schedule

Study Timepoint	Consent for Extension	Pre-Enrolment	Enrolment (D0)	Y2D1[1]	Y2D3[1]	Y2D7	Y2D28 (Wk 4)	Y2D56 (Wk 8)	Y2D84 (Wk 12)	Y2D112 (Wk 16)	Y2D140 (Wk 20)	Y2D168 (Wk 24)*	Y2D169[1]	Y2D171[1]	Y2D175	Y2D196 (Wk 28)	Y2D224 (Wk 32)	Y2D252 (Wk 36)	Y2D280 (Wk 40)	Y2D308 (Wk 44)	Y2D336 (Wk 48)	Y2D364 (Wk 52)	Unscheduled
Window (days)	Before Parasit Clearance[0]	-21 to -14	-	+1	±1	±1	±7	±7	±7	±7	±7	±7	+1	±1	±1	±7	±7	±7	±7	±7	±7	±7	
**Clinical Procedures/Evaluations**																							
Study comprehension exam	X																						
Informed consent	X																						
Confirmation of identity, age, and residency	X																						
Physical exam		X	X			X	X	X	X	X	X	X			X	X	X	X	X	X	X	X	X
Height		X																					
Weight		X	X						X			X						X				X	
Vital signs (temperature, pulse)		X	X			X	X	X	X	X	X	X			X	X	X	X	X	X	X	X	X
Medical history	X	X	X	X	X	X	X	X	X	X	X	X	X	X	X	X	X	X	X	X	X	X	X

Study Timepoint	Consent for Extension	Pre-Enrolment	Enrolment (D0)	Y2D1[1]	Y2D3[1]	Y2D7	Y2D28 (Wk 4)	Y2D56 (Wk 8)	Y2D84 (Wk 12)	Y2D112 (Wk 16)	Y2D140 (Wk 20)	Y2D168 (Wk 24)*	Y2D169[1]	Y2D171[1]	Y2D175	Y2D196 (Wk 28)	Y2D224 (Wk 32)	Y2D252 (Wk 36)	Y2D280 (Wk 40)	Y2D308 (Wk 44)	Y2D336 (Wk 48)	Y2D364 (Wk 52)	Unscheduled
Window (days)	Before Parasit Clearance[0]	-21 to -14	-	+1	±1	±1	±7	±7	±7	±7	±7	±7	+1	±1	±1	±7	±7	±7	±7	±7	±7	±7	
Concomitant medications		X	X	X	X	X	X	X	X	X	X	X	X	X	X	X	X	X	X	X	X	X	X
Dihydroartemisinin-piperaquine administration		X[4]																					
Randomization			X																				
Study agent administration			X[5]									X[5]											
Adverse events		X	X	X	X	X	X	X	X	X	X	X	X	X	X	X	X	X	X	X	X	X	

Laboratory Evaluations

Test	Tube		Pre-Enrolment	Enrolment (D0)	Y2D1	Y2D3	Y2D7	Y2D28	Y2D56	Y2D84	Y2D112	Y2D140	Y2D168	Y2D169	Y2D171	Y2D175	Y2D196	Y2D224	Y2D252	Y2D280	Y2D308	Y2D336	Y2D364	Unscheduled
			(Blood volume in mL)																					
CBC with differential	EDTA		0.1	0.1			0.1	0.1					0.1			0.1						0.1		
ALT, Cr	SST		0.2	0.2			0.2	0.2					0.2			0.2						0.2		
Blood smear and dried	-		0.4	0.4[8]			0.4	0.4	0.4	0.4	0.4	0.4	0.4			0.4	0.4	0.4	0.4	0.4	0.4	0.4	0.4	0.4

**Study Timepoint**		**Consent for Extension**	**Pre-Enrolment**	**Enrolment (D0)**	**Y2D1[1]**	**Y2D3[1]**	**Y2D7**	**Y2D28 (Wk 4)**	**Y2D56 (Wk 8)**	**Y2D84 (Wk 12)**	**Y2D112 (Wk 16)**	**Y2D140 (Wk 20)**	**Y2D168 (Wk 24)***	**Y2D169[1]**	**Y2D171[1]**	**Y2D175**	**Y2D196 (Wk 28)**	**Y2D224 (Wk 32)**	**Y2D252 (Wk 36)**	**Y2D280 (Wk 40)**	**Y2D308 (Wk 44)**	**Y2D336 (Wk 48)**	**Y2D364 (Wk 52)**	**Unscheduled**
Window (days)		Before Parasit Clearance[0]	-21 to -14	-	+1	±1	±1	±7	±7	±7	±7	±7	±7	+1	±1	±1	±7	±7	±7	±7	±7	±7	±7	
blood spot for Pf RT-PCR																								
PK studies	SST		3.1[9]					3.1									3.1					3.1		
Serum storage	SST		(X)					(X)									(X)					(X)		
ADA	SST		(X)					(X)									(X)					(X)		
**Daily volume (mL)**		**-**	**3.8**	**0.7**	**-**	**-**	**0.7**	**3.8**	**0.4**	**0.4**	**0.4**	**0.4**	**0.7**	**-**	**-**	**0.7**	**3.5**	**0.4**	**0.4**	**0.4**	**0.4**	**3.8**	**0.4**	
**Cumulative volume (mL)**		**-**	**3.8**	**4.5**	**4.5**	**4.5**	**5.2**	**9.0**	**9.4**	**9.8**	**10.2**	**10.6**	**11.3**	**11.3**	**11.3**	**12.0**	**15.5**	**15.9**	**16.3**	**16.7**	**17.1**	**20.9**	**21.3**	**-**

Abbreviations: ADA, anti-drug antibodies; ALT, alanine transaminase; CBC, complete blood count; Cr, creatinine; D, day; EDTA, ethylenediaminetetraacetic acid; HIV, human immunodeficiency virus; M, month; Pf, *Plasmodium falciparum*; PK, pharmacokinetics; SST, serum-separating tube; RT-PCR, reverse transcription polymerase chain reaction.

(X) indicates that no additional blood will be drawn; the test will be performed from the tube shown in the preceding row.

Footnotes:

[0] The Consent for the extension phase visit can take place during the Day 364 close-out visit for part 2 or thereafter anytime before the extension phase parasite clearance visit. The extension phase pre-enrolment visit must take place within 3 months of the (year 1) Day 364 visit.

[1] Home-based visits.

[2] At any time during the study, the subject may have an unscheduled illness visit if experiencing malaria symptoms or other symptoms. The subject may be referred for standard care according to local guidelines.

[4] The first dose of dihydroartemisinin-piperaquine will be directly observed in the clinic if possible, but might be given in the participant's home. The subsequent 2 doses given over the following 2 days will be observed by guides in the subject's home or at the clinic. All dihydroartemisinin-piperaquine doses will be completed prior to day 0 and will be initiated between 21 and 14 days prior to D0.

[5] L9LS or placebo. All other study procedures must be completed prior to study agent administration. Subjects who received L9LS at day 0 will receive L9LS at month 6; those in the placebo arm will receive a second placebo injection at 6 months (if a two-dose regimen is used). Each subject will be monitored for at least 60 minutes after study agent administration and vital signs will be recorded 1 hour after study agent administration. Prior to discharge, subject will be assessed for local reactogenicity (including pain/tenderness, swelling, redness, bruising, and pruritus at the site of injection) and systemic reactogenicity (including fever, feeling unusually tired or unwell, muscle aches, headache, chills, nausea, and joint pain). If the subject is assessed as being unwell or has ongoing reactogenicity symptoms, he or she will be asked to remain in the clinic until evaluation and discharge by a study clinician. Clinicians will follow any solicited symptoms that are ongoing until they have resolved.

[8] Collected prior to study agent administration. Other indicated tests may be performed after study agent administration.

[9] For PK, 3.5 mL serum will be collected prior to malaria treatment administration.

* The timing of all visits subsequent to Day 168 (administration of dose 2) will be in relation to the Day 168 visit, whenever that occurs; for example, if the Day 168 visit occurs on Day 161, the first day of the window, the Days 169 and 171 home visits will occur 1 and 3 days later (so on days 162 and 164 in relation to dose 1).

INTRODUCTION

Study Rationale

Malaria is a mosquito-borne disease caused by a protozoan belonging to the genus *Plasmodium* that affected approximately 229 million people and killed approximately 409,000 individuals in 2019, with an enormous economic impact in the developing world, especially sub-Saharan Africa. [31111 1 1 11] The 5 recognized species of *Plasmodium* that cause human malaria infection are *Plasmodium falciparum, P. vivax*, *P. ovale*, *P. malariae*, and *P. knowlesi*. Among these, *P. falciparum* (Pf) causes more deaths in children worldwide than any other single infectious agent. An estimated 30,000 travelers from North America, Europe, and Japan contract malaria per year. Although malaria is preventable with chemoprophylaxis and completely curable with early intervention, drug treatment is not readily accessible in many parts of the world. Additionally, the use of antimalarial drugs over time has been associated with the emergence of drug-resistant strains. Lack of compliance with preventive drug treatment by individuals travelling to endemic areas may also result in fatal malaria infection. The world's first malaria vaccine, RTS,S/AS01 (Mosquirix™), a recombinant protein-based vaccine targeting Pf, was approved for use by European regulatory authorities in 2015. RTS,S/AS01 is currently being evaluated in an ongoing pilot immunization program in sub-Saharan Africa despite having been found to provide partial protection against malaria to children and infants.[4,5] Based on results from the ongoing pilot program, the World Health Organization (WHO) recently recommended widespread use of RTS,S/AS01 in a schedule of 4 doses in infants from 5 months of age in sub-Saharan Africa and in other regions with moderate to high Pf malaria transmission. [64] Therefore, based on the ~40% efficacy of RTS,S/AS01 after 4 years, there is an urgent need for the development of safe and more effective malaria vaccines and other novel interventions. Importantly, the use of monoclonal antibodies (MAbs) offers a new modality for mediating prevention of malaria to limit the morbidity and mortality in infants and young children.

Background

The WHO has set a target for the development of a malaria vaccine efficacy (VE) with at least 75% VE against clinical malaria by 2030. Currently, RTS,S/AS01 is the only malaria vaccine for which phase 3 trials have been completed. It was found to have VE in children aged 5-17 months against clinical malaria (36.3%) and severe malaria (32.2%) after 4 years of follow-up; [42] the vaccine had 60.1% efficacy against all episodes of clinical malaria after 6 months of follow-up. [75] More recently, in a Phase 2 trial, the R21/MM vaccine had 77% vaccine efficacy against the first/only episode of clinical malaria at 6 months. The efficacy remained at this level over 1 year; however, there were very few cases of malaria after 6 months in this study given the highly seasonal malaria transmission in Burkina Faso, so it remains unclear whether this vaccine would achieve the targeted level of protection over longer periods of time or in settings of perennial transmission. RTS,S/AS01 has been recently recommended for widespread use in infants as a first step toward reducing clinical infection. Importantly, there is now focus on second-generation vaccines or other immune interventions that may enhance protective efficacy. An intervention that could prevent malaria in children for a full 12 months in settings of perennial malaria transmission, such as Kenya, holds potential to reduce malaria morbidity and mortality substantially in this population at risk for severe malaria and death. If one year of protection can be achieved with a single dose of

MAb, this has important implications for feasibility, acceptability, and cost of the intervention.[8,9] In addition, children discharged after hospitalization with severe anemia have been shown to have high rates of subsequent morbidity and mortality in the immediate post-discharge period; administering a dose of MAb prior to discharge could help prevent poor outcomes in these children, possibly with better outcomes than was recently obtained with chemoprophylaxis. [108888 8 8 88] Another group to consider would be children with sickle cell disease as a replacement for their ongoing malaria chemoprophylaxis.

Recent evidence has shown that a non-vaccine biologic MAb, specifically CIS43, against a conserved region on the Pf circumsporozoite protein (PfCSP) which covers the infecting sporozoite, can prevent malaria infection.[11,12] A phase 1 trial in US adults showed that CIS43LS, a version of CIS43 modified to extend half-life, was safe, well tolerated, and conferred complete protection against controlled human malaria infection (CHMI) by mosquito bite in all nine participants. [13111111 11 11 1111] It was notable that 2 of the participants were protected 9 months after receiving CIS43LS following CHMI. These data provided the first proof-of-concept that MAbs can be used to prevent malaria infection with a single administration. Based on these promising findings in terms of safety and efficacy, an ongoing placebo-controlled phase 2 study of CIS43LS mAb in a total of 330 adult volunteers in Mali is assessing its safety and efficacy against high-level transmission during the rainy season. That study in Mali will assess 2 doses of CIS43LS administered intravenously (IV) compared to placebo and should provide the first evidence of efficacy in the field against natural exposure.

MAbs have a number of potential advantages over malaria vaccines: 1) MAb provide a consistent concentration of a highly potent antibody against a conserved site on the major sporozoite protein (PfCSP), independent of host/parasite factors that could alter vaccine immune responses, 2) they could confer protection against malaria after a single dose by preventing infection, while all malaria vaccines to date have required a minimum of 3 doses to limit clinical malaria; 3) MAbs are hypothesized to provide higher efficacy compared to malaria vaccines (26% of the children vaccinated with R21/MM were infected after 6 months); and 4) they are likely to be better tolerated, as MAbs do not contain adjuvants and are not meant to stimulate an immune response.

An important aspect of using a MAb for prevention of malaria is the potency and durability. Improving the potency will be critical for improving protection, reducing cost as well as allow for sub-cutaneous (SC) dosing across all ages. Accordingly, a second-generation MAb, L9, was recently discovered. [14122122 12 12 1212] L9 binds to a different region of the sporozoite than CIS43 and is approximately 2- to 3-fold more potent for mediating protection in 2 mouse models of malaria infection. L9 mediates protection through a distinct mechanism from CIS43. Thus, clinical development of L9LS was initiated as described below. As part of this development, we propose a 3-part study of L9LS in children in western Kenya to assess safety and efficacy in a phase 2 trial.

Study Agent: L9LS

Development

The Vaccine Research Center (VRC) of the National Institute for Allergies and Infectious Diseases (NIAID), NIH, has developed a MAb called L9LS as a possible preventive therapeutic against Pf infection. L9LS represents a second-generation anti-malaria MAb that follows CIS43LS, which was also developed by the VRC. As described above, CIS43LS was shown to be safe and conferred complete protection against controlled Pf infection in a phase 1 study, [13111111 11 11 1111] and is currently being evaluated in an ongoing phase 2 study in Mali (ClinicalTrials.gov Identifier: NCT04329104; FMOS/FAPH Protocol Number: 2020/32/CE/FMOS/FAPH).

L9, the wild-type parent of L9LS, was isolated by sorting the PfCSP-reactive memory B cells obtained from a subject immunized with a radiation-attenuated Pf whole-sporozoite malaria vaccine in the VRC 314 clinical trial (NCT02015091) using a junctional epitope mimic probe designed to select for “CIS43-like” MAbs. Epitope mapping showed L9 bound to the sporozoite NPNV motifs associated with NVDP minor repeats of PfCSP and this defined a new site of neutralization on PfCSP. When compared to a published large panel of the most protective human PfCSP MAbs, L9 protected mice against intravenous (IV) and mosquito bite sporozoite challenge, and demonstrated the lowest effective dose (ED)80 and effective concentration (EC)80 values (325.7 µg and 145.1 µg/mL, respectively) of any human PfCSP MAbs evaluated, including CIS43LS (685.92 µg and 363.93 µg/mL, respectively). [14122122 12 12 1212] The unique preference of L9 for NPNV motifs was further underscored by the fact that all four NVDP tetrapeptide motifs had to be mutated to NANP to disrupt the recognition of L9 for the NPNV motifs found in recombinant PfCSP. As 100% of known Pf field isolates have one or more NVDP motifs, these data suggest that L9 should bind all circulating strains of Pf malaria. [14122122 12 12 1212]

Half-life extension of L9 was accomplished by modifying the L9 Fc heavy chain to include an LS mutation (L9LS). The LS mutation is a methionine to leucine (L) and asparagine to serine (S) (M432L/N434S, collectively, LS) replacement, and changes have been reported to increase antibody Fc region binding affinity to FcRn, resulting in the extended recirculation of functional IgG and consequent longer antibody serum half-life. Other than these two amino acid sequences in the heavy chain Fc, the sequences of L9LS and L9 are identical. Immune protection and half-life data acquired with L9 and L9LS identify L9LS as a promising clinical candidate for passive malaria prophylaxis. Of note, LS mutations have been used for half-life extension of a large number of HIV MAbs tested in humans, and 2 complement-inhibitor MAbs that contain LS mutations are approved for human use for the treatment of patients with paroxysmal nocturnal hemoglobinuria to reduce hemolysis and patients with atypical hemolytic uremic syndrome to inhibit complement-mediated thrombotic microangiopathy, as well as other indications.[15,16] Thus, in all published studies, LS mutations have been safe and well tolerated.

Preclinical Experience

To assess L9LS as a candidate for clinical trials, research-grade MAb was evaluated for *in vitro* functional activity including binding properties, auto-reactivity, and PK, and in mouse models of *in*

vivo protection following challenge. In addition, PK studies in non-human primates (NHPs) were performed. In mice, L9 was more potent than CIS43, and both L9LS and L9 mediated the same potency following challenge. In NHP PK studies, L9LS exhibited significantly longer half-life in blood as compared to the parental L9 MAb without the LS mutation.

Two preclinical toxicology studies were conducted with a process-representative developmental batch of L9LS. An *in vitro* tissue cross-reactivity (TCR) assay to screen for potential cross-reactivity and an *in vivo* rat toxicity study to demonstrate safety both were conducted in compliance with 21 CFR 58 Good Laboratory Practice (GLP) for Nonclinical Laboratory Studies.

TCR Study

The TCR assay screened a standard panel of normal human tissues (three donors per tissue) and Sprague Dawley rat tissues (two rats per tissue). Mammalian cells transfected to express CSP were used as a tissue-positive control. Two concentrations of L9LS were tested: 1.15 μg/mL (selected as the concentration which saturated the positive control tissue), and 11.5 μg/mL (10-fold excess). A negative control IgG1κ antibody (GR338422-1, no mammalian target antigen) was tested at the same two concentrations. L9LS exhibited scattered specific membrane binding in 3/3 human salivary gland tissue samples and localized to the epithelial cells lining the ducts and acini. Binding was rare (1-5% of these epithelial cells) at 1.15 μg/mL, and rare to occasional (5-25% of these epithelial cells) at 11.5 μg/mL. The cause of the salivary epithelial cell membrane binding has not been identified. Specific membrane binding to salivary gland tissue was not observed for the negative control antibody. L9LS did not bind to rat salivary epithelium. No specific membrane binding to other human tissues was observed.

Rat Toxicity Study

Sprague Dawley rats were dosed with L9LS to evaluate toxicity and toxicokinetics. Rats received 0, 40, or 400 mg/kg by IV bolus injection twice (Day 1 and Day 11). Female rats were dosed with 10 mg/kg by SC injection once (D1); male rats were dosed with 10 mg/kg SC twice (D1 and D11); both male and female rats were dosed with 100 mg/kg SC twice (Day 1 and Day 11). For all dose levels, the main group was necropsied at Day 12 to evaluate potential immediate effects, and recovery animals were necropsied at Day 46, to evaluate the potential for delayed effects and recovery. Treated rats exhibited a transient increase in body temperature post-dose (up to + 0.5°C, considered a non-adverse response). Serum clinical chemistry tests detected slightly increased globulin, consistent with the administration of L9LS, an IgG MAb. For IV dosing, the no observed adverse effect level (NOAEL) was the high dose, 400 mg/kg IV x2. For SC dosing, the NOAEL was the high-dose, 100 mg/kg SC x2. SC injection of L9LS did not cause reactogenicity (edema, erythema, eschar) in any rat. Histopathology evaluation of the skin at the SC injection site reported minimal to moderate SC mixed cell infiltration, considered treatment related and predictive for human volunteers. One treated rat (in the 100 mg/kg SC group) was found dead; this event was attributed to a procedural error the previous day and is not considered treatment related. One treated female (in the 100 mg/kg SC recovery group) had grossly visible heart enlargement (2-fold increase in both heart weight and heart-body weight compared to the control means), with normal heart histology. Spontaneous cardiomyopathies are occasionally observed for the Sprague Dawley strain of rat. Based on the singular incidence, this observation is not considered treatment related.

With reference to the US Food and Drug Administration (FDA) Center for Drug Evaluation and Research 2005 Guidance for Industry Estimating the Maximum Safe Starting Dose in Initial Clinical Trials for Therapeutics in Adult Healthy Volunteers, scaling based on body weight is appropriate for a MAb expected to distribute mainly in the vascular space. The rat IV NOAEL of 400 mg/kg x2 supports the IV clinical high dose with a 20-fold dose margin. The rat SC NOAEL of 100 mg/kg x2 supports the SC clinical dose with a 20-fold dose margin.

The FDA provides guidance on “the nonclinical safety studies recommended to support human clinical trials” in the Guidances for Industry ICH M3(R2) Nonclinical Safety Studies for the Conduct of Human Clinical Trials and Marketing Authorization for Pharmaceuticals and ICH S6 Addendum to Preclinical Safety Evaluation of Biotechnology-Derived Pharmaceuticals. Taken together, the L9LS TCR and rat toxicity study results meet the safety standard set in these guidances and support proceeding with L9LS clinical trials.

Clinical Experience

L9LS was evaluated in a phase 1, dose-escalation, open-label clinical trial with experimental CHMI to evaluate safety and protective efficacy of L9LS in healthy, malaria-naive adults at the VRC. The phase 1 trial at the VRC was a two-part, dose-escalation, adaptive-design study evaluating the safety, tolerability, PK, and protective efficacy of L9LS.

Doses evaluated ranged from 1 to 20 mg/kg delivered via IV administration, and 5 mg/kg delivered via SC administration. No safety concerns were identified. A total of 17 L9LS recipients and 6 control participants underwent CHMI. Of the 17 participants who received a single dose of L9LS, 15 (88%) were protected after CHMI. Parasitemia did not develop in any of the participants who received 5 or 20 mg/kg of IV L9LS. Parasitemia developed in 1 of 5 participants who received 1 mg/kg IV, 1 of 5 participants who received 5 mg/kg SC, and all 6 control participants through 21 days after CHMI. Protection conferred by L9LS was seen at serum concentrations as low as 9.2 µg/mL. Thus, in this small phase 1 trial, L9LS administered IV or SC protected recipients against malaria after controlled infection, without evident safety concerns, and all IV and SC administrations were well tolerated. [1715]

For the first proof-of-concept study with CIS43LS in Malian adults, CIS43LS was administered by the IV route at doses of 40 mg/kg and 10 mg/kg. The first generation anti-malaria MAb CIS43LS was shown to be safe and conferred complete protection against controlled Pf infection in a phase 1 study in healthy adult volunteers at the VRC. [1311] CIS43LS was evaluated in a completed phase 2 study of adults in Mali (ClinicalTrials.gov Identifier: NCT04329104). For the first proof-of-concept study with CIS43LS in Malian adults against intense seasonal transmission, CIS43LS was administered by the IV route at doses of 40 mg/kg and 10 mg/kg. No significant safety concerns were noted. By time-to-event analysis, compared to placebo at 6 months, CIS43LS efficacy was 88.2% (adjusted 95% CI, 79.3 to 93.3; P<0.0001) for 40 mg/kg, and 75.0% (adjusted 95% CI, 61.0 to

84.0; P<0.0001) for 10 mg/kg [1816] Together, the phase 1 and 2 trials of CIS43LS provide proof of concept that a MAb with an extended half-life can safely confer protection against Pf infection.

As a follow-up to the VRC phase 1 L9LS study, we conducted a phase 2 trial in Mali to assess the safety and efficacy of a single subcutaneous injection of L9LS against *P. falciparum* infection in healthy children aged 6-10 years over a 6-month malaria season (ClinicalTrials.gov number, NCT05304611). In Part A, safety was assessed at three escalating dose levels in adults, followed by two escalating dose levels in children aged 6-10 years. In Part B, children aged 6-10 years were randomly assigned (in a 1:1:1 ratio) to receive 150 mg of L9LS, 300 mg of L9LS, or placebo. The primary efficacy end point, assessed in a time-to-event analysis, was the first *P. falciparum* infection detected on blood-smear examination, which was performed at least every 2 weeks for 24 weeks. A secondary efficacy end point, assessed in a time-to-event analysis, was the first episode of clinical malaria. At enrollment, all the participants received artemether–lumefantrine to clear possible *P. falciparum* infection. In Part B, 225 children underwent randomization; 75 were assigned to each trial group. Subcutaneous injection of L9LS was well tolerated and there were no serious adverse events. At 6 months, the efficacy against *P. falciparum* infection of 300 mg of L9LS as compared with placebo was 69.9% (adjusted 95% confidence interval [CI], 50.1 to 81.9; P<0.001), and the efficacy of 150 mg of L9LS as compared with placebo was 66.0% (adjusted 95% CI, 45.0 to 79.0; P<0.001). At 6 months, the efficacy against clinical malaria of 300 mg of L9LS as compared with placebo was 77.4% (adjusted 95% confidence interval [CI], 55.0 to 88.7; P<0.001), and the efficacy of 150 mg of L9LS as compared with placebo was 67.0% (adjusted 95% CI, 39.0 to 82.0; P<0.001). In summary, subcutaneous administration of L9LS to children was protective against *P. falciparum* infection and clinical malaria over a 6-month malaria season in Mali without evident safety concerns.

Based on the increased potency of L9LS in pre-clinical models, its higher concentration (150 mg/mL), and the lower weight of infants and children, we administered L9LS by the SC route, which is the optimal route for application in this age group. As a follow-up to the phase 1 VRC study, this protocol is designed to evaluate the safety and efficacy of L9LS administered SC in healthy children ages 5 months to 10 years in a setting of perennial high malaria transmission in western Kenya. Kenya is currently participating in the RTS,S/AS01 pilot scheduled to end in May 2023. The immediate catchment area of Siaya County Referral Hospital is a control area, so children are not receiving RTS,S/AS01 or any other malaria chemoprevention currently.

Laboratory Assessments of L9LS

Some laboratory assessments in this study are designed to characterize the investigational product. This includes PK analysis and evaluation for anti-drug antibody (ADA) development after product exposure. Other assays may also be completed from stored samples at a later date.

The KEMRI laboratories at Siaya County Referral Hospital and at the Kisian campus in Kisumu will process blood and store coded samples and will either perform sample testing or ship coded samples to designated research laboratories at LIG/NIAID and VRC/NIAID or other approved

collaborators for assays that cannot be performed in Kenya. The key to the code will remain at KEMRI. See section 0 for schedules, blood volumes, and tube types to be used for research sample collection. Research assays will be performed on samples from both study product recipients and placebo controls at baseline and throughout the study.

Tube types for clinical labs are according to institutional requirements and are shown in section 0 to estimate blood volumes. Different tubes for clinical evaluations may be used to meet site requirements. In some instances, coded samples may be transported directly by study staff to the laboratory of an approved collaborator.

PK Analysis

Concentrations of L9LS will be measured by a Meso Scale Discovery LLC–based automation platform and similar methodology as previously described for other VRC MAb products. [1917]

Detection of Anti-Drug Antibodies

Assays for detection of ADA will be performed at specified timepoints following product administration and compared to baseline status using a similar methodology as previously described for other VRC MAb products. [1917]

Measures of MAb-Mediated Protection Against Pf infection and Clinical Malaria

L9LS-mediated protection against naturally occurring Pf infection will be assessed and compared to control subjects. The endpoint defining MAb-mediated protection from Pf infection is the absence of Pf parasites in blood samples obtained from L9LS-recipients collected from day 7 through week 52 (day 196) after study agent administration (and the same for the part 2 extension). The criterion for a case of Pf infection is based on blood smear analysis.

Giemsa-stained thick blood films will be prepared and examined by trained personnel following the standard operating procedure (SOP) based on the standard WHO protocol. Thick blood smears will be prepared from the blood remaining in the collection device, or (at timepoints when no blood collection is planned) from a finger prick sample. The smears will be examined microscopically.

As secondary endpoints, L9LS-mediated protection against clinical malaria will be assessed and compared to control subjects from day 7 through week 24 (month 6) after study agent administration. The two definitions of clinical malaria in this protocol are 1) an illness accompanied by measured fever ≥37.5°C in the previous 24 hours, and Pf asexual parasitemia >5,000 parasites/μL as detected from microscopic examination of thick blood smear, and 2) an illness accompanied by measured fever ≥37.5°C, or history of fever (subjective or objective) in the previous 24 hours accompanied by any level of Pf asexual parasitemia as detected from microscopic examination of thick blood smear that requires administration of anti-malarial treatment.

Clinical malaria will be assessed by both passive surveillance (unscheduled sick visits) and active surveillance (medical history, physical exam, and blood smear at all scheduled visits), but the blood smear will be read in an expedited manner, ideally within one hour, only if the medical history and/or physical exam are suggestive of clinical malaria. Otherwise, blood smears will be read within 48 hours by two independent microscopists. Children with a positive blood smear, regardless of symptoms, will be treated with artemether-lumefantrine (AL), the first-line antimalarial in Kenya. If the child has already departed the clinic, a treatment course of AL will be sent to the child's home with a community interviewer (ideally within 72 hours of taking the blood smear), who will counsel the parent/guardian about treating the child with the full course. Thick and thin blood smears will be prepared from the blood remaining in the collection device, or from a venipuncture or finger prick sample when clinically indicated (or at timepoints when no other blood collection is planned). Giemsa-stained thick and thin blood films will be prepared and examined microscopically by trained personnel following the SOP based on the standard WHO protocol. Thick and thin blood smears will be used for diagnosis throughout the study.

Additionally, another secondary endpoint, *Plasmodium* 18S ribosomal ribonucleic acid (rRNA) RT-PCR, will be performed by Dr. Sean Murphy, University of Washington. The assay will be applied to dried blood spots using methods substantially equivalent to those reviewed extensively by the FDA for Biomarker Qualification of the 18S rRNA by the University of Washington for CHMI trials (Biomarker Qualification Letter DDTBMQ000044, Oct 12, 2018). The assay has been validated for use on dried blood spots.

Research blood samples may also be used for Pf malaria parasite genome analysis that will be conducted by Dr. Daniel Neafsey, Harvard School of Public Health.

Repeat Dosing: ADA, PK

All MAbs are potentially immunogenic and can result in the formation of ADA, particularly with repeated dosing.[2018, 1917] An ADA response can result in diminished efficacy by altering the PK properties of the MAb, and can also promote adverse reactions to the MAb, including infusion reactions and local and systemic immune reactions.[20,21] In the case of the widely used tumor necrosis factor (TNF)-alpha inhibitor MAbs, rates of ADA formation are generally higher in patients treated with chimeric anti-TNF constructs compared with fully human MAbs.[2220, 2119] Formation of ADA to anti-TNF MAbs has also been linked to subtherapeutic serum MAb levels that may occur during intermittent dosing.[2321, 2220] Moreover, some studies suggest that intramuscular and subcutaneous administration of MAbs is more immunogenic than intravenous administration.[2321, 2220]

As described above, L9 is a fully human IgG1 MAb that was modified with the Fc region LS mutation (L9LS) to increase neonatal Fc receptor (FcRn) binding and consequent antibody half-life. Other human IgG1 MAbs with LS mutations produced by the VRC that target HIV and Ebola have been tested in over 30 clinical trials, and ADA has not been observed to date, including in individuals who

received up to five administrations of the same MAb. For example, VRC01 was found to be safe and well tolerated at doses ranging from 5-40 mg/kg administered IV and at 5 mg/kg SC in clinical trials that involved HIV-infected and non-infected adults.[19,24-26] In these studies, VRC01 retained its expected neutralizing activity in participants' serum and no ADA responses were detected over the course of multiple administrations. ADA responses against the anti-malarial MAbs CIS43LS and L9LS have not yet been assessed in serum samples collected during the phase 1 US trials[13,17] or the ongoing phase 2 trials in Mali.

Regulatory authorities in the US and Europe request premarketing evaluations for immunogenicity (ADA testing) for all novel biologics.[21,27] FDA recommends the following regarding the implementation of ADA testing in clinical trials:

*"FDA recommends that sponsors obtain **pre-treatment samples** from all subjects. Because there is the potential for pre-existing antibodies or confounding components in the matrix, understanding the degree of reactivity before treatment is essential. The sponsor should obtain subsequent samples, with the timing depending on the frequency of dosing. Optimally, samples taken **7 to 14 days after the first exposure** can help elucidate an early IgM response. Samples taken at **3 to 6 weeks after the first exposure** are generally optimal for determining IgG responses. IgA responses may peak earlier than IgG responses, at around 2 to 3 weeks after antigen exposure (Schütz et al. 2013; Macpherson et al. 2008). For individuals receiving a single dose of a therapeutic protein product, these time frames may be adequate. However, for subjects receiving a therapeutic protein product at multiple times during the trial, the sponsor should obtain samples at appropriate intervals throughout the trial and obtain a sample approximately 30 days after the last exposure. For products with long half-lives, samples should be obtained **approximately five half-lives after last exposure**."*

"Samples to determine serum concentrations of the therapeutic protein product should be obtained at the same time as immunogenicity samples. Testing such samples can provide information on whether the therapeutic protein product in the samples is interfering with ADA testing and whether ADA is altering the therapeutic protein product's pharmacokinetics."

In addition to measuring ADA in the pre-MAb administration samples (already collected in the first year of the study), the protocol extension will follow FDA recommendations by collecting serum samples for PK and ADA testing before re-dosing of L9LS/placebo and periodically after re-dosing. The final assessment time point (Day 364) represents approximately 6.5 half-lives after the last exposure since the estimated half-life of L9LS was 56 days in the phase 1 US trial.

Risk/Benefit Assessment

Known Potential Risks

Risks of L9LS: There have been no notable safety signals in the VRC 614 phase 1 trial (the first in human study of this product) to date, which includes 12 weeks of follow-up after initial administration and 8 weeks after CHMI. A similar malaria antibody, CIS43LS, was evaluated as safe and well tolerated in the VRC 612 study. [1311] Similarly, no significant safety concerns have arisen in the completed trial of CIS43LS in healthy Malian adults at 6 months after product administration. [1816] The study in Mali was done with a dose of 40 mg/kg IV (110 participants), 10 mg/kg IV (110 participants), and placebo (110 participants). As described above (Section 2.2.1.3), an ongoing phase 2 trial in Mali is assessing the safety and efficacy of subcutaneous injection of L9LS against *P. falciparum* infection in healthy children aged 6-10 years over a 6-month malaria season (July-December). In Part A (initiated in March 2022), safety was first assessed at three escalating dose levels in adults (n=18), followed by two escalating dose levels in children aged 6-10 years (n=18). In Part B (initiated in July 2022), 225 children aged 6-10 years were randomly assigned (in a 1:1:1 ratio) to receive 150 mg of L9LS, 300 mg of L9LS, or placebo. Before the second malaria season (starting in June 2023) approximately 90% of pediatriac participants were re-enrolled in a 1-year extension and were re-randomized within their original study arms and received placebo or L9LS at the dose they received in year 1 (150 mg or 300 mg; or 150 mg for children who received placebo in year 1). Both the first and second subcutaneous injections of L9LS were well tolerated and there have been no serious adverse events.

In Part 1 of this study, safety was assessed at three escalating dose levels in older children 6 – 10 years (n=36), and also at these same doses in children aged 5 – 59 months (n=36). From the 72 participants, 54 received L9LS. The subcutaneous injections of L9LS were well tolerated and no serious adverse events were reported.

In a preclinical TCR study, L9LS produced membrane and cytoplasmic binding in infrequent ductal and acinar epithelial cells in the human salivary gland which is of unclear clinical significance. Binding occurred in only 1-5% of cells and was scattered without evidence of clustering. Histopathology findings suggests the human salivary tissues were sourced from surgical excisions with evidence of pre-existing inflammation, which may have contributed to the observed binding. We suspect any effect on the salivary glands from L9LS, if present, would be transient due to the clearance of the passively transferred antibody and the rapid turnover of salivary gland epithelium. To address these findings, the trial will exclude any population at an increased risk for salivary gland dysfunction and will prospectively monitor for signs and symptoms that may be indicative of salivary gland hypofunction. Subject matter experts in salivary gland disorders at the National Institute of Dental and Craniofacial Research who are board-certified by the American Board of Oral and Maxillofacial Pathology have agreed to assist the protocol team by being available for consultation throughout the trial. In addition, L9LS is being evaluated in an ongoing phase 1 study in healthy adult volunteers at the VRC to assess safety, tolerability, PK, and protective efficacy, as described in section 2.2.1. Safety assessment in the phase 1 trial includes prospective monitoring

for signs and symptoms that may be indicative of salivary gland hypofunction. Since September 13, 2021, 18 subjects have received L9LS either IV or SC, and there have been no SAEs and no safety pauses. All systemic symptoms were only mild to moderate and self-limited. Mild or moderate solicited systemic AEs were reported by 5 subjects (27.8%). Solicited systemic AEs include mild malaise (5 subjects), mild or moderate headache (4 subjects), mild chills (2 subjects), mild or moderate nausea (2 subjects), and mild joint pain (1 subject). There were also 2 related unsolicited AEs that were reported (lymphadenopathy and elevated serum creatinine), both of which were grade 1. All IV and SC administrations were well tolerated. Of the 13 subjects who received L9LS IV, 4 reported mild pain and 1 reported moderate bruising at the site of administration. Of the 5 subjects who received L9LS SC, 2 reported mild pain, 1 reported mild pruritus and 1 reported mild redness at the site of administration. The protocol and consent will be updated if new information about risks and side effects becomes available from the ongoing phase 1 study.

Risks of MAb Administration: Administration of MAbs may cause immune reactions such as acute anaphylaxis, serum sickness, and the generation of antibodies. However, these reactions are rare and more often associated with MAbs targeted to human proteins or with the use of mouse MAbs that would have a risk of human anti-mouse antibodies. [2826] In this regard, because L9LS is targeted to a parasite antigen and is a human MAb, it is expected to have a low risk of such side effects.

Typically, the side effects of MAbs are mild to moderate and may include local reactions at the injection site (including pain, redness, bruising, swelling, pruritis) and systemic reactions such as fever, chills, rigors, nausea, vomiting, pain, headache, myalgia, arthralgia, dizziness, fatigue, shortness of breath, bronchospasm, hypotension, hypertension, pruritus, rash, urticaria, angioedema, diarrhea, tachycardia, or chest pain. Healthcare staff will be appropriately trained and necessary medical equipment will be readily available at the clinic where the study agent is administered. Clinical use of MAbs that are targeted to cytokines or antigens associated with human cells may be associated with an increased risk of infections; [2826] however, this is not expected to be a risk for a MAb targeted to a parasite antigen.

Severe reactions, such as anaphylaxis, angioedema, bronchospasm, hypotension, and hypoxia, are infrequent and more often associated with MAbs targeted to human proteins or with non-human MAbs, such as a mouse MAb. [2826] Most administration-related events occur within the first 24 hours after MAb administration.

Published experience with human MAbs directed against cell surface targets on lymphocytes shows that administration of a MAb may be associated with cytokine release, causing a reaction known as cytokine release syndrome (CRS). [2927] CRS reactions commonly occur within the first few hours of administration and with the first MAb administration received. This is because the cytokine release is associated with lysis of the cells targeted by the MAb and the burden of target cells is greatest at the time of the first MAb treatment. With licensed therapeutic MAbs, CRS is managed by

administering histamine blockers.[3028] Supportive treatment may also be indicated for some signs and symptoms.

Delayed allergic reactions that include a serum sickness type of reaction characterized by urticaria, fever, lymph node enlargement, and joint pains, typically occur several days after MAb exposure and are more commonly associated with chimeric types of MAbs.[2826] In general, and with due consideration of the needs dictated by individual subject symptoms and treating clinician discretion, immediate and delayed reactions to study product will be managed according to the principles of the American Academy of Allergy, Asthma, and Immunology guidelines.[3129]

Participation in this study may limit a subject's eligibility for future MAb studies.

Risks of Placebo Administration: There are no risks of the placebo (normal saline) other than injection related events such as transient vasovagal-mediated hypotension or local site injection bruising, swelling, or infection, the risk of which will be minimized by using sterile techniques.

Risks of Blood Drawing: Drawing blood by venipuncture or fingerprick/ heel prick may cause pain, bruising, and a feeling of lightheadedness or fainting. Rarely, an infection may develop at the site where blood is taken.

Risks of DP Administration: Before study agent administration, all subjects will be given DP, the second-line antimalarial according to the Kenyan national guidelines, to clear any preexisting Pf blood-stage infection. DP is being selected over AL for logistic reasons, as it is administered only once daily for three days, simplifying the process of observing each administration and therefore improving compliance and ensuring parasite clearance prior to MAb administration. Clearance of preexisting Pf blood-stage infection is necessary to accurately assess the primary endpoint of Pf blood-stage infection as detected by microscopic examination of thick blood smear after administration of L9LS or placebo, since preexisting Pf blood-stage parasites cannot be reliably distinguished from newly transmitted Pf blood-stage parasites.

Although there is some evidence to suggest that exposure to Pf blood-stage infection may interfere with the human immune response to malaria vaccine candidates, clearance of preexisting Pf blood-stage infection is not considered necessary for L9LS efficacy since it directly mediates its protective effect against sporozoites and does not depend on induction of any host immune response.[1412]

Adverse reactions (ARs) to DP are estimated to occur in 1.8% of patients, according to two studies included in a recent meta-analysis. [3230] Reactions typically do not require stopping treatment. In postmarketing experience, serious hypersensitivity reactions including anaphylaxis and serious skin reactions (bullous eruption) have been reported. Children receiving this medication should have a weight of at least 5 kilograms. Medication like macrolide antibiotics, quinolones, and certain antihistamines like astemizole will be avoided during the period of giving DP. Very common side effects (≥1 in 10) in children include pyrexia; common side effects (≥1 in 100 to <1 in 10) diarrhea, vomiting, abdominal pain, rashes, irregular heart rate, QT/QTc prolongation, leukopenia/neutropenia. Possible side effects will be communicated to the parent/guardian. Individuals with known sensitivity or contraindications to the antimalarials administered in this study are excluded from participation.

In a recently completed therapeutic efficacy trial assessing AL and DP (ClinicalTrials.gov Identifier: NCT05060198), both drugs were well tolerated (<2% of children vomited the first dose of medication in both arms). Only one severe adverse event was recorded—a participant in the AL arm hospitalized with severe dehydration due to a diarrheal illness; the child recovered fully. This event was deemed unrelated to the study drug.

A complete list of side effects and contraindications is provided in the product Guide for Healthcare Professionals (insert). [3331]

Known Potential Benefits

Subjects may not receive direct health benefit from study participation. Depending on whether L9LS confers protective efficacy, subjects receiving a sufficient dose of L9LS may experience some protection against Pf infection and clinical malaria.

In the future, others may benefit from knowledge gained in this study that may aid in the development of malaria prevention.

Assessment of Potential Risks and Benefits

The study population lives in a setting of perennial high transmission and so is at significant risk of malarial infection and disease. As described above, it is possible that some subjects may benefit from study participation by receiving some protection from malaria. Therefore, the value of the information that will be gained from this study for developing malaria prevention strategies justifies the potential risks of study participation described above. Additionally, potential risks are minimized by careful design of subject eligibility criteria and monitoring after study product administration.

OBJECTIVES AND ENDPOINTS

OBJECTIVES	ENDPOINTS	JUSTIFICATION FOR ENDPOINTS
Primary		
4. Part 1: Safety: To evaluate the safety and tolerability of L9LS administered at doses of 5 mg/kg, 10 mg/kg, and 20 mg/kg by SC administration to healthy Kenyan children.<br>5. Part 2: Safety: To evaluate the safety and tolerability of L9LS administered at doses of 10-20 mg/kg by SC administration to healthy Kenyan children<br>6. Part 2: Efficacy: To assess the efficacy of two doses of L9LS at a concentration of 10-20 mg/kg, administered SC to participants aged 5-59 months of age against first/only Pf malaria infection diagnosed by blood smear microscopy (irrespective of fever) over 12 months compared to placebo.	3. Parts 1 & 2: Incidence and severity of local and systemic AEs occurring within 7 days after the administration of L9LS, and incidence of SAEs throughout the study period.<br>4. Part 2: Pf blood-stage infection as detected by microscopic examination of thick blood smear for 52 weeks after administration of L9LS or placebo.	1. Assessment of AEs is a standard measure of study agent safety and tolerability.<br>2. Blood smear is the gold standard for diagnosis of blood-stage Pf infection.

OBJECTIVES	ENDPOINTS	JUSTIFICATION FOR ENDPOINTS
Secondary		
8. To assess the efficacy of one dose of L9LS at 10-20 mg/kg against first/only Pf malaria infection diagnosed by blood smear microscopy over 3 and 6 months compared to placebo.<br>9. To assess the efficacy of one dose of L9LS at 10-20 mg/kg against first/only Pf malaria infection diagnosed by blood smear microscopy over 12 months compared to placebo.<br>10. To evaluate the efficacy of L9LS at 10-20 mg/kg against first/only Pf malaria infection as detected by PCR over 3, 6 and 12 months compared to placebo.<br>11. To assess protection of L9LS at 10-20 mg/kg against clinical malaria (first/only and all episodes) at 3, 6 and 12 months compared to a placebo.<br>12. To assess the primary objective and secondary objectives 1-4 among children 5-17 months of age.<br>13. To assess the primary objective and secondary objectives 1-4 among children 18-59 months of age.<br>14. To evaluate the PK of L9LS throughout the study at weight-based dose levels of 5 mg/kg, 10 mg/kg, and 20	8. Pf blood-stage infection as detected by microscopic examination of thick blood smear for 12 and 24 weeks after administration of L9LS or placebo.<br>9. Pf blood-stage infection as detected by microscopic examination of thick blood smear for 52 weeks after administration of L9LS or placebo.<br>10. Pf blood-stage infection as detected by RT-PCR for 12, 24 and 52 weeks after administration of L9LS or placebo.<br>11. Incidence of clinical malaria (definitions 1 and 2, see section 0) for 12, 24 and 52 weeks after administration of L9LS or placebo.<br>12. See 1 – 4 above<br>13. See 1 – 4 above<br>14. Measurement of L9LS in sera of recipients, including PK analysis of L9LS and the association of L9LS concentration with Pf infection risk and with clinical malaria risk.	1. Blood smear is the gold standard for diagnosis of blood-stage Pf infection.<br>2. See 1 above.<br>3. RT-PCR is more sensitive than blood smear for detecting Pf blood-stage infection.<br>4. 6–59-month-old children in Kenya are at high risk for clinical malaria.<br>5. See 1 – 5 above<br>6. See 1 – 5 above<br>7. Concentrations of L9LS in blood will help assess durability of L9LS at each dose level and will allow for correlation with<br>a. Pf infection<br>b. Clinical malaria risk.

OBJECTIVES	ENDPOINTS	JUSTIFICATION FOR ENDPOINTS
mg/kg (part 1) and fixed doses of 10-20 mg/kg (part 2) in healthy Kenyan children, and<br>a. To correlate L9LS serum concentration with Pf infection risk.<br>b. To correlate L9LS serum concentration with clinical malaria risk.		
Tertiary/Exploratory		
9. To assess the impact of L9LS on hospitalizations with malaria<br>10. To determine whether ADA to L9LS can be detected in sera of recipients at specific timepoints throughout the study.<br>11. To assess for IgG1 allotypes and allotype specific effects on L9LS PK.<br>12. To determine if the efficacy of L9LS is specific to certain Pf parasite genotypes at the CSP locus.<br>13. To explore the impact of pre-existing parasitemia on the protective efficacy and PK of L9LS.<br>14. To explore the impact of pre-existing CSP antibodies	9. Proportion of participants hospitalized with malaria<br>10. Measurement of ADA to L9LS in sera of recipients.<br>11. Assessment of IgG1 allotypes and allotype-specific effects on L9LS PK.<br>12. CSP genotyping of parasites isolated from study subjects.<br>13. Pre-existing parasitemia detected by microscopic examination of thick blood smears or RT-PCR before L9LS administration.<br>14. Pre-existing CSP-specific antibodies measured in sera collected before L9LS administration.	1. It is possible that L9LS might not prevent malaria but might prevent the progression to severe malaria.<br>2. ADA to L9LS may impact the PK and activity of L9LS.<br>3. Subject IgG1 allotype may impact the PK and activity of L9LS.<br>4. L9LS efficacy may be specific to certain Pf parasite genotypes at the CSP locus.<br>5. Pre-existing parasitemia may impact the protective efficacy and PK of L9LS.

OBJECTIVES	ENDPOINTS	JUSTIFICATION FOR ENDPOINTS
on the protective efficacy and PK of L9LS.<br>15. To assess the effect of L9LS on antibodies to measles in the younger age group.<br>16. To compare the efficacy of one versus two doses of L9LS against Pf infection and clinical malaria over 12 months.	15. Measles virus-specific IgG antibodies among subjects <9 months of age at enrollment.<br>16. Pf infection and clinical malaria over 12 months.	6. Pre-existing CSP-specific antibodies may impact the protective efficacy and PK of L9LS.<br>7. Address concerns that L9LS might impact immune response to measles vaccine.<br>8. Pf infection and clinical malaria are our primary and secondary endpoints.
Follow-on Study Primary Objectives		
Follow-on Safety (part 1b)<br>5. To evaluate the safety and tolerability of L9LS administered at doses of 30 mg/kg and 40 mg/kg by SC administration to healthy Kenyan children.<br>*Follow-on Part 2 Extension*<br>6. To evaluate the safety and tolerability of a third and fourth SC dose of L9LS at 20–40 mg/kg (compared to placebo) in healthy Kenyan children.<br>7. To evaluate the PK of L9LS throughout the follow-up phase at 20–40 mg/kg in healthy Kenyan children.<br>8. To determine if ADAs to L9LS can be detected in sera of recipients at specific timepoints throughout the	4. Incidence and severity of local and systemic AEs occurring within 7 days after the administration of L9LS and incidence of serious adverse events (SAEs) throughout the study period.<br>5. Measurement of L9LS in sera of recipients.<br>6. Measurement of ADA in sera of recipients.	1. Assessment of AEs is a standard measure of study agent safety and tolerability.<br>2. Concentrations of L9LS in blood will help assess durability of L9LS at each dose level.<br>3. ADA to L9LS may impact the PK and activity of L9LS.

OBJECTIVES	ENDPOINTS	JUSTIFICATION FOR ENDPOINTS
study and to correlate the occurrence of ADAs with L9LS PK.		
Extension Phase Exploratory Objectives (see **Figure 4** for study arm definitions)		
4. To assess the efficacy of an additional one or two SC doses of L9LS over one year at 20–40 mg/kg in mediating protection against Pf infection and clinical malaria in healthy Kenyan children during a second year of follow-up compared to those who received placebo only (arms 1 vs. 3; arms 2 vs. 3) and in those receiving two L9LS doses in the extension versus one (arms 1 vs. 2)<br>5. To evaluate the PK of L9LS throughout the study and to correlate L9LS serum concentration with Pf infection risk and clinical malaria risk<br>6. To evaluate the effect of a third (and fourth) dose of L9LS on development of acquired immunity (measured by antibodies)	7. Pf blood-stage infection as detected by microscopic examination of thick blood smears obtained between 1 week and 52 weeks after administration of L9LS or placebo.<br>8. Pf blood-stage infection as detected by RT-PCR from dried blood spots obtained between 1 week and 52 weeks after administration of L9LS or placebo.<br>9. Incidence of clinical malaria (see section 0) between 1 week and 52 weeks after administration of L9LS or placebo.<br>10. PK analysis of L9LS and the association of L9LS concentration with Pf infection risk.<br>11. PK analysis of L9LS and the association of L9LS concentration with clinical malaria risk.<br>12. Levels of naturally occurring antimalaria antibodies in participant blood at selected time points	1. Blood smear is the gold standard for diagnosis of blood stage Pf infection.<br>2. RT-PCR is more sensitive than blood smear for detecting Pf blood-stage infection.<br>3. Children <5 years of age in Kenya are at high risk for clinical malaria.<br>4. Concentrations of L9LS in blood will help assess durability of L9LS and will allow for correlation with Pf infection risk.<br>5. Concentrations of L9LS in blood will help assess durability of L9LS and will allow for correlation with clinical malaria risk.<br>6. Concentrations of anti-malaria antibodies will help assess potential for rebound after L9LS

STUDY DESIGN

Overall Design

This is a three-part, phase 2 trial evaluating the safety and tolerability of SC administration of L9LS in healthy Kenyan children, as well as its protective efficacy against naturally occurring Pf infection over a 12-month period in healthy Kenyan children 5-59 months of age. The primary study hypotheses are that L9LS will be safe and will produce protection against malaria infection. Before study agent administration, all subjects will be given an antimalarial (in most cases, DP) to clear any preexisting Pf blood-stage infection.

The target age group for this trial is children 5 to 59 months. Young children are notable for having substantial morbidity and mortality from malaria. For this reason, RTS,S/AS01 is now being routinely implemented in a large-scale pilot and as of 2023 as part of the routine immunization programme in western Kenya by the Kenya National Vaccines and Immunizations Program (NVIP) to children beginning at 6 months, with the second, third, and fourth doses occurring at 7, 9, and 24 months, respectively. RTS/S/AS01 confers ~ 36% protection against clinical infection after 4 years. Thus, an intervention that could provide high-level durable efficacy for 6 or 12 months (>70%) could have important implications for malaria control in perennial transmission settings, with advantages related to compliance and increased protection. Given the potential interest in MAbs as an alternative to malaria vaccines, we propose to increase the sample size in the 5- to 17-month-old age group to enable a comparison to efficacy of RTS,S/AS01 from data in this age group in the same region. A key feature of this study will be assessment of 1 of 3 fixed-dose regimens given by the SC route at 1 injection site (in infants and children up to 15 kg) or possibly 2 sites (children > 15 kg). (Children >15 kg would receive a greater dose administered in 1 or 2 sites to maintain a minimum dose concentration of 10 mg/kg.) This fixed-dose regimen design is simple, easy for implementation by clinicians, and provides a “real” world setting for administration.

For the current study in western Kenya, we propose an age de-escalation approach to test the safety of L9LS first in older children (5-10 years of age) and then in the younger target age group for the trial (5-59 months of age), at doses of 5 mg/kg, 10 mg/kg, and 20 mg/kg administered via SC injection. The initial age de-escalation component will enroll 12 participants in each age-dose group in a 3:1 ratio of L9LS to placebo. The second part is a randomized, double-blind, placebo-controlled trial to assess safety and protective efficacy of L9LS versus placebo in children ages 5-59 months. In this part of the study, children will be randomized 2:1 to L9LS or placebo in an age-stratified manner so that there will be one L9LS cohort for younger children, aged 5-17 months, and one L9LS cohort for older children, aged 18–59 months, which together will constitute one L9LS study arm. A placebo study arm will be composed of children aged 5–59 months (composed of subjects from both age groups).

Part 1: Double-blinded, age de-escalation, dose-escalation trial to assess tolerability and safety

In a stepwise fashion, children aged 5-10 years will be screened, and those eligible will undergo a pre-enrolment process after obtaining informed consent. Two to three weeks after a treatment course with DP for parasite clearance, individuals will receive 5 mg/kg of L9LS or placebo via SC injection and will be followed for 3 months post-injection to assess tolerability and safety. If acceptable tolerability and safety profiles are met at 1 week post-injection, we will dose children ages 5-59 months with 5-mg/kg L9LS or placebo while dosing another cohort of children ages 5-10 years with 10 mg/kg of L9LS or placebo. If after 1 week, L9LS at 10 mg/kg is found to be safe in children aged 5-10 years, and 5 mg/kg is found to be safe in children ages 5-59 months, we will then assess tolerability and safety of 20 mg/kg L9LS or placebo in children ages 5-10 years, and 10 mg/kg L9LS or placebo among children ages 5-59 months, using the approach in Figure 1A above. Should tolerability and safety of all doses be acceptable in children ages 5-59 months, we will move to part 2 of the trial. Dosing in part 1 of the study will be weight-based and all doses will be administered via SC injection. All participants in part 1 will be followed for a total of 3 months.

See section 0 for a detailed schedule of assessments.

Part 2: Evaluating efficacy of monoclonal antibodies

This will be a phase 2, double-blind, randomized, controlled clinical trial to evaluate the efficacy of L9LS against malaria infection among children ages 5 to 59 months in a setting of perennial high transmission. Participants will be stratified by age group (5-17 months and 18-59 months) and within each age group randomly assigned in a 2:1 ratio to receive L9LS or placebo as an initial injection. The two age groups will be enrolled in parallel. Study arms are as follows:

1. L9LS at 10-20 mg/kg (SC administration):
    a. 5-17 months of age
        i. L9LS dose 2 at 6 months
        ii. Placebo dose 2 at 6 months
    b. 18 months to 59 months of age
        i. L9LS dose 2 at 6 months
        ii. Placebo dose 2 at 6 months
2. Placebo arm:
    a. 5-17 months of age (with placebo dose 2 at 6 months)
    b. 18 months to 5 years of age (with placebo dose 2 at 6 months)

After an initial screening, we will obtain informed consent from those eligible; for those consenting, we will conduct a more complete screening that includes a physical exam and blood draw to assess parasitemia, hematological and biochemistry values, antimalarial antibody and antigen titers, and other parameters; for those who remain eligible, we will administer a full, weight-based course of DP, the second-line antimalarial in Kenya, to clear parasites. (For children who have taken

an antimalarial within the last 2–4 weeks, this antimalarial course will be deferred at least 7 days from the date of the last dose of AL or at least 4 weeks after the last dose of DP.) Between 14 and 21 days after the first dose of DP (considered day 1 for dosing window), children will return and then be randomized to receive a SC injection(s) of either placebo or L9LS, according to study arm. Children will be considered enrolled once they are randomized on the Day 0 dosing visit. We will use a 'constrained' fixed dosing approach that ensures each child receives a minimum of 10 mg/kg in a relatively simple dosing scheme. See section 0 for exact dosing information and the number of required injections of two syringe types for various participant weights.

Children will be scheduled for monthly visits at the study health facility throughout the duration of the trial, and parents/guardians will be asked to bring their child to the study facility for unscheduled visits for assessment of any concerns or illness (free of charge). Starting after the first monthly visit after IP administration, study staff will contact parents/caregivers 2 weeks after scheduled visits (via home visits or telephone calls) to administer questionnaires about symptomatology and care-seeking behavior. If a child has fever or history of fever, they will be brought to the clinic for a finger prick for malaria testing.

At monthly visits, a capillary blood draw will be conducted to collect a sample for blood smear microscopy and PCR. Questionnaires will be administered focusing on development of symptoms, care-seeking behavior and any medications taken. At key time points, venous blood will be collected to assess antibody titers.

Benefit of a second dose at 6 months: Participants in the L9LS arm will be randomized to receive either a second dose of L9LS or placebo at 6 months to evaluate the additional efficacy of a second dose. (Those in the placebo arm will receive a second injection of placebo.)

See section 0 for a detailed schedule of assessments.

Year 2 Follow-on Study (Safety and Extension)

<u>Part 1b. Follow-on Safety</u>

As part of the follow-on study, an initial dose escalation study will assess the safety and tolerability of 30 mg/kg L9LS and 40 mg/kg L9LS. An additional 12 children ages 5 – 71 months will be randomized to receive L9LS at 30 mg/kg (weight-based dose) or placebo in a 3:1 ratio. If no safety signals are seen after 7 days, an additional 12 children aged 5 – 71 months will be randomized to receive L9LS at 40 mg/kg (weight-based dose) or placebo in a 3:1 ratio. If no safety signals are seen then the follow-on extension will move forward, pending DSMB review of the data and approval to proceed. Children will be followed for a total of three months after dosing. Subjects will be followed at study visits 1, 3, 7, 14, 21, and 28 days after administration, and then at month 2 and month 3.

Follow-on part 2 extension

In the extension phase, all parents/guardians of participants who are currently enrolled in the part 2 efficacy study will be invited to extend their child's participation for approximately 13 months from the time of re-consenting. Parents/guardians of children can be consented to allow participation of their children in the extension phase at any time before their termination in part 2 and up to 3 months after their part 2 close-out. Participants will remain in their same randomization groups from part 2. Those who received two doses of L9LS in part 2 will also receive two doses of L9LS, six months apart, in the extension phase. Those who received one dose of L9LS will receive one dose (plus a dose of placebo six months later), and those who received two doses of placebo will receive an additional two doses of placebo, six months apart.

Before study agent administration, all subjects will be given dihydroartemisinin-piperaquine to clear any preexisting Pf blood-stage infection. Subjects will be followed at study visits 1, 3, 7, and 28 days after administration, and then will have alternating biweekly home visits and clinic visits, identical to the visit schedule during year one, up through 52 weeks after the initial follow-on extension dose in year two. Primary study assessments include medical history, physical examination, and blood collection for pharmacokinetics (PK), anti-drug antibody (ADA) assessments, identification of Pf infection by microscopic examination of thick blood smears and RT-PCR, and other research laboratory evaluations.

Safety outcomes will be evaluated within arms to assess the safety of a third and fourth L9LS dose vs. placebo in year 2 (1 vs. 3 and 2 vs. 3). Finally, safety outcomes of L9LS vs. placebo will be evaluated in aggregate (1 + 2 vs. 3).

PK of L9LS will be evaluated throughout the study for those receiving various numbers of L9LS doses. The presence of ADA to L9LS will be assessed in sera of recipients at specific timepoints throughout the study and will be correlated with L9LS PK.

In exploratory analysis, L9LS serum concentration will be correlated with the risk of Pf infection (assessed by blood smear and RT-PCR) and clinical malaria. The protective efficacy of L9LS against *P. falciparum* infection (by blood smear and RT-PCR) and clinical malaria will be assessed by comparing: 1) arm 1 vs. arm 3 (over 12 months); 2) arm 2 vs. arm 3 (over 12 months); arms 1+2 vs. arm 3 (over 6 months); and arm 1 vs. 2 (over 12 months).

Study site

The CDC has collaborated with KEMRI in western Kenya to evaluate tools for malaria transmission and burden reduction for over 40 years. The collaboration has a highly experienced clinical trials unit that includes two pediatricians, immunologists, medical officers, pharmacists, clinical officers, nurses, quality control and regulatory officers. This group has been involved in phase 2, 3, and 4 trials of RTS,S/AS01 and conducted the largest phase 2 trial of the PfSPZ Vaccine in 5- to 12-month-old infants as well as a number of pharmaceutical clinical trials. The clinical trials unit is based in Siaya County Referral Hospital, the largest hospital in Siaya County, western Kenya. Here, malaria transmission is perennial with seasonal peaks following the long and short rains in June/July and November/December, respectively. Clinical malaria incidence (any parasite density by microscopy in a child who reports fever within the previous 24 hours) among children aged 5 to 17 months was 3.06 cases per year in 2019–2020. In the recent PfSPZ Vaccine trial among infants 5-12 months of age at the time of first vaccination (with a 3-dose series over 5 months), 66.7% of participants in the placebo arm had at least one Pf infection based on microscopy within 6 months after the last dose; in another trial among 5- to 17-month-olds at the time of first vaccination (with a 3-dose series over 3 months), 46.8% had at least one clinical malaria episode (parasitemia >0 parasites/µL and fever/history of fever within 24 hours) within 6 months after the last dose. Currently, the RTS,S/AS01 malaria vaccine is being rolled out by the Kenya Expanded Programme on Immunization in select sub-counties in western Kenya; the RTS,S/AS01 vaccine is not being implemented in Alego-Usonga sub-county (where the clinical research site is located). The current Malaria Vaccine Pilot Evaluation is scheduled to run until May 2023, so the earliest that RTS,S/AS01 vaccinations would be offered in Karemo, where the study site hospital is, would be June 2023. In part 2, staff will also recruit and follow up patients at Kogelo Dispensary, a satellite clinic referring patients to Siaya County Referral Hospital.

Scientific Rationale for Study Design

This two-part study was designed to test L9LS in the setting of naturally occurring Pf infection and in a population that could potentially benefit from a novel therapeutic for malaria prevention, with initial safety data collected from older children prior to proceeding with the enrollment of younger ages.

Both parts of the study will use randomization and a placebo control, and double-blinding to minimize bias in subject selection and study assessments. The placebo will be inactive (normal saline) rather than a comparator MAb, as currently there are no licensed anti-malaria MAbs available.

Year 2 Follow-on Study (Safety and Extension)

The protocol follow-on study was designed to evaluate the safety and PK of a third and fourth L9LS dose in the setting of naturally occurring Pf infection and in a population that could potentially benefit from a novel therapeutic for malaria prevention.

The protocol extension will use a double-blind design in the cohorts of children to minimize bias in subject selection and study assessments. The placebo will be inactive (normal saline) rather than a comparator MAb, as currently there are no licensed anti-malaria MAbs available.

Justification for Dose

The doses used in this study were selected based on preclinical data and data generated in the phase 1 VRC study, which tested the dose range included in this protocol and showed that 4/5 individuals that received 5 mg/kg SC were protected and 4/4 that received 20 mg/kg IV were protected following CHMI. This study will test fixed doses of L9LS in children to approximate the weight-based dosing used in the phase 1 VRC study. Compared to weight-based dosing, fixed dosing will provide a greater range of antibody concentrations *in vivo* for determining a protective L9LS titer. Fixed dosing is also simpler to administer, reduces the risk of medication errors, and is generally more cost effective—factors that are relevant to the potential use of L9LS in malaria-endemic regions.

In Part 1, subjects aged 5 months to 10 years will receive weight-based doses of 5 mg/kg, 10 mg/kg, and 20 mg/kg. Children weighing >30 kg will be excluded given that administration of 20 mg/kg would entail more than 2 mL of L9LS at two different injection sites for heavier children. According to a recent study in the area, female children 10 years old had an average weight of 26.3 kg (SD = 5.4 kg) and male children 10 years old an average of 26.9 kg (SD = 5.1 kg).

The doses used in the efficacy study will complement the data generated in the phase 1 VRC study. The doses used in the phase 1 VRC study were derived from 1) efficacy data from the challenge studies performed in mice showing that the protective concentration of antibody *in vivo* is between 5-100 μg/mL in 2 different mouse models of malaria infection; [1412] and 2) PK data from NHP studies with L9LS and prior clinical experience in healthy adults with human MAbs targeting HIV (i.e., VRC01, VRC01LS, and VRC07-523LS) and ebolavirus (MAb114) at the same dose.

In the ongoing phase 2 trial of L9LS in Mali involving healthy children aged 6-10 years, a pre-specified exploratory analysis of the relationship between the weight-based dose of L9LS (mg of L9LS administered per kg of body weight) and protective efficacy suggested that ~12 mg/kg of L9LS corresponds to a protective efficacy against clinical malaria of approximately 75% over six months compared to placebo. In addition, preliminary PK analysis indicates that the average half-life of L9LS in Malian children is ~35 days, compared to ~55 days in Malian adults, and ~65 days in U.S. adults. After receiving 10-20 mg/kg of L9LS, the average number of days required for the serum concentration of L9LS to fall below the lower limit of detection (0.61 μg/mL) was 243 (95% CI 232–254). Therefore, we anticipate that a single dose of L9LS in the range of 10-20 mg/kg will not provide high-level protection (>75%) against malaria over 12 months in healthy Kenyan children 5-59 months of age who are exposed to intense perennial malaria transmission. Therefore, the 1-year

extension of the phase 2 Kenya trial will assess the safety and protective efficacy of L9LS at a dose range of 20-40 mg/kg.

STUDY POPULATION

Inclusion Criteria

Individuals must meet all of the following criteria to be eligible for study participation:

8. Healthy children aged 5 months to 10 years (Part 1) or 5-59 months (Part 2).
9. Weight ≥5 kg and weight ≤30 kg (Part 1) or weight ≥5 kg and ≤22.5 kg (Part 2) or weight ≥5 kg and ≤20 kg (for 30 mg/kg group) or weight ≥5 kg and ≤15 kg (for 40 mg/kg group), to ensure a maximum volume of two 2-mL (Part 1b)
10. Hemoglobin level ≥8 g/dL.
11. Height and weight Z-scores >-2.
12. Living within Alego-Usonga sub-county.
13. Able to participate for the duration of the trial.
14. Parent and/or guardian of participant able to provide informed consent.

Year 2 Extension Inclusion Criteria

Individuals must meet all of the following criteria to be eligible for participation:

1. Participated in part 2 of the protocol.
2. Able to participate for the duration of the extension period.
3. Parent and/or guardian of participant able to provide informed consent.

Exclusion Criteria

Individuals meeting any of the following criteria will be excluded from study participation:

15. Taking long-term cotrimoxazole.
16. Participation or planned participation in any other interventional trial with an investigational product prior to the last required protocol visit or receipt of an investigational product within the past 30 days. (Note: Past, current, or planned participation in observational studies is NOT exclusionary.)
17. Received any doses of any malaria vaccine.
18. Participation in part 1 of this study (for individuals being screened for enrollment into part 2)
19. Age < 12 months at the time the RTS,S/AS01 vaccine is anticipated to become available in the whole of Siaya County
20. Current significant medical condition (neurologic, cardiac, pulmonary, hepatic, endocrine, rheumatologic, autoimmune, renal, oncologic, or hematological) or evidence of any serious

underlying medical condition identified by medical history, physical examination, or laboratory examination.

a. Known sickle cell disease. (Note: Known sickle cell trait is NOT exclusionary.)
b. White blood cell, absolute neutrophil, or platelet count outside the local laboratory-defined limits of normal. Subjects may be included at the investigator's discretion for values that are not clinically significant (ie., do not require any repeat or follow-up).
c. Alanine transaminase (ALT) or creatinine (Cr) level above the local laboratory-defined upper limit of normal. (Subjects may be included at the investigator's discretion for values that are not clinically significant.)
d. Infected with HIV.
e. History of a severe allergic reaction or anaphylaxis.
f. Severe asthma (defined as asthma that is unstable or required emergency care, urgent care, hospitalization, or intubation during the past 2 years, or that has required the use of oral or parenteral corticosteroids at any time during the past 2 years).
g. Pre-existing autoimmune or antibody-mediated diseases including but not limited to: systemic lupus erythematosus, rheumatoid arthritis, multiple sclerosis, Sjogren's syndrome, or autoimmune thrombocytopenia.
h. Known immunodeficiency syndrome.
i. Use of chronic (≥14 days) oral or IV corticosteroids (excluding topical or nasal) at immunosuppressive doses (i.e., prednisone >10 mg/day) or immunosuppressive drugs within 30 days of day 0.
j. Known asplenia or functional asplenia.
k. Clinical signs of malnutrition.
l. Receipt of immunoglobulins and/or blood products within the past 6 months.

21. Any history of menses.
22. Behavioral, cognitive, or psychiatric disease that in the opinion of the investigator affects the ability of the subject to understand and comply with the study protocol.
23. Parental/guardian study comprehension examination score of <80% correct or per investigator discretion.
24. Receipt of a live vaccine or a killed vaccine within the past 2 weeks prior to study agent administration.
25. Known allergies or contraindication to dihydroartemisinin-piperaquine.
26. Use or known need at the time of pre-enrolment (DP administration) of concomitant prohibited medication, including:
    a. Antimicrobial agents of the following classes (systemic use only):
        i. Macrolides (e.g. erythromycin, clarithromycin, azithromycin, roxithromycin)
        ii. Fluoroquinolones (e.g., levofloxacin, moxifloxacin, sparfloxacin)

iii. Pentamidine

b. Antiarrhythmic agents (e.g. amiodarone, sotalol)
c. Antihistamines (e.g. promethazine)
d. Antifungals (systemic): ketoconazole, fluconazole, itraconazole
e. Antiretrovirals: Saquinavir
f. Diuretics (e.g. hydrochlorothiazide, furosemide)
g. Antipsychotics (neuroleptics): haloperidol, thioridazine
h. Antidepressants: imipramin, citalopram, escitalopram
i. Antiemetics: domperidone, chlorpromazine, ondansetron

27. Increased risk of salivary gland hypofunction (dryness of the mouth, swelling under the tongue and/or below the ear, halitosis)
28. History of any other illness or condition which, in the investigator's judgment, may substantially increase the risk associated with the subject's participation in the protocol or compromise the scientific objectives, or other condition(s) that, in the opinion of the investigator, would jeopardize the safety or rights of a subject participating in the trial, interfere with the evaluation of the study objectives, or render the subject unable to comply with the protocol.

If enrollment of subjects (minimum age of 5 months) is completed by November 2022, then there is no chance of overlap between subjects in the MAb trial and those potentially eligible for RTS,S/AS01 (who need to be 6–12 months of age), if the RTS,S/AS01 vaccine is introduced in the entirety of Siaya county in June 2023 (see section 0). Should the anticipated timeline for RTS,S/AS01 routine introduction in all of Siaya county shift earlier, we will re-evaluate eligibility of our MAb trial subjects, to ensure that participation in the MAb trial would not prevent anyone from receiving RTS,S—see exclusion criterion number 6 above. For example, if RTS,S implementation were scheduled for March 2023, we would add an exclusion criterion that subjects need to be at least 12 months of age (and thus ineligible for RTS,S) by March 2023. If children <12 months of age are excluded from enrolment in part 2 of the trial, enrolment will no longer be stratified by age.

Inclusion of Vulnerable Participants

Children: L9LS was evaluated in a phase 1 clinical trial to evaluate safety and protective efficacy of L9LS in healthy, malaria-naive adults. The results showed that L9LS is highly protective at a low dose given by the SC route, is safe and well tolerated and has favorable PK (see summary of safety data in section 0).[17][15] L9LS was also evaluated in an ongoing phase 2 clinical trial in Mali to evalute the safety and protective efficacy of L9LS in healhy children aged 6-10 years. As described in Section 2.2.1.3, the results of the first year of the Mali trial demonstrated that SC administration of L9LS to children was protective against *P. falciparum* infection and clinical malaria over a 6-month malaria season without evident safety concerns. As noted in Section 2.3.1, the Mali trial was extended a second year starting in June 2023. Approximately 90% of participants were re-enrolled in the extension and were re-randomized within their original study arms and received placebo or L9LS at the dose they received in year 1 (150 mg or 300 mg; or 150 mg for children who received placebo in year 1). The second SC administration of L9LS was well tolerated and there have been no safety concerns to date.

Children aged 5–59 months in Kenya are at high risk for malaria infection and clinical disease. Previous research in the study area has indicated that children aged 5 to 17 months had on average 3.06 malaria cases per year in 2019–2020.

Children who have Z scores below -2 for weight-for-age (or BMI for older children) and/or for height-for-age will be excluded, as these children are likely to be malnourished and/or stunted. Children will also be excluded if they weigh <5 kg, which is the minimum weight for dosing with DP. For Part 1, children in the 5- to 10-years age group will be excluded if they weigh >30 kg, as one of the dose groups will be 20 mg/kg; this will require 600 mg of L9LS to be given, up to 2 mL at each of 2 injection sites. Excluding children weighing >30 kg will allow us to avoid using more than 2 injection sites. In Part 2, children weighing >22.5 kg will be excluded, as they would receive less than the minimum desired 10 mg/kg with the proposed fixed dose of 225 mg. In the area surrounding the study site, according to recent survey data, 95% of children between 4 and 5 years old weighed <20 kg.

Illiterate Individuals: We anticipate that many individuals eligible for this study and their parents/guardians will be illiterate in English, so the study team will translate the consent into local languages when appropriate, as described in section 0.

Inclusion of Pregnant Women, Fetuses or Neonates

Not applicable.

Lifestyle Considerations

Beyond the prohibited treatments and procedures listed in section 0, this study would not impact the lifestyle of the subjects.

Screen Failures

Screen failures are defined as subjects who consent to participate in the clinical trial but are not pre-enrolled and dosed with DP. Late screen failures are those who are pre-enrolled and dosed with DP but do not get randomized to receive IP/placebo. A minimal set of screen failure information is required to ensure transparent reporting of screen failure subjects, to meet the Consolidated Standards of Reporting Trials (CONSORT) publishing requirements and to respond to queries from regulatory authorities. Minimal information includes demography, screen failure details, eligibility criteria, and any SAEs.

Individuals who initially do not meet the criteria for participation in this trial (screen failure) because of an acute illness or a transient lab or other screening procedure findings (e.g., abnormal transient lab evaluation, has taken antimalarials within 2 weeks) may be rescreened or may repeat individual screening procedures at the investigator's discretion. Rescreened subjects should be assigned the same subject number as for the initial screening.

Strategies for Recruitment and Retention

Study subjects will be selected based on the eligibility criteria described in sections 0 and 0. The total target sample size is 72 subjects for part 1 and 324 subjects in part 2. We expect to enroll all subjects for both parts of the study within the first 6 months of the study from August 2022 through February 2023. Subject selection will not be limited based on sex, race, or ethnicity.

As is customary for all studies conducted under the ongoing KEMRI and CDC collaboration, a series of community sensitization measures will be undertaken. Meetings will be coordinated with the local County Health Management Teams and among local community leaders (village chiefs/assistance chiefs) prior to training activities, in addition to the meetings with health facility and community health workers. Community meetings (barazas) will be held in the selected areas to explain the purpose of the study and what will happen as part of the study. During the meetings we will disseminate information about the purpose of the study and the procedures. After community sensitization, children will be recruited from the well child clinic or from communities surrounding the study clinic. Parents/guardians of children potentially eligible for inclusion will be approached and asked whether they would like to learn more about the trial. Interested parents/guardians will have the trial explained, and if interested, the consent process will proceed as described in section 0.

For the year 2 follow-on, 24 new participants will be recruited for the follow-on safety study (Part 1b) using the methods described above. For the part 2 extension, study participants who remain enrolled in the part 2 efficacy study will be invited to participate in the extension study before completing their last visit of the original study.

Costs

There are no costs associated with participation in this trial.

Compensation

Parents/guardians of participants will be reimbursed per visit for their travel expenses per KEMRI guidelines to minimize loss to follow-up and to prevent the family from incurring extra expenses in travel to and from the clinic for the purpose of participating in the study. Participants will receive 500 Kenyan shillings (KShs) per scheduled visit in part 1 and in part 2. In both part 1 and 2, parents will also be provided with partial reimbursement (400 KShs) for sick visits, as it is important that we capture every malaria episode. If participants pre-enrolled at Kogelo Dispensary in part 2 come to Siaya for their IP adminsitration visits (Day 0 and/or Day 168), they will be reimbursed 700 KShs or offered free transport from Kogelo Dispensary. In addition, medication (typically essential drugs at outpatient facilities as listed in the pediatric guidelines) to treat illnesses during the course of the study will be provided to them free of charge by the study.

In the year 2 follow-on study (both part 1b and the part 2 extension), participants will receive 800 Kenyan shillings (KShs) per scheduled visit; parents will also be provided with partial reimbursement (600 KShs) for sick visits, given increased fuel prices in the study area.

COVID-19 Risk Mitigation

Given the ongoing risk posed by COVID-19 in Kenya, this trial will adopt mitigation measures put in place by other trials at the site. Measures have already been put in place to enable social distancing in the study clinic, including making a partition on the veranda of the clinic so that participants can be registered and reviewed in an open-air space. Only one participant will be seen at a given time in a study room. The emergency room has also been modified so that two children could be admitted for overnight observation or care, should the wards in the main hospital be overcrowded with potential COVID-19 patients.

This study will be conducted while strictly adhering to the KEMRI-SERU guidelines (section 3.2.5) on mitigation measures needed to minimize COVID-19 exposure to research participants, staff and the community. [3432] We will also adhere to any new Government, Kenya Ministry of Health (MoH), or KEMRI-SERU directives on COVID-19 control measures which may come as new evidence emerges. All staff will be trained on infection prevention and control (IPC) for COVID-19 based on MoH and WHO guidelines. Study staff and participants will be screened for COVID-19 symptoms or possible exposure before conducting interviews or exams. Should the interviewer determine that a participant has potentially been exposed to or is infected with COVID-19, they will immediately alert the Hospital IPC team. During the conduct of study procedures IPC measures will be observed, including appropriate hand washing with soap and water or use of alcohol-based hand rubs/sanitizers, use of facemasks/gowns, and physical distancing of at least 1.5 meters in a well aerated room or in an open-air space. The study will provide all the necessary materials needed for effective IPC.

STUDY INTERVENTION

Study Interventions(s) Administration

Study Intervention Description

The study intervention involves an initial administration of L9LS SC or placebo SC to all subjects. At six months, subjects in part 2 of the study will receive either a second dose of L9LS (half those in MAb groups) or placebo (other half of those in MAb groups and those in placebo group). During the part 2 extension, participants will receive 2 additional SC doses (either two of L9LS, one of L9LS and one of placebo, or two of placebo), 6 months apart, following their initial randomization.

Dosing and Administration.

Study agent and dosing will be dependent on the progress of the study and study arm assignment (Table 1). Total effective doses by subject weight for part 2 of the study are provided in Table 2 (section 0). The dose escalation study dosing plan is described in section 0. Procedures for administration are described in section 0.

Table 1. Study agent assignment and dosing by age group/arm

Age group/arm	Total Subjects	Study Agent and Dose
**Part 1**		
5 to 10 years	12	5 mg/kg L9LS SC (n=9) or placebo SC (normal saline; n=3)
	12	10 mg/kg L9LS SC (n=9) or placebo SC (normal saline; n=3)
	12	20 mg/kg L9LS SC (n=9) or placebo SC (normal saline; n=3)
5 to 59 months	12	5 mg/kg L9LS SC (n=9) or placebo SC (normal saline; n=3)
	12	10 mg/kg L9LS SC (n=9) or placebo SC (normal saline; n=3)
	12	20 mg/kg L9LS SC (n=9) or placebo SC (normal saline; n=3)
**Part 1b**		
5 to 71 months	12	30 mg/kg L9LS SC (n=9) or placebo SC (normal saline; n=3)
	12	40 mg/kg L9LS SC (n=9) or placebo SC (normal saline; n=3)
**Part 2**		
Arm 1 (5 to 17 months of age)	108	10-20 mg/kg L9LS SC plus second dose of 10-20 mg/kg L9LS SC or second placebo dose at 6 months
Arm 1 (18 to 59 months of age)	108	10-20 mg/kg L9LS SC plus second dose of 10-20 mg/kg L9LS SC or second placebo dose at 6 months
Arm 2 (5 to 59 months of age)	108	Placebo (normal saline) SC plus second dose of placebo at 6 months
**Part 2 Extension (year 2)**		
Arm 1 (from part 2	108*	20-40 mg/kg L9LS SC plus second dose of 20-40 mg/kg L9LS SC at 6 months
Arm 2 (from part 2	108*	20-40 mg/kg L9LS SC plus placebo dose at 6 months
Arm 3 (from part 2	108*	Placebo (normal saline) SC plus second dose of placebo at 6 months

*108 represents the maximum enrollment, as participants might already be lost to follow-up during part 2.

Dose Escalation

In part 1 of the study, dose escalation will proceed in a stepwise fashion in each age-based cohort, with dosing in 5- to 59-month-olds proceeding only after confirmation of safety in the respective 5- to 10-year-old dosing group, as described in section 0 above.

Dose Limiting Toxicity

Pausing and halting rules are provided in sections 0 and 0.

Dose Modifications

Not applicable.

Drug Administration

Prior to study agent administration on day 0, subjects will undergo vital signs measurement and a targeted physical examination (as needed based on signs, reported symptoms, or interim medical history). The assigned study agent will be administered as 1 or 2 SC injections, depending on total dose, in the upper inner thigh, abdomen, or triceps area (1 injection per arm/thigh when 2 injections are required), using proper technique to ensure administration into SC fatty layer and a slow push to minimize discomfort or the excessive distention of overlying skin. The abdomen may be used as an alternate injection site if the outer triceps area or inner thigh is not suitable. SC administration sites in the abdomen should be at least 2 inches apart from each other and at least 2 inches away from the umbilicus. Because L9LS has a slight yellow tint, and the placebo (normal saline) is colorless, all syringes will be covered with transparent yellow tape by the study pharmacist prior to administration to maintain blinding. L9LS is more viscous than normal saline, so the study agents will only be administered to subjects by designated individuals who remain separate from the team of blinded investigators who conduct all subsequent follow-up study assessments.

The first two subjects in each arm of the dose-escalation study in part 1 will be observed for at least 4 hours following completion of product administration, and subsequent subjects will be observed for at least 2 hours following completion of product administration. The first two subjects in each dose group in part 1b will be observed for two hours, and subsequent subjects will be observed for at least one hour following dosing. All subjects in the efficacy study and the part 2 extension will be observed for at least 60 minutes following completion of product administration. Prior to discharge from the clinic, vital signs will be recorded and subjects will be assessed for local reactogenicity (including pain/tenderness, swelling, redness, bruising, and pruritus at the site of injection) and systemic reactogenicity (including fever, feeling unusually tired or unwell, muscle aches, headache, chills, nausea, and joint pain). Any subject who is assessed as being unwell or has ongoing reactogenicity symptoms will be asked to remain in the clinic until evaluation and discharge by a study clinician. If necessary, the subject would be referred to the County hospital to evaluate for safety and possible treatment. Clinicians will follow any solicited adverse events that are ongoing until they have resolved.

Preparation/Handling/Storage/Accountability

Acquisition and Accountability

Acquisition: L9LS and the normal saline placebo will be shipped from the US to the study site where administration will take place, in compliance with all FDA, US Department of Transportation, and United Nations transport guidelines for shipping biohazardous materials.

Accountability: The study pharmacist will be responsible for maintaining an accurate record of the study arm codes, inventory, and an accountability record of study agent supplies. Electronic documentation as well as paper copies may be used.

The empty vials and the unused portion of a vial will be discarded in a biohazard containment bag and incinerated or autoclaved in accordance with the regulatory, institutional or pharmacy policy. Any unopened vials that remain at the end of the study will be returned to the production facility or discarded at the discretion of the manufacturer in accordance with policies that apply to investigational agents. Partially used vials will not be administered to other subjects or used for *in vitro* experimental studies. These vials will be disposed of in accordance with regulatory, institutional or pharmacy policy.

Formulation, Appearance, Packaging, and Labeling

L9LS was manufactured under current Good Manufacturing Practice (cGMP) by the Vaccine Clinical Materials Program (VCMP) operated under contract by Leidos Biomedical Research, Inc., Frederick, MD. It is a sterile, aqueous, buffered solution that is filled into single -dose vials at 150 + 15 mg/mL to a target fill volume of 2.25 mL in a 3-mL vial. The formulation buffer is the same as the drug substance (DS). The drug product container closure system consists of Type I glass vials, chlorobutyl rubber stoppers, and seals purchased from approved manufacturers. Any diluent composition will be described in the IND. The placebo product will be sterile isotonic (0.9%) normal saline. The products will be prepared by an unblinded pharmacist and placed in a sterile syringe when preparing for SC administration. Because L9LS has a slight yellow tint, and the placebo (normal saline) is colorless, the pharmacist will cover all syringes with transparent yellow tape to maintain blinding. L9LS is more viscous than normal saline, so the study agents will only be administered to subjects SC by designated individuals who remain separate from the team of blinded investigators who conduct all subsequent follow-up study assessments.

Vials of L9LS and placebo will be individually labeled with the name of the material, volume, lot number, concentration, storage instructions, Investigational Use Statement ("Limited by Federal Law to Investigational Use"), and manufacturer information.

Product Storage and Stability

L9LS: L9LS vials should be stored frozen at −35°C to −15°C in a qualified, continuously monitored, temperature-controlled freezer. The site pharmacist must promptly report any storage temperature excursions outside of the normal allowance for the storage device to the IND sponsor. The affected product must be quarantined in a separate area under protocol-specific temperature ranges until further notice from the sponsor. If the excursion results in thawed material, DO NOT REFREEZE; store the thawed, vialed material at 2°C to 8°C.

When a storage/shipping/handling excursion occurs, the IND sponsor designee must send a notification of the occurrence of an excursion to VRCProductinquiries@nih.gov. An automatic email

reply will be sent to the notifier, including (as an attachment) the Clinical Excursion Reporting Form, which can be filled electronically (or manually and scanned, if needed). The completed form and relevant attachments (e.g., temperature charts) must be emailed to the VRC via the same email address (VRCProductinquiries@nih.gov) using the “reply” function. The IND sponsor will notify the site pharmacist if continued clinical use of the product is acceptable or will provide further instructions.

Prior to preparation for SC administration, thaw and equilibrate vials for a minimum of 90 minutes at ambient temperature (15°C to 32°C). If thawed vials are removed from 2°C to 8°C, equilibrate at ambient temperature for a minimum of 30 minutes. [3533]

After product preparation in a syringe, the prepared L9LS may be stored at 2°C to 8°C for a maximum of 24 hours and/or at ambient temperature (15°C to 32°C) for a maximum of 4 hours. Product may not be stored in direct sunlight. [3533]

Placebo: Normal saline will be stored at room temperature in a controlled room per product standards.

Preparation

Study product will be prepared by an unblinded pharmacist. For Part 1, dosing will be weight-based and the pharmacist will prepare one or two syringes to deliver doses of 5 mg/kg, 10 mg/kg, and 20 mg/kg. For those receiving 20 mg/kg L9LS, 2 mL syringes will be used so that a maximum of two injection sites will be used for those requiring up to 600 mg L9LS.

For Part 2, three fixed doses of L9LS will be prepared depending on the participant’s weight band, as shown in Table 2. Because L9LS has a slight yellow tint, and the placebo (normal saline) is colorless, the pharmacist will cover all syringes with transparent yellow tape to maintain blinding.

Table 2. Dosing of L9LS (10 to 20 mg/kg) by weight of child for part 2

L9LS 10 to 20 mg/kg		
**Subject weight (kg)**	**Total dose, mg L9LS**	**Effective dose in mg/kg**
5.00-7.50	75	10-15

7.51-15.00	150	10-20
15.01-22.50	225	10-15

Weight data from a household survey in the study area indicated that the mean weight for children between 4 and 5 years = 15.7 kg (boys), with 95% of weights falling below 20 kg; thus, we do not anticipate any children <59 months will weigh >22 kg.

For the extension phase (year 2), dosing will be constrained to be within a 20–40 mg/kg range to test higher average doses for ADA and increase the chances for high efficacy, given the known dose-response relationship with mAbs. Depending on the child's weight, one of three dosing bands will be selected, as shown in **Table 3**.

Table 3. Dosing of L9LS (20 to 40 mg/kg) by weight of child for the extension phase

L9LS Extension 20–40 mg/kgmg/kg		
**Subject weight, kg**	**Total dose**	**Total volume, mL**
6 – 7.4	225	1.5
7.5 – 14.9	300	2.0
15 – 30	600	4.0

Note: given current weights of part 2 participants, no child is expected to weigh more than 30 kg during the extension phase.

For each injection order, the dose level and study arm code will be included in the pharmacy order. To prepare an injection, the pharmacist will follow the IP Preparation SOP.

Measures to Minimize Bias: Randomization and Blinding

Randomization

Randomization lists for each randomized phase of the trial will be generated by the study statistician, and the randomization code lists will be maintained by a designated pharmacist at the study site where the study intervention will take place.

Randomization is further described in section 0.

Blinding

Parts 1 and 2, as well as the follow-on (part 1 b and part 2 extension), of this trial will be conducted with a double blind. Because L9LS has a slight yellow tint, and the placebo (normal saline) is colorless, the pharmacy team will cover all syringes with transparent yellow tape to maintain blinding for all parts of the study. The subjects, the clinical staff, and the study team will be blinded to study treatment allocation, with the exception of designated individuals who administer the study agents and remain separate from the team of blinded investigators who conduct all subsequent follow-up study assessments. The study pharmacy team will be unblinded, and they are responsible for maintaining security of study treatment assignments.

Data will remain blinded until the last subject completes the final study visit for each phase of the study (part 1, part 1b, part 2, and the follow-on). Blinded subjects will then be informed about their study treatment assignment.

Unscheduled unblinding, either intentional (e.g., in the case of a medical emergency in a subject) or unintentional, will be handled according to SOPs. Intentional and unintentional unscheduled unblinding will be documented in the appropriate source and/or research record and will include the reason for the unscheduled unblinding, the date it occurred, who approved the unblinding, who was unblinded, who was notified of the unblinding, and the plan for the subject.

The principal investigator will report all cases of intentional and unintentional unscheduled unblinding to the DSMB in writing within 1 business day after site awareness via email to the DSMB mailbox (niaiddsmbia@niaid.nih.gov) outlining the reason for the unblinding and the date it occurred. The report will also be submitted to the IRBs. If an SAE has resulted in unblinding, this information will be included in the SAE Report.

Study Intervention Compliance

Study intervention administration will be documented by study staff.

Concomitant Therapy

All concomitant prescription and nonprescription (including over-the-counter, herbal, or traditional) medications taken during study participation will be recorded. For this protocol, a prescription medication is defined as a medication that can be prescribed only by a properly authorized/licensed clinician.

Treatment with the following drugs and procedures will not be permitted unless discussed with and approved by the investigator:

- Live or killed vaccines within 2 weeks of study agent administration. (In Kenya there are no routine vaccinations scheduled for 5- to 10-year-old children; see Appendix A)
- Immunoglobulins for the duration of the study.
- Receipt of any investigational product or co-enrollment in other clinical studies of investigational products.
- Oral or IV corticosteroids at immunosuppressive doses (i.e., prednisone >10 mg/day for 6 or more days) or immunosuppressive drugs for the duration of the study.

Patients taking any of the following drugs at the time of enrolment will be excluded due to the potential for prolongation of the QT interval when used concomitantly with DP. These drugs will not be prohibited at other time points in the follow-up period.

- Antimicrobial agents of the following classes (systemic use only):
    - Macrolides (e.g. erythromycin, clarithromycin, azithromycin, roxithromycin)
    - Fluoroquinolones (e.g., levofloxacin, moxifloxacin, sparfloxacin)
    - Pentamidine
- Antiarrhythmic agents (e.g. amiodarone, sotalol)
- Antihistamines (e.g. promethazine)
- Antifungals (systemic): ketoconazole, fluconazole, itraconazole
- Antiretrovirals: Saquinavir
- Diuretics (e.g. hydrochlorothiazide, furosemide)
- Antipsychotics (neuroleptics): haloperidol, thioridazine
- Antidepressants: imipramin, citalopram, escitalopram
- Antiemetics: domperidone, chlorpromazine, ondansetron

STUDY INTERVENTION DISCONTINUATION AND PARTICIPANT DISCONTINUATION/WITHDRAWAL

Discontinuation of Study Intervention

Study intervention may be discontinued for a protocol-defined group or arm (in part 1) or an individual (in part 2) (i.e., pausing), or it may be discontinued for all subjects and enrollment suspended (i.e., halting). Pausing and halting are described in sections 0 and 0. Subjects who have already received the study agent at the time of a pause or halt will continue planned follow-up under the protocol.

Participant Discontinuation/Withdrawal from the Study

Plans for managing the involuntary withdrawal of a subject are provided in section 0. The reason for subject discontinuation or withdrawal from the study will be recorded on the case report form (CRF).

Subjects who withdraw after receiving study agent but prior to study completion will be encouraged to attend an early termination visit, where they will complete as many of the procedures and evaluations indicated in the schedule of activities (section 0) as possible.

Lost to Follow-up

A subject will be considered lost to follow-up if he or she fails to return for 3 consecutive scheduled visits and is unable to be contacted by the study site staff.

The following actions must be taken if a subject fails to return to the clinic for a required study visit:

- The site will attempt to contact the subject/parent/guardian and reschedule the missed visit within 7 days, counsel them on the importance of maintaining the assigned visit schedule, and ascertain if they wish to and/or should continue in the study.
- Before a subject is deemed lost to follow-up, the investigator or designee will make every effort to regain contact with the subject/parent/guardian (where possible, 3 telephone calls and/or home visits). These contact attempts should be documented in the subject's medical record or study file.
- Should the subject/parent/guardian continue to be unreachable, he or she will be considered to have withdrawn from the study with a primary reason of lost to follow-up.

STUDY ASSESSMENTS AND PROCEDURES

Screening and Pre-Enrollment Procedures

At screening, the study staff will explain the study to prospective subjects' parents/guardians, and complete a pre-screening process to determine whether children are eligible based on age, weight, and not taking prohibited medications. For potentially eligible children, study staff will take parents/guardians through the informed consent form and then administer them the study comprehension examination; if they pass and consent (section 0) to participate, study staff will further assess eligibility. Consent will be obtained before any study-related procedures are performed. Once a subject has consented a unique participant identification number (PID) will be assigned that will be used for the duration of the study.

The following screening and pre-enrollment procedures and evaluations must be performed within 21 days of study intervention. Screening may take place over multiple visits if necessary or it could

be combined with the pre-enrolment visit. As long as the physical exam, height and weight measurement, vital signs, medical history, and concomitant medications are done at the pre-enrolment visit, they do not necessarily need to be done at the screening visit, if the two are separate visits.

- Confirmation of identity, age, and residency.
- Complete review of medical history and medication use.
- Complete physical examination, including height and weight.
- Vital signs (temperature and pulse).
- Blood collection via finger prick or venipuncture for screening evaluations:
    - HIV tests: 2 rapid diagnostic tests (RDTs), plus DNA PCR if the RDTs are discordant. A subject will be referred for medical care for 2 positive RDTs or a positive PCR. If the PCR is negative, no further work-up will be done. Pre and post-test HIV counseling will be provided.
    - Hemoglobin typing.
    - Complete blood count (CBC) with differential.
    - ALT.
    - Cr.
- Pre- and post-test HIV counseling.

For children who are still breastfeeding, their child health booklet will be checked for the mother's HIV status. If the mother is HIV-positive and the child is not taking cotrimoxazole, the child will be referred for cotrimoxazole prophylaxis according to Kenyan MoH guidelines and excluded from the study. If the mother had a negative HIV test more than six months prior to the current date or no documented HIV test, the mother will be referred for HIV testing, per Kenyan MoH guidelines. If the mother refuses HIV testing, the child will still be enrolled. However, if study investigators subsequently become aware that the mother tested positive for HIV and the child is still breastfeeding (or was breastfeeding when the mother could have potentially transmitted the virus), the child will be referred for cotrimoxazole prophylaxis and followed for safety but no additional study products will be administered.

A prospective subject who has any clinically significant abnormal finding and/or is diagnosed with a medical condition at screening or during the conduct of the study will be notified and referred for medical care. Per national requirements for reporting communicable diseases, positive test results for HIV will be reported to the local health department according to applicable laws and appropriate medical referrals initiated. The cost of initial and long-term treatment and care of medical conditions diagnosed during the screening process will not be reimbursed by the study but referrals to relevant specialist will be provided.

Screening evaluations may be repeated as described in section 0, at the discretion of the investigator. If screening is completed outside the specified window, all screening procedures and evaluations must be repeated. If an individual screens and is enrolled into part 1 of the study but for any reason does not receive study agent, they may later consent to be screened and enrolled in part 2; otherwise, participation in part 1 is exclusionary for part 2. If the subject is still eligible at the end of the pre-enrolment visit, he/she will be given a three-day course of DP, directly observed each day, and a Day 0 (Enrolment) clinic appointment will be fixed for 14 to 21 days after the first dose of DP.

Enrollment: If the individual is eligible and agrees to participate, has completed a three-day course of DP, and returns 14 to 21 days after DP administration for their Day 0 (Enrolment) visit, he or she will be randomized to study product or placebo and will be considered enrolled. Enrollment is defined as the time of randomization, immediately after which the subject will receive IP/placebo. The identification number will link subject samples and data collected throughout the study.

Efficacy Assessments

Clinical Evaluations

The following clinical evaluations will be performed as efficacy assessments.

Medical History and Medication Review: A complete review of all medical history and medications will be conducted at screening. Subsequent visits will include a targeted review of changes in medical history or medications since the last study visit.

Physical Examination: A complete physical examination (including height and weight) will be done at screening. A targeted physical examination based on signs, reported symptoms, and medical history will be conducted at subsequent study visits. Weight will also be recorded on day 0 prior to study agent administration.

DP: At the end of the pre-enrollment visit, all subjects will be orally administered standard doses of DP presumptively to clear any possible Pf blood-stage infection prior to study agent administration. The dosing will be once per day and by weight as shown in Table 3.

Table 3. Dosing schedule for dihydroartemisinin piperaquine

Body Weight	Dihydroartemisinin	Piperaquine	Number of tablets (160 mg/20 mg)	Number of tablets (320 mg/40 mg)
5 to <8 kg	20 mg	160 mg	1 tablet	½ tablet
8 to <11 kg	30 mg	240 mg	1.5 tablets	¾ tablet

11 to <17 kg	40 mg	320 mg	2 tablets	1 tablet
17 to < 25 kg	60 mg	480 mg	3 tablets	1.5 tablets
25 to <36 kg	80 mg	640 mg	4 tablets	2 tablets

Randomization Procedures: Randomization is described in sections 0 and 9.4.1.

Study Agent Administration and Monitoring: Study agent will be administered according to the assigned arm and monitoring will be performed as described in section 0.

Illness Visit: A subject will be instructed to come in for an unscheduled visit if he or she has symptoms of malaria or other symptoms. The subject will be evaluated by the study team and treated according to local guidelines. At an illness visit, the subject may undergo review of medical history and concomitant medications, a focused physical exam for symptoms of malaria or other diseases, vital sign measurement, and a fingerprick/heelprick blood collection for blood smear for malaria diagnosis as well as a dried blood spot for Pf RT-PCR for research purposes.

Malaria Diagnosis and Management: If a subject has a malarial infection, we will share these results with the subject and provide standard treatment in accordance with the recommendations of the Kenyan Division of National Malaria Program guideline. RT-PCR is not commonly used for routine malaria diagnosis.

Biospecimen Evaluations

Blood will be collected under this protocol by the following methods:

- Venipuncture will be performed with single-use needles. Venous blood samples will be used as follows:
  - Safety evaluations described in section 0.
  - Shipment to the research laboratories in Kenya and the US for evaluation (including assays described in section 0) and storage.
  - Blood smear and dried blood spot for Pf RT-PCR if unable to obtain ample sample from finger prick.
- Finger or heel prick will be performed using single-use disposable lancets. Finger/ heel prick blood samples will be used as follows:
  - Blood smear and dried blood spot for Pf RT-PCR.
  - Biochemical and hematologic tests for safety described in section 0.

The amount of blood drawn for research purposes will be within the limits allowed for research subjects by the NIH Clinical Center: no more than 5 mL/kg in a single day, and no more than 9.5 mL/kg over any 8-week period.

The collection schedule, volumes, and test tubes are presented in section 0.

Correlative Studies for Research/Pharmacokinetic Studies

The following evaluations will be performed according to the schedule presented in section 0.

Blood smear: Thick and thin blood smears will be prepared and analyzed by the standard WHO method (section 0) to identify Pf infection for the primary endpoint and to identify clinical malaria for a secondary endpoint. This evaluation will be performed within 48 hours of collection, unless the subject is displaying signs or symptoms of clinical malaria in which case the blood smear will be analyzed at the time of the scheduled or unscheduled visit. For asymptomatic participants with positive blood smears, a home visit will be conducted within 72 hours of the blood draw to notify the family and provide treatment.

Pf RT-PCR: RT-PCR will be performed to identify Pf infection as a secondary endpoint. This evaluation will be performed at the University of Washington in the US on coded dried blood spots. Real-time PCR might also be performed by KEMRI on the dried blood spots.

PK studies: Blood L9LS concentrations will be measured by Meso Scale Discovery LLC-based automation platform. The concentration at the visit prior to the first Pf infection will be used to assess L9LS-mediated protection. This evaluation will be performed at the NIAID.

ADA detection: Assays for detection of ADA will be performed at specified timepoints (section 0) following product administration and compared to baseline status. This evaluation will be performed at the NIAID.

Parasite genotyping: For subjects who become infected during the study, coded blood samples collected around the time of the first infection will be used to perform a genotypic sieve analysis to analyze sequences of breakthrough parasites (section 3.4). This evaluation will be performed by Dr. Daniel Neafsey at the Harvard School of Public Health.

Pf-specific antibodies: Levels of antibodies specific for pre-erythrocytic and blood-stage Pf antigens will be measured by NIH in stored serum samples collected at enrollment and at one of

the subsequent blood draws to explore the impact of L9LS on naturally acquired humoral immunity to malaria.

Samples for Genomic Analysis

Description of the Scope of Genomic Analysis

Genetic testing performed in this protocol will be limited to analyzing the genetic material of infection-inducing parasites in blood samples. No human genetic analyses will be performed.

Description of How Privacy and Confidentiality of Medical Information/Biological Specimens Will Be Maximized

Privacy and confidentiality of medical information and biological samples is described in section 0.

Management of Results

As no human genetic analyses will be performed in this protocol, no genetic results will be returned to subjects.

Genetic Counseling

Not applicable.

Safety and Other Assessments

The following study procedures and evaluations will be done according to the schedule in section 0 to monitor safety and support the understanding of the study intervention's safety. The assessment and collection of safety events such as AEs are described in section 0.

Physical Examination: As described in section 0, physical examination will also be performed for assessment of safety.

Vital Signs: Vital signs (temperature and heart rate) will be collected at visits, including before and after study agent injection, as described in section 0.

Safety Blood Laboratory Evaluations: The following safety laboratory evaluations will be performed at a frequency presented in section 0:

- CBC with differential.
- ALT, Cr.

Safety Definitions, Management, and Sponsor Reporting

Definitions

Adverse Event: An AE is any untoward or unfavorable medical occurrence in a human subject, including any abnormal sign (e.g., abnormal physical exam or laboratory finding), symptom, or

disease, temporally associated with the subject's participation in the research, whether or not considered related to the research.

Adverse Reaction: An AR means any AE caused (see "Causality" below) by a study agent. ARs are a subset of all suspected adverse reactions (SARs; defined below) where there is reason to conclude that the study agent caused the event.

Suspected Adverse Reaction: SAR means any AE for which there is a reasonable possibility that the study agent caused the AE.

Per US FDA guidance:

For the purposes of IND safety reporting, "reasonable possibility" means there is evidence to suggest a causal (see "Causality" below) relationship between the study agent and the AE. A SAR implies a lesser degree of certainty about causality than an AR, which means any AE caused by a study agent.

SARs are the subset of all AEs for which there is a reasonable possibility that the study agent caused (see "Causality" below) the event. Inherent in this definition, and in the requirement to report SARs, is the need for the sponsor to evaluate the available evidence and make a judgment about the likelihood that the study agent actually caused the AE.

The sponsor is responsible for making the causality judgment.

Serious Adverse Event (SAE):

- is an AE that results in death.
- is an AE that is a life-threatening event (places the subject at immediate risk of death from the event as it occurred).
- is an AE that requires inpatient hospitalization or prolongs an existing hospitalization.
  NOTE:
    - Hospitalization is considered required if outpatient treatment would generally be considered inappropriate.
    - Same-day surgical procedures that are required to address an AE are considered hospitalizations, even if they do not involve an overnight admission.
    - Hospitalization due to a condition that has not worsened and that pre-dates study participation (e.g., elective correction of an unchanged baseline skin lesion), or due to social circumstance (e.g., prolonged stay to arrange aftercare), or that is

planned/required “per protocol” AND that proceeds without prolongation or complication, is NOT considered an SAE by this criterion.

- is, or results in, a persistent or significant incapacity or substantial disruption of the ability to conduct normal life functions.
- is a medically important event.

  NOTE: Medical and scientific judgment should be exercised. Events that significantly jeopardize the subject and/or require intervention to prevent one of the SAE outcomes listed above are generally considered medically important, and are thus SAEs.

Unexpected Adverse Event: An AE is unexpected if it is not listed in the investigator’s brochure or package insert (for marketed products) at the frequency, AND specificity, AND severity that has been observed.

NOTE:

- Such events should also be evaluated for possible reporting as unanticipated problems (UPs) (see section 0 below).
- Unexpected, as used in this definition, also refers to AEs or SARs that are mentioned in the investigator’s brochure as occurring with a class of drugs/biologics, or as anticipated from the pharmacological properties of the study agent but are not specifically mentioned as occurring with the particular study agent under investigation.

Serious and Unexpected Suspected Adverse Reaction (SUSAR): A SUSAR is an SAR (defined above) that is both serious and unexpected.

Unanticipated Problem: A UP is any incident, experience, or outcome that meets all the following criteria:

1. Unexpected (in terms of nature, severity, or frequency) given
   a. the research procedures that are described in the protocol-related documents, such as the IRB-approved research protocol and informed consent document; and
   b. the characteristics of the subject population being studied; and
2. Related or possibly related to participation in the research (possibly related means there is a reasonable possibility that the incident, experience, or outcome may have been caused by the procedures involved in the research), and
3. Suggests the research places subjects or others (which may include research staff, family members or other individuals not directly participating in the research) at a

greater risk of harm (including physical, psychological, economic, or social harm) related to the research than was previously known or expected.

NOTE:

- Per the sponsor, an SAE always meets this “greater risk” criterion.
- An incident, experience, or outcome that meets the definition of a UP generally will warrant consideration of changes to the protocol or informed consent form, or to study procedures (e.g., the manual of procedures for the study), in order to protect the safety, welfare, or rights of participants or others. Some UPs may warrant a corrective and preventive action plan at the discretion of the sponsor or other oversight entities.

Unanticipated Problem that is not an Adverse Event (UPnonAE): A UPnonAE belongs to a subset of UPs that:

- meets the definition of a UP, AND
- does NOT fit the definition of an AE or an SAE.

NOTE: Examples of UPnonAEs include, but are not limited to:

- a breach of confidentiality
- subject departure from an isolation unit prior to meeting all discharge criteria
- accidental destruction of study records
- unaccounted-for study agent
- overdosage, underdosage, or other significant error in administration or use of study agent or intervention, even if there is no AE/SAE
- development of an actual or possible concern for study agent purity, sterility, potency, dosage, etc.

  NOTE: A decision to temporarily quarantine, or to permanently not use all or part of study agent supply due to an unexpected finding or event (e.g., particulate, cloudiness, temperature excursion), even if there is no known or proven issue (i.e., out of an “abundance of caution”), is considered a UPnonAE.

Protocol Deviation: Any change, divergence, or departure from the IRB-approved research protocol.

1. **Major deviations:** Deviations from the IRB-approved protocol that have, or may have the potential to negatively impact the rights, welfare, or safety of the subject, or to substantially negatively impact the scientific integrity or validity of the study.
2. **Minor deviations:** Deviations that do not have the potential to negatively impact the rights, safety, or welfare of subjects or others, or the scientific integrity or validity of the study.

Noncompliance: Failure of investigator(s) to follow the applicable laws, regulations, or institutional policies governing the protection of human subjects in research, or the requirements or determinations of the IRBs, whether intentional or not.

1. **Serious noncompliance:** Noncompliance, whether intentional or not, that results in harm or otherwise materially compromises the rights, welfare and/or safety of the subject. Noncompliance that materially affects the scientific integrity or validity of the research may be considered serious non-compliance, even if it does not result in direct harm to research subjects.
2. **Continuing noncompliance:** A pattern of recurring noncompliance that either has resulted, or, if continued, may result in harm to subjects or otherwise materially compromise the rights, welfare and/or safety of subjects, affect the scientific integrity of the study or validity of the results. The pattern may comprise repetition of the same non-compliant action(s), or different noncompliant events. Such non-compliance may be unintentional (e.g., due to lack of understanding, knowledge, or commitment), or intentional (e.g., due to deliberate choice to ignore or compromise the requirements of any applicable regulation, organizational policy, or determination of the IRB).

Documenting, Assessing, Recording, and Reporting Events

ALL AEs, including those that may appear to have a non-study cause (see "Causality" below), will be documented (e.g., on the clinical chart/progress notes/clinical laboratory record), recorded (e.g., in the study-specified CRF/research database), and reported (e.g., cumulatively from the research database, or according to protocol-specified expedited reporting mechanism) to the sponsor from the time informed consent is obtained through the timeframe specified below. At each contact with the subject, information regarding AEs will be elicited by open-ended questioning and examinations.

AEs and SAEs will generally be recorded, assessed, and reported according to the timeframes outlined in Table 4.

Table 4. Standard event recording, assessment, and reporting timeframes.

Event type	Record, assess, and report through
Related SAEs	End of subject participation in study, or if study personnel become aware thereafter
Unrelated SAEs	End of subject participation in study
Non-serious AEs of grade 3 or higher, irrespective of relatedness	End of subject participation in study
All malaria episodes, whether related or not, including asymptomatic parasitemias, irrespective of seriousness or severity	End of subject participation in study
Related non-serious AEs of grade 1 and 2	End of subject participation in study
Unrelated non-serious AEs of grade 1 and 2	End of subject participation in study in part 1 and part 1b; 28 days following IP dosing in part 2 and part 2 extension

Investigator Assessment of Adverse Events

The investigator will assess all AEs with respect to **seriousness** (according to SAE definition above), **severity** (intensity or grade, see below), and **causality** (relationship to study agent and relationship to participation in the research, see below).

Severity Grading

The investigator will grade the severity of fever (by non-oral temperature reading) and each blood laboratory testing AE according to the “Adverse Event Grading Scale” (see Appendix B).

Events that are not gradable using the “Adverse Event Grading Scale” (e.g. seizures) will be graded according to the Common Toxicity Criteria for Cancer Research table version 2.0 (https://ctep.cancer.gov/protocoldevelopment/electronic_applications/docs/ctcv20_4-30-992.pdf)

Events that are NOT gradable using either of the above specified tables will be graded as follows:

- Mild = grade 1
- Moderate = grade 2
- Severe = grade 3
- Potentially life threatening = grade 4
- Death = grade 5

NOTE: A subject death should always be reported as grade 5.

Laboratory Value Assessment and Clinical Significance Criteria

ALL abnormal lab values of grade 3 or above are REPORTABLE **as AE or SAE (if applicable, i.e. if the child needs admission or other SAE criteria are fulfilled)**.

Grade 1 and 2 abnormal laboratory values will be considered CLINICALLY SIGNIFICANT, and will be recorded in the research database, and reported, ONLY if they meet ONE or more of the following criteria:

- result in a study agent dosage adjustment, interruption, or discontinuation
- are accompanied by clinically abnormal signs or symptoms that are likely related to the laboratory abnormality (e.g., clinical jaundice)
- indicate a possible organ toxicity (e.g., elevated serum creatinine [Cr])
- result in additional/repeat testing or medical intervention (procedures/treatments) (e.g., electrolytes have to be repeated after potassium supplementation for hypokalemia)
- are considered clinically significant by the investigator or SMM

Causality

Causality (likelihood that the event is caused by the study agents) will be assessed by the principal investigator or designee considering the factors listed under the following categories:

Definitely Related

- reasonable temporal relationship
- follows a known response pattern
- clear evidence to suggest a causal relationship
- there is no alternative etiology

Probably Related

- reasonable temporal relationship
- follows a suspected response pattern (based on similar agents)
- no evidence of a more likely alternative etiology

Possibly Related

- reasonable temporal relationship
- little evidence for a more likely alternative etiology

Unlikely Related

- does not have a reasonable temporal relationship

  AND/OR

- there is good evidence for a more likely alternative etiology

Not Related

- does not have a temporal relationship

  AND/OR

- definitely due to an alternative etiology

Note: Other factors (e.g., dechallenge, rechallenge, if applicable) should also be considered for each causality category when appropriate. Causality assessment is based on available information at the time of the assessment of the AE. The investigator may revise the causality assessment as additional information becomes available.

Causality assessment will be reviewed by the sponsor. The sponsor may make a separate and final determination on the “reasonable possibility” that the event was “related” (comprising definitely, probably, and possibly related) or “unrelated” (comprising unlikely and not related) to the study agent, in keeping with applicable (US FDA) guidance on sponsor IND safety reporting.

Recording of Events

AEs will be promptly recorded in the research database, regardless of possible relationship to study interventions. If a diagnosis is clinically evident (or subsequently determined), the diagnosis rather than the individual signs and symptoms or laboratory abnormalities will be recorded as the AE. The investigator will review events regularly to ensure they have been captured correctly and to perform assessment of events individually and cumulatively to assess possible safety trends.

Investigator Reporting Responsibilities

The principal investigators and/or equally qualified designees will check daily for events that may require expedited reporting.

The principal investigators and/or equally qualified designees will also monitor all accumulating data no less than weekly, or according to superseding NIH or NIAID policy, whichever is more frequent.

Data will be reviewed by the principal investigators/designees on a regular basis for accuracy and completeness.

Data will be submitted to the sponsor in keeping with all applicable agreements and when requested, such as for periodic safety assessments, review of IND annual reports, review of IND safety reports, and preparation of final study reports.

The principal investigators and/or other study designee will ensure prompt reporting to safety oversight bodies (e.g., Clinical Safety Office [CSO], ISM, DSMB), regulatory entities, and stakeholders as specified below, and according to any additional requirements or agreements.

Adverse Events

Unless otherwise specified above, AE data will be entered into the research database no less than every other week and will include all data through 1 week prior to database entry. AEs will be reported in the form of a line list and sent periodically to the different IRBs/ regulatory authorities.

Serious Adverse Events (Expedited Reporting)

Unless otherwise specified above, all SAEs (regardless of relationship and whether or not they are also UPs) must be reported to the CSO as specified by the CSO (e.g., Research Electronic Data Capture [REDCap] system; use the Safety Expedited Report Form [SERF]/email if REDCap is not available). If the preferred/indicated mechanism for reporting is not available, the CSO/SMM should be contacted by telephone, fax, or other reasonable mechanism to avoid delays in reporting.

CSO CONTACT INFORMATION:

Clinical Safety Office

5705 Industry Lane
Frederick, MD 21704
Phone: 301-846-5301
Fax: 301-846-6224
Email: rchspsafety@mail.nih.gov

https://crimsonredcap.cc.nih.gov/redcap/index.php

Unless otherwise specified above, deaths, related SAEs, and immediately life-threatening SAEs must be reported to the CSO promptly, and no later than the **first business day** following the day of study personnel awareness. All other unrelated SAEs must be reported to the CSO no later than the **third business day** following the day of study personnel awareness and to SERU within 48 hours of the PI becoming aware of the SAE.

KEMRI SERU Reporting

Unless specified, all SAEs regardless of whether related or unrelated must be reported to the SERU via email (seru@kemri.org/kemriseru18@gmail.com) within **twenty-four (24) hours** after the PI becomes aware of the event. Follow-up reports should be submitted as soon as more information becomes available.

Kenya Pharmacy and Poisons Board (KPPB) Reporting

KPPB reporting follows the Guidelines for the Conduct of Clinical Trials in Kenya https://pharmacyboardkenya.org/files/?file=Guidelines%20for%20Conduct%20of%20Clinical%20Trials%20in%20Kenya%202nd%20Revision.pdf (Section 16): All SAEs (including life-threatening and fatal SUSARs) are to be reported to KPPB within 7 days via an online portal at http://www.ctr.pharmacyboardkenya.org/

All SAEs are also submitted to KEMRI SERU and KPPB in a line list during continuation approval requests.

If an individual subject experiences multiple SAEs in a closely timed/overlapping "cause-and-effect" (cascade) sequence, the principal investigators, after careful evaluation, will report ONLY primary/precipitating event(s) individually. SAEs that are determined to be definitely secondary to other SAEs will be detailed in the narrative portion of the report of the relevant primary/precipitating SAE. A clinical rationale and findings to support such reporting should be part of the narrative.

For each SAE report, the research database entry MUST match the corresponding entries on the SAE report (e.g., start and stop dates, event type, relationship, and grade), and **must be updated if necessary** (e.g., if the SAE report was generated after the corresponding AE was entered in the research database).

Unless otherwise specified above, SAEs that have not resolved by the end of the per-protocol follow-up period for the subject are to be followed until final outcome is known (to the degree permitted by the IRB-approved informed consent form). If it is not possible to obtain a final outcome for an SAE (e.g., the subject is lost to follow-up), and to update the CSO, the last known status and the reason a final outcome could not be obtained will be recorded by the investigator on an SAE report update and the CRF.

Unanticipated Problems

Unless otherwise specified above, UPs (as defined in this protocol, or as defined by the IRB[s] of record, whichever definition is more conservative) that are also AEs or SAEs, must be reported to

the CSO (by REDCap, or by email and SERF if REDCap is not available) no later than when they are due to be reported to the IRB(s).

UPnonAEs are NOT reported to the CSO but must be reported to the Clinical Trials Management (CTM) group according to their requirements and preferred methods. If the UPnonAE raises a significant potential subject safety concern, the SMM should be consulted by email or phone no later than when reports are made to the CTM.

Sponsor's Reporting Responsibilities

Events reported to the sponsor will be promptly evaluated and will be reported as required according to FDA IND safety reporting guidance and regulations. IND safety reports will be sent to other investigators conducting research under the same IND and will be shared with other stakeholders according to applicable agreements.

The sponsor will also submit an IND annual report of the progress of the investigation to the FDA as defined in 21 CFR 312.33.

All UPs will be evaluated by the sponsor, and a summary of the event, and any necessary (corrective/preventative) actions, will be distributed to investigators conducting research under the same IND as may be relevant and appropriate.

Withdrawal Criteria for an Individual Subject

An individual subject will be withdrawn from the study for any of the following:

- An individual subject's/parent's/guardian's decision. (The investigator should attempt to determine the reason for the decision if the participant guardian is willing to disclose the reason.)
- Non-compliance with study procedures to the extent that it is potentially harmful to the subject or to the integrity of the study data.
- A change in the subject's condition as follows:
    - Loss of the ability of the subject/parent/guardian to provide informed consent.
- The investigator determines that continued participation in the study would not be in the best interest of the subject.

Re-enrollment and Unplanned Procedure Repetition

Unless otherwise specified within this protocol (e.g., rescreening as described in section 0), each person who is a subject in this study may be enrolled and may pass through each step and process outlined in the protocol, only **ONCE** (i.e., subjects may not "go back" and repeat a protocol step already completed). On a case-by-case basis, a request for re-enrollment, or for repetition of a protocol step or procedure already completed, may be submitted to, reviewed by, and approved by

the SMM in writing. The SMM may also recommend or require consultation of the IRBs, DSMB, and/or ISM.

Replacement of Withdrawn Subjects or Subjects Who Discontinue Study Agent

In the dose-escalation study, subjects withdrawn prior to the day 7 safety evaluation will be replaced. In the efficacy study, subjects withdrawn prior to study agent administration will be replaced. Subjects who are withdrawn prior to or during the year 2 extension study will not be replaced.

All subjects exposed to study agents MUST be included in the safety dataset.

Additional Safety Oversight

Safety Review and Communications Plan

A safety review and communication plan (SRCP) is required for this protocol. The SRCP is an internal communications document between the principal investigators and the CSO, as the sponsor representative, which delineates key safety oversight responsibilities of the principal investigators, the CSO, and other stakeholders. The SRCP includes a plan for conducting periodic safety surveillance assessments by the CSO.

Sponsor Medical Monitor

An SMM, representing the sponsor, has been appointed for oversight of safety in this clinical study. The SMM will be responsible for performing safety assessments as outlined in the SRCP.

Oversight Committees

Independent Safety Monitor in Kenya

The ISM is an expert who does not have direct involvement in the conduct of the study and has no significant conflicts of interest as defined by NIAID policy. An ISM in Kenya will review the study prior to initiation and will be available to advise the investigators on study-related medical issues and act as a representative for the welfare of the subjects. The ISM will conduct independent safety monitoring. The ISM is an expert in the oversight of clinical trials conducted in Kenya and in pediatrics, specifically in the population under study in Kenya.

All deaths, SAEs, UPs, and FDA IND safety reports will be reported by the principal investigators to the ISM prior to or at the same time they are submitted to the IRBs or CSO unless otherwise specified herein. The ISM will be notified immediately if any pausing rule is met. The principal investigators will also notify the ISM if intentional or unintentional unblinding occurs. If the ISM is unblinded to the study agent given to an individual subject during medical management, the ISM will report that unblinding to the DSMB Executive Secretary. The ISM will not have access to unblinded data reported to the DSMB.

Data and Safety Monitoring Board

The NIAID intramural DSMB includes independent experts that do not have direct involvement in the conduct of the study and have no significant conflicts of interest as defined by NIAID policy. The

DSMB will review the study protocol, consent documents, and investigator brochure prior to initiation and twice a year thereafter, or as may be determined by the DSMB. Additionally, the DSMB will conduct 1 interim analysis when safety data are available from the dose-escalation study subjects (section 3.5).

The DSMB may convene additional reviews as necessary. The DSMB will review the unblinded study data as needed to evaluate the safety, efficacy, study progress, and conduct of the study.

All deaths, SAEs, UPs, and IND safety reports will be reported to the DSMB at the same time they are submitted to the IRBs and CSO unless otherwise specified herein.

All cases of intentional or unintentional unblinding will be reported to the DSMB not later than 1 business day from the time of study personnel awareness.

The principal investigators will notify the DSMB at the time pausing or halting criteria are met and obtain a recommendation concerning continuation, modification, or termination of the study.

Pausing Rules

"Pausing" is a temporary discontinuation of study intervention/treatment/dosing (agent/placebo/procedure, etc.) in a protocol-defined group or "arm" (in part 1 or part 1b) or an individual (in part 2 and the follow-on) until a decision is made to either resume or permanently discontinue such activity. Subjects continue to be followed for safety during a pause.

The pausing criteria in this study include the following:

- A subject experiences **2 AEs of grade 3** or higher that are unexpected (per the investigator's brochure or product label) and possibly, probably, or definitely related to a study agent.

The principal investigators or the CSO may also pause dosing/study interventions for one or more subjects for any safety issue. The study safety oversight bodies (e.g., DSMB, ISM) may recommend a pause to the CSO.

Reporting a Pause

If a pausing criterion is met, a description of the AE(s) or safety issue must be reported by the principal investigators within 1 business day to the CSO and the IRBs according to their requirements. The principal investigators will also notify the DSMB and ISM. In addition, the CSO or designee will notify all other site investigators by email or through the specified pathway.

Resumption Following a Pause

The CSO, in collaboration with the principal investigators, the DSMB, and the ISM, will determine if study activities, including agent administration and/or other study interventions may be resumed, and any additional modifications or requirements that may apply, for the impacted subject(s), or whether the events that triggered the pause require expansion to a study halt (see below).

The CSO or sponsor designee will notify the principal investigators of the decision. The principal investigators will notify the IRBs of the decision according to the IRBs' processes.

Discontinuation of Study Agent

If a decision is made to permanently discontinue study agent administration, any subject who has received the study agent or undergone any other study intervention/treatment will continue to be followed for protocol-specified safety assessments or as clinically indicated, whichever is more conservative.

Halting Rules for the Protocol

"Halting" is discontinuation of study intervention/treatment/dosing (agent/placebo/procedure, etc.) for all subjects in a study and suspension of enrollment until a decision is made to either resume or permanently discontinue such activity. Subjects continue to be followed for safety during a halt.

The halting rules are:

- PART 1 AND PART 1B: **Two or more subjects** experience the same or similar SAEs that are unexpected and possibly, probably, or definitely related to a study agent; OR ONE subject experiences an SAE of **Grade 4 severity** or greater that is unexpected (per the investigator's brochure or product label) and possibly, probably, or definitely related to a study agent; OR
- PART 2 AND THE EXTENSION PHASE: **Three or more subjects** experience the same or similar SAEs that are unexpected and possibly, probably, or definitely related to a study agent; OR ONE subject experiences an SAE of **severity 4** or greater that is unexpected (per the investigator's brochure or product label) and possibly, probably, or definitely related to a study agent;

  OR

- Any safety issue that the principal investigators or the CSO determines should halt the study (in Parts 1, 1b and 2 and the follow-on). The study safety oversight bodies (e.g., DSMB) may recommend a halt to the CSO.

In addition, the FDA, IRBs, or any regulatory body having oversight authority may halt the study at any time. The DSMB or ISM may recommend a study halt.

Reporting a Study Halt

If a halting criterion is met, a description of the AE(s) or safety issue must be reported by the principal investigators, within 1 business day to the CSO and the IRBs according to their requirements. The principal investigators will also notify the DSMB and ISM. In addition, the CSO or designee will notify all other site investigators by email or through the specified pathway.

Resumption of a Halted Study

The CSO, in collaboration with the principal investigators and DSMB and ISM will determine if study activities, including enrollment, study agent administration, and/or other study interventions, may be resumed and any additional modifications or requirements that may apply.

The CSO or sponsor designee will notify the principal investigators of the decision. The principal investigators will notify the IRBs of the decision according to the IRBs' processes.

Discontinuation of Study Agent

If a decision is made to permanently discontinue study agent administration, any subjects who have received study agent or undergone any other study intervention will continue to be followed for protocol-specified safety assessments or as clinically indicated, whichever is more conservative.

Unanticipated Problems

Definition of Unanticipated Problems

The definition of a UP is provided in section 0.

Unanticipated Problem Reporting

The investigator will report UPs to the IRBs as described in section 0.

ADDITIONAL REPORTING REQUIREMENTS

Protocol Deviation Reporting

Reporting to NIAID

The following will be reported within 7 calendar days of any investigator or individual associated with the protocol first becoming aware:

- Actual or suspected noncompliance. Determinations by the IRB(s) of serious and/or continuing non-compliance by an NIH investigator must also be reported to the NIH within 7 calendar days.
- Actual or suspected major deviation.
- Actual or suspected UPs.
- New information that might affect the willingness of a subject to enroll or remain in the study.

- Suspension or termination of research activities, including holds on new enrollment, placed upon the research by the sponsor, NIH or NIAID leadership, or any regulatory agency.

Any death of a research subject that is possibly, probably, or definitely related to the research must be reported within 24 hours of the investigator becoming aware of the death.

Additionally, investigators must provide the following information to the IRBs in summary format at the time of continuing review, or when otherwise specifically requested by the IRBs or the NIH Office of Human Subjects Research Protections Office of Compliance and Training:

- Major and minor protocol deviations.
- Noncompliance reported to the IRB(s) that is not related to a protocol deviation.
- AEs and SAEs that do not meet the definition of an UP.
- UPs reported to the IRB(s).

Reporting to the NIAID Clinical Director

The protocol chair will report UPs, major protocol deviations, and deaths to the NIAID clinical director according to institutional timelines.

Reporting Protocol Deviations that Result from the COVID-19 Pandemic

The following addresses the reporting requirements to the IRBs with regard to protocol deviations that result from disruption of study visits from the COVID-19 pandemic. These requirements follow the direction of the IRBs as well as the NIH reporting requirements.

Investigators may modify the protocol without prospective IRB approval when necessary to prevent an immediate apparent harm to a study subject. Typically, when this occurs the event must be reported to the IRB(s) via a Reportable Event Form (REF) within 7 days of the deviation. Given the potential need for this to occur on a much larger than usual scale, it is not required that all planned deviations be reported to the IRBs in an expedited timeframe. Only those deviations which meet the definition of a major deviation will require reporting, as defined in section 0.

If a subject cannot complete a protocol-specified study visit or intervention, the principal investigator should assess the impact of the missed visit on the safety of the subject and the scientific validity of the trial. If in the principal investigator's determination neither of these are meaningfully impacted by the deviation, these do not need to be reported to the IRBs in an expedited manner. The event should be included in the summary of events reported at the time of continuing review.

If in the opinion of the principal investigator the missed visit or intervention poses a risk to the safety of the subject, the investigator should develop a plan to minimize the impact of the deviation. For example, if the subject is scheduled to return to the study site for safety lab work, the investigator may arrange for labs to be drawn at a location closer to the subject's home. In cases such as this, if the change is necessary to assure the safety of the subject, the investigator may implement the change without prospective IRB approval. If the change meets the definition of a major deviation, it must be reported via a REF within 7 days.

STATISTICAL CONSIDERATIONS

Statistical Hypothesis

The primary study hypotheses are that L9LS will be safe and will confer protection against Pf infection. In the dose-escalation study, the primary objective is to evaluate the safety and tolerability. In the efficacy study, the primary objective is to evaluate the efficacy of L9LS compared to placebo.

Primary Endpoints:

1. Part 1 and Part 2: Incidence and severity of local and systemic AEs occurring within 7 days after the administration of L9LS, and incidence of SAEs throughout the study period.
2. Part 2: Pf blood-stage infection as detected by microscopic examination of thick blood smear for 52 weeks after administration of L9LS or placebo.

Secondary Endpoints:

Data from both parts of the study will be used to assess the following endpoints:

1. Pf blood-stage infection as detected by microscopic examination of thick blood smear for 12 and 24 weeks after administration of L9LS or placebo
2. Pf blood-stage infection as detected by RT-PCR for 24 and 52 weeks after administration of L9LS or placebo.
3. Incidence of clinical malaria (definition 1, see section 0) for 24 and 52 weeks after administration of L9LS or placebo.
4. Incidence of clinical malaria (definition 2, see section 0) for 24 and 52 weeks after administration of L9LS or placebo.
5. To assess the primary objective and first four secondary objectives among children 5-17 months of age.
6. To assess the primary objective and first four secondary objectives among children 18-59 months of age.
7. Measurement of L9LS in sera of recipients.
8. PK analysis of L9LS and the association of L9LS concentration with Pf infection risk.

9. PK analysis of L9LS and the association of L9LS concentration with clinical malaria risk.

Sample Size Determination

Sample Size Considerations for the Dose-Escalation Study

The ability of the study to identify safety events can be expressed in terms of the probability of observing 1 or more event of interest (e.g., AEs) within each arm. With the sample size n=9 (receiving MAb) in each arm, there is over a 90% chance to observe at least 1 AE if the true rate is at least 0.226 and over a 90% chance to observe no AE if the true rate is no more than 0.011. Probabilities of observing 0 or more than 1 AE within a group are presented in Table 5 for a range of possible true event rates.

Table 5. Probability of events for different safety scenarios within an arm (n=9).

True event rate	n=9	
	Pr(0)	Pr(>1)
0.005	0.956	0.001
0.01	0.914	0.003
0.02	0.834	0.013
0.035	0.726	0.037
0.05	0.63	0.071
0.1	0.387	0.225
0.15	0.232	0.401
0.2	0.134	0.564
0.3	0.04	0.804

Abbreviations: Pr, probability.

Sample Size Considerations for the Efficacy Study

Table 6 shows initial sample size estimations powered to detect a 70% vaccine efficacy (1 – [proportion infected in L9LS/proportion infection in control]) at 12 months comparing L9LS at 10-20 mg/kg to placebo. Sample size calculations were done assuming the endpoint of any Pf infection will be calculated using a proportional analysis, 80% or 90% power, an alpha of 0.05, and that either 30%, 35%, or 40% of children in the placebo group are infected at 6 months and that 40% or 45% are infected at 12 months. (Time-to-event analyses typically require smaller sample sizes.) The primary endpoint will be at 12 months and will combine both MAb age groups who received two doses of L9LS and compare those subjects to the overall placebo group, i.e., we will assess MAb efficacy among children 5–59 months. However, the trial sample size is based on the secondary

endpoint of 12-month MAb efficacy with two doses among the younger age group (5–17 months) versus the 5–17-month-old subjects in the placebo group, as these will require larger sample sizes than the primary endpoint. Factoring in 25% loss to follow-up over 12 months, and a doubling of the sample size in each intervention arm to allow for comparison of L9LS with a second dose (at 6 months) to placebo and comparison of L9LS without a second dose to placebo would require 108 children in each L9LS group and 54 children in the placebo arm, assuming 35% infected in the placebo group at 6 months.

Table 6. Sample size calculations based on secondary 12-month endpoint, to achieve 80% power to detect efficacy of 70% at 6 months and 60% at 12 months, assuming 35% of the control group are infected by 6 months and 45% by 12 months.

Arm	Assumed proportion infected in control group by 6 mos	mAb Efficacy at 6 months	Power	Number needed at 6 months	Number needed to enroll assuming 20% LTFU and booster split	Assumed % infected in control group by 12 mos	mAb Efficacy at 12 months	Number needed at 12 months	Number needed to enroll	Numbe r with 20% LTFU*
mAb 5-17 mos + Dose 2	0.30	70%	80%	52	130	0.40	60%	52	104	130
mAb 5-17 mos No dose 2	0.30					0.40	60%	52		
mAb 18 mos-5 years + Dose 2	0.30	70%	80%	52	130	0.40	60%	52	104	130
mAb 18 mos-5 years No dose 2	0.30					0.40	60%	52		
Placebo 5 -17 mos	0.30	70%	80%	52	65	0.40	60%	52	52	65
Placebo 18 mos-5 years	0.30	70%	80%	52	65	0.40	60%	52	52	65
mAb 5-17 mos + Dose 2	0.30	70%	90%	70	175	0.40	60%	69	138	173
mAb 5-17 mos No dose 2	0.30					0.40	60%	69		
mAb 18 mos-5 years + Dose 2	0.30	70%	90%	70	175	0.40	60%	69	138	173
mAb 18 mos-5 years No dose 2	0.30					0.40	60%	69		
Placebo 5-17 mos	0.30	70%	90%	70	175	0.40	60%	69	69	87
Placebo 18 mos-5 years	0.30	70%	90%	70	175	0.40	60%	69	69	87

mAb 5-17 mos + Dose 2	0.35	70%	80%	43	108	0.45	60%	43	86	108
mAb 5-17 mos No dose 2	0.35					0.45	60%	43		
mAb 18 mos-5 years + Dose 2	0.35	70%	80%	43	108	0.45	60%	43	86	108
mAb 18 mos-5 years No dose 2	0.35					0.45	60%	43		
Placebo 5-17 mos	0.35	70%	80%	43	54	0.45	60%	43	43	54
Placebo 18 mos-5 years	0.35	70%	80%	43	54	0.45	60%	43	43	54
mAb 5-17 mos + Dose 2	0.35	70%	90%	57	143	0.45	60%	57	114	143
mAb 5-17 mos No dose 2	0.35					0.45	60%	57		
mAb 18 mos-5 years + Dose 2	0.35	70%	90%	57	143	0.45	60%	57	114	143
mAb 18 mos-5 years No dose 2	0.35					0.45	60%	57		
Placebo 5-17 mos	0.35	70%	90%	57	72	0.45	60%	57	57	72
Placebo 18 mos-5 years	0.35	70%	90%	57	72	0.45	60%	57	57	72
mAb 5-17 mos + Dose 2	0.40	70%	80%	35	88	0.45	60%	43	86	108
mAb 5-17 mos No dose 2	0.40					0.45	60%	43		
mAb 18 mos-5 years + Dose 2	0.40	70%	80%	35	88	0.45	60%	43	86	108
mAb 18 mos-5 years No dose 2	0.40					0.45	60%	43		
Placebo 5-17 mos	0.40	70%	80%	35	44	0.45	60%	43	43	54
Placebo 18 mos-5 years	0.40	70%	80%	35	44	0.45	60%	43	43	54
mAb 5-17 mos + Dose 2	0.40	70%	90%	47	118	0.45	60%	43	86	115
mAb 5-17 mos No dose 2	0.40					0.45	60%	43		
mAb 18 mos-5 years + Dose 2	0.40	70%	90%	47	118	0.45	60%	43	86	115

mAb 18 mos-5 years No dose 2	0.40					0.45	60%	43		
Placebo 5-17 mos	0.40	70%	90%	47	59	0.45	60%	43	43	58
Placebo 18 mos-5 years	0.40	70%	90%	47	59	0.45	60%	43	43	58

Note: If infections in the control group are higher at 6 or 12 months than assumed but VE remains the same, then the statistical power increases.

*LTFU = loss to follow up

Sample Size Considerations for the Year 2 Extension Study

In part 1b of the study for evaluating the safety of L9LS at 30mg/kg and 40mg/kg, 12 new participants will be enrolled into each age-dose group where 9 participants will receive L9LS of 30mg/kg (or 40mg/kg) and 3 will receive placebo. The probabilities of observing 0 or more than 1 AE among the L9LS recipients within a group are presented in Table 5 over a range of possible true event rates.

In year 2 extension study, safety endpoints will be assessed within each arm, and each L9LS arm will additionally be compared with the placebo arm. Table 7 presents the minimum detectable difference in adverse event rate with 80% and 90% power over possible event rates under placebo, with each arm of 108 participants.

Table 7. The minimum detectable difference in event rate between L9LS arm and placebo arm

Event rate (under placebo)	With 80% power		With 90% power	
	Detectable difference	Event rate (under L9LS)	Detectable difference	Event rate (under L9LS)
0.05	0.119	0.169	0.143	0.193
0.1	0.143	0.243	0.17	0.27
0.2	0.172	0.372	0.201	0.401
0.3	0.186	0.486	0.215	0.515

In year 2 extension study to assess the protective efficacy, each L9LS arm will be compared with the placebo arm. Assuming a lost to follow up rate of 20%, Table 8 shows the comparison power at various rates of the infection with 108 participants per arm. In addition, the two L9LS arms will also be compared with each other, and the comparison power is shown in Table 9.

Table 8.The comparison power with 108 participants per arm and a lost to follow up rate of 20%

Sample size per arm	Lost to follow up rate (%)	Infection rate (under placebo)	Infection rate (under L9LS)	mAb efficacy	Power (%)
108	20%	0.45	0.225	0.5	89
			0.18	0.6	97
			0.135	0.7	100
		0.4	0.2	0.5	82
			0.16	0.6	95
			0.12	0.7	99
		0.35	0.175	0.5	75
			0.14	0.6	90
			0.105	0.7	98

Table 9. The comparison power between two L9LS arms with 108 participants per arm and a lost to follow up rate of 20%

Sample size per arm	Lost to follow up rate	Infection rate in the high infection arm	Infection rate in the low infection arm	Power (%)
108	20%	0.15	0.05	59
		0.15	0.1	17
		0.2	0.05	85
		0.2	0.1	45
		0.2	0.15	14
		0.25	0.05	96
		0.25	0.1	74
		0.25	0.15	37
		0.25	0.2	12
		0.3	0.05	99
		0.3	0.1	91
		0.3	0.15	66
		0.3	0.2	33

Populations for Analyses

The following datasets will be considered in study analyses:

- Intention-to-treat (ITT) analysis dataset will include all subjects that are randomized and will be analyzed according to the initial randomization assignment.
- Modified intention-to-treat (MITT) analysis dataset will include all randomized subjects that receive the study intervention and will be analyzed according to the initial randomization assignment.
- Per-protocol (PP) analysis dataset will include all randomized subjects that receive the study intervention consistent with the initial randomization assignment and complete the scheduled visits, and will be analyzed according to the initial randomization assignment. In cases where subjects receive an intervention other than the one randomly assigned, an as-treated analysis will be additionally performed according to the actual product received.

Evaluable for toxicity

All participants will be evaluable for toxicity from the time of their first treatment with L9LS or placebo. The toxicity analysis will be MITT.

Evaluable for objective response

Not applicable.

Evaluable Non-Target Disease Response

Not applicable.

Statistical Analyses

A detailed study statistical analytical plan for the final analysis, which will supersede the study protocol, will be drawn up during the study before the unblinding of data at database lock.

General Approach

In general, descriptive statistics will be tabulated by treatment arm for endpoints of interest. This will include point estimates (mean, geometric mean, median, or proportions) and their respective 95% confidence intervals. Formal comparisons will use standard methods, contingency tables for categorical variables, t-tests for comparing means if data follow a normal distribution or geometric means if data after log transformation follow a normal distribution, or nonparametric analogs for comparing medians. Unless specified in the subsequent sections, comparisons will be two-sided with type I error rate of 0.05.

Missing data will be considered as “missing completely at random” provided the amount of missing data is modest (e.g., <10%). We will examine the “missing completely at random” assumption if the amount of missing data is more than 10%. If the assumption does not hold, missing data will be handled under the “missing at random” assumption (that is, missingness depends only on observed variables) via methods such as multiple imputation and inverse propensity weighting. To handle the possibility of “missing not at random,” a sensitivity analysis will be performed by imputing missing binary observations with the observed proportion in the opposite arm. A

secondary sensitivity analysis will be considered by imputing missing binary observations as failures.

Randomization: In the efficacy study, participants will be stratified by age (5-17 months versus 18-59 months) and then each age group will be randomized **2:1** to the L9LS arm and the placebo arm. The total number of participants will be 108 in each arm. In the dose-escalation study, participants in each age group, starting with 5-10 years, followed by 5-59 months, will be recruited and randomized 3:1 to receive L9LS at the dose of 5 mg/kg or placebo; if no safety concerns arise, randomization into the L9LS at the dose of 10 mg/kg or placebo, and finally 20 mg/kg or placebo, ensues in the same manner. To limit the number of dropouts before L9LS administration, randomization and L9LS administration will occur as close in time as possible.

Analysis of the Primary Endpoints

Analysis for the primary endpoint for safety and tolerability is described in section 3.2.

Analysis for the primary efficacy endpoint, protective efficacy with Pf infection determined by blood smear, is described in section 3.3.

Analysis of the Secondary Endpoint(s)

Analysis of the secondary efficacy endpoints, for example, incidence of Pf infection determined by RT-PCR and incidence of Pf infection against clinical malaria, is described in section 3.3.

Analyses of the secondary endpoints for the PK of L9LS and the association of L9LS concentration with Pf infection risk are described in section 0.

Efficacy Analyses

The primary efficacy endpoint (efficacy study primary endpoint) is the incidence of malaria infection defined as blood smear–positive Pf infection, starting on Day 7 after MAb/placebo administration, through 52 weeks after administration. The secondary efficacy endpoints are the incidence of malaria infection as determined by RT-PCR and the incidence of clinical malaria, up to 12, 24 weeks and up to 52 weeks, as well as the incidence of malaria infection by blood smear up to 12 and up to 24 weeks. The efficacy analyses will be MITT over participants in the efficacy study.

The primary efficacy analysis will be based on time to the first infection. The survival patterns will be described by Kaplan-Meier curves for each arm and compared by the logrank test across different arms. The protective efficacy of the study product will be estimated by the hazard ratio from the Cox proportional hazards model. These analyses will be carried out by R packages or commands in similar statistical software that account for interval censoring: package "interval" for deriving a nonparametric maximum likelihood estimation based on the Kaplan-Meier survival curve and logrank test, and package "icenReg" for Cox proportional hazards regression. To address the heterogeneity of the study population, a Cox regression with regressors other than the study arm

will be additionally performed to account for potential differences among participants. The regressors will include time of enrollment and possibly those covariates that are significantly different between the study product arms and the placebo arm in spite of randomization. The primary analysis will compare all children (both age groups) receiving MAb to placebo. Secondary endpoint analyses will compare the younger children receiving MAb to the younger children in the placebo group and the older children receiving MAb to the older children in the placebo group. Since these are secondary endpoints, no adjustments for multiple comparisons will be made.

The secondary efficacy analysis will be based on the proportion of infection. The proportion of infection will be estimated for each arm and compared across arms based on Kaplan-Meier estimates: the proportion of infection by one minus the Kaplan-Meier estimate in the arm, and the vaccine efficacy by one minus the relative risk of infection with melding method for constructing the melded 95% confidence interval of the vaccine efficacy. [3634]

Finally, all clinical malaria episodes will be included in exploratory analyses. All person-time during the trial, including the time after the initial positive evaluation for Pf infection or clinical malaria, will be included in these analyses. This approach will use a counting process which is an extension of the Cox proportional hazards model to incorporate multiple events. [3735] The 14 days following treatment with artemether-lumefantrine will be removed from these analysis. In these models the number of prior events will be included as a covariate in order to ensure that the conditional independence assumption of the counting process is still met since a previous infection may confer some future protection. [3836]

Although the trial will not be weight-stratified, given that we have age stratification, we anticipate that participants will cover the dose range from 10 mg/kg to 20 mg/kg in the efficacy analyses. In addition to the above efficacy analyses that compare each dose arm with the placebo arm, an exploratory analysis will be additionally conducted to assess how dosage, in terms of mg of L9LS per kg of subject body weight, affects the protective efficacy.

Making these data available in a timely manner will add to the evidence base needed to plan a potential phase 3 trial of L9LS in settings with seasonal malaria transmission.

Safety Analyses

Safety analysis will be primarily MITT where individuals who receive assignment but do not receive any product are excluded. Because of blinding and the brief length of time between assignment and administration, such cases will be very few. In the rare case of subjects receiving a regimen different from assignment, an as-treated analysis will be additionally performed where subjects will be analyzed according to the product they actually receive.

Safety data will be presented by line listings and tables at the individual level to provide details on safety events such as severity, duration, and relationship to study product. The number and percentage of subjects with 1 or more AE(s) will be summarized by dose arm along with the exact 95% confidence intervals of the AE rate. For subjects experiencing more than 1 AE, the subjects will be counted once under the event of highest severity.

In the efficacy study, comparisons between the dose arms and the control arm will be additionally performed in terms of the proportions of solicited AEs, related AEs, and SAEs.

In the rare case of subjects receiving a regimen different from assignment, a per-protocol analysis will be performed as a secondary analysis, which will include subjects according to the product they actually receive in the study.

Pharmacokinetics Analysis

PK analysis will be carried out for subjects in the dose-escalation study and in the efficacy study with blood samples collected at defined timepoints as listed in section 0. The PK analysis will be PP. The following PK analysis will be performed as needed.

Individual Subject Pharmacokinetic Analysis: A non-compartmental (NC) PK analysis will be performed on the L9LS concentration data generated from each subject. Individual subject and dosing arm concentration-versus-time profiles will be constructed in linear and semi-log scales. In the NC analysis, the maximum concentration (Cmax) and time of maximal concentration (Tmax) will be taken directly from the observed data. The area under the concentrations vs. time curve (AUC) will be calculated using the trapezoidal method and determined out to the final concentration collected. If a subject's L9LS concentration falls below the quantitative limit (QL) of the assay, the sample with concentration below the QL will be assigned a L9LS concentration value of "0" for AUC calculations. In addition to the total AUC, partial AUCs will also be determined over certain time intervals. The time-weighted average concentrations (Cave) during these intervals will be calculated as the AUC divided by the AUC collection interval (e.g., $Cave_{0\text{-}16WK}$ = ($AUC_{0\text{-}16WK}$) / 16 weeks). The terminal slope, λ_z, will be determined by regression of the terminal, log-linear portion of the concentration-versus-time profile. If the final PK sample has measurable L9LS concentrations

greater than the assay QL, the AUC post-final PK collection ($AUC_{last-infinity}$) will be estimated as C_{last} / λ_z and $AUC_{0-infinity}$ will be calculated as the sum of AUC_{0-last} + $AUC_{last-infinity}$.

Population Pharmacokinetic Analyses: Based on preclinical PK results for L9LS and known PK behavior studies of MAbs, the two-compartment model will be used for population PK analysis. The population analysis will estimate compartmental PK parameters such as the clearance (CL), central and peripheral volumes of distribution (Vd1 and Vd2), and intercompartmental clearance (Q). Total volume of distribution at steady-state (Vdss), will be calculated as the sum of Vd1 + Vd2. Alpha and beta half-lives will be calculated from CL, Q, Vd1, and Vd2 using standard equations. [3937]

To assess the association of L9LS concentration with protection, we will perform a Cox proportional hazards regression for the time to the first infection with L9LS concentration as a time-varying covariate. A logistic regression analysis will be additionally performed to model the infection rate as a function of L9LS concentration using generalized estimating equation to account for repeated measures. This analysis will be performed over all participants that receive L9LS including those aged 5-10 years in the dose-escalation study and those in the efficacy study.

Baseline Descriptive Statistics

Treatment arms will be compared for baseline subject characteristics using descriptive statistics. For continuous variables, the mean or median will be calculated for each treatment arm. For categorial variables, the proportion under each category will be calculated for each arm.

Planned Interim Analyses

Interim safety analysis will be performed when safety data are available from the age de-escalation and dose escalation study subjects, starting at least 7 days after the last participant in part 1 receives their antibody (or placebo) dose. The purpose of the interim analysis on safety data is to clear safety concerns for proceeding to the efficacy study.

An interim efficacy analysis will be conducted when clean data are available for all participants through the 6-month endpoint (at 24 weeks). This will assess Pf infection up to 24 weeks by blood smear and clinical malaria using time-to-event analysis and proportional analyses. Having these data available in a timely manner will add to the evidence base needed to plan a potential phase 3 trial of L9LS in settings with seasonal malaria transmission where the time for protection is 6 months. The interim analysis will not impact the conduct of this trial or induce significance level adjustment in the efficacy analyses of this trial.

Sub-Group Analyses

Not applicable.

Tabulation of individual Participant Data

Safety data will be presented by line listing and tables at the individual level, as described in section 3.2.

Exploratory Analyses

To explore the impact of L9LS on the genotype of infection -inducing parasites at the CSP locus, a genotypic sieve analysis will be performed to analyze CSP sequences of breakthrough parasites in the blood samples of infected subjects. The sieve analysis will differentiate protective efficacy against different genotypes of infection-inducing parasites with genotype defined by, for example, number of mismatches to the L9LS footprint. To explore whether receipt of MAb affects development of measles-specific antibodies among those who receive the measles vaccine after their first MAb dose, mean and median antibody titers will be compared among subjects <9 months of age at enrollment, prior to and after receipt of MAb. The exploratory analysis comparing one versus two doses of L9LS against Pf infection and clinical malaria will be done as outlined in section 3.3 above.

REGULATORY AND OPERATIONAL CONSIDERATIONS

Informed Consent Process

Consent/Assent Procedures and Documentation

As this study will include only children 10 years of age and younger, informed consent/permission will be obtained from parents or guardians prior to any study procedure. All consent forms will be translated into Dholuo and Kiswahili and back translated into English to ensure accuracy.

The consent form will detail the design of the study, the intervention (vaccination with L9LS or placebo), collection of blood samples, and study requirements for follow-up. A designated and trained study staff will describe the protocol to potential participants' parent(s)/guardian(s) face to face, the informed consent form will be read together with the participants' parent(s)/guardian(s), and the participants' parent(s)/guardian(s) shall be given ample opportunity to inquire about details of the study and ask any questions, including from clinical staff, before dating and signing (or thumbprinting, in the case of illiterate persons) the informed consent form. Consenting of an illiterate parent/guardian will be performed in the presence of an impartial witness who will sign the consent/permission form on behalf of the parent/guardian. The illiterate parent/guardian will be asked to sign or place their thumbprint on the consent form to document their consent/permission.

Details about the trial and its benefits and potential risk will be explained to the parent/guardian in the language in which they are most fluent. All information regarding the participants will remain confidential to the extent allowed by law. Unique numerical identifiers will be used for data entry.

The storage of blood samples will then be explained to the parents or guardians. If they agree to have their child's blood samples stored at KEMRI, each individual will be asked to sign consent to store samples. Permission will also be sought for shipping of some samples to the NIH and other

collaborating laboratories in the US for immunologic testing and parasite genotyping. All parents/guardians will be provided with a signed consent form after they have agreed to participate in the study. Should a parent/guardian prefer not to keep a copy of the consent form, this will be documented. Should the consenter be non-medical personnel, a clinical/medical officer or nurse will be available to answer further questions.

Each parent's/guardian's signed informed consent form will be kept on file by the investigator for possible inspection by regulatory authorities. Any amendments to the written information will be provided to participants' parents/guardians.

Justification for Alteration of Informed Consent

Due to the high illiteracy rate in the area, we are seeking an alteration of informed consent to accept a witnessed thumbprint in lieu of a signature for parent(s)/guardian(s) who cannot sign their names.

Study Discontinuation and Closure

The study may be temporarily suspended or permanently terminated as described in the halting rules (section 0). In addition to the reporting described in that section, the principal investigator(s) will promptly contact the study subjects, provide the reason(s) for the termination or suspension, and, if applicable, inform them of changes to study visit schedule.

The principal investigators will consult with the IRBs prior to resuming the study following a halt.

Confidentiality and Privacy

All records will be kept confidential to the extent provided by federal, state, and Kenyan law. The study monitors and other authorized representatives of the sponsor may inspect all documents and records required to be maintained by the investigator, including but not limited to, medical records. Records will be kept in the study clinics and/or KEMRI-CGHR under key and lock and data will be coded. Any personally identifiable information maintained for this study will be kept on restricted-access computers and networks. Personally identifiable information will only be shared with individuals authorized to receive it under this protocol. Individuals not authorized to receive personally identifiable information will be provided with coded information only, as needed. Clinical information will not be released without written permission of the subject, except as necessary for monitoring by the IRBs, FDA, NIAID, Office for Human Research Protections (OHRP), the VRC, or the sponsor's designee.

To further protect the privacy of study subjects, a Certificate of Confidentiality has been issued by the NIH. This certificate protects identifiable research information from forced disclosure. It allows the investigator and others who have access to research records to refuse to disclose identifying information on research participation in any civil, criminal, administrative, legislative, or other proceeding, whether at the federal, state, or local level. By protecting researchers and institutions from being compelled to disclose information that would identify research subjects, Certificates of

Confidentiality help achieve the research objectives and promote participation in studies by helping assure confidentiality and privacy to subjects.

Samples and data will be collected and stored under this protocol. All of the stored study research samples are labeled by a code that only the investigators can link to the subject. Samples are stored in secure research laboratories in locked freezers with limited access at the KEMRI, and the NIH. Data will be kept in password-protected computers. Only investigators or their designees will have access to the samples and data.

Samples and data acquired under this protocol will be tracked using BSI Systems software.

Any loss or unanticipated destruction of samples (for example, due to freezer malfunction) or data (for example, misplacing a printout of data with identifiers) that meets the definition of a reportable event will be reported to the IRBs.

Additionally, subjects may decide at any point before the end of the trial not to have their samples stored. In this case, the principal investigator will destroy all known remaining samples and report what was done to both the subject and to the IRBs. This decision will not affect the individual's participation in this protocol or any other protocols at NIH.

Future use of Stored Specimens and Data

Subjects are consented at enrollment for permission for storage and future use of specimens and data. Samples, specimens, and data collected under this protocol may be used to study malaria and the immune system. Genetic testing will only be performed on the malaria parasite, but no human genetic testing will be conducted.

Storage and Tracking: Access to and tracking of stored samples and data will be secured and limited as described above (section 0).

Disposition: In the future, other investigators (both at NIH and outside) may wish to use these samples and/or data for research purposes. If the planned research falls within the category of "human subjects research" on the part of the NIH researchers, IRB review and approval will be obtained. This includes the NIH researchers sending out coded and linked samples or data and getting results that they can link back to their subjects.

Safety Oversight

Safety oversight is described in section 0.

Clinical Monitoring

According to the ICH E6(R2) GCP guidelines, section 5.18, and FDA 21 CFR 312.50, clinical protocols are required to be adequately monitored by the study sponsor. This study monitoring will be conducted according to the "NIAID Intramural Clinical Monitoring Guidelines." Monitors under contract to the NIAID/OCRPRO will visit the clinical research site to monitor aspects of the study in accordance with the appropriate regulations and the approved protocol. The objectives of a monitoring visit will be: 1) to verify the existence of signed informed consent documents and documentation of the consent process for each monitored subject; 2) to verify the prompt and accurate recording of all monitored data points in DFdiscover and prompt reporting of all SAEs; 3) to compare abstracted information entered into DFdiscover with individual subjects' records and source documents (subjects' charts, laboratory analyses and test results, physicians' progress notes, nurses' notes, and any other relevant original subject information); and 4) to help ensure investigators are in compliance with the protocol. The monitors also will inspect the clinical site regulatory files to ensure that regulatory requirements (OHRP, FDA) and applicable guidelines (ICH GCP) are being followed. During the monitoring visits, the investigator (and/or designee) and other study personnel will be available to discuss the study progress and monitoring visit.

The investigator (and/or designee) will make study documents (e.g., consent forms, DFdiscover abstracts) and pertinent hospital or clinical records readily available for inspection by the local IRB, FDA, the site monitors, and the NIAID staff for confirmation of the study data.

A specific protocol monitoring plan will be discussed with the principal investigator and study staff prior to enrollment. The plan will outline the frequency of monitoring visits based on such factors as study enrollment, data collection status, and regulatory obligations.

Quality Assurance and Quality Control

During the study, the principal investigator and study team will be responsible for ensuring study activities are conducted in compliance with the protocol, ICH GCP, and applicable regulatory requirements. Each clinical site will perform internal quality management of study conduct, data and biological specimen collection, and documentation according to study SOPs.

Data Handling and Record Keeping

Data Collection and Management Responsibilities

Study data will be maintained in CRFs and collected directly from subjects during study visits and telephone calls or will be abstracted from subjects' medical records. Source documents include all recordings of observations or notations of clinical activities, including CRFs, and all reports and records necessary to confirm the data abstracted for this study. Data entry into CRFs will be performed by authorized individuals. The investigator is responsible for assuring that the data collected are complete, accurate, and recorded in a timely manner. Study data, including cumulative subject accrual numbers, should be generated via the chosen data capture method and submitted to the IRBs as needed.

Study Records Retention

Study documents will be retained in accordance with regulatory and institutional requirements, ICH GCP guidelines, and the NIH Intramural Records Retention Schedule. No records will be destroyed without the written consent of the principal investigators/protocol chair and sponsor, as applicable.

Should the investigator wish to assign the study records to another party and/or move them to another location, the investigator will provide written notification of such intent to OCRPRO/NIAID and SERU with the name of the person who will accept responsibility for the transferred records and/or their new location. NIAID will be notified in writing and written OCRPRO/NIAID permission shall be obtained by the site prior to destruction or relocation of research records.

Protocol Deviations and Non-Compliance

It is the responsibility of the investigators to use continuous vigilance to identify and report deviations and non-compliance to the IRBs and the NIH (as applicable) according to NIH Human Research Protection Program Policy 801 (as described in section 0). All deviations must be addressed in study source documents and reported to the NIAID Program Official and sponsor. The investigators are responsible for knowing and adhering to the reviewing IRB requirements.

NIH Definition of Protocol Deviation

The definition of a protocol deviation is provided in section 0.

Publication and Data Sharing Policy

Human Data Sharing Plan

This study will be conducted in accordance with the following publication and data sharing policies and regulations:

- NIH Public Access Policy, which ensures that the public has access to the published results of NIH funded research. It requires scientists to submit final peer-reviewed journal manuscripts that arise from NIH funds to the digital archive PubMed Central upon acceptance for publication.
- This study will comply with the NIH Data Sharing Policy and Policy on the Dissemination of NIH-Funded Clinical Trial Information and the Clinical Trials Registration and Results Information Submission rule. As such, this trial will be registered at ClinicalTrials.gov, and results information from this trial will be submitted to ClinicalTrials.gov. In addition, every attempt will be made to publish results in peer-reviewed journals. Data from this study may be requested from other researchers indefinitely after the completion of the primary endpoint by contacting one of the principal investigators.

Human data generated in this study for future research will be shared as follows:

- De-identified or identified data with approved outside collaborators under appropriate agreements.
- De-identified results or data in publication and/or public presentations.

Data will be shared at the time of publication or shortly thereafter.

Genomic Data Sharing Compliance

Not applicable.

Collaborative Agreements

Not applicable.

Agreement Type

Not applicable.

Conflict of Interest Policy

The independence of this study from any actual or perceived influence, such as by the pharmaceutical industry, is critical. Therefore, any actual conflict of interest of persons who have a role in the design, conduct, analysis, publication, or any aspect of this trial will be disclosed and managed. Furthermore, persons who have a perceived conflict of interest will be required to have such conflicts managed in a way that is appropriate to their participation in the design and conduct of this trial. The study leadership will follow policies and procedures for all study group members to disclose all conflicts of interest and will establish a mechanism for the management of all reported dualities of interest.

BUDGET AND BUDGET JUSTIFICATION

Budget

**Country : Kenya**			
**Study: Malaria mAb Vaccine**			
**ANNUAL BUDGETS**			
**KEMRI Research**<br>**Cooperative Agreement**	**YEAR 1**<br>***Budget***<br>**Jan-Dec 2024**	**YEAR 2**<br>***Budget***<br>**Jan-Dec 2025**	**TOTAL (USD)**

Personnel	86,149.15	90,233.26	176,382.40
Equipment	65,000.00	35,000.00	100,000.00
Supplies	131,510.20	140,762.90	272,273.10
Travel	26,387.40	15,368.30	41,755.70
Other	311,801.81	297,180.05	608,981.87
Contractual	1,143,250.59	1,170,397.39	2,313,647.98
TOTAL DIRECT COSTS	**1,764,099.15**	**1,750,441.90**	**3,514,541.05**
INDIRECT COSTS	*44,467.88*	*43,603.56*	*88,071.45*
TOTAL AWARD	**1,808,567.04**	**1,794,045.46**	**3,602,612.49**

11.2 Budget Justification

Personnel: Funds will be used to pay salary and fringe benefits for study personnel.

Laboratory supplies: These funds will be used to purchase supplies (rapid tests, reagents,

transport medium and other disposable lab supplies) to be used in the laboratory for the conduct of the study.

Clinical supplies: Funds will be used to purchase clinical supplies; drugs, PPE, syringes, gloves

and other clinical supplies for use in hospital where study participants will be enrolled and managed.

Subcontracts: These funds will be utilized to procure the Clinical Trials Insurance for the study

Equipment: Funds will be utlised to purchase study equipment ie laptops and preventative maintenance of lab and pharmacy equipment.

Travel: These are funds for both local and international travel to conferences and meetings related to study activities.

Transportation: The funds will be utilized for fuel and maintenance of the study motorvehicles and motorcycles.

Other project costs: These include funds for payment of regulatory institutions ie KPPB, SERU and other general study costs ie internet, airtime, customas clearance of study supplies and samples among others.

Other supplies: These include transport reimbursement and refreshments for study participants.

KEMRI OH Costs: These are indirect costs paid to KEMRI to cater for administrative support and utilities.

REFERENCES

1. World Health Organization. Microscopy for the detection, identification and quantification of malaria parasites on stained thick and thin blood films in research settings. . Geneva, 2015.
2. Swysen C, Vekemans J, Bruls M, et al. Development of standardized laboratory methods and quality processes for a phase III study of the RTS, S/AS01 candidate malaria vaccine. *Malar J* 2011; **10**: 223.
3. World Health Organization. World Malaria Report 2020: World Health Organization, 2020.
4. RTS,S Clinical Trials Partnership. Efficacy and safety of RTS,S/AS01 malaria vaccine with or without a booster dose in infants and children in Africa: final results of a phase 3, individually randomised, controlled trial. *Lancet* 2015; **386**(9988): 31-45.
5. Mahmoudi S, Keshavarz H. Efficacy of phase 3 trial of RTS, S/AS01 malaria vaccine: The need for an alternative development plan. *Hum Vaccin Immunother* 2017; **13**(9): 2098-101.
6. World Health Organization. WHO recommends groundbreaking malaria vaccine for children at risk: Historic RTS,S/AS01 recommendation can reinvigorate the fight against malaria.
7. RTS,S Clinical Trials Partnership. Efficacy and safety of the RTS,S/AS01 malaria vaccine during 18 months after vaccination: a phase 3 randomized, controlled trial in children and young infants at 11 African sites. *PLoS Med* 2014; **11**(7): e1001685.
8. Opoka RO, Hamre KES, Brand N, Bangirana P, Idro R, John CC. High Postdischarge Morbidity in Ugandan Children With Severe Malarial Anemia or Cerebral Malaria. *J Pediatric Infect Dis Soc* 2017; **6**(3): e41-e8.
9. Phiri KS, Calis JC, Faragher B, et al. Long term outcome of severe anaemia in Malawian children. *PLoS One* 2008; **3**(8): e2903.
10. Kwambai TK, Dhabangi A, Idro R, et al. Malaria Chemoprevention in the Postdischarge Management of Severe Anemia. *N Engl J Med* 2020; **383**(23): 2242-54.
11. Kisalu NK, Idris AH, Weidle C, et al. A human monoclonal antibody prevents malaria infection by targeting a new site of vulnerability on the parasite. *Nat Med* 2018; **24**(4): 408-16.
12. Livingstone MC, Bitzer AA, Giri A, et al. In vitro and in vivo inhibition of malaria parasite infection by monoclonal antibodies against Plasmodium falciparum circumsporozoite protein (CSP). *Sci Rep* 2021; **11**(1): 5318.
13. Gaudinski MR, Berkowitz NM, Idris AH, et al. A Monoclonal Antibody for Malaria Prevention. *N Engl J Med* 2021; **385**(9): 803-14.
14. Wang LT, Pereira LS, Flores-Garcia Y, et al. A Potent Anti-Malarial Human Monoclonal Antibody Targets Circumsporozoite Protein Minor Repeats and Neutralizes Sporozoites in the Liver. *Immunity* 2020; **53**(4): 733-44 e8.
15. Soliris (eculizumab) injection [package insert]. Boston, MA: Alexion Pharmaceuticals, Inc.; 2020.
16. ULTOMIRIS (ravulizumab-cwvz) injection [package insert]. Boston, MA: Alexion Pharmaceuticals, Inc.; 2021.
17. Wu RL, Idris AH, Berkowitz NM, et al. Low-Dose Subcutaneous or Intravenous Monoclonal Antibody to Prevent Malaria. *N Engl J Med* 2022; **387**(5): 397-407.
18. Kayentao K, Ongoiba A, Preston AC, et al. Safety and Efficacy of a Monoclonal Antibody against Malaria in Mali. *N Engl J Med* 2022; **387**(20): 1833-42.
19. Ledgerwood JE, Coates EE, Yamshchikov G, et al. Safety, pharmacokinetics and neutralization of the broadly neutralizing HIV-1 human monoclonal antibody VRC01 in healthy adults. *Clin Exp Immunol* 2015; **182**(3): 289-301.
20. Vaisman-Mentesh A, Rosenstein S, Yavzori M, et al. Molecular Landscape of Anti-Drug Antibodies Reveals the Mechanism of the Immune Response Following Treatment With TNFalpha Antagonists. *Front Immunol* 2019; **10**: 2921.

21. European Medicines Agency. Guideline on Immunogenicity assessment of therapeutic proteins 2017.
22. Strand V, Balsa A, Al-Saleh J, et al. Immunogenicity of Biologics in Chronic Inflammatory Diseases: A Systematic Review. *BioDrugs* 2017; **31**(4): 299-316.
23. van Schouwenburg PA, Rispens T, Wolbink GJ. Immunogenicity of anti-TNF biologic therapies for rheumatoid arthritis. *Nat Rev Rheumatol* 2013; **9**(3): 164-72.
24. Lynch RM, Boritz E, Coates EE, et al. Virologic effects of broadly neutralizing antibody VRC01 administration during chronic HIV-1 infection. *Sci Transl Med* 2015; **7**(319): 319ra206.
25. Mayer KH, Seaton KE, Huang Y, et al. Safety, pharmacokinetics, and immunological activities of multiple intravenous or subcutaneous doses of an anti-HIV monoclonal antibody, VRC01, administered to HIV-uninfected adults: Results of a phase 1 randomized trial. *PLoS Med* 2017; **14**(11): e1002435.
26. Walsh SR, Seaman MS. Broadly Neutralizing Antibodies for HIV-1 Prevention. *Front Immunol* 2021; **12**: 712122.
27. FDA. Immunogenicity Testing of Therapeutic Protein Products —Developing and Validating Assays for Anti-Drug Antibody Detection. 2019.
28. Hansel TT, Kropshofer H, Singer T, Mitchell JA, George AJ. The safety and side effects of monoclonal antibodies. *Nat Rev Drug Discov* 2010; **9**(4): 325-38.
29. Bugelski PJ, Achuthanandam R, Capocasale RJ, Treacy G, Bouman-Thio E. Monoclonal antibody-induced cytokine-release syndrome. *Expert Rev Clin Immunol* 2009; **5**(5): 499-521.
30. Vogel WH. Infusion reactions: diagnosis, assessment, and management. *Clin J Oncol Nurs* 2010; **14**(2): E10-21.
31. Drug allergy: an updated practice parameter. *Ann Allergy Asthma Immunol* 2010; **105**(4): 259-73.
32. Shibeshi W, Alemkere G, Mulu A, Engidawork E. Efficacy and safety of artemisinin-based combination therapies for the treatment of uncomplicated malaria in pediatrics: a systematic review and meta-analysis. *BMC Infect Dis* 2021; **21**(1): 326.
33. Eurartesim (piperaquine tetraphosphate/dihydroartemisinin) Guide for Healthcare Professionals. Sigma Tau; 2016.
34. Kenya Medical Research Institute. KEMRI SERU GUIDELINES FOR THE CONDUCT OF RESEARCH DURING THE COVID-19 PANDEMIC IN KENYA, Version 1: Kenya Medical Research Institute, 2020.
35. CIS43LS [investigator's brochure]. Bethesda, MD: NIAID Vaccine Research Center; 2019.
36. Fay MP, Proschan MA, Brittain E. Combining one-sample confidence procedures for inference in the two-sample case. *Biometrics* 2015; **71**(1): 146-56.
37. Andersen PK, Gill RD. Cox's Regression Model for Counting Processes: A Large Sample Study. *The Annals of Statistics* 1982; **10**(4): 1100-20.
38. Amorim LD, Cai J. Modelling recurrent events: a tutorial for analysis in epidemiology. *Int J Epidemiol* 2015; **44**(1): 324-33.
39. Gibaldi M, Perrier D. Pharmacokinetics. New York, NY: Marcel Dekker, Inc.; 1975.
40. Bain BJ. Ethnic and sex differences in the total and differential white cell count and platelet count. *J Clin Pathol* 1996; **49**(8): 664-6.
41. Haddy TB, Rana SR, Castro O. Benign ethnic neutropenia: what is a normal absolute neutrophil count? *J Lab Clin Med* 1999; **133**(1): 15-22.

APPENDIX A: KENYA NATIONAL IMMUNIZATION SCHEDULE

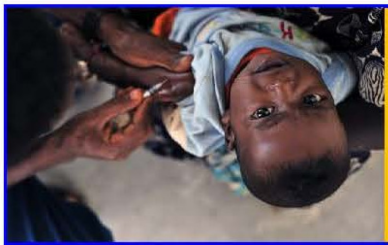

NATIONAL IMMUNIZATION SCHEDULE

Kenya

Recommended routine immunization

Vaccine	Description	Schedule	Comments
**Primary Infant and Adolescent Vaccination Schedule**			
**BCG**	Bacille Calmette-Guérin vaccine	Birth	
**OPV**	Oral polio vaccine	Birth; 6, 10, 14 weeks	
**Rotavirus**	Rotavirus vaccine	6, 10 weeks	
**Pneumo_conj**	Pneumococcal conjugate vaccine	6, 10, 14 weeks	
**DTwPHibHepB**	Diphtheria and Tetanus and Pertussis and Haemophilus influenzae and Hepatitis B vaccine	6, 10, 14 weeks	
**IPV**	Inactivated polio vaccine	14 weeks	
**YF**	Yellow fever vaccine	9 months	Not available in all parts of the country
**Measles**	Measles vaccine	9, 18 months	
**HPV**	Human Papillomavirus vaccine	10 years; +6 months	From january 2018
**Adult Vaccination Schedule**			
**HepB_Adult**	Hepatitis B adult dose vaccine	1st contact; +1, +1 months	High risk groups (e.g. health workers)
**TT**	Tetanus toxoid vaccine	1st contact pregnancy; +1, +6 months; +1, +1 year	
**Vaccines for Travellers**			
**Typhoid**	Typhoid fever vaccine	1st contact travellers	

Reference: Modified from World Health Organization (WHO)
Date accessed: 29 March 2018

APPENDIX B: KENYA ADVERSE EVENT GRADING SCALE

PART 1 AND 1B:

Evaluation	Mild (Grade 1)	Moderate (Grade 2)	Severe (Grade 3)	Potentially Life-threatening (Grade 4)
**Hematology and Biochemistry Values[1, 2]**				
**Hemoglobin[1] – gm/dL**	7.9 – 6.0	5.9 – 5.0	< 5.0	< 5.0 and clinical signs of heart failure
**WBC Increase[2] – 10^3/µL**	14.5 – 16.0	16.1 – 20.0	20.1 – 30.0	> 30.0
**WBC Decrease[1] – 10^3/µL**	2.5 – 4.0	1.5 – 2.4	1.0 – 1.4	< 1.0
**Neutrophil/Granulocyte Decrease[2,3] – 10^3/µL**	0.75 – 0.99	0.50 – 0.74	< 0.50	< 0.50 with fever
**Platelet Decrease[1] – 10^3/µL**	50 - 99	25 - 49	<25	< 25 with clinical signs of bleeding
**Creatinine [1]**	1.1–1.5 x ULN	1.6-3.0 x ULN	3.1-6.0 x ULN	>6.0 x ULN or requires dialysis
**Liver Function Test/ALT[1]**	1.1-2.5 x ULN	2.6-5.0 x ULN	5.1 – 10 x ULN	>10 x ULN
**Other Values**				
**Fever[4] – °C**	37.5 – 37.9	38.0 – 38.4	38.5 – 39.5	> 39.5

Abbreviations: ALT, alanine transaminase; WBC, white blood cell. ULN – Upper limit of normal

[1] The same grading was used in the most recent clinical trial of the RTS,S Malaria vaccine at the same site (Efficacy, safety and immunogenicity study of GSK Biologicals' candidate malaria vaccine (SB257049) evaluating schedules with or without fractional doses, early Dose 4 and yearly doses, in children 5-17 months of age)

[2] The same grading is used in the 'Safety and Efficacy of L9LS, a Human Monoclonal Antibody Against *Plasmodium falciparum*, in a Dose-Escalation Trial in Adults and Children and a Randomized, Double-Blind Trial of Children in Mali'

[3] Note: Neutropenias are graded and followed, but based on previous experience in African populations, should be interpreted with caution since lower values are more frequently observed in people of African descent.[40,41]

[4] Values presented are for non-oral temperature reading (i.e., axillary or no-touch), which is the preferred method at the study site.

PART 2 AND THE EXTENSION PHASE:

Evaluation	Mild (Grade 1)	Moderate (Grade 2)	Severe (Grade 3)	Potentially Life-threatening (Grade 4)
**Hematology and Biochemistry Values[1, 2]**				
**Hemoglobin[1] – gm/dL**	7.9 – 6.0	5.9 – 5.0	< 5.0	< 5.0 and clinical signs of heart failure
**WBC Increase[2] – $10^3/\mu L$**	15.5-20.0	20.1-30.0	> 30.0	Not applicable
**WBC Decrease[1] – $10^3/\mu L$**	2.5 – 4.0	1.5 – 2.4	1.0 – 1.4	< 1.0
**Neutrophil/Granulocyte Decrease[2,3] – $10^3/\mu L$**	0.75 – 0.99	0.50 – 0.74	< 0.50	< 0.50 with fever
**Platelet Decrease[1] – $10^3/\mu L$**	50 - 99	25 - 49	<25	< 25 with clinical signs of bleeding
**Creatinine [1]**	1.1–1.5 x ULN	1.6-3.0 x ULN	3.1-6.0 x ULN	>6.0 x ULN or requires dialysis
**Liver Function Test/ALT[1]**	1.1-2.5 x ULN	2.6-5.0 x ULN	5.1 – 10 x ULN	>10 x ULN
**Other Values**				
**Fever[4] – °C**	37.5 – 38.4	38.5 – 39.4	39.5 – 41.9	≥ 42

Abbreviations: ALT, alanine transaminase; WBC, white blood cell. ULN – Upper limit of normal

[1] The same grading was used in the most recent clinical trial of the RTS,S Malaria vaccine at the same site (Efficacy, safety and immunogenicity study of GSK Biologicals' candidate malaria vaccine (SB257049) evaluating schedules with or without fractional doses, early Dose 4 and yearly doses, in children 5-17 months of age)

[2] WBC increase numbers in part 2 have been adjusted to reflect the fact that this is a younger population than in Mali or in Part 1, and the normal range is higher. Additionally, elevated WBC in and of itself is not life threatening, thus we have noted this as not applicable.

[3] Note: Neutropenias are graded and followed, but based on previous experience in African populations, should be interpreted with caution since lower values are more frequently observed in people of African descent.[40,41]

[4] Values presented are for non-oral temperature reading (i.e., axillary or no-touch), which is the preferred method at the study site.

Statistical Analysis Plan Version 3.0

December 2, 2024

Safety and Efficacy of L9LS, a Human Monoclonal Antibody Against *Plasmodium falciparum*, in an Age De-Escalation, Dose-Escalation Trial and a Randomized, Placebo-Controlled, Double-Blind Trial of Children in Western Kenya

Protocol version 6.0. Date 2 April 2024

Study Identifiers:		
	CLINICALTRIALS.GOV REGISTRATION ID:	NCT05400655

Co-Principal Investigators:
Titus Kwambai, MD, PhD, Centers for Disease Control and Prevention Kenya Malaria Program
Laura Steinhardt, PhD, MPH, Malaria Branch, Centers for Disease Control and Prevention (CDC)
Peter D. Crompton, MD, MPH, Malaria Infection Biology and Immunity Section Laboratory of Immunogenetics (LIG), National Institute of Allergy and Infectious Diseases (NIAID), National Institutes of Health (NIH)
Co-Investigators:

- **NIH:** Robert A. Seder, MD
- **CDC**: Julie Gutman, MD, MS; Peter McElroy, PhD, MPH
- **LSTM:** Feiko ter Kuile, MD, PhD
- **KEMRI:** Eunice Ouma, MD; Martina Oneko, MD; Simon Kariuki, PhD; Ruth Njoroge, BPharm; Kephas Otieno, MSc

SAP version History:						
Version Date	SAP version #	Details of Changes	Signatures			
			PI 1	PI 2	PI 3	Statistician

November 2023	*1.0*		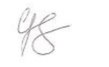	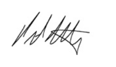	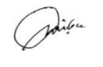	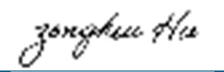
12 August 2024	*2.0*	*Aligned objectives to the protocol*	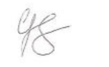	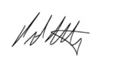	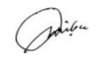	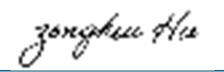
2 December 2024	*3.0*	*Added recurrent event analysis for first/only infection*	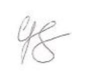	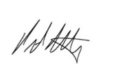	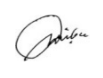	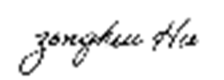

Contents

1. Study objectives and endpoints 196
2. Study populations 197
3. Statistical Analyses 197

3.1 Baseline Descriptive Statistics 197

3.2 Safety Analyses 198

3.3 Efficacy Analyses 198

3.3.1 Time window and comparison arms for efficacy analyses 198

3.3.2 Analysis method 199

3.3.3 Sensitivity analysis 200

3.3.4 Subgroup analysis 201

3.4 Exploratory Analyses 201

3.5 Planned Interim Analyses 202

3.6 Analyses for part 2 extension 202

This document describes the statistical analyses to be carried out on the Kenya L9LS clinical trial study data. Most analyses, except the interim analysis specified below, will be conducted after the database is finalized and locked.

1. Study objectives and endpoints[2]

Primary Objectives and Endpoints :

Data from Part 1, Part 1b, and Part 2 will be used to assess the following objectives and endpoints:

3. Part 1 and Part 2: To evaluate the safety and tolerability of L9LS administered at doses of 5 mg/kg, 10 mg/kg, 20 mg/kg, 30 mg/kg, and 40 mg/kg by SC administration to healthy Kenyan children
   - *Endpoint*: Incidence and severity of local and systemic AEs occurring within 7 days after the administration of L9LS, and incidence of SAEs throughout the study period.
4. Part 2: To assess the efficacy of two doses of L9LS at a concentration of 10-20 mg/kg, administered SC to participants aged 5-59 months of age against first/only Pf malaria infection diagnosed by blood smear microscopy (irrespective of fever) over 12 months compared to placebo
   - *Endpoint:* Pf blood-stage infection as detected by microscopic examination of thick blood smear for 52 weeks after administration of L9LS or placebo.

Secondary Objectives and Endpoints:

Data from Part 2 will be used to assess the following objectives and endpoints:

10. To assess the efficacy of one dose of L9LS at 10-20 mg/kg against first/only Pf malaria infection diagnosed by blood smear microscopy over 3, 6, and 12 months compared to placebo.[3]
    - *Endpoint:* Pf blood-stage infection as detected by microscopic examination of thick blood smear for 12, 24, and 52 weeks after administration of L9LS or placebo.
11. To evaluate the efficacy of L9LS at 10-20 mg/kg against first/only Pf malaria infection as detected by PCR over 12, 24, and 52 weeks compared to placebo.
    - *Endpoint*: Pf blood-stage infection as detected by RT-PCR for 12, 24, and 52 weeks after administration of L9LS or placebo.
12. To assess protection of L9LS at 10-20 mg/kg against clinical malaria (first/only and all episodes) at 3, 6 and 12 months compared to a placebo.

---

[2] Please note that endpoints related to pharmacokinetics (PK) and pharmacodynamics (PD) are not covered in this SAP but will be addressed in a separate document.
[3] The comparator study populations for Secondary Objectives 1, 2, and 3 will be different for the 3- and 6-month analyses than the 12-month analyses. Two-thirds of participants will have received one dose of L9LS by 3- and 6- months and, given the study randomization scheme, only one-third of participants will have received two doses of L9LS by 12 months. The reference group for all these analyses will be the placebo group.

- *Endpoint 1:* Incidence of clinical malaria (defined as an illness accompanied by measured fever ≥37.5°C in the previous 24 hours, and Pf asexual parasitemia >5,000 parasites/μL as detected from microscopic examination of thick blood smear) for 12, 24 and 52 weeks after administration of L9LS or placebo.
- *Endpoint 2:* Incidence of clinical malaria (defined as an illness accompanied by measured fever ≥37.5°C, or history of fever (subjective or objective) and any level of Pf asexual parasitemia for 12, 24 and 52 weeks after administration of L9LS or placebo.

13. To assess the primary objective and all secondary objectives among children 5-17 months of age.
14. To assess the primary objective and all secondary objectives among children 18-59 months of age.

2. Study populations

- Intention-to-treat (ITT) analysis dataset will include all subjects that are randomized and will be analyzed according to the initial randomization assignment.
- Modified intention-to-treat (MITT) analysis dataset will include all randomized subjects that receive the study intervention and will be analyzed according to the initial randomization assignment.
- Per-protocol (PP) analysis dataset will include all randomized subjects that receive the study intervention consistent with the initial randomization assignment and complete the scheduled visits with no major violation and will be analyzed according to the initial randomization assignment.
- As- treated Safety (AS) analysis dataset will include subjects who receive an intervention and be analyzed according to the actual product received.

3. Statistical Analyses

3.1 Baseline Descriptive Statistics

Treatment arms will be compared for baseline subject characteristics using descriptive statistics. For continuous variables, the mean or median will be calculated for each treatment arm. For categorial variables, the proportion under each category will be calculated for each arm.

In general, descriptive statistics will be tabulated by treatment arm for endpoints of interest. This will include point estimates (mean, geometric mean, median, or proportions) and their respective 95% confidence intervals. Formal comparisons will use standard methods, contingency tables for categorical variables, t-tests for comparing means if data follow a normal distribution or geometric means if data after log transformation follow a normal distribution, or

nonparametric analogs for comparing medians. Unless specified in the subsequent sections, comparisons will be two-sided with type I error rate of 0.05.

Missing data will be considered as "missing completely at random" provided the amount of missing data is modest (e.g., <10%). We will examine the "missing completely at random" assumption if the amount of missing data is more than 10%. If the assumption does not hold, missing data will be handled under the "missing at random" assumption (that is, missingness depends only on observed variables) via methods such as multiple imputation and inverse propensity weighting. To handle the possibility of "missing not at random," a sensitivity analysis will be performed by imputing missing binary observations with the observed proportion in the opposite arm. A secondary sensitivity analysis will be considered by imputing missing binary observations as failures.

3.2 Safety Analyses

Safety analysis will be primarily MITT where individuals who receive assignment but do not receive any product are excluded. Because of blinding and the brief length of time between assignment and administration, such cases will be very few. In the rare case of subjects receiving a regimen different from assignment, a safety analysis using an AS cohort will be additionally performed where subjects will be analyzed according to the product actually received.

Safety data will be presented by line listing and in tables at the individual level to provide details on safety events such as severity, duration, and relationship to study product. The number and percentage of subjects with 1 or more AE(s) will be summarized by dose arm along with the exact 95% confidence intervals of the AE rate. For subjects experiencing more than 1 AE, the subjects will be counted once under the event of highest severity.

In Part 2, comparisons between the dose arms and the control arm will be additionally performed in terms of the proportions of participants with solicited AEs, related AEs, and SAEs.

3.3 Efficacy Analyses

The efficacy analyses will be primarily MITT and additionally PP for participants in the efficacy study.

3.3.1 Time window and comparison arms for efficacy analyses

Efficacy objectives and endpoints listed in Section 1 will be assessed and compared between study arms. The primary time point for efficacy assessment will be 52 weeks after monoclonal antibody (mAb)/placebo administration, which will include cases of infection occurring from Day 7 after administration through 52 weeks after administration. The primary comparison arms will be the

two-dose L9LS recipients versus the placebo recipients. Additionally, as a secondary comparison, the one-dose L9LS recipients will be compared with the placebo recipients. The secondary efficacy endpoints are the incidence of malaria infection as determined by RT-PCR and the incidence of clinical malaria.

Efficacy for the primary and secondary endpoints will additionally be assessed at 12 and 24 weeks post MAB/placebo administration. The primary and secondary comparisons will also be conducted separately for the younger age group (5–17 months of age at enrolment) and for the older age group (18–59 months of age at enrollment).

3.3.2 Analysis method

The primary efficacy analysis will be based on time to the first infection. The survival patterns will be described by Kaplan-Meier curves for each arm and compared by the logrank test across different arms. The protective efficacy of the study product will be estimated by the hazard ratio from the Cox proportional hazards model. These analyses will be carried out primarily by R packages that account for interval censoring: package “interval” for deriving a nonparametric maximum likelihood estimation based on the Kaplan-Meier survival curve; package “icenReg” for Cox proportional hazards regression. Efficacy will be calculated as (1 – Hazard Ratio * 100%).

To address the heterogeneity of the study population, a Cox regression with regressors other than the study arm will be additionally performed to account for potential differences among participants. The regressors will include time of enrollment and possibly those covariates that are significantly different between the study product arms and the placebo arm in spite of randomization.

The secondary efficacy analysis will be based on the proportion of infections by study arm. The proportion of infections will be estimated for each arm and compared across arms based on Kaplan-Meier estimates: the proportion of infection by one minus the Kaplan-Meier estimate in the arm, and the L9LS efficacy by one minus the relative risk of infection with melding method for constructing the melded 95% confidence interval of L9LS efficacy.

As an exploratory analysis, multiple cases of infection (and clinical malaria) within a subject will be included in a recurrent event analysis. The Andersen-Gill model, which is based on counting process and an extension of the Cox proportional hazards model, will be fitted. In the Andersen-Gill model, conditional independence is assumed given the timing-varying covariate which is the number of prior infections. In case the Andersen-Gill model fails to converge, a Poisson regression will be used to model the multiple events and to account for the dependence of events within a subject.

If a participant receives antimalarial treatment, he/she will be protected from parasitemia for a period of time following initiation of the treatment; the participant will be excluded from the risk set over the protection period. The 14 days following initiation of treatment with artemether-lumefantrine and 28 days (4 weeks) following initiation of treatment with dihydro-artemisinin-piperaquine will be removed from these analysis. This analysis with adjusted risk set will be conducted based on antimalarial treatments received inside the study clinics where the starting time of antimalarial treatment is recorded, as well as on antimalarial treatment received both inside and outside the study clinics. In the latter case, a self-reported starting date of antimalarial treatment will be used. Fourteen days will also be subtracted after each Pf infection at the study clinic; all participants are supposed to be treated for any study-diagnosed parasitemia, even if asymptomatic, within 72 hours of blood smear reading, so this would simply remove from the time at risk the 1 – 3 days until antimalarial treatment is given. In the rare instance a participant is not treated within 72 hours, any infections diagnosed within 14 days would likely represent the same infection so should not be double counted. This discontinuous risk set analysis will be applied to both the time to first event analysis and recurrent event analysis.

Although the trial will not be weight-stratified, given that we have age stratification, we anticipate that participants will cover the dose range from 10 mg/kg to 20 mg/kg in the efficacy analyses. In addition to the above efficacy analyses that compare each dose arm with the placebo arm, an exploratory analysis may be additionally conducted to assess how dosage, in terms of mg of L9LS per kg of subject body weight, affects the protective efficacy.

3.3.3 Sensitivity analysis

It is possible that pediatric participants may go to non-study facilities (hospitals, clinics, community health volunteers, pharmacies, etc.) for diagnosis and/or treatment of malaria infection. For children who report a malaria diagnosis/treatment at an outside clinic, they will be referred to the study clinic (if this information is disclosed at a home visit) for a finger prick for RDT, blood smear, and filter paper, even if they do not have current malaria symptoms. Two sensitivity analyses on the primary efficacy endpoint, i.e., blood smear Pf infection, will be carried out to deal with the possibility that the reported infection is not detectable by blood smear at the study clinic due to the treatment received at the non-study clinic.

The first sensitivity analysis will be the same as that in Section 3.3.2 except that infection/clinical malaria will be defined as having either a study blood smear positive for Pf infection or a self-

reported infection[4] with a positive RDT done at the study clinic at the same/next visit. For those blood smear negative but RDT positive participants, the reported malaria diagnosis date (at the non-study clinic) will be the considered as the positive test result date, if available, or the date of malaria treatment provided by the non-study facility.

The second sensitivity analysis will be the same as in Section 3.3.2 except that infection/clinical malaria is defined as having either blood smear Pf infection or any self-reported infection[3] with or without a positive RDT at the study clinic. For those with self-reported infection, the self-reported diagnosis date will be considered as the positive test result date, if available, or the date of malaria treatment provided by the non-study facility.

3.3.4 Subgroup analysis

In addition to efficacy analysis comparing all children (both age groups) receiving MAb with those receiving placebo, similar analyses described above will be carried out in the two age groups, 5-17 months of age and 18-59 months of age, separately to compare the younger children receiving MAb to the younger children receiving placebo and the older children receiving MAb to the older children receiving placebo. No multiplicity adjustments will be applied to the subgroup analyses.

3.4 Exploratory Analyses

To explore the impact of L9LS on the genotype of infection -inducing parasites at the CSP locus, a genotypic sieve analysis will be performed to analyze CSP sequences of breakthrough parasites in the blood samples of infected subjects. The sieve analysis will differentiate protective efficacy against different genotypes of infection-inducing parasites with genotype defined by, for example, number of mismatches to the L9LS footprint. To explore whether receipt of MAb affects development of measles-specific antibodies among those who receive the measles vaccine after their first MAb dose, mean and median antibody titers will be compared among subjects <9 months of age at enrollment, prior to and after receipt of MAb. The exploratory analysis comparing one versus two doses of L9LS against Pf infection and clinical malaria will be done as outlined in the **Efficacy Analyses**.

Another exploratory analysis will relate dose group and L9LS concentrations in the blood to development of humoral immunity, through measurements of titers of malaria antibodies, such as CSP, AMA, MSP. Additional exploratory analyses, including those specified in the protocol, might also be conducted.

[4] Self-reported infection will include those whose parents/guardians indicate malaria diagnosis at a non-study facility as well as those who were given antimalarials (from any source) outside the study clinic.

3.5 Planned Interim Analyses

Interim safety analysis will be performed when safety data are available from the age de-escalation and dose escalation study subjects, starting at least 7 days after the last participant in part 1 receives their antibody (or placebo) dose. The purpose of the interim analysis on safety data is to clear safety concerns for proceeding to Part 2.

An interim efficacy analysis will be conducted when clean data are available for all participants through the 6-month endpoint (at 24 weeks) in Part 2. This will assess Pf infection up to 24 weeks by blood smear and clinical malaria using time-to-event analysis and proportional analyses. Having these data available in a timely manner will add to the evidence base needed to plan a potential phase 3 trial of L9LS in settings with seasonal malaria transmission where the time for protection is 6 months. The interim analysis will not impact the conduct of this trial or induce significance level adjustment in the efficacy analyses of this trial.

3.6 Analyses for part 2 extension

The part 2 extension will re-consent participants in part 2 and re-consented participants will continue in their current randomization groups (2 doses L9LS, one dose L9LS (plus one dose placebo), or placebo (two doses). (The doses of L9LS will be higher than in part 2 and will range from 20 – 40 mg/kg.) The analyses for the part 2 extension will follow the analyses for part 2 described above, except the sub-group analyses by age described in section 3.3.4 will not be carried out. An exploratory analysis by age group (as defined in part 2) might be conducted on the part 2 extension data. The efficacy models might include an additional covariate for the actual dose (in mg/kg) of L9LS the participant received during part 2.
